# Genetic and Metabolic Characterization of Insomnia

**DOI:** 10.1371/journal.pone.0018455

**Published:** 2011-04-06

**Authors:** Hyo-Jeong Ban, Sang Cheol Kim, Jungmin Seo, Ho-Bum Kang, Jung Kyoon Choi

**Affiliations:** 1 Division of Bio-Medical Informatics, Center for Genome Science, National Institute of Health, Korea Centers for Disease Control and Prevention (KCDC), Choongchung-Buk-do, Korea; 2 Department of Applied Statistics, Yonsei University, Seoul, Korea; 3 Research Institute of Bioinformatics, Omicsis, Inc., BVC, Korea Research Institute of Bioscience and Biotechnology, Daejeon, Korea; 4 Medical Genomics Research Center, Korea Research Institute of Bioscience and Biotechnology, Daejeon, Korea; 5 Department of Bio and Brain Engineering, KAIST, Daejeon, Korea; 6 Computational and Systems Biology, Genome Institute of Singapore, Singapore, Singapore; Istituto Dermopatico dell'Immacolata, Italy

## Abstract

Insomnia is reported to chronically affect 10∼15% of the adult population. However, very little is known about the genetics and metabolism of insomnia. Here we surveyed 10,038 Korean subjects whose genotypes have been previously profiled on a genome-wide scale. About 16.5% reported insomnia and displayed distinct metabolic changes reflecting an increase in insulin secretion, a higher risk of diabetes, and disrupted calcium signaling. Insomnia-associated genotypic differences were highly concentrated within genes involved in neural function. The most significant SNPs resided in ROR1 and PLCB1, genes known to be involved in bipolar disorder and schizophrenia, respectively. Putative enhancers, as indicated by the histone mark H3K4me1, were discovered within both genes near the significant SNPs. In neuronal cells, the enhancers were bound by PAX6, a neural transcription factor that is essential for central nervous system development. Open chromatin signatures were found on the enhancers in human pancreas, a tissue where PAX6 is known to play a role in insulin secretion. In PLCB1, CTCF was found to bind downstream of the enhancer and interact with PAX6, suggesting that it can probably inhibit gene activation by PAX6. PLCB4, a circadian gene that is closely located downstream of PLCB1, was identified as a candidate target gene. Hence, dysregulation of ROR1, PLCB1, or PLCB4 by PAX6 and CTCF may be one mechanism that links neural and pancreatic dysfunction not only in insomnia but also in the relevant psychiatric disorders that are accompanied with circadian rhythm disruption and metabolic syndrome.

## Introduction

Sleep is a complex physiological process. Genetic determinants underlying sleep phenotypes have only recently begun to be revealed. Reduced sleep has been associated with a mutation in the transcription factor DEC2 in a family-based genetic study [1]. Several quantitative traits related to sleep, such as sleepiness, usual bedtime, and sleep duration, have been examined in a genome-wide association study for 749 subjects [2]. A common sleep disorder, restless legs syndrome, has been characterized by genome-wide association analyses for larger populations [3], [4].

However, little is known about the genetic background of insomnia, one of the most common sleep disorders, which affects 10∼15% of the adult population. In a classic twin study [5], >1,000 monozygotic twins and >800 dizygotic twins were examined in terms of insomnia and sleepiness. Heritability was estimated at 57% for insomnia and 38% for sleepiness. Interestingly, obesity was also under strong genetic influence and shared genetic effects were found between insomnia and obesity [5]. However, the particular genetic contributions common to the two phenotypes remain to be identified.

To study genetic and metabolic aspects of insomnia, we employed the population dataset from the Korea Association Resource (KARE) project. The Ansung and Ansan cohorts, consisting of 5,018 and 5,020 participants ranging in age from 40 to 69 years, were investigated. A genome-wide association analysis has been carried out based on the genotypes of the subjects [6]. In addition, demographic information, medical history and health conditions, family disease history, dietary intake, and lifestyle, were examined as part of the Korean Genome Epidemiology Study (KoGES) [7]. We focused on body composition, and biochemical and anthropometric traits.

## Results and Discussion

The blood cells of the 10,038 subjects were genotyped using the Affymetrix Genome-Wide Human SNP array 5.0. We discarded SNPs with a minor allele frequency <0.01 or a Hardy-Weinberg equilibrium P value <10^−6^. Our quality control and filtering criteria left 81,055 SNPs and 8,842 individuals. Sleeping behavior was examined by questionnaires. The case and control groups were identified based on their answers to questions as to overall insomnia, onset insomnia (difficulty in falling asleep), middle insomnia (difficulty in maintaining sleep or returning to sleep after awakening in the middle of the night), and terminal insomnia (early awakening in the morning). A total of 1,439 respondents reported to have overall insomnia, leaving 7,280 in the control group and 123 in the non-response group. Genotypic differences between the case and control were tested for association by logistic regression analysis with the additive model after adjustment for age and sex.

**Figure 1:**
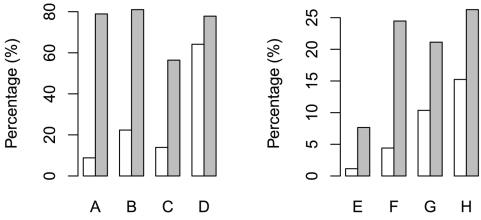
Percentages of sleepers who (A) have difficulty falling asleep, (B) wake up during the night and not be able to fall back asleep, (C) wake up too early in the morning, (D) have irregular sleeping time, (E) have received sleep treatment, (F) have taken sleeping medicine, (G) have the symptoms of restless legs syndrome, and (H) have experienced periodic limb movements during sleep. White bars represent the normal and gray the insomniac group.

A subset of the insomniacs was able to specify their insomnia type. About 78.9%, 81.0%, and 56.4% of the respondents reported onset, middle, and terminal insomnia, respectively (Figs. 1A–1C). Insomnia is known to be 1.4 times more common in women than in men. Our survey confirms this tendency by measuring 21.3% and 11.2% in women and men, respectively. The percentage of those who have irregular sleeping time was marginally higher among the insomniacs (Figure 1D). The insomniacs were ∼6.7 times more likely to have relied on medical treatment (Figures 1E–1F). In addition, 21.1% and 26.3% of the insomniacs complained of restless legs syndrome and periodic limb movement disorder, respectively (Figures 1G–1H).

To observe the physiological phenotypes of insomnia, we examined a total of 12 outputs of body composition analysis as well as 67 biochemical and 41 anthropometric traits. Multiple regression analysis was performed for each variable, controlling for sex and age, to compare the insomniac and normal respondents.

A significantly lower body weight was observed with the insomniacs (Table 1). Body composition analysis supported this, showing lower skeletal muscle mass, total protein mass, and lean body mass (body mass minus fat mass). The insomniacs displayed decreased serum albumin and elevated levels of γ-glutamyl transferase (GGT), insulin, and triglyceride (Table 1). Low serum albumin reflects hepatic dysfunction. Elevated serum GGT and insulin levels can both indicate insulin resistance. In fact, increased insulin secretion was interpreted as a consequence of higher insulin resistance among insomniacs [8]. A higher proportion (8.9%) of the insomniacs have been diagnosed with diabetes as compared to 6.5% of the normal subjects (p = 0.00117). Insulin facilitates the conversion of carbohydrates into fat and increases triglyceride levels, which seems to result in the higher levels of obesity degree (p = 0.01730) among the insomniacs.

**Table 1:** Physiological traits of the insomniac and normal sleepers.

Trait	Unit	Average of insomniacs	Average of non-insomniacs	t value	Pr(>|t|)
Weight	kg	60.463	63.646	−3.509	0.00045
Skeletal muscle mass	kg	40.777	44.201	−3.407	0.00066
Total protein mass	kg	10.889	11.801	−3.368	0.00076
Lean body mass	kg	43.255	46.821	−3.321	0.00090
Serum albumin	g/dL	4.176	4.261	−3.393	0.00069
GGT	IU/L	35.804	35.585	3.539	0.00040
Insulin	µIU/mL	8.150	7.520	3.285	0.00103
Triglyceride	mg/dL	167.657	161.845	2.991	0.00279
Ca^++^	mg/dL	9.651	9.589	5.853	0.00000

Besides the primary circadian clock in the brain, local biological clocks are also found in tissues such as the pancreas and liver. A recent study [9] showed that β cells in the pancreas have their own clock that governs the rhythmic behavior of genes involved in insulin secretion. The knockout of certain clock genes was shown to inhibit insulin secretion and trigger the onset of diabetes [9]. In this respect, the observed metabolic changes in insomnia may stem from genetic problems that simultaneously affect the brain and pancreatic circadian clock.

The insomniacs also displayed higher serum calcium (Table 1). High serum calcium levels are associated with a faster decline in cognitive function [10]. There is a strong correlation between serum and brain calcium levels, owing to free diffusion across the blood-brain barrier [11]. In fact, increased serum calcium leads to calcium accumulation in rat brains [12]. Taken together, higher calcium levels among the insomniacs may reflect a disruption of neuronal calcium homeostasis. Calcium concentration is also important in insulin release from pancreatic β cells [13]. Increased serum calcium may be related to increased insulin levels.

**Figure 2:**
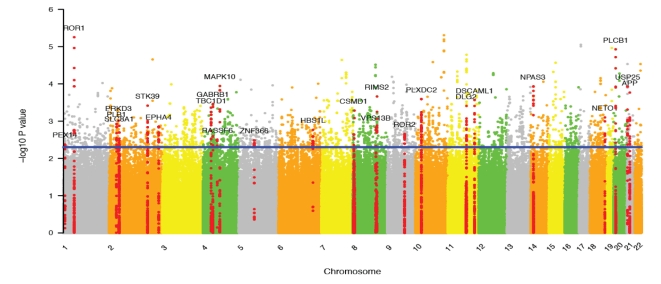
Manhattan plot for genetic association of insomnia. The name of the genes harboring more than 10 significant SNPs is shown at the corresponding chromosomal position. The SNPs residing in these genes are shown as red dots.

We sought to find the underlying genetic causes of insomnia by utilizing the previous genotype data [6]. Our association data for insomnia has been made publicly available at author's website (http://compgen.kaist.ac.kr). All information pertaining to the 3354 SNPs identified at a marginal P-value cutoff (<0.005) is given in Table S1. The name of the genes harbouring more than 10 significant SNPs is shown at the corresponding chromosomal location of the Manhattan plot (Figure 2). Functional analysis of the genes containing at least one of the SNPs showed a remarkable enrichment for neural function (Table S2). For example, the top two functional groups showed enrichments of synaptic genes and another group contained many genes related to neuron differentiation. This implies combinatorial effects of multiple weak genetic determinants in shaping insomniac phenotypes.

The most significant genic SNP (rs11208305, P = 5.6×10^−6^) was found within the ROR1 gene. ROR1 modulates neurite growth and synapse formation in complex with ROR2 [14]. ROR1 and ROR2 harbored 16 and 14 of the identified SNPs, respectively (Table S1 and Figure 2). The second significant SNP (rs718712, P = 8.5×10^−6^) was located within the PLCB1 gene along with 16 other identified SNPs. PLCB1 functions via calcium signaling in long-term depression (Figure S1) and long-term potentiation (Figure S2). Both processes are involved in the molecular basis of learning and memory. CACNA1A, GNAS, NOS3, and ADCY8 in calcium signaling (Figure S3) also harbored a few of the insomniac SNPs. The calcium channel CACNA1A is implicated in several neurological disorders [15] (e.g. it is downregulated in bipolar disorder [16]) and in diabetes as well [17].

**Figure 3:**
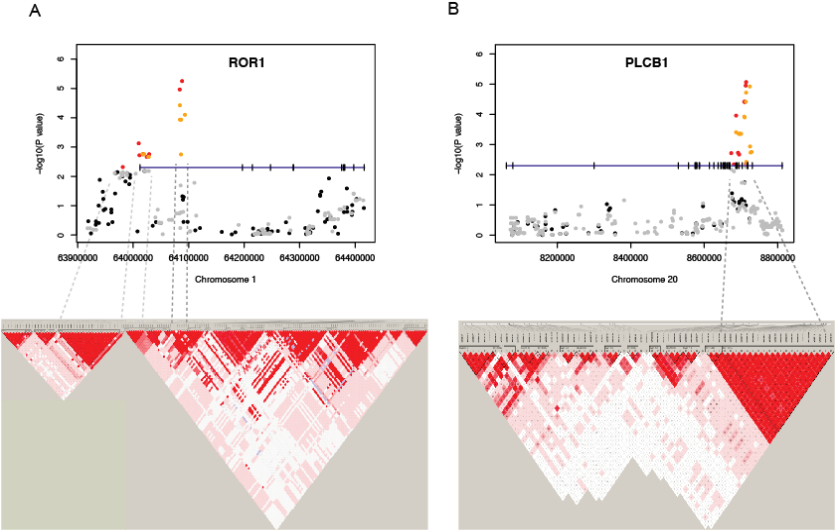
P value plots, genomic structures and LD maps of (A) the ROR1 gene and (B) the PLCB1 gene. The –log10 of the association P value of each SNP was plotted according to its chromosomal position. The gene structure was drawn at P = 5×10-3 with the vertical ticks indicating exons, and the SNPs above this threshold were colored red (experimental) or orange (imputed) and those below it black (experimental) or gray (imputed). The promoter region was attached to the ROR1 genebody to show the SNPs near the transcription start site. The LD maps based on D' were drawn by Haploview using the genotype data of all cases.

The ROR1 promoter was identified to exert high genetic background effects from a genome-wide association study of bipolar disorder [18]. In our study, many SNPs with marginal P values were found in the ROR1 promoter (Figure 3A and Figure S4), including rs7552384 (P = 7.4×10^−4^), 2 kb upstream of the transcription start site. PLCB1, a gene that has long been studied with regard to schizophrenia [19], [20], is reported to also affect sleep-associated theta rhythms [20]. It is worth noting that bipolar disorder and schizophrenia are commonly characterized and genetically linked with each other by circadian rhythm disruption [21], [22], [23], [24]. It has been demonstrated in a large-scale population-based study that these two disorders indeed share common genetic causes [25]. Moreover, these psychiatric disorders are accompanied with metabolic syndrome [26], [27], [28], similar to what we have observed for insomnia.

**Figure 4:**
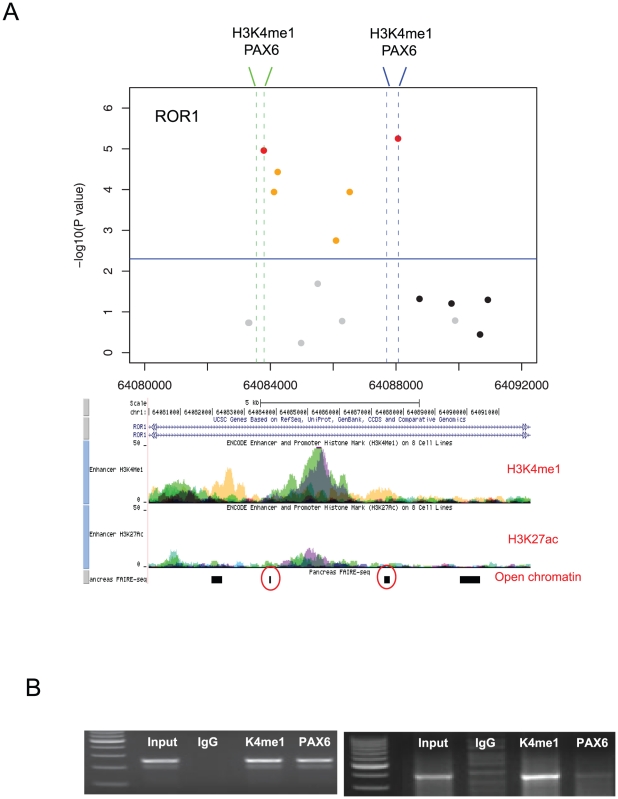
Zoomed-in P value plot and chromatin signatures for the ROR1 region. (A) The vertical dotted lines indicate the DNA regions selected for H3K4me1 and PAX6 ChIP PCR. Below is the UCSC Genome Browser screen for the corresponding region showing H3K4me1 and H3K27ac in multiple cell lines. Open chromatin signatures in human pancreatic islets were employed from published data [34]. (B) The results of ChIP PCR for H3K4me1 and PAX6 in the two regions. Input DNA and IgG antibody were used as positive and negative controls, respectively.

**Figure 5:**
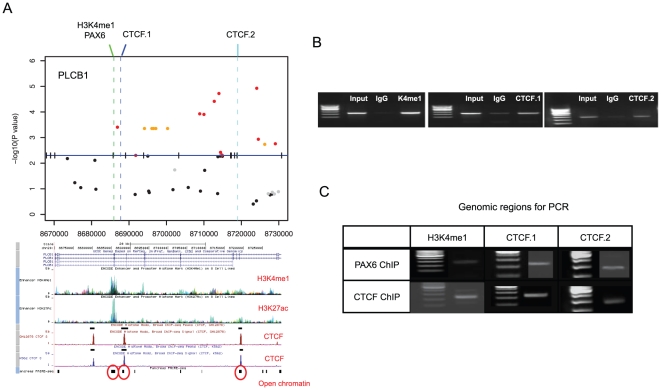
Zoomed-in P value plot and chromatin signatures for the PLCB1 region. (A) The dotted lines indicate the DNA regions selected for H3K4me1, PAX6, and CTCF ChIP PCR. Below is the UCSC Genome Browser screen showing H3K4me1, H3K27ac, and CTCF. Corresponding open chromatin [34] is shown. (B) The results of ChIP PCR for H3K4me1, PAX6, and CTCF. Input DNA and IgG were used as control. (C) Cross ChIP PCR between PAX6 and CTCF. PAX6 ChIP DNA was detected in the two CTCF sites, and CTCF ChIP DNA was detected in the PAX6 site.

We thus focused on the ROR1 and PLCB1 regions (Figure 3). Because the most significant SNPs fell within introns, we suspected a role for regulatory elements. Interestingly, the ENCODE data from multiple cell lines predicted distinct chromatin marks around these SNPs. The ROR1 SNPs flanked a strong peak of H3K4me1 (Figure 4A) and the PLCB1 SNPs resided near an H3K4me1 peak and two CTCF binding sites (Figure 5A). H3K4me1, often with H3K27ac, is a chromatin marker for enhancers [29]. CTCF is an insulator-binding element that can interfere with nearby enhancers.

We experimentally detected H3K4me1 and CTCF in two neuronal cell lines (Figures 4B and 5B). Interestingly, the TRANSFAC database predicted multiple PAX6 and PAX4 binding sites within or near the H3K4me1 marks. PAX6 is known to be coexpressed with PLCB1 and circadian clock genes in the pineal gland [30]. We observed that PAX6 is expressed in one of the two examined cell lines (SK-N-MC) and binds the predicted enhancer of ROR1 (Figure 4B) and PLCB1 (Figures 5C). In the other cell line (SH-SY5Y), there was no signal demonstrating PAX6 binding to ROR1 even with the H3K4me1 mark (Figure S5). We also found that PAX6 and CTCF were interacting across the identified PLCB1 region. The PAX6 protein was detected in the two CTCF sites, and CTCF was detected in the PAX6-bound enhancer (Figure 5C). All the primer sequences used are given in Table S3.

What should be the regulation target of PAX6 and CTCF? Which gene is activated by the enhancer and inhibited by the insulators? The nearest promoter was found ∼300 Kb downstream where the PLCB4 gene is located. Although it is also possible that the PLCB1 gene itself is regulated by PAX6 and CTCF, the fact that PLCB4 is directly related to circadian control makes this gene a more likely candidate. PLCB4 is expressed in tissues in the circadian entrainment pathway including suprachiasmatic nucleus. Based on the sleeping behaviors of PLCB4 knockout mice, it has been reported that this gene plays a role in translating circadian oscillations of the molecular clock into rhythmic outputs of suprachiasmatic nucleus neurons [31].

PAX6 plays a role at the interface of metabolism and neural function. As a neuronal transcription factor that is essential for central nervous system development [32], it also plays a role in pancreatic development and insulin secretion [33]. In the pancreas, it binds to the insulin promoter for activation [33]. We discovered that the binding sites of PAX6 and CTCF in PLCB1 and ROR1 maintain open chromatin structure in the human pancreas [34] (red circles in Figures 4A and 5A). Therefore, it is possible that the identified SNPs in these regions contribute to both the circadian and metabolic phenotypes of insomniacs.

Calcium signaling is important not only for learning and memory in neurons but also for the exocytosis of insulin from pancreatic β cells [13]. Many SNPs were embedded in calcium signaling components, including PLCB1. APP, the precursor of β-amyloid, is also known to stabilize intracellular free calcium levels (reviewed in [35]). A recent report showed a connection between the sleep-wake cycle and the pathogenesis of Alzheimer's disease via β-amyloid regulation [36]. APP was another gene bearing many identified SNPs (Table S1). Surprisingly, the 12 SNPs in APP were also located near H3K4me1 and H3K27ac. The enrichment of H3K4me1 and H3K27ac was confirmed in neural cells (Figure S6).

To understand the effect of the regulatory SNPs in ROR1 and PLCB1 in different sub-populations, we repeated our association analysis within two subgroups of the population as divided by sex, age, insulin level, or calcium level. Distinct differences were found between male and female, and between high-insulin and low-insulin groups.

Interestingly, the ROR1 SNPs were better associated with female insomniacs (Table S4) while the PLCB1 SNPs with male insomniacs (Table S5). Our previous association results were obtained by using sex and age as a covariate, which means that there is no genetic bias between different genders. Hence, a more likely explanation is based on different degrees of penetrance; that is, the insomniac genotypes in PLCB1 exhibit a higher degree of penetrance in males while those in ROR1 are better expressed in females. However, its mechanism remains unclear.

Also, the associations in ROR1 and PLCB1 were relatively better pronounced in the low-insulin group (Tables S6–S7). There are two possibilities. Insomniacs in the high-insulin group are affected by other genetic changes, which may also influence insulin secretion. It is also possible that the normal in these groups have the same genetic changes as the insomniacs but they do not express the associated traits. Given that high insulin levels are often accompanied with insomnia, it is likely that self-reported normal subjects in these groups have potential to develop insomnia.

This is the first large-scale genome-wide association study of sleeplessness that is supported by matched metabolic profiles. Although a replication association study is required, our association results are supported by the high relevance of the identified genes and regulatory factors. First, we find that the genotypic differences are highly concentrated in genes pertaining to neuronal function. Second, the SNPs of interest were present in the regions of high regulatory potential as predicted by public epigenomic data and confirmed experimentally. Third, the identified genes and regulatory factors could provide molecular links to the relevant psychiatric disorders and the observed metabolic signatures. Further studies on the detailed regulatory mechanisms will shed new light on our understanding of insomnia.

## Methods

### Study Population

The population data used in this study are from the Korea Association Resource (KARE) project that entailed a large-scale genome-wide association analysis of 10,038 participants from two regional cohorts. The Ansung and Ansan cohorts, consisting of 5018 and 5020 participants ranging in age from 40 to 69 years, were investigated as part of the Korean Genome Epidemiology Study (KoGES) [7]. More than 260 traits, including demographic information, medical history and health conditions, family disease history, dietary intake, and lifestyle, were extensively examined through epidemiological surveys, physical examinations, and laboratory tests as described, but here we focused on body composition, and biochemical and anthropometric traits. Sleeping patterns were surveyed based on questionnaires. Ethics committee approval and written informed consent have already been obtained in the KoGES [7] and are not required for the present study. The information of the human participants was anonymously used in this study.

### Genome-wide association analysis of case and control groups

The blood cells of the 10,038 subjects were genotyped using the Affymetrix Genome-Wide Human SNP array 5.0. The genotypes from the Affymetrix arrays were determined using the BRLMM algorithm [37]. Low quality SNPs were removed before the case-control study for quality control (quality control method was previously described [6]). In brief, we discarded SNPs with minor allele frequency <0.01 or a Hardy-Weinberg equilibrium P value<10^−6^. The quality control and filtering criteria left out 81,055 SNPs and 8,842 individuals. The case and control groups were identified based on their answers to questions as to overall insomnia, onset insomnia, middle insomnia, and terminal insomnia. A total of 1,439 respondents reported to have overall insomnia, leaving 7,280 in the control group and 123 in the non-response group. Genotypic differences between the case and control were tested for association by logistic regression analysis with the additive model after adjustment for sex and age. A multivariate logistic regression model including sex and age as covariates was implemented using the PLINK software package [38]. The association results are available at http://compgen.kaist.ac.kr/home/data.

### Linkage disequilibrium

Linkage disequilibrium (LD) is the non-random association of two or more loci on a chromosome with recombination between them. The level of linkage disequilibrium is influenced by a number of factors including genetic linkage, selection, the rate of recombination, genetic drift, and population structure. To better facilitate understanding of selected SNPs in disease association studies, we identified common LD blocks in the context of the D' and r(2) linkage disequilibrium. We used the Haploview software (http://www.broadinstitute.org/haploview/) to infer the LD structures in regions containing loci associated with insomnia risk. D' = 1 and LOD >2 were colored bright red.

### SNP Imputation

The genotypes for missing markers in a data set can be confidently inferred by LD and the correlation between genotypes in a reference data set. Association tests of genotypic markers should show similar levels of association compared with imputed markers. We imputed missing SNPs by means of the PLINK program [4] based on the E-M algorithm using the JPT/CHB panel of HapMap reference. After SNP imputation, we included the imputed SNPs for the association study.

### Function and pathway analysis

Functional analysis of genes harboring identified SNPs was done by using DAVID (http://david.abcc.ncifcrf.gov). The ‘Functional Clustering’ function was used to group genes and functions with related terms. The KEGG pathway database (http://www.genome.jp/kegg/pathway.html) was used for the annotation of identified genes.

### Cell culture

Two different human neuroblastoma cell lines, SK-N-MC and SH-SY5Y, were commercially obtained from ATCC (Rockville, MD). Cells were grown in monolayer using DMEM medium (Invitrogen, Carlsbad, CA, USA) and supplemented with 10% fetal bovine serum. They were incubated at 37°C in 5% CO_2_ and plated onto 175 mm culture plates.

### Chromatin Immunoprecipitation and PCR

To investigate histone modification and transcription factor binding, we performed chromatin immunoprecipitation (ChIP) assays using a ChIP assay kit (Millipore, Billerica, MA, USA) according to the manufacturer's protocol. Briefly, formaldehyde was added to culture medium at a final concentration of 1% for 10 min at 25°C, and crosslinking was stopped by incubating in 0.125 M glycine for 5 min. Cells were rinsed, sonicated on ice, and pelleted by centrifugation; supernatant was then collected. Lysates were incubated with Protein G beads with either 5 µg of H3K4me1 (ab8895; abcam), H3K4me3 (ab8580; abcam), H3K27ac (ab4729; abcam), PAX6 (ab5790; abcam), CTCF (ab70303; abcam) or IgG antibody, according to the manufacturer's recommendations. They were rotated overnight at 4°C. Beads were washed, treated with 50 µg proteinase K for 2 h at 55°C, and cross-links were reversed overnight at 65°C. DNA was recovered, ethanol precipitated with glycogen carrier, and resuspended in 10 µl water. PCR amplification was done using 1 µl of ChIP DNA. For ChIP PCR, primers were designed using Primer3 software according to the following criteria: expected amplified fragment size between 150 and 300 bp, primer size between 18 and 25 bases, and primer melting temperature (Tm) between 55 and 60°C. Reverse cross-linked chromatin prior to immunoprecipitation was used as a positive control (input) for PCR amplification.

## Supporting Information

Figure S1 Signaling pathway for long-term depression. The genes identified in this study are marked by a red star, including the PLC protein.
(PNG)

Figure S2 Signaling pathway for long-term potentiation. The genes identified in this study are marked by a red star, including the PLC protein.
(PNG)

Figure S3 Calcium signalling pathway. The pathway for long-term depression. The genes identified in this study are marked by a red star, including the PLCB gene.
(PNG)

Figure S4 P value plot and chromatin signatures for the ROR1 promoter. The –log10 of the association P value of each SNP was plotted according to its chromosomal position. The gene structure was drawn at P = 5×10-3 with the vertical ticks indicating exons, and the SNPs above this threshold were colored red (experimental) or orange (imputed) and those below it black (experimental) or gray (imputed). Below is the UCSC Genome Browser screen for the corresponding region showing H3K4me1, H3K27ac, and H3K4me3 in multiple cell lines.
(PDF)

Figure S5 Same as Figure 4 except for the negative PAX6 ChIP-PCR results when the PAX6(-) cell line was used.
(PDF)

Figure S6 P value plot and chromatin signatures for the APP gene. (A–B) The dotted lines indicate the DNA regions selected for H3K4me1, H3K4me3, and H3K27ac ChIP PCR. Below is the UCSC Genome Browser screen showing H3K4me1, H3K27ac, and corresponding open chromatin in pancreatic cells. (C) The results of ChIP PCR for H3K4me1, H3K4me3, and H3K27ac. Input DNA and IgG were used as controls.
(PDF)

Table S1 Statistical information pertaining to the 3354 SNPs identified at the P-value cutoff of <0.005.
(PDF)

Table S2 Functional analysis of the genes containing at least one of the SNPs as an output of the ‘Functional Clustering’ function of DAVID (http://david.abcc.ncifcrf.gov).
(PDF)

Table S3 Primer sequences used in ChIP-PCR.
(PDF)

Table S4 Differences of P values for the significant SNPs in ROR1 between different sex groups.
(PDF)

Table S5 Differences of P values for the significant SNPs in PLCB1 between different sex groups.
(PDF)

Table S6 Differences of P values for the significant SNPs residing in ROR1 between different insulin levels.
(PDF)

Table S7 Differences of P values for the significant SNPs residing in PLCB1 between different insulin levels.
(PDF)
