# Supplementary figures and images for: Genetic and Metabolic Characterization of Insomnia
Source: PLoS One. 2011 Apr 6;6(4):e18455. doi: 10.1371/journal.pone.0018455 (PMC3071826; doi:10.1371/journal.pone.0018455)

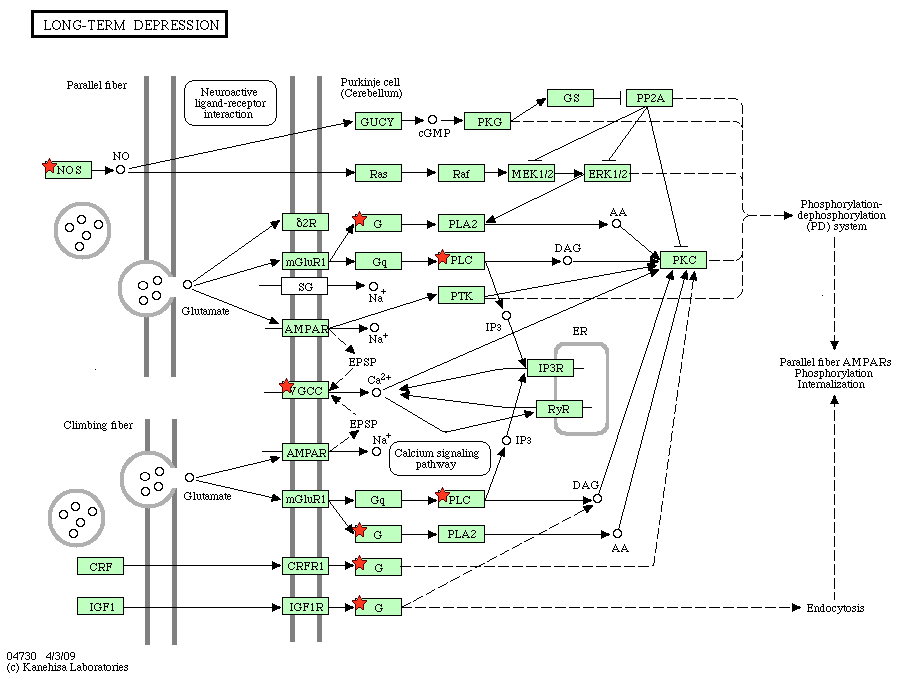

Supplement: Figure S1 — Signaling pathway for long-term depression. The genes identified in this study are marked by a red star, including the PLC protein. (PNG) [file pone.0018455.s001.png]

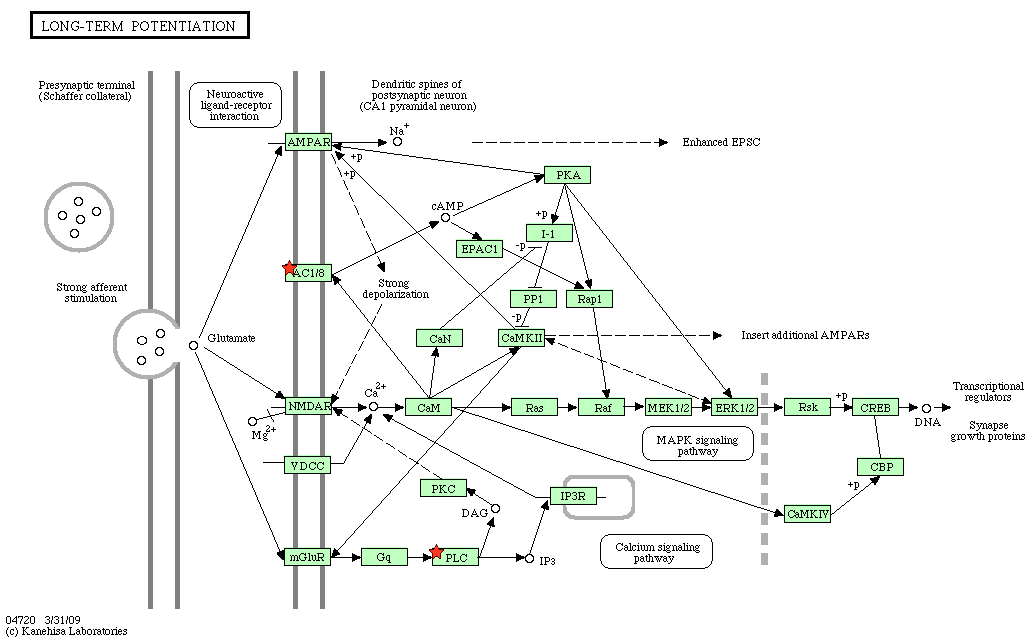

Supplement: Figure S2 — Signaling pathway for long-term potentiation. The genes identified in this study are marked by a red star, including the PLC protein. (PNG) [file pone.0018455.s002.png]

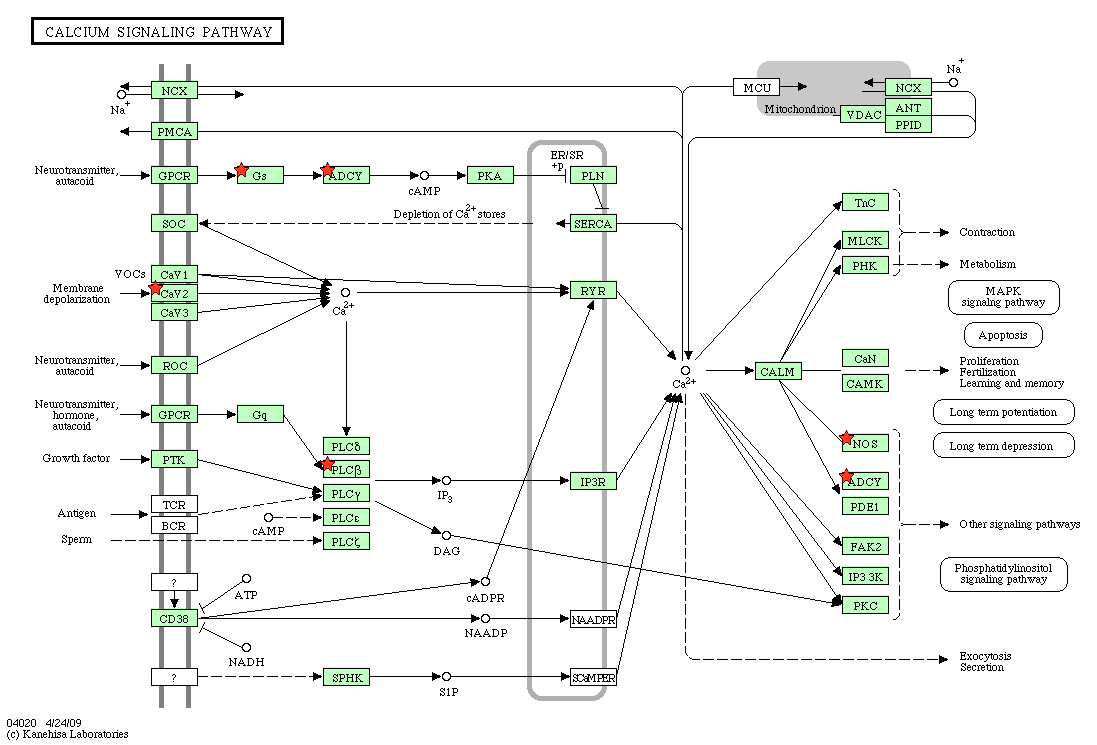

Supplement: Figure S3 — Calcium signalling pathway. The pathway for long-term depression. The genes identified in this study are marked by a red star, including the PLCB gene. (PNG) [file pone.0018455.s003.png]

Figure S4

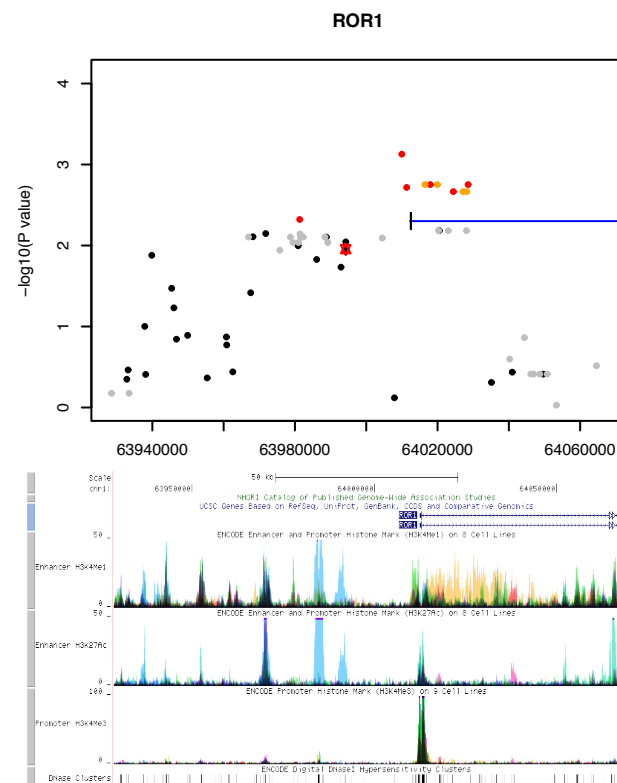

Supplement: Figure S4 — P value plot and chromatin signatures for the ROR1 promoter. The –log10 of the association P value of each SNP was plotted according to its chromosomal position. The gene structure was drawn at P = 5×10^-3^ with the vertical ticks indicating exons, and the SNPs above this threshold were colored red (experimental) or orange (imputed) and those below it black (experimental) or gray (imputed). Below is the UCSC Genome Browser screen for the corresponding region showing H3K4me1, H3K27ac, and H3K4me3 in multiple cell lines. (PDF) [file pone.0018455.s004.pdf]

Figure S5

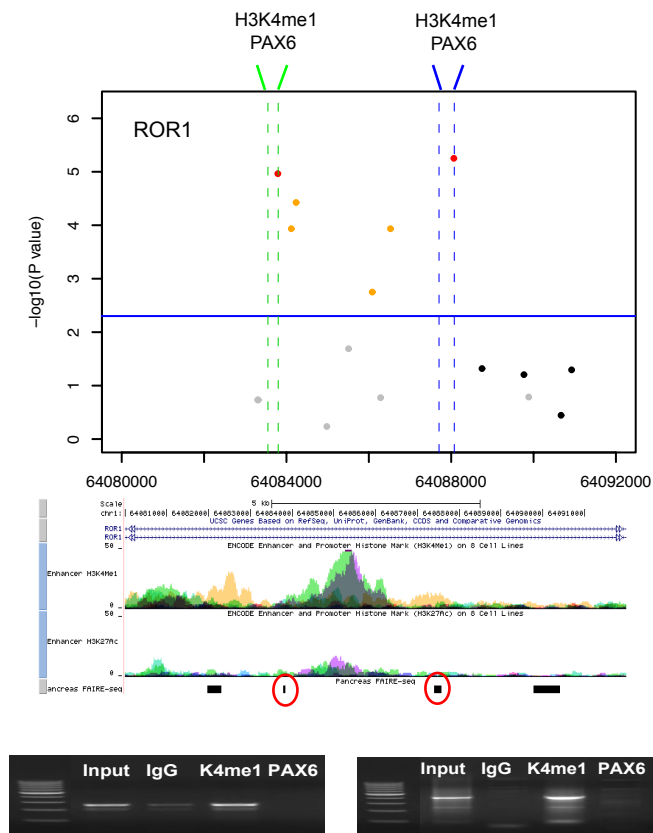

Supplement: Figure S5 — Same as Figure 4 except for the negative PAX6 ChIP-PCR results when the PAX6(-) cell line was used. (PDF) [file pone.0018455.s005.pdf]

Figure S6

A

APP

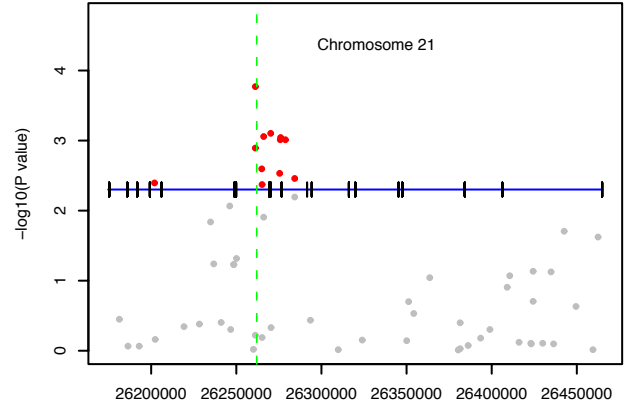

B

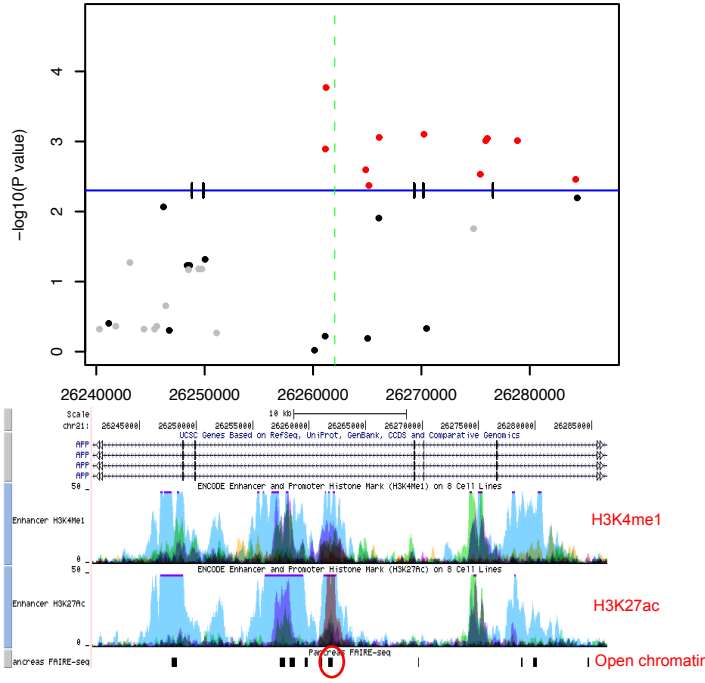

C

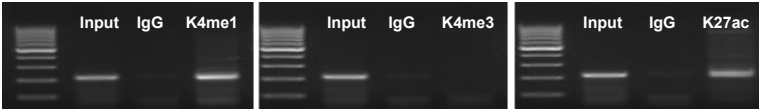

Supplement: Figure S6 — P value plot and chromatin signatures for the APP gene. (A–B) The dotted lines indicate the DNA regions selected for H3K4me1, H3K4me3, and H3K27ac ChIP PCR. Below is the UCSC Genome Browser screen showing H3K4me1, H3K27ac, and corresponding open chromatin in pancreatic cells. (C) The results of ChIP PCR for H3K4me1, H3K4me3, and H3K27ac. Input DNA and IgG were used as controls. (PDF) [file pone.0018455.s006.pdf]
