# Supplementary material for: Genetic and Metabolic Characterization of Insomnia
Source: PLoS One. 2011 Apr 6;6(4):e18455. doi: 10.1371/journal.pone.0018455 (PMC3071826; doi:10.1371/journal.pone.0018455)
Supplement: Table S1 — Statistical information pertaining to the 3354 SNPs identified at the P-value cutoff of <0.005. (PDF) [file pone.0018455.s007.pdf]

rsNum	CHR	BP	OR	STAT	P	A1	A2	GENO	O(HET)	E(HET)	HWE_P	MAF	Gene
rs10915683	1	4896045	0.8886	-2.828	0.004679	G	A	1675/3610/1995	0.4959	0.499	0.5891	0.474	
rs10864350	1	8317383	1.133	2.841	0.004495	A	G	727/3138/3411	0.4313	0.432	0.8921	0.3194	SLC45A1
rs914999	1	8912898	1.251	2.839	0.004521	G	C	26/915/6339	0.1257	0.124	0.2975	0.06875	
rs2268170	1	9238434	0.7616	-3.423	0.0006196	T	C	59/1210/6011	0.1662	0.1658	0.8876	0.08783	H6PD
rs2480781	1	10516128	0.886	-2.819	0.004822	T	A	1188/3397/2671	0.4682	0.4791	0.05289	0.3942	PEX14
rs2480775	1	10529583	0.886	-2.819	0.004822	G	T	1188/3397/2671	0.4682	0.4791	0.05289	0.3942	PEX14
rs710130	1	10529981	0.886	-2.819	0.004822	C	T	1188/3397/2671	0.4682	0.4791	0.05289	0.3942	PEX14
rs4846230	1	10531183	0.8855	-2.835	0.004578	T	C	1185/3417/2678	0.4694	0.479	0.08678	0.3938	PEX14
rs2480773	1	10531677	0.8857	-2.82	0.004809	C	T	1173/3349/2678	0.4651	0.4782	0.02188	0.3919	PEX14
rs12128630	1	10531982	0.8857	-2.82	0.004809	T	C	1173/3349/2678	0.4651	0.4782	0.02188	0.3919	PEX14
rs2480772	1	10532047	0.8852	-2.838	0.00454	C	T	1178/3417/2668	0.4705	0.479	0.135	0.3939	PEX14
rs4846231	1	10535223	0.8857	-2.82	0.004809	A	C	1173/3349/2678	0.4651	0.4782	0.02188	0.3919	PEX14
rs4846232	1	10535397	0.886	-2.819	0.004822	T	C	1188/3397/2671	0.4682	0.4791	0.05289	0.3942	PEX14
rs10157111	1	10535931	0.8857	-2.82	0.004809	G	T	1173/3349/2678	0.4651	0.4782	0.02188	0.3919	PEX14
rs10864463	1	10536147	0.8864	-2.814	0.004895	A	G	1189/3414/2676	0.469	0.4791	0.07411	0.3942	PEX14
rs10779739	1	10537776	0.8853	-2.835	0.004581	C	A	1178/3416/2670	0.4703	0.4789	0.1228	0.3939	PEX14
rs9660006	1	10539859	0.8858	-2.826	0.004712	A	T	1183/3422/2674	0.4701	0.479	0.1118	0.394	PEX14
rs6658073	1	10542552	0.8857	-2.82	0.004809	G	C	1173/3349/2678	0.4651	0.4782	0.02188	0.3919	PEX14
rs6663688	1	10546902	0.8857	-2.82	0.004809	A	G	1173/3349/2678	0.4651	0.4782	0.02188	0.3919	PEX14
rs11121594	1	10549509	0.8845	-2.861	0.00422	G	C	1177/3401/2702	0.4672	0.4781	0.05277	0.3916	PEX14
rs12565494	1	10552149	0.8862	-2.816	0.004863	G	A	1188/3415/2672	0.4694	0.4792	0.08238	0.3944	PEX14
rs6687502	1	10555860	0.8854	-2.834	0.004595	G	A	1179/3426/2674	0.4707	0.4789	0.142	0.3937	PEX14
rs6540938	1	10559046	0.8864	-2.814	0.004895	G	A	1189/3414/2676	0.469	0.4791	0.07411	0.3942	PEX14
rs2086650	1	10563025	0.8862	-2.816	0.004863	C	T	1188/3415/2672	0.4694	0.4792	0.08238	0.3944	PEX14
rs747124	1	10563520	0.8862	-2.816	0.004863	C	G	1188/3415/2672	0.4694	0.4792	0.08238	0.3944	PEX14
rs1201110	1	12075775	0.87	-3.334	0.0008575	G	A	1797/3627/1855	0.4983	0.5	0.7785	0.4905	TNFRSF8
rs4661955	1	13833450	0.7645	-2.908	0.003638	C	G	35/881/6210	0.1236	0.1245	0.5057	0.06387	
rs4661415	1	13836421	0.7645	-2.908	0.003638	A	G	35/881/6210	0.1236	0.1245	0.5057	0.06387	
rs3897424	1	13838205	0.7616	-2.974	0.002944	C	A	34/908/6168	0.1277	0.1278	0.926	0.06562	
rs10927925	1	13838752	0.7645	-2.908	0.003638	C	T	35/881/6210	0.1236	0.1245	0.5057	0.06387	
rs10927926	1	13838805	0.7655	-2.93	0.003386	A	G	36/907/6189	0.1272	0.1278	0.6429	0.06566	
rs11579756	1	14820507	1.14	2.861	0.004229	C	T	646/2852/3314	0.4187	0.4233	0.3749	0.3085	KIAA1026
rs563835	1	18073905	0.8877	-2.851	0.004361	T	C	1798/3611/1801	0.5008	0.5	0.9062	0.4942	
rs1198423	1	23607828	0.7296	-3.452	0.0005562	G	T	15/1009/6228	0.1391	0.133	1.93E-05	0.06921	TCEA3
rs1198439	1	23622180	0.7865	-2.816	0.004861	A	T	30/1059/6180	0.1457	0.1421	0.03147	0.07471	TCEA3
rs1767148	1	23638059	0.7823	-2.831	0.004639	A	G	29/1023/6179	0.1415	0.1383	0.06065	0.0726	ASAP3
rs9659099	1	29914162	1.339	3.223	0.001269	C	A	17/628/6594	0.08675	0.08727	0.5891	0.0479	
rs10799108	1	30753353	1.151	3.053	0.002266	T	C	651/2971/3199	0.4356	0.4302	0.3111	0.3185	
rs2133041	1	30757250	1.134	2.815	0.004879	T	C	651/3044/3579	0.4185	0.419	0.9109	0.3034	

rs942144	1	31011083	1.179	2.939	0.00329	A	C	164/1798/5318	0.247	0.2494	0.3979	0.1492	
rs7517349	1	33467116	1.836	3.538	0.0004032	G	C	2/137/7087	0.01896	0.01932	0.1488	0.01127	
rs3767049	1	37100727	0.792	-3.396	0.0006849	C	T	89/1631/5560	0.224	0.2176	0.0113	0.1204	GRIK3
rs513823	1	37100817	0.7841	-2.879	0.003995	C	T	39/1066/5789	0.1546	0.1522	0.2049	0.0803	GRIK3
rs565537	1	37102574	0.7853	-2.9	0.003727	T	C	41/1091/5782	0.1578	0.1553	0.1881	0.08213	GRIK3
rs506066	1	37104495	0.8045	-2.991	0.002783	T	C	66/1439/5774	0.1977	0.1925	0.02405	0.1046	GRIK3
rs16825217	1	38811756	0.817	-2.875	0.004041	T	C	89/1466/5725	0.2014	0.2003	0.7254	0.1101	
rs4970605	1	38819706	0.7107	-3.722	0.000198	G	A	49/889/6317	0.1225	0.1268	0.006915	0.06532	
rs16830895	1	43753206	1.264	3.269	0.00108	A	C	39/1091/6150	0.1499	0.1477	0.2336	0.08296	
rs6694834	1	53359767	1.135	2.978	0.002903	C	T	1030/3405/2845	0.4677	0.4689	0.8221	0.3797	SLC1A7
rs11206103	1	53361718	1.208	3.074	0.002115	A	G	99/1500/5591	0.2086	0.2083	0.9548	0.1217	SLC1A7
rs3766792	1	53363011	1.19	2.866	0.004156	T	C	103/1584/5591	0.2176	0.2157	0.4798	0.1264	SLC1A7
rs2780293	1	56677660	1.571	3.026	0.00248	A	G	1/205/7074	0.02816	0.02803	1	0.01532	
rs6421495	1	56678033	1.547	2.904	0.003686	T	A	1/204/7075	0.02802	0.0279	1	0.01534	
rs1999537	1	56678112	1.792	3.371	0.0007489	G	A	1/132/7132	0.01817	0.01827	0.4615	0.01049	
rs7537229	1	56678862	1.792	3.371	0.0007489	G	A	1/132/7132	0.01817	0.01827	0.4615	0.01049	
rs7529440	1	56678896	1.752	3.032	0.002431	T	C	0/122/7090	0.01692	0.01677	1	0.00955	
rs11206809	1	56679375	1.745	3.261	0.001109	G	A	1/139/7092	0.01922	0.01931	0.498	0.01104	
rs4411163	1	56680268	1.562	2.965	0.003026	T	C	1/203/7073	0.0279	0.02777	1	0.0154	
rs3892374	1	56681082	1.719	3.047	0.002311	A	G	1/128/7103	0.0177	0.01781	0.4429	0.01016	
rs12087888	1	57575347	0.7993	-3.233	0.001225	C	T	103/1491/5686	0.2048	0.2059	0.6487	0.1131	DAB1
rs11207010	1	57575474	0.811	-3.043	0.00234	C	G	102/1506/5672	0.2069	0.2073	0.8653	0.1142	DAB1
rs17120264	1	60385670	0.8357	-3.129	0.001754	A	G	222/2119/4930	0.2914	0.2904	0.7775	0.1712	
rs11208284	1	63981297	1.155	2.822	0.004778	C	T	281/2300/4670	0.3172	0.3168	0.9409	0.2008	
rs7552384	1	64009992	1.213	3.373	0.0007442	G	C	165/1642/5239	0.233	0.2407	0.008646	0.1431	
rs589465	1	64011312	1.191	3.103	0.001916	A	C	155/1880/5232	0.2587	0.256	0.3838	0.1535	[illegible]R1 LOC6460[illegible]
rs680643	1	64016463	1.2	3.128	0.001763	T	A	137/1724/5162	0.2455	0.244	0.6598	0.1453	ROR1
rs583192	1	64017940	1.2	3.128	0.001763	G	A	137/1724/5162	0.2455	0.244	0.6598	0.1453	ROR1
rs619535	1	64019978	1.2	3.128	0.001763	T	C	137/1724/5162	0.2455	0.244	0.6598	0.1453	ROR1
rs833528	1	64024446	1.195	3.068	0.002157	C	T	137/1735/5232	0.2442	0.2428	0.6601	0.1444	ROR1
rs632076	1	64027114	1.195	3.068	0.002157	A	G	137/1735/5232	0.2442	0.2428	0.6601	0.1443	ROR1
rs647215	1	64028221	1.195	3.068	0.002157	G	A	137/1735/5232	0.2442	0.2428	0.6601	0.1444	ROR1
rs659406	1	64028606	1.2	3.128	0.001763	T	C	137/1724/5162	0.2455	0.244	0.6598	0.1453	ROR1
rs10889450	1	64083788	1.582	4.398	1.09E-05	T	A	4/447/6829	0.0614	0.06055	0.3288	0.03337	ROR1
rs11208300	1	64084120	1.538	3.855	0.0001158	G	A	3/395/6834	0.05462	0.05391	0.3797	0.0295	ROR1
rs679622	1	64084235	1.542	4.123	3.74E-05	A	T	4/450/6798	0.06205	0.06116	0.3315	0.03416	ROR1
rs2132161	1	64086086	1.291	3.125	0.001779	G	T	24/826/6221	0.1168	0.116	0.6083	0.06399	ROR1
rs11208302	1	64086528	1.538	3.855	0.0001158	A	G	3/395/6834	0.05462	0.05391	0.3797	0.0295	ROR1
rs11208305	1	64088067	1.601	4.541	5.60E-06	C	G	4/448/6827	0.06155	0.06068	0.3295	0.03353	ROR1
rs11208306	1	64093324	1.537	3.946	7.95E-05	A	G	2/424/6831	0.05843	0.05724	0.0961	0.0313	ROR1

rs6677577	1	64351938	1.771	2.883	0.003943	A	G	1/106/7148	0.01461	0.01478	0.3305	0.00842	ROR1
rs12026203	1	64606933	1.18	2.836	0.004567	C	T	128/1761/5368	0.2427	0.2393	0.259	0.1428	
rs7539795	1	67382034	0.8689	-3.231	0.001233	A	G	1111/3527/2642	0.4845	0.4779	0.249	0.3888	
rs6588245	1	67384387	0.8686	-3.24	0.001196	C	T	1111/3528/2641	0.4846	0.4779	0.2392	0.3888	
rs790631	1	67449510	0.6963	-2.91	0.003617	C	T	12/525/6729	0.07225	0.0727	0.5206	0.03601	IL23R
rs790634	1	67453516	0.6911	-2.985	0.00284	C	T	11/536/6691	0.07405	0.07412	0.8738	0.03671	IL23R
rs790635	1	67456074	0.6911	-2.985	0.00284	T	C	11/536/6691	0.07405	0.07412	0.8738	0.03671	IL23R
rs17446753	1	67678660	0.661	-2.907	0.003652	G	C	8/427/6845	0.05865	0.059	0.55	0.02888	
rs6696968	1	77582434	1.137	2.934	0.003344	G	A	843/3327/3072	0.4594	0.4526	0.2127	0.3505	AK5
rs12126871	1	78378487	1.163	2.907	0.003653	A	G	252/2134/4890	0.2933	0.2968	0.3046	0.1844	
rs11579434	1	78382610	1.222	3.127	0.001767	C	T	88/1394/5130	0.2108	0.2093	0.5967	0.122	
rs7524877	1	78388233	1.222	3.127	0.001767	G	A	88/1394/5130	0.2108	0.2093	0.5967	0.122	
rs41424046	1	80062223	1.172	3.529	0.0004174	G	A	571/2879/3829	0.3955	0.3998	0.3635	0.2817	
rs6684037	1	81205529	0.8817	-2.999	0.002706	A	G	1490/3531/2256	0.4852	0.4945	0.1122	0.4434	
rs17365096	1	84497934	1.334	3.315	0.0009177	G	A	23/654/6000	0.09795	0.09934	0.2654	0.05527	
rs12047850	1	84501722	1.298	3.073	0.00212	C	A	18/732/6512	0.1008	0.1002	0.7245	0.05534	
rs11161475	1	85118006	0.8735	-2.994	0.002752	A	T	788/3245/3247	0.4457	0.443	0.6151	0.3263	
rs272500	1	86600221	0.838	-3.571	0.0003562	A	T	485/2824/3971	0.3879	0.3854	0.5841	0.2548	ODF2L
rs272514	1	86618151	0.8482	-3.337	0.0008474	T	C	483/2858/3938	0.3926	0.3874	0.2503	0.2573	ODF2L
rs11161808	1	86619396	0.8158	-3.004	0.002662	G	C	102/1588/5589	0.2182	0.2159	0.3854	0.1198	ODF2L
rs3737671	1	86634431	0.8166	-2.985	0.002838	T	G	101/1584/5589	0.2178	0.2154	0.3836	0.1195	ODF2L
rs2791465	1	86681286	1.146	3.208	0.001337	G	A	1109/3433/2738	0.4716	0.475	0.5374	0.3935	CLCA2
rs2249296	1	86682852	1.138	3.001	0.002688	T	C	953/3353/2974	0.4606	0.4615	0.8789	0.3661	CLCA2
rs4314864	1	87519503	0.8509	-3.264	0.0011	A	C	508/2768/3706	0.3964	0.3951	0.8085	0.2668	
rs4556331	1	87519586	0.8509	-3.264	0.0011	C	A	508/2768/3706	0.3964	0.3951	0.8085	0.2668	
rs6675584	1	87525364	0.8603	-3.183	0.001458	C	A	606/2980/3690	0.4096	0.4102	0.909	0.2839	
rs6688319	1	87525474	0.8592	-3.192	0.001415	A	C	582/2950/3748	0.4052	0.4054	0.9769	0.2785	
rs10873827	1	87527272	0.8509	-3.264	0.0011	C	T	508/2768/3706	0.3964	0.3951	0.8085	0.2669	
rs35214987	1	87998917	0.8313	-2.951	0.003167	C	G	165/1777/5336	0.2442	0.2476	0.2366	0.1418	
rs2296622	1	91934166	0.6439	-2.889	0.003864	G	A	7/378/6887	0.05198	0.05245	0.3725	0.02573	TGFBR3
rs659030	1	100140479	0.5461	-2.821	0.004783	A	G	1/225/7054	0.03091	0.0307	1	0.01489	AGL
rs2274569	1	100207667	0.5397	-2.888	0.003878	C	T	3/228/7042	0.03135	0.03166	0.4376	0.01536	
rs12145880	1	100218596	0.5397	-2.888	0.003878	G	C	3/228/7042	0.03135	0.03166	0.4376	0.0153	SLC35A3
rs17121827	1	100310152	0.4654	-3.227	0.001251	G	C	1/215/7043	0.02962	0.02945	1	0.01409	HIAT1
rs4908127	1	101567617	1.141	2.867	0.004144	T	C	521/2898/3847	0.3988	0.3952	0.458	0.2757	
rs7516360	1	101599515	1.141	2.871	0.004097	A	C	521/2898/3848	0.3988	0.3952	0.458	0.2757	
rs7541383	1	101606668	1.14	2.856	0.004291	T	G	521/2909/3848	0.3997	0.3955	0.3741	0.276	
rs11185058	1	107404792	0.8357	-2.99	0.002789	A	G	198/1876/5191	0.2582	0.2638	0.07512	0.1518	
rs961559	1	111714864	0.8496	-2.909	0.003629	C	T	239/2130/4809	0.2967	0.2973	0.8738	0.1774	
rs961557	1	111715110	0.8496	-2.909	0.003629	A	G	239/2130/4809	0.2967	0.2973	0.8738	0.1774	

rs1415384	1	111715425	0.8496	-2.909	0.003629	G	C	239/2130/4809	0.2967	0.2973	0.8738	0.1774	
rs7417712	1	111720141	0.8496	-2.909	0.003629	C	T	239/2130/4809	0.2967	0.2973	0.8738	0.1774	
rs17027560	1	111726283	0.8496	-2.909	0.003629	G	A	239/2130/4809	0.2967	0.2973	0.8738	0.1773	
rs17027569	1	111727959	0.8496	-2.909	0.003629	G	A	239/2130/4809	0.2967	0.2973	0.8738	0.1775	
rs17027572	1	111728332	0.8485	-2.998	0.002713	A	T	271/2201/4808	0.3023	0.3058	0.3379	0.1839	
rs17027578	1	111729417	0.8446	-3.072	0.002123	G	A	266/2205/4809	0.3029	0.3053	0.5139	0.1834	
rs12077338	1	111732439	0.8521	-2.817	0.004843	C	T	239/2012/4852	0.2833	0.2891	0.09232	0.171	LOC441897
rs17027591	1	111732513	0.8499	-2.897	0.003762	T	C	239/2119/4809	0.2957	0.2967	0.7503	0.1769	LOC441897
rs7523084	1	111732614	0.8499	-2.897	0.003762	T	A	239/2119/4809	0.2957	0.2967	0.7503	0.1769	LOC441897
rs7543384	1	111749537	0.86	-2.89	0.003849	C	A	338/2432/4510	0.3341	0.3358	0.6502	0.2086	
rs1343350	1	111751046	0.8605	-2.865	0.004172	A	G	337/2394/4507	0.3308	0.334	0.3985	0.2071	
rs17027794	1	111827972	1.272	2.971	0.002972	A	G	21/831/6428	0.1141	0.1127	0.3482	0.06258	ADORA3
rs4839457	1	115904151	1.132	2.879	0.003987	A	T	903/3314/3044	0.4564	0.4565	0.9795	0.3561	
rs3860222	1	115938333	1.132	2.879	0.003987	C	T	903/3314/3044	0.4564	0.4565	0.9795	0.3561	
rs17024487	1	120255609	0.6074	-2.986	0.002827	G	A	5/333/6817	0.04654	0.04679	0.6078	0.02226	
rs1043964	1	120256618	0.6074	-2.986	0.002827	C	T	5/333/6817	0.04654	0.04679	0.6078	0.02226	NOTCH2
rs7527186	1	120259024	0.6265	-2.828	0.004683	T	C	5/330/6833	0.04604	0.04631	0.6033	0.02211	NOTCH2
rs3795666	1	120260447	0.6254	-2.839	0.004528	A	G	5/331/6832	0.04618	0.04644	0.6046	0.02217	NOTCH2
rs10494233	1	120262439	0.6254	-2.839	0.004528	C	A	5/331/6832	0.04618	0.04644	0.6046	0.02217	NOTCH2
rs17024519	1	120267107	0.6074	-2.986	0.002827	T	A	5/333/6817	0.04654	0.04679	0.6078	0.02226	NOTCH2
rs16835911	1	148545957	0.7844	-3.199	0.001378	C	A	71/1303/5906	0.179	0.1788	1	0.09606	MRPS21
rs16836940	1	148683537	0.7853	-3.181	0.001469	G	A	70/1303/5906	0.179	0.1786	0.8956	0.09601	RPRD2
rs3795702	1	148812248	0.7765	-3.328	0.0008746	T	C	69/1319/5889	0.1813	0.1802	0.649	0.09689	MCL1
rs382436	1	156432285	1.245	2.965	0.003031	T	C	45/997/6016	0.1413	0.1422	0.5586	0.07975	
rs10797059	1	158368880	0.8747	-3.037	0.002388	A	G	923/3264/3093	0.4484	0.4556	0.1809	0.3458	ATP1A2
rs905720	1	160600724	1.157	3.054	0.00226	T	C	405/2633/4242	0.3617	0.3611	0.9224	0.2418	NOS1AP
rs2421707	1	169399875	1.133	2.967	0.003006	G	A	1506/3451/2061	0.4917	0.4969	0.3872	0.4649	
rs10912478	1	169402749	1.133	2.967	0.003006	G	A	1506/3451/2061	0.4917	0.4969	0.3872	0.4649	
rs12134777	1	169405305	1.131	2.936	0.003325	A	T	1479/3667/2134	0.5037	0.496	0.1858	0.4595	
rs7891433	1	169406382	1.133	2.967	0.003006	C	T	1506/3451/2061	0.4917	0.4969	0.3872	0.4649	
rs7887295	1	169406447	1.134	2.984	0.002849	G	A	1506/3454/2050	0.4927	0.497	0.471	0.4657	
rs12136568	1	169413229	1.133	2.995	0.00274	C	A	1507/3642/2131	0.5003	0.4963	0.5085	0.4617	
rs4579718	1	179682417	1.662	3.187	0.001436	C	T	7/152/7121	0.02088	0.02254	3.85E-05	0.01293	
rs12562685	1	182151893	1.125	2.831	0.004638	T	C	1448/3630/2202	0.4986	0.4946	0.507	0.4533	RGL1
rs10489705	1	183860062	0.7112	-2.811	0.004946	A	G	11/546/6705	0.07519	0.07516	1	0.03727	
rs10489707	1	183895507	0.7108	-2.815	0.00487	G	A	11/546/6696	0.07528	0.07525	1	0.03732	
rs12081870	1	183904547	0.7112	-2.811	0.004946	G	A	11/546/6705	0.07519	0.07516	1	0.03727	
rs16824344	1	183908020	0.7025	-2.931	0.003381	C	G	12/558/6686	0.0769	0.07699	0.8784	0.0382	
rs10489711	1	183911286	0.7112	-2.811	0.004946	C	T	11/546/6705	0.07519	0.07516	1	0.03727	
rs12080798	1	183912211	0.7025	-2.931	0.003381	C	T	12/558/6686	0.0769	0.07699	0.8784	0.0382	

rs16824352	1	183912310	0.7025	-2.931	0.003381	T	C	12/558/6686	0.0769	0.07699	0.8784	0.0382	
rs586727	1	184422315	0.4726	-2.899	0.003747	T	C	0/175/7105	0.02404	0.02375	0.6282	0.01108	HMCN1
rs651967	1	184431522	0.3818	-3.033	0.002418	G	A	0/141/7117	0.01943	0.01924	1	0.00876	
rs10798085	1	185333434	0.8169	-3.45	0.0005598	T	G	209/2080/4991	0.2857	0.2843	0.6803	0.1674	
rs118654	1	185336978	0.8145	-3.499	0.0004664	G	T	208/2088/4984	0.2868	0.2848	0.5647	0.1677	
rs230611	1	185360289	0.834	-2.999	0.002707	T	A	192/1885/4766	0.2755	0.2766	0.7267	0.1619	
rs12411137	1	189142632	1.16	2.824	0.00474	T	C	230/2101/4949	0.2886	0.2899	0.6862	0.1797	
rs6690126	1	190274660	0.8864	-2.81	0.004953	C	T	1208/3504/2555	0.4822	0.4828	0.9226	0.4036	
rs4657865	1	190278276	0.8864	-2.81	0.004953	T	C	1208/3504/2555	0.4822	0.4828	0.9226	0.4035	
rs10801086	1	190278693	0.8864	-2.81	0.004953	T	C	1208/3504/2555	0.4822	0.4828	0.9226	0.4035	
rs10921067	1	190279269	0.8864	-2.81	0.004953	A	T	1208/3504/2555	0.4822	0.4828	0.9226	0.4035	
rs6660143	1	190283458	0.8864	-2.81	0.004953	A	G	1208/3504/2555	0.4822	0.4828	0.9226	0.4035	
rs10218711	1	190283844	0.8864	-2.81	0.004953	T	C	1208/3504/2555	0.4822	0.4828	0.9226	0.4035	
rs6428083	1	190285177	0.8864	-2.81	0.004953	G	A	1208/3504/2555	0.4822	0.4828	0.9226	0.4035	
rs12074993	1	190292347	0.8864	-2.81	0.004953	T	C	1208/3504/2555	0.4822	0.4828	0.9226	0.4035	
rs2999593	1	190311996	0.873	-3.114	0.001849	T	C	1113/3401/2602	0.4779	0.4781	0.9802	0.3911	
rs2999597	1	190313631	0.873	-3.114	0.001849	A	G	1113/3401/2602	0.4779	0.4781	0.9802	0.391	
rs3012197	1	190317026	0.8835	-2.878	0.004007	A	T	1146/3515/2618	0.4829	0.4796	0.5576	0.395	
rs10801090	1	190321682	0.8754	-3.036	0.002394	T	C	1115/3358/2538	0.479	0.4794	0.9404	0.3942	
rs10801093	1	190326942	0.8807	-2.899	0.003749	C	T	1136/3379/2493	0.4822	0.4813	0.8816	0.399	
rs1830680	1	192311587	1.131	2.932	0.003373	G	A	1374/3572/2309	0.4924	0.4917	0.9239	0.4403	
rs677459	1	192319137	1.128	2.88	0.00398	C	A	1378/3591/2311	0.4933	0.4918	0.8116	0.4406	
rs2031146	1	192322780	1.128	2.88	0.00398	C	T	1378/3591/2311	0.4933	0.4918	0.8116	0.4405	
rs638023	1	192327045	1.128	2.88	0.00398	T	A	1378/3591/2311	0.4933	0.4918	0.8116	0.4405	
rs7539570	1	196587607	1.167	3.723	0.0001972	A	G	1618/3586/2068	0.4931	0.4981	0.3967	0.4758	
rs1998996	1	196587980	1.166	3.704	0.0002124	G	C	1618/3601/2061	0.4946	0.4981	0.5564	0.4764	
rs6428453	1	196592080	1.136	2.979	0.002887	T	G	1017/3452/2811	0.4742	0.4696	0.4245	0.3816	
rs6702048	1	196593791	1.135	2.945	0.003225	A	G	1012/3472/2795	0.477	0.47	0.2124	0.3823	
rs10922406	1	196595174	1.142	3.111	0.001863	C	T	1027/3437/2815	0.4722	0.4698	0.6898	0.3822	
rs12040677	1	196605235	1.14	3.083	0.002047	A	G	1013/3417/2843	0.4698	0.4683	0.8023	0.379	
rs12084605	1	196607253	1.14	3.083	0.002047	C	T	1013/3417/2843	0.4698	0.4683	0.8023	0.3792	
rs12058799	1	196607714	1.14	3.083	0.002047	T	C	1013/3417/2843	0.4698	0.4683	0.8023	0.3792	
rs12568033	1	196609556	1.14	3.083	0.002047	A	G	1013/3417/2843	0.4698	0.4683	0.8023	0.3792	
rs12062068	1	196610358	1.141	3.093	0.001979	T	C	1025/3436/2815	0.4722	0.4697	0.6714	0.382	
rs7413752	1	196615037	1.135	2.97	0.002974	A	G	1015/3445/2811	0.4738	0.4695	0.4388	0.3813	
rs4501863	1	196615978	1.14	3.083	0.002047	G	A	1013/3417/2843	0.4698	0.4683	0.8023	0.3792	
rs7529123	1	196618419	0.885	-2.885	0.003915	C	T	1275/3436/2544	0.4736	0.4847	0.05264	0.4069	
rs6695684	1	196627120	1.135	2.97	0.002974	T	G	1015/3445/2811	0.4738	0.4695	0.4388	0.3813	
rs4420115	1	196631842	1.166	3.72	0.0001995	T	C	1618/3565/2096	0.4898	0.4978	0.1649	0.474	
rs16842978	1	196635746	1.14	3.069	0.002146	T	A	1019/3418/2843	0.4695	0.4686	0.8807	0.3797	

rs12030985	1	196637277	1.143	3.129	0.001753	G	C	1013/3419/2842	0.47	0.4684	0.783	0.3793	
rs10922416	1	196638640	1.135	2.97	0.002974	A	G	1015/3445/2811	0.4738	0.4695	0.4388	0.3813	
rs7550723	1	196639775	1.143	3.131	0.001739	T	C	1017/3421/2842	0.4699	0.4686	0.8219	0.3798	
rs12075124	1	196645633	1.144	3.167	0.001539	A	C	1015/3419/2846	0.4696	0.4684	0.8413	0.3795	
rs4915286	1	196645692	1.168	3.751	0.000176	A	T	1617/3576/2086	0.4913	0.4979	0.2585	0.4746	
rs12076335	1	196646045	1.14	3.073	0.002119	A	C	1014/3418/2843	0.4698	0.4684	0.8024	0.3792	
rs12404460	1	196662866	1.139	3.093	0.001983	A	G	1537/3402/1976	0.492	0.498	0.3221	0.4747	
rs6665264	1	196672770	1.138	2.849	0.004381	A	G	558/2925/3797	0.4018	0.401	0.8839	0.2825	
rs6427830	1	198731426	0.7727	-2.81	0.004954	C	T	32/890/6358	0.1223	0.1225	0.8482	0.06337	
rs7542210	1	198928838	0.8747	-2.896	0.003779	T	C	675/3043/3548	0.4188	0.4218	0.5407	0.2986	
rs1572993	1	203311710	0.7897	-2.837	0.004561	G	A	52/1071/5865	0.1533	0.154	0.6974	0.08179	CNTN2
rs4142866	1	205322526	0.8543	-2.941	0.003267	C	T	327/2257/4613	0.3136	0.3227	0.01757	0.1988	
rs4403634	1	205334714	0.8628	-2.813	0.004908	A	C	348/2299/4633	0.3158	0.3268	0.004585	0.2026	C4BPB
rs1883774	1	207697379	0.8862	-2.854	0.004316	G	T	1381/3633/2266	0.499	0.4926	0.2738	0.4348	
rs5007029	1	207699677	0.8856	-2.867	0.004146	T	C	1365/3628/2287	0.4984	0.492	0.2732	0.4323	
rs11119280	1	207699801	0.8865	-2.843	0.004474	C	A	1366/3633/2281	0.499	0.4921	0.2338	0.4327	
rs2038184	1	207701946	0.884	-2.905	0.00367	C	T	1354/3633/2293	0.499	0.4917	0.2065	0.431	
rs1022567	1	207703719	0.8868	-2.839	0.00453	T	C	1368/3608/2304	0.4956	0.4917	0.5199	0.4313	
rs6693270	1	207705257	0.8833	-2.924	0.003456	T	C	1352/3633/2295	0.499	0.4916	0.2064	0.4306	
rs7526417	1	207706482	0.8783	-3.064	0.002184	C	G	1354/3605/2321	0.4952	0.4912	0.504	0.4289	
rs10863773	1	207707524	0.8841	-2.903	0.003701	G	C	1355/3630/2295	0.4986	0.4917	0.2333	0.4309	
rs11119284	1	207707712	0.8844	-2.893	0.003811	C	G	1353/3632/2295	0.4989	0.4916	0.2151	0.4308	
rs2206112	1	207708148	0.8828	-2.937	0.003313	T	C	1351/3633/2296	0.499	0.4916	0.198	0.4305	
rs12069966	1	207709424	0.8835	-2.918	0.003518	G	C	1352/3633/2295	0.499	0.4916	0.2064	0.4307	
rs6688435	1	207710809	0.885	-2.88	0.003979	G	A	1350/3630/2299	0.4987	0.4915	0.215	0.4303	
rs4844476	1	207715739	0.8818	-2.957	0.003108	C	G	1345/3641/2294	0.5001	0.4915	0.1393	0.4302	
rs4240836	1	207715753	0.8849	-2.881	0.003967	G	T	1351/3634/2295	0.4992	0.4916	0.1979	0.4307	
rs742254	1	207719569	0.8822	-2.951	0.003164	C	A	1351/3638/2291	0.4997	0.4917	0.1668	0.4309	
rs7525925	1	207723897	0.8691	-3.228	0.001247	A	T	1081/3515/2679	0.4832	0.4759	0.2001	0.3849	
rs10863775	1	207740231	0.8689	-3.231	0.001232	A	T	1081/3517/2679	0.4833	0.4759	0.1917	0.3847	
rs17562778	1	213960182	1.148	2.938	0.003304	T	C	464/2760/4052	0.3793	0.3784	0.8526	0.258	USH2A
rs1436899	1	215124105	0.8349	-2.809	0.004977	G	A	132/1685/5392	0.2337	0.2338	0.9598	0.132	ESRRG
rs17696625	1	215419112	1.651	3.903	9.49E-05	A	G	3/282/6995	0.03874	0.03878	0.7622	0.02155	
rs2646858	1	215558911	0.8528	-3.03	0.002444	T	C	410/2716/3455	0.4127	0.393	4.48E-05	0.2638	
rs2646860	1	215559289	0.8528	-3.03	0.002444	T	C	410/2716/3455	0.4127	0.393	4.48E-05	0.2638	
rs17010005	1	219657373	1.165	2.842	0.00448	G	A	192/2077/5001	0.2857	0.2812	0.1821	0.1728	
rs17010272	1	219801664	1.298	3.246	0.001169	C	T	44/717/6063	0.1051	0.111	6.68E-05	0.06214	
rs17041848	1	219803723	1.277	3.018	0.002547	G	A	44/709/6040	0.1044	0.1104	3.64E-05	0.06167	
rs17041852	1	219803863	1.277	3.018	0.002547	T	C	44/709/6040	0.1044	0.1104	3.64E-05	0.06167	
rs17041853	1	219803977	1.277	3.018	0.002547	G	T	44/709/6040	0.1044	0.1104	3.64E-05	0.06168	

rs17661530	1	219804140	1.277	3.018	0.002547	C	A	44/709/6040	0.1044	0.1104	3.64E-05	0.06167	
rs12401875	1	219806433	1.277	3.018	0.002547	A	G	44/709/6040	0.1044	0.1104	3.64E-05	0.06168	
rs17010290	1	219807897	1.277	3.018	0.002547	T	C	44/709/6040	0.1044	0.1104	3.64E-05	0.06167	
rs12744344	1	219884304	1.154	2.95	0.003174	T	C	445/3021/3463	0.436	0.4051	1.71E-10	0.287	
rs6668249	1	222965456	0.8601	-2.959	0.003091	C	T	383/2733/4157	0.3758	0.3654	0.01605	0.2371	CNIH3
rs9793705	1	227327134	0.8871	-2.879	0.003987	G	A	1628/3615/2021	0.4977	0.4985	0.8877	0.4685	
rs853459	1	228792447	0.8808	-2.988	0.002812	A	G	1421/3440/2253	0.4836	0.4932	0.1021	0.4369	LOC729257
rs12410954	1	229310379	1.202	3.079	0.00208	T	G	107/1608/5563	0.2209	0.219	0.4865	0.129	
rs9787273	1	230396036	1.138	3.09	0.002002	A	G	1531/3649/2100	0.5012	0.4969	0.4792	0.4676	
rs10752796	1	230397242	1.137	3.076	0.002099	T	G	1528/3642/2100	0.501	0.4969	0.4937	0.4674	
rs7555102	1	230398089	1.137	3.076	0.0021	C	T	1528/3643/2100	0.501	0.4969	0.4937	0.4674	
rs7418143	1	230404992	1.137	3.076	0.002099	C	T	1528/3642/2100	0.501	0.4969	0.4937	0.4674	
rs6605042	1	230413462	1.137	3.076	0.002099	T	G	1528/3642/2100	0.501	0.4969	0.4937	0.4672	
rs10752787	1	230417406	0.8803	-2.837	0.004558	A	G	816/3135/3329	0.4306	0.4404	0.05883	0.3217	
rs10797563	1	230417445	0.8802	-2.839	0.004532	T	C	813/3140/3327	0.4313	0.4404	0.07904	0.3216	
rs16839314	1	238240106	1.192	3.698	0.0002169	T	C	378/2582/4320	0.3547	0.3534	0.7908	0.2349	
rs6698474	1	238361987	1.14	2.875	0.004044	A	G	551/2996/3733	0.4115	0.4045	0.1395	0.2845	FMN2
rs1005838	1	239678207	1.163	3.463	0.0005335	G	C	1015/3429/2598	0.4869	0.4747	0.03286	0.3943	
rs2994978	1	239700036	1.143	3.164	0.001558	C	A	1085/3442/2748	0.4731	0.4739	0.9015	0.3922	
rs2994979	1	239700354	1.153	3.292	0.0009931	C	A	1085/3380/2532	0.4831	0.4786	0.4536	0.4032	
rs7527610	1	244679756	1.556	3.121	0.001802	C	T	3/230/7047	0.03159	0.03189	0.4414	0.01758	
rs12617403	2	4815810	0.8169	-3.552	0.0003817	T	C	229/2192/4859	0.3011	0.2978	0.3652	0.1773	
rs11692765	2	4826227	0.8176	-3.539	0.0004018	T	C	230/2189/4861	0.3007	0.2977	0.4083	0.1773	
rs6419561	2	4833817	0.8358	-3.345	0.0008217	T	C	302/2415/4563	0.3317	0.3287	0.454	0.2024	
rs6753305	2	4853310	0.8084	-3.785	0.0001539	T	G	243/2262/4775	0.3107	0.3062	0.2207	0.1837	
rs1371473	2	4856019	0.8233	-3.665	0.0002473	C	T	315/2532/4433	0.3478	0.34	0.05352	0.2117	
rs17020172	2	4884942	0.8212	-3.525	0.0004242	C	T	253/2236/4789	0.3072	0.3058	0.7016	0.1835	
rs10495513	2	4894814	0.8394	-3.21	0.001329	A	G	290/2203/4787	0.3026	0.3092	0.06889	0.1867	
rs41520250	2	4903432	0.8216	-3.509	0.0004506	T	A	248/2239/4791	0.3076	0.3052	0.5138	0.1835	
rs1371485	2	4907095	0.8246	-3.439	0.0005847	A	G	243/2234/4803	0.3069	0.3038	0.4178	0.1822	
rs17293732	2	5519735	1.178	3.023	0.002499	C	T	193/2002/5085	0.275	0.2742	0.8309	0.1676	
rs792094	2	5521617	1.179	3.043	0.002346	C	G	193/1999/5085	0.2747	0.274	0.8642	0.1675	
rs792090	2	5524884	1.137	2.87	0.004108	T	A	634/2965/3622	0.4106	0.4144	0.4432	0.2965	
rs792043	2	5527433	1.137	2.87	0.004108	C	A	634/2965/3622	0.4106	0.4144	0.4432	0.2966	
rs10194520	2	5529230	1.137	2.871	0.004096	G	A	634/2965/3630	0.4102	0.4141	0.4103	0.2963	
rs6743805	2	5530261	1.179	3.043	0.002346	A	T	193/1999/5085	0.2747	0.274	0.8642	0.1675	
rs17360123	2	5531665	1.168	2.827	0.004703	T	G	185/1961/5085	0.2712	0.2704	0.8279	0.1645	
rs2722596	2	5819377	1.19	3.054	0.002261	A	G	154/1703/5423	0.2339	0.2381	0.1396	0.1416	
rs2163030	2	5829023	1.186	2.975	0.002932	T	C	147/1697/5436	0.2331	0.2361	0.2749	0.1399	
rs2564068	2	5830153	1.185	2.953	0.003145	C	A	147/1700/5433	0.2335	0.2364	0.2978	0.1401	

rs1429222	2	5838153	1.176	2.832	0.00462	G	T	149/1703/5428	0.2339	0.2371	0.2555	0.1405	
rs1560238	2	5843308	1.138	2.959	0.003089	C	T	759/3033/3453	0.4186	0.4309	0.0164	0.3185	
rs16864204	2	5844196	1.138	2.959	0.003089	T	C	759/3033/3453	0.4186	0.4309	0.0164	0.3186	
rs1346608	2	5844370	1.181	2.898	0.003761	T	G	148/1698/5434	0.2332	0.2364	0.2542	0.1401	
rs16864209	2	5845544	1.133	2.856	0.004296	T	C	762/3059/3459	0.4202	0.4314	0.02774	0.3191	
rs10495524	2	5848753	1.14	2.992	0.00277	G	A	759/3035/3450	0.419	0.431	0.01771	0.3188	
rs7425362	2	5849348	1.136	2.932	0.003366	G	A	761/3060/3459	0.4203	0.4313	0.02973	0.3191	
rs10803749	2	5851296	1.135	2.907	0.003651	C	T	761/3064/3455	0.4209	0.4315	0.03648	0.3194	
rs2350199	2	5860682	1.183	2.842	0.004477	C	T	121/1680/5107	0.2432	0.2395	0.2095	0.1422	
rs2564006	2	5900776	0.8819	-2.821	0.004784	T	G	839/3098/3343	0.4255	0.4408	0.003164	0.3236	
rs4315493	2	7547099	0.8869	-2.885	0.00392	G	C	1742/3545/1943	0.4903	0.4996	0.1148	0.4804	
rs10495556	2	7548391	0.8869	-2.885	0.00392	C	T	1742/3545/1943	0.4903	0.4996	0.1148	0.4804	
rs10495557	2	7548910	0.8898	-2.826	0.004716	T	C	1778/3552/1943	0.4884	0.4997	0.05434	0.4831	
rs6741211	2	7549527	0.887	-2.883	0.003941	T	C	1742/3552/1943	0.4908	0.4996	0.1383	0.4804	
rs9287692	2	7550469	0.887	-2.883	0.003941	A	G	1742/3552/1943	0.4908	0.4996	0.1383	0.4804	
rs6718029	2	7568437	1.142	2.881	0.00397	C	G	637/2625/3289	0.4007	0.4181	0.0008362	0.3023	
rs6740480	2	7570218	1.142	2.881	0.00397	A	C	637/2625/3289	0.4007	0.4181	0.0008362	0.3022	
rs6727796	2	7572649	1.142	2.881	0.00397	A	G	637/2625/3289	0.4007	0.4181	0.0008362	0.3023	
rs6431885	2	7573891	1.142	2.881	0.00397	T	C	637/2625/3289	0.4007	0.4181	0.0008362	0.3023	
rs7558848	2	12210576	0.7241	-3.824	0.0001316	C	T	51/1130/6099	0.1552	0.1549	0.9397	0.08078	
rs11689406	2	13814514	1.228	3.133	0.00173	A	G	66/1349/5830	0.1862	0.1835	0.2487	0.1049	
rs10166106	2	13824391	1.361	3.125	0.00178	G	C	4/545/6729	0.07488	0.0731	0.0354	0.03992	
rs6432412	2	13825283	1.356	3.089	0.002005	G	A	4/547/6729	0.07514	0.07333	0.03565	0.04002	
rs1470494	2	13826960	1.374	3.202	0.001365	C	A	3/540/6729	0.07426	0.07226	0.01377	0.0395	
rs7599789	2	13836056	1.372	3.204	0.001355	A	C	4/539/6736	0.07405	0.07232	0.04885	0.03953	
rs6724022	2	16659386	1.164	2.957	0.00311	T	A	259/2237/4784	0.3073	0.3068	0.9391	0.1931	FAM49A
rs35807106	2	17921874	1.407	2.848	0.004405	A	G	4/352/6814	0.04909	0.04895	1	0.02656	
rs9973485	2	18131458	1.128	2.809	0.004966	G	C	1000/3321/2948	0.4569	0.4641	0.1888	0.3707	
rs7602668	2	19302600	0.858	-2.983	0.002853	C	G	379/2594/4306	0.3564	0.3545	0.6673	0.2253	
rs6704992	2	28698462	0.8618	-2.961	0.003064	A	G	494/2654/3443	0.4027	0.3999	0.6005	0.2722	PLB1
rs11684628	2	28701138	0.8673	-3.077	0.002088	A	G	681/3143/3456	0.4317	0.4274	0.3952	0.3049	PLB1
rs6729040	2	28704049	0.8618	-2.961	0.003064	T	G	494/2654/3443	0.4027	0.3999	0.6005	0.2722	PLB1
rs6713845	2	28708074	0.85	-3.241	0.001189	C	T	468/2771/3636	0.4031	0.3938	0.05371	0.2653	PLB1
rs2199619	2	28708462	0.85	-3.241	0.001189	T	C	468/2771/3636	0.4031	0.3938	0.05371	0.2654	PLB1 PLB1
rs3752901	2	28709551	0.8608	-3.184	0.001453	C	T	609/3035/3636	0.4169	0.4136	0.5145	0.2879	PLB1
rs6719289	2	28710305	0.8747	-2.858	0.004263	G	A	616/2896/3768	0.3978	0.4063	0.07841	0.2796	PLB1
rs11127185	2	28714571	0.8356	-3.195	0.001397	A	G	258/2146/4876	0.2948	0.2988	0.2553	0.1786	PLB1
rs11679188	2	28716990	0.861	-2.982	0.002867	A	G	425/2768/3918	0.3893	0.3794	0.02864	0.2503	PLB1
rs11894900	2	28719026	0.8804	-2.847	0.004416	G	C	863/3284/2958	0.4622	0.4565	0.2987	0.3484	PLB1
rs2272386	2	28719973	0.8758	-2.949	0.003185	C	A	852/3245/2958	0.46	0.4554	0.4177	0.3466	PLB1

rs6738334	2	28720432	0.8781	-2.937	0.003311	G	A	902/3408/2970	0.4681	0.4597	0.1199	0.3539	PLB1
rs6741437	2	28720705	0.8804	-2.847	0.004416	G	A	863/3284/2958	0.4622	0.4565	0.2987	0.3485	
rs2049690	2	28720910	0.878	-2.886	0.003904	G	T	839/3245/2958	0.4608	0.4547	0.2711	0.3454	
rs899156	2	28721527	0.8804	-2.847	0.004416	T	G	863/3284/2958	0.4622	0.4565	0.2987	0.3485	
rs2126890	2	28725743	0.8714	-2.819	0.004822	T	C	490/2833/3788	0.3984	0.3925	0.2153	0.2642	
rs755591	2	28726716	0.8672	-2.994	0.002754	T	C	556/2936/3788	0.4033	0.4015	0.7044	0.274	
rs17007156	2	28727596	0.8643	-2.971	0.002966	C	G	488/2806/3788	0.3962	0.3914	0.3165	0.2629	
rs12477114	2	28728621	0.8694	-2.868	0.004137	A	C	495/2835/3788	0.3983	0.393	0.2645	0.2647	
rs6753015	2	28729177	0.8589	-3.102	0.001921	T	A	495/2808/3774	0.3968	0.3927	0.3966	0.2641	
rs11678098	2	28730361	0.8694	-2.868	0.004137	G	A	495/2835/3788	0.3983	0.393	0.2645	0.2647	
rs11694841	2	28730510	0.8694	-2.868	0.004137	T	C	495/2835/3788	0.3983	0.393	0.2645	0.2647	
rs6547865	2	28731089	0.8615	-3.137	0.001707	G	A	564/2942/3774	0.4041	0.4028	0.7934	0.2753	
rs11684670	2	28731286	0.8694	-2.868	0.004137	C	G	495/2835/3788	0.3983	0.393	0.2645	0.2647	
rs2061757	2	28739384	0.8795	-2.877	0.004011	A	G	864/3285/3013	0.4587	0.455	0.4996	0.3464	
rs7594379	2	28758905	0.8807	-2.85	0.004369	G	C	1201/3469/2079	0.514	0.4915	0.0001841	0.4315	
rs2687209	2	33968307	0.8855	-2.876	0.004032	A	G	1665/3588/1944	0.4985	0.4992	0.906	0.4752	
rs2461614	2	33968365	0.8855	-2.876	0.004032	G	C	1665/3588/1944	0.4985	0.4992	0.906	0.4752	
rs17014268	2	33968702	0.876	-3.125	0.001777	G	A	1624/3659/1985	0.5034	0.4988	0.4377	0.4693	
rs773823	2	33968755	0.8832	-2.94	0.003279	T	C	1674/3661/1945	0.5029	0.4993	0.5573	0.4758	
rs773820	2	33970192	0.8843	-2.908	0.003638	G	T	1665/3659/1945	0.5034	0.4993	0.4957	0.4753	
rs10495795	2	33970845	0.876	-3.125	0.001777	T	C	1624/3659/1985	0.5034	0.4988	0.4377	0.4693	
rs811790	2	33971092	0.8843	-2.908	0.003638	G	C	1665/3659/1945	0.5034	0.4993	0.4957	0.4753	
rs2461615	2	33971808	0.8843	-2.908	0.003638	G	A	1665/3659/1945	0.5034	0.4993	0.4957	0.4753	
rs1510874	2	33972748	0.8843	-2.908	0.003638	T	G	1665/3659/1945	0.5034	0.4993	0.4957	0.4753	
rs10495799	2	33972928	0.8747	-3.164	0.001558	A	G	1634/3661/1985	0.5029	0.4988	0.4957	0.47	
rs1705143	2	33973136	0.8843	-2.908	0.003638	A	G	1665/3659/1945	0.5034	0.4993	0.4957	0.4752	
rs7575904	2	33974297	0.876	-3.125	0.001777	C	T	1624/3659/1985	0.5034	0.4988	0.4377	0.4693	
rs10194487	2	33974964	0.876	-3.125	0.001777	C	T	1624/3659/1985	0.5034	0.4988	0.4377	0.4693	
rs10182691	2	33975512	0.8785	-3.084	0.002043	C	G	1704/3617/1949	0.4975	0.4994	0.7424	0.4775	
rs4410270	2	34021125	0.8628	-2.825	0.004728	G	A	343/2475/4462	0.34	0.3399	1	0.2131	
rs1567513	2	34022314	0.8634	-2.81	0.004957	C	T	341/2472/4462	0.3398	0.3396	0.9725	0.2128	
rs12712404	2	34873191	1.171	3.46	0.0005396	C	A	515/2879/3886	0.3955	0.3928	0.5709	0.2738	
rs2438428	2	34885495	1.176	3.392	0.0006944	A	G	422/2636/3893	0.3792	0.3753	0.406	0.2555	
rs2456800	2	34885592	1.167	3.215	0.001304	G	T	418/2602/3928	0.3745	0.3724	0.6521	0.2524	
rs2438429	2	34885619	1.176	3.392	0.0006944	C	T	422/2636/3893	0.3792	0.3753	0.406	0.2555	
rs10191460	2	34903582	1.156	3.141	0.001682	G	C	492/2780/4008	0.3819	0.3834	0.7367	0.263	
rs383473	2	34904496	1.172	3.395	0.0006851	A	G	498/2856/3754	0.4018	0.3951	0.1582	0.2754	
rs6733633	2	34905612	1.165	3.255	0.001132	A	C	471/2729/3932	0.3826	0.3823	0.9506	0.2619	
rs11680038	2	34906744	1.165	3.255	0.001132	G	A	471/2729/3932	0.3826	0.3823	0.9506	0.2619	
rs13417686	2	34910939	1.169	3.226	0.001254	G	C	390/2743/3932	0.3883	0.3743	0.001832	0.254	

rs280709	2	34911029	1.164	3.316	0.0009129	T	C	510/2824/3944	0.388	0.3887	0.8802	0.269	
rs7600597	2	34912263	1.165	3.255	0.001132	C	T	471/2729/3932	0.3826	0.3823	0.9506	0.2619	
rs280711	2	34913041	1.172	3.395	0.0006851	C	G	498/2856/3754	0.4018	0.3951	0.1582	0.2755	
rs280713	2	34913353	1.172	3.395	0.0006851	A	T	498/2856/3754	0.4018	0.3951	0.1582	0.2755	
rs280726	2	34922863	1.167	3.246	0.00117	T	G	471/2726/3734	0.3933	0.3892	0.3876	0.2692	
rs280691	2	34927111	1.153	3.136	0.001714	C	T	759/3089/2893	0.4582	0.4499	0.1296	0.3468	
rs280692	2	34927213	1.154	3.146	0.001657	G	C	758/3089/2893	0.4583	0.4498	0.1294	0.3469	
rs280694	2	34927659	1.135	3.023	0.002504	G	A	1149/3441/2690	0.4727	0.4776	0.3771	0.3989	
rs280696	2	34929144	1.16	3.486	0.0004908	A	T	1009/3344/2893	0.4615	0.4662	0.3916	0.3754	
rs280697	2	34929349	1.16	3.486	0.0004908	T	C	1009/3344/2893	0.4615	0.4662	0.3916	0.3754	
rs280698	2	34929506	1.157	3.455	0.0005506	T	A	1022/3355/2902	0.4609	0.4666	0.3031	0.3763	
rs2254958	2	37229795	0.8359	-3.146	0.001655	G	A	229/2152/4899	0.2956	0.2942	0.72	0.1756	EIF2AK2
rs12620426	2	37233373	0.8434	-2.954	0.003135	T	A	217/2088/4975	0.2868	0.2864	0.9348	0.1699	EIF2AK2
rs13429526	2	37320608	1.132	2.851	0.004357	G	C	819/3285/3174	0.4514	0.4476	0.496	0.3423	C2orf56
rs2041838	2	37321952	1.132	2.851	0.004357	G	T	819/3285/3174	0.4514	0.4476	0.496	0.3424	C2orf56
rs2287092	2	37334518	1.13	2.808	0.004985	A	T	826/3288/3148	0.4528	0.4489	0.4803	0.3443	PRKD3
rs2300891	2	37345170	1.132	2.843	0.004475	C	G	820/3285/3174	0.4513	0.4477	0.5128	0.3425	PRKD3
rs9309003	2	37346386	1.13	2.808	0.004985	A	T	826/3288/3148	0.4528	0.4489	0.4803	0.3443	PRKD3
rs2300888	2	37347840	1.13	2.808	0.004985	G	A	826/3288/3148	0.4528	0.4489	0.4803	0.3443	PRKD3
rs2300886	2	37349466	1.13	2.815	0.004883	C	T	825/3289/3148	0.4529	0.4488	0.4484	0.3443	PRKD3
rs13390055	2	37351956	1.13	2.815	0.004883	G	A	825/3289/3148	0.4529	0.4488	0.4484	0.3443	PRKD3
rs10460527	2	37358609	1.13	2.815	0.004883	G	T	825/3289/3148	0.4529	0.4488	0.4484	0.3443	PRKD3
rs13421912	2	37359160	1.13	2.815	0.004883	T	C	825/3289/3148	0.4529	0.4488	0.4484	0.3442	PRKD3
rs1160516	2	37359732	1.13	2.815	0.004883	T	C	825/3289/3148	0.4529	0.4488	0.4484	0.3443	PRKD3
rs3770761	2	37360443	1.13	2.815	0.004883	G	A	825/3289/3148	0.4529	0.4488	0.4484	0.3443	PRKD3
rs11887618	2	37361332	1.13	2.815	0.004883	C	T	825/3289/3148	0.4529	0.4488	0.4484	0.3442	PRKD3
rs3821144	2	37362985	1.13	2.815	0.004883	A	G	825/3289/3148	0.4529	0.4488	0.4484	0.3442	PRKD3
rs4670682	2	37364023	1.13	2.815	0.004883	G	T	825/3289/3148	0.4529	0.4488	0.4484	0.3442	PRKD3
rs11124576	2	37372220	0.8306	-3.298	0.0009748	C	T	227/2351/4691	0.3234	0.3114	0.0009152	0.1887	PRKD3
rs4670684	2	37374574	1.132	2.816	0.004863	T	C	827/3205/3001	0.4557	0.4522	0.5269	0.3499	PRKD3
rs435923	2	40231136	1.179	2.897	0.003762	A	C	165/1793/5322	0.2463	0.2491	0.3465	0.149	SLC8A1
rs10221571	2	40231253	1.213	3.209	0.001331	G	C	111/1625/5322	0.2302	0.2274	0.3202	0.1346	SLC8A1
rs17025367	2	40248980	1.131	2.864	0.004179	G	A	975/3423/2742	0.4794	0.4694	0.07344	0.3801	SLC8A1
rs404005	2	40249582	1.145	3.09	0.002	T	C	890/3239/2813	0.4666	0.4616	0.3768	0.3659	SLC8A1
rs17025380	2	40250121	1.145	3.09	0.002	G	A	890/3239/2813	0.4666	0.4616	0.3768	0.3659	SLC8A1
rs2072529	2	40250669	1.145	3.09	0.002	A	G	890/3239/2813	0.4666	0.4616	0.3768	0.3659	SLC8A1
rs409522	2	40252760	1.134	2.932	0.003368	C	T	975/3429/2784	0.477	0.4683	0.1184	0.3781	SLC8A1
rs105287	2	40253683	1.13	2.852	0.004349	G	A	1007/3471/2795	0.4772	0.4698	0.1778	0.3807	SLC8A1
rs385341	2	40253993	1.138	2.975	0.002933	A	G	923/3283/2817	0.4675	0.4636	0.5034	0.3692	SLC8A1
rs433572	2	40258343	1.135	2.832	0.004629	T	C	783/3333/2823	0.4803	0.4568	1.83E-05	0.3566	SLC8A1

rs1012311	2	40328526	1.125	2.852	0.004351	G	C	1664/3578/2015	0.493	0.4988	0.323	0.4796	SLC8A1
rs10187179	2	45775647	0.8421	-2.91	0.003616	T	C	189/2007/5034	0.2776	0.2755	0.55	0.162	PRKCE
rs6720782	2	45775974	0.8421	-2.91	0.003616	T	C	189/2007/5034	0.2776	0.2755	0.55	0.1619	PRKCE
rs10490341	2	45777848	0.8468	-2.904	0.003686	G	A	220/2090/4970	0.2871	0.2871	1	0.1707	PRKCE
rs563601	2	45782134	0.8608	-2.919	0.003507	A	G	369/2623/4285	0.3605	0.3552	0.222	0.2275	PRKCE
rs637889	2	45785550	0.8643	-2.885	0.003913	G	A	405/2707/4168	0.3718	0.3664	0.2123	0.2379	PRKCE
rs11893174	2	49835492	0.8908	-2.813	0.004901	G	C	1839/3500/1907	0.483	0.5	0.004144	0.4906	
rs10490232	2	50312581	0.8364	-2.856	0.00429	G	C	151/1842/5276	0.2534	0.2515	0.5442	0.1436	NRXN1
rs17045546	2	54244074	0.8836	-2.935	0.003334	G	A	1554/3667/2057	0.5038	0.4976	0.2892	0.4603	ACYP2
rs2941581	2	54717365	0.8393	-2.866	0.00416	G	A	169/1865/5246	0.2562	0.2568	0.8196	0.148	SPTBN1
rs1370360	2	55416992	1.128	2.861	0.004226	G	A	1482/3618/2118	0.5012	0.4961	0.393	0.4612	CCDC88A
rs3791642	2	55426448	1.127	2.843	0.004473	T	C	1482/3614/2114	0.5012	0.4962	0.3929	0.4614	CCDC88A
rs6724338	2	55439129	1.128	2.861	0.004226	C	G	1482/3618/2118	0.5012	0.4961	0.393	0.4612	CCDC88A
rs12621289	2	55442446	1.128	2.861	0.004226	C	T	1482/3618/2118	0.5012	0.4961	0.393	0.4612	CCDC88A
rs3791650	2	55445822	1.128	2.861	0.004226	C	T	1482/3618/2118	0.5012	0.4961	0.393	0.4612	CCDC88A
rs9789365	2	55446278	1.128	2.861	0.004226	G	C	1482/3618/2118	0.5012	0.4961	0.393	0.4612	CCDC88A
rs3791652	2	55452081	1.128	2.861	0.004226	C	T	1482/3618/2118	0.5012	0.4961	0.393	0.4612	CCDC88A
rs10496042	2	55453314	1.128	2.861	0.004226	C	T	1482/3618/2118	0.5012	0.4961	0.393	0.4612	CCDC88A
rs7600456	2	55535852	0.8855	-2.882	0.003946	A	G	1661/3662/1893	0.5075	0.4995	0.1792	0.4781	
rs2008040	2	55540794	0.8855	-2.882	0.003946	C	G	1661/3662/1893	0.5075	0.4995	0.1792	0.478	
rs12612931	2	55557837	0.8854	-2.886	0.003901	A	C	1662/3663/1894	0.5074	0.4995	0.187	0.478	
rs12620109	2	55558710	0.8854	-2.886	0.003901	G	A	1662/3663/1894	0.5074	0.4995	0.187	0.4782	
rs957267	2	55562920	0.8854	-2.886	0.003901	A	G	1662/3663/1894	0.5074	0.4995	0.187	0.478	
rs13011640	2	55566606	0.8834	-2.925	0.003442	A	G	1662/3618/1850	0.5074	0.4997	0.1924	0.481	
rs12989168	2	55568990	0.8834	-2.925	0.003442	C	G	1662/3618/1850	0.5074	0.4997	0.1924	0.481	
rs2868435	2	55996502	1.366	2.918	0.003519	A	C	6/449/6825	0.06168	0.06132	0.8472	0.03364	EFEMP1
rs2903838	2	56003906	1.359	2.839	0.004527	G	A	6/442/6816	0.06085	0.06055	0.8463	0.03337	EFEMP1
rs3762515	2	56004368	1.377	2.958	0.003101	T	C	5/441/6825	0.06065	0.0601	0.5593	0.03301	EFEMP1
rs4672087	2	56208196	1.264	2.942	0.003262	T	C	23/886/6371	0.1217	0.1198	0.2034	0.06594	
rs10490394	2	56226740	1.278	3.121	0.001803	G	A	24/904/6349	0.1242	0.1223	0.2114	0.06736	
rs1961746	2	56228153	1.283	3.159	0.001581	A	T	24/891/6356	0.1225	0.1208	0.2443	0.06669	
rs7602685	2	57764659	0.8085	-2.908	0.003636	A	G	70/1376/5834	0.189	0.1866	0.2855	0.1015	
rs17049058	2	57765371	0.8067	-2.901	0.00372	G	A	68/1332/5872	0.1832	0.1815	0.4771	0.09823	
rs7419776	2	58706520	0.7575	-2.988	0.002811	G	A	22/902/6355	0.1239	0.1215	0.1006	0.06281	
rs17190597	2	58727889	0.7181	-2.851	0.004355	T	C	17/575/6586	0.08011	0.08124	0.2423	0.04076	FLJ30838
rs17190646	2	58760770	0.7533	-2.945	0.003228	G	C	24/840/6386	0.1159	0.115	0.609	0.05902	FLJ30838
rs17190653	2	58761165	0.7533	-2.945	0.003228	G	C	24/840/6386	0.1159	0.115	0.609	0.05902	FLJ30838
rs10168517	2	59310680	0.8449	-2.826	0.004706	G	C	176/1989/5115	0.2732	0.2699	0.3177	0.1569	
rs2244259	2	65564192	0.8815	-2.972	0.002957	G	A	1343/3612/2325	0.4962	0.4909	0.377	0.4278	FLJ16124
rs11900746	2	68529155	0.6519	-2.944	0.003243	G	T	8/400/6750	0.05588	0.05643	0.3969	0.02759	

rs17749719	2	76099282	1.41	2.842	0.004484	C	A	5/342/6933	0.04698	0.04718	0.6172	0.02564	
rs17339549	2	79931553	0.8316	-2.886	0.003906	A	G	136/1741/5403	0.2391	0.2383	0.8057	0.1346	CTNNA2
rs17339556	2	79931793	0.8357	-2.834	0.004602	C	A	139/1770/5371	0.2431	0.2417	0.6626	0.1371	CTNNA2
rs2861914	2	79934690	0.8299	-2.928	0.003412	G	T	139/1753/5388	0.2408	0.2401	0.8451	0.1357	CTNNA2
rs12104712	2	82498459	1.759	2.954	0.003134	T	G	1/113/7150	0.01556	0.01571	0.3654	0.00892	
rs1159790	2	84539400	1.229	3.02	0.002524	T	C	64/1179/6036	0.162	0.1634	0.4303	0.09221	SUCLG1
rs6717128	2	85534936	1.259	2.854	0.00432	G	C	34/805/5989	0.1179	0.1197	0.2238	0.06657	
rs6743301	2	85823031	0.8572	-3.073	0.002119	T	C	423/2788/4069	0.383	0.3746	0.06042	0.2453	
rs17026608	2	85826140	0.8527	-3.178	0.001482	G	C	429/2784/4067	0.3824	0.3751	0.1042	0.2458	
rs7593742	2	103211208	0.8595	-2.905	0.00367	A	G	369/2431/4262	0.3442	0.3481	0.356	0.2207	
rs2576742	2	105265254	1.164	3.137	0.001706	T	A	365/2467/4447	0.3389	0.3428	0.3384	0.2236	TGFBRAP1
rs3792048	2	105268105	0.8779	-2.992	0.002772	T	C	995/3328/2946	0.4578	0.464	0.266	0.3604	TGFBRAP1
rs920217	2	105271094	1.174	3.354	0.0007974	T	C	386/2411/4459	0.3323	0.3425	0.0123	0.2236	TGFBRAP1
rs2679895	2	105272912	1.168	3.196	0.001391	C	G	357/2465/4458	0.3386	0.3413	0.4923	0.2225	TGFBRAP1
rs10865027	2	109045029	0.8285	-2.842	0.004481	A	G	127/1610/5517	0.2219	0.2239	0.432	0.1256	
rs260667	2	109046627	0.8285	-2.842	0.004481	A	G	127/1610/5517	0.2219	0.2239	0.432	0.1254	
rs260666	2	109046657	0.8285	-2.842	0.004481	T	G	127/1610/5517	0.2219	0.2239	0.432	0.1255	
rs260654	2	109051831	0.8294	-2.841	0.004493	T	G	137/1593/5550	0.2188	0.2236	0.07447	0.1252	
rs260652	2	109053000	0.8285	-2.842	0.004481	G	T	127/1610/5517	0.2219	0.2239	0.432	0.1256	
rs260649	2	109055404	0.8298	-2.825	0.004735	G	C	127/1611/5541	0.2213	0.2234	0.4311	0.1252	
rs7575835	2	110337635	1.125	2.847	0.004419	A	G	1303/3432/2545	0.4714	0.4854	0.0138	0.4187	
rs11123222	2	114517132	0.8676	-3.247	0.001165	C	T	1012/3420/2847	0.4698	0.4682	0.783	0.3682	
rs465010	2	114518173	0.8797	-2.836	0.004562	G	C	781/3278/3219	0.4504	0.4439	0.2147	0.3275	
rs156719	2	114526503	0.8801	-2.83	0.00465	T	C	782/3280/3216	0.4507	0.4441	0.2147	0.3278	
rs4848361	2	114527177	0.875	-3.018	0.002546	A	C	1008/3279/2744	0.4664	0.4695	0.5763	0.3712	
rs10200251	2	114541508	0.8657	-3.087	0.002025	C	T	750/3128/2972	0.4566	0.4474	0.08892	0.332	
rs7561168	2	114542608	0.8732	-3.007	0.002639	A	G	859/3293/2970	0.4624	0.4561	0.253	0.3461	
rs923229	2	114547273	0.872	-3.069	0.002144	C	A	902/3290/2973	0.4592	0.4582	0.8771	0.3496	
rs3853140	2	114551286	0.8732	-3.007	0.002639	G	T	859/3293/2970	0.4624	0.4561	0.253	0.3461	
rs12615208	2	114554292	0.8657	-3.087	0.002025	C	T	750/3128/2972	0.4566	0.4474	0.08892	0.3321	
rs3912544	2	114556809	0.8674	-3.21	0.001328	G	T	926/3329/3014	0.458	0.4587	0.8983	0.3504	
rs2900893	2	114561377	0.8701	-3.098	0.001949	C	T	868/3331/3014	0.4618	0.4557	0.2666	0.3455	
rs4089089	2	114562752	0.8674	-3.21	0.001328	G	A	926/3329/3014	0.458	0.4587	0.8983	0.3504	
rs6712445	2	114637366	0.834	-3.056	0.002243	C	T	224/1869/4956	0.2651	0.2747	0.004176	0.1605	
rs1118558	2	114644502	0.834	-3.056	0.002243	C	T	224/1869/4956	0.2651	0.2747	0.004176	0.1605	
rs3132076	2	114647751	0.834	-3.054	0.002255	C	A	222/1871/4955	0.2655	0.2745	0.006238	0.1604	
rs3132078	2	114650688	0.834	-3.056	0.002243	C	A	224/1869/4956	0.2651	0.2747	0.004176	0.1605	
rs17759541	2	114653899	0.834	-3.056	0.002243	G	A	224/1869/4956	0.2651	0.2747	0.004176	0.1605	
rs7570888	2	114654074	0.834	-3.056	0.002243	T	C	224/1869/4956	0.2651	0.2747	0.004176	0.1605	
rs17759715	2	114656432	0.834	-3.056	0.002243	T	C	224/1869/4956	0.2651	0.2747	0.004176	0.1605	

rs17048216	2	114656451	0.834	-3.056	0.002243	T	C	224/1869/4956	0.2651	0.2747	0.004176	0.1605	
rs11900957	2	114658299	0.834	-3.056	0.002243	G	A	224/1869/4956	0.2651	0.2747	0.004176	0.1605	
rs1430671	2	114662559	0.834	-3.054	0.002255	A	G	222/1871/4955	0.2655	0.2745	0.006238	0.1604	
rs17709089	2	114665504	0.8511	-2.852	0.004338	A	T	245/2136/4899	0.2934	0.2957	0.5258	0.1765	
rs34616215	2	114665564	0.8515	-2.843	0.004475	A	G	245/2135/4900	0.2933	0.2956	0.5004	0.1764	
rs2421091	2	125053510	0.8892	-2.813	0.004901	C	T	1514/3498/2268	0.4805	0.4946	0.01467	0.4445	CNTNAP5
rs6430501	2	134660614	1.136	2.834	0.004604	T	C	619/2961/3695	0.407	0.4106	0.4579	0.2934	
rs6743206	2	135410007	0.6902	-2.954	0.003135	G	C	10/510/6760	0.07005	0.07015	0.8666	0.035	CCNT2
rs10180515	2	136638173	0.8669	-3.346	0.0008206	A	T	1267/3568/2445	0.4901	0.4869	0.5799	0.4144	
rs10199153	2	137213276	0.8695	-3.09	0.002003	G	C	792/3105/3383	0.4265	0.4367	0.04705	0.3172	
rs7564005	2	137229168	0.8742	-2.961	0.003063	G	T	765/3103/3412	0.4262	0.4339	0.1305	0.3138	
rs1427591	2	137230888	0.8675	-2.909	0.003631	T	G	488/2796/3996	0.3841	0.3839	1	0.2552	
rs1427300	2	144998915	1.346	2.913	0.003574	G	C	18/451/6650	0.06335	0.06607	0.001789	0.03616	
rs13027627	2	145002135	1.349	2.941	0.003277	T	C	18/450/6650	0.06322	0.06595	0.001726	0.03611	
rs12465209	2	147402478	0.869	-2.816	0.004858	A	C	438/2678/4164	0.3679	0.369	0.7994	0.241	
rs10931753	2	154277003	1.211	2.886	0.003907	C	G	65/1283/5932	0.1762	0.1753	0.6882	0.09993	
rs16844494	2	160478671	1.158	2.866	0.004158	A	G	264/2325/4691	0.3194	0.3151	0.2644	0.1992	
rs2271379	2	160496729	1.163	3.015	0.002567	C	A	307/2364/4609	0.3247	0.3254	0.8571	0.208	
rs3736448	2	160512362	1.202	2.978	0.0029	G	A	83/1656/5198	0.2387	0.2282	7.58E-05	0.1338	PLA2R1
rs3828322	2	160515843	1.17	3.051	0.002282	T	A	258/2220/4800	0.305	0.3053	0.9388	0.1914	PLA2R1
rs2715917	2	160539744	1.166	2.976	0.002916	C	G	256/2215/4801	0.3046	0.3047	0.9693	0.1909	PLA2R1
rs1567538	2	160550607	1.16	2.85	0.004376	C	T	240/2171/4866	0.2983	0.2979	0.9373	0.1854	PLA2R1
rs270958	2	161423884	0.8737	-2.864	0.004187	A	G	620/3013/3540	0.42	0.4171	0.5713	0.2921	
rs17643505	2	168303212	1.192	2.985	0.002832	A	G	125/1700/5455	0.2335	0.232	0.6134	0.1372	
rs2044681	2	168304029	1.2	3.126	0.001773	C	T	125/1727/5428	0.2372	0.2347	0.3957	0.1393	
rs10196226	2	168363838	0.8819	-2.863	0.004196	A	G	1006/3383/2891	0.4647	0.4665	0.744	0.3657	
rs7576736	2	168365653	0.8815	-2.846	0.004425	G	A	968/3365/2890	0.4659	0.4646	0.8394	0.3622	
rs4667975	2	168366721	0.8815	-2.846	0.004425	T	C	968/3365/2890	0.4659	0.4646	0.8394	0.3623	
rs908595	2	168707447	0.8675	-3.293	0.0009908	T	C	1219/3501/2456	0.4879	0.4851	0.6439	0.4093	STK39
rs2063958	2	168718905	0.8588	-2.908	0.003639	G	A	340/2498/4442	0.3431	0.3413	0.6553	0.215	STK39
rs3769391	2	168721934	0.8508	-3.077	0.002094	T	G	342/2464/4456	0.3393	0.3395	0.9449	0.2133	STK39
rs3754781	2	168723722	0.8548	-2.981	0.002876	A	G	342/2449/4419	0.3397	0.3401	0.9173	0.214	STK39
rs3754777	2	168724160	0.8508	-3.077	0.002094	T	C	342/2464/4456	0.3393	0.3395	0.9449	0.2133	STK39
rs3820861	2	168724696	0.8508	-3.077	0.002094	T	A	342/2464/4456	0.3393	0.3395	0.9449	0.2133	STK39
rs3820860	2	168724706	0.8508	-3.077	0.002094	T	C	342/2464/4456	0.3393	0.3395	0.9449	0.2133	STK39
rs3820859	2	168725215	0.8503	-3.088	0.002015	C	G	342/2463/4457	0.3392	0.3395	0.9449	0.2132	STK39
rs16854951	2	168725615	0.8503	-3.088	0.002015	T	A	342/2463/4457	0.3392	0.3395	0.9449	0.2133	STK39
rs13023246	2	168731688	0.8503	-3.088	0.002015	A	G	342/2463/4457	0.3392	0.3395	0.9449	0.2132	STK39
rs13022764	2	168731722	0.8503	-3.088	0.002015	G	A	342/2463/4457	0.3392	0.3395	0.9449	0.2133	STK39
rs13023468	2	168731795	0.8503	-3.088	0.002015	A	G	342/2463/4457	0.3392	0.3395	0.9449	0.2132	STK39

rs12616582	2	168735075	0.8503	-3.088	0.002015	A	T	342/2463/4457	0.3392	0.3395	0.9449	0.2133	STK39
rs10497337	2	168735395	0.8528	-3.043	0.002343	A	G	346/2472/4461	0.3396	0.3402	0.8904	0.214	STK39
rs16855027	2	168735530	0.8428	-3.265	0.001095	T	A	346/2518/4416	0.3459	0.3437	0.6091	0.2166	STK39
rs4668044	2	168750016	0.8822	-2.909	0.003628	G	A	1294/3440/2269	0.4912	0.4903	0.8838	0.426	STK39
rs13385577	2	168755029	0.8785	-3.062	0.002198	A	T	1332/3556/2390	0.4886	0.4894	0.8857	0.423	STK39
rs10202854	2	168755530	0.8784	-3.063	0.002192	T	C	1328/3556/2396	0.4885	0.4892	0.9047	0.4223	STK39
rs10930311	2	168766011	0.8811	-2.931	0.003374	T	C	1309/3396/2221	0.4903	0.4913	0.8641	0.4297	STK39
rs932167	2	168773714	0.8823	-2.901	0.003721	C	A	1309/3372/2219	0.4887	0.4913	0.6592	0.4297	STK39
rs13034253	2	168775742	0.8451	-3.181	0.001467	C	A	340/2445/4298	0.3452	0.3439	0.7822	0.2169	STK39
rs16855122	2	168776192	0.8451	-3.181	0.001467	T	C	340/2445/4298	0.3452	0.3439	0.7822	0.2169	STK39
rs4438452	2	168779594	0.8597	-2.971	0.002968	T	C	399/2569/4312	0.3529	0.3555	0.5311	0.2278	STK39
rs10176669	2	168793105	0.8593	-3.55	0.0003845	T	C	1263/3539/2478	0.4861	0.4861	1	0.4116	STK39
rs1399960	2	172192979	1.124	2.807	0.004997	G	A	1336/3534/2410	0.4854	0.4891	0.5177	0.4307	
rs13007575	2	174386625	0.8664	-2.864	0.004187	C	T	500/2788/3457	0.4133	0.4039	0.05752	0.2766	
rs12463717	2	174387754	0.8664	-2.864	0.004187	A	C	500/2788/3457	0.4133	0.4039	0.05752	0.2766	
rs867458	2	174406951	0.8637	-3.077	0.002093	A	G	592/2992/3693	0.4112	0.4092	0.7096	0.2825	
rs4072893	2	174407933	0.8625	-3.107	0.001892	T	C	595/2992/3693	0.411	0.4095	0.7747	0.2827	
rs16862127	2	174409243	0.8637	-3.077	0.002093	C	G	592/2992/3693	0.4112	0.4092	0.7096	0.2826	
rs12996017	2	192269452	1.247	3.085	0.002037	G	A	53/1110/6099	0.1529	0.1534	0.7594	0.0857	
rs6722563	2	192278944	1.246	3.079	0.002074	G	T	54/1116/6103	0.1534	0.1541	0.7034	0.08594	
rs10931515	2	192283356	1.288	4.243	2.20E-05	G	C	115/1589/5576	0.2183	0.2186	0.8723	0.129	
rs10202549	2	192293260	1.334	2.879	0.003988	C	T	7/537/6707	0.07406	0.0731	0.3343	0.04017	
rs7596512	2	192294847	1.239	3.63	0.0002839	A	G	132/1634/5514	0.2245	0.2267	0.3798	0.1341	
rs6731095	2	192297687	1.246	3.131	0.001743	G	A	55/1130/6095	0.1552	0.1558	0.7072	0.08801	
rs17513156	2	194994653	1.566	2.875	0.004041	T	C	3/179/7076	0.02466	0.02516	0.1123	0.01398	
rs10166118	2	196904972	1.533	2.896	0.003782	C	T	2/216/7052	0.02971	0.0298	0.6823	0.01626	HECW2
rs2168369	2	196905337	1.533	2.896	0.003782	A	G	2/216/7052	0.02971	0.0298	0.6823	0.01626	HECW2
rs1014255	2	196908436	1.533	2.896	0.003782	G	A	2/216/7052	0.02971	0.0298	0.6823	0.01626	HECW2
rs7593977	2	196912979	1.533	2.895	0.003788	A	G	2/216/7048	0.02973	0.02982	0.6825	0.01627	HECW2
rs12622284	2	199408976	0.8705	-3.145	0.001663	A	C	1059/3520/2605	0.49	0.4768	0.02004	0.387	
rs13017811	2	200172805	1.483	2.863	0.004201	A	G	2/245/6991	0.03385	0.03381	1	0.01874	
rs1961468	2	200196130	1.483	2.863	0.004201	A	T	2/245/6991	0.03385	0.03381	1	0.01874	
rs13404330	2	200302553	0.8327	-3.017	0.002549	C	G	170/1932/5082	0.2689	0.2662	0.425	0.1538	
rs4673448	2	200305526	0.8327	-3.017	0.002549	A	G	170/1932/5082	0.2689	0.2662	0.425	0.1538	
rs4673450	2	200305926	0.8327	-3.017	0.002549	C	A	170/1932/5082	0.2689	0.2662	0.425	0.1537	
rs1996947	2	200306383	0.8327	-3.017	0.002549	G	C	170/1932/5082	0.2689	0.2662	0.425	0.1538	
rs1996946	2	200310073	0.8466	-2.821	0.004785	A	G	187/2010/5082	0.2761	0.2739	0.5208	0.1595	
rs12328774	2	202591142	0.7885	-2.834	0.004599	G	T	37/1074/6169	0.1475	0.1453	0.197	0.07613	LOC729487
rs933985	2	204624309	1.245	3.009	0.002617	G	A	52/996/6190	0.1376	0.1404	0.09351	0.07854	
rs4675453	2	204983435	1.133	2.931	0.003383	C	T	1518/3741/1973	0.5173	0.498	0.001032	0.4737	

rs16841256	2	205008062	1.129	2.833	0.004611	T	G	1199/3353/2450	0.4789	0.484	0.3741	0.4152	
rs2306432	2	208398705	0.8649	-3.279	0.001042	T	G	931/3374/2975	0.4635	0.4606	0.6108	0.354	
rs11893393	2	208633155	1.151	3.125	0.001777	A	G	1307/3553/1782	0.5349	0.4974	9.34E-10	0.4698	
rs1250246	2	216007526	0.7186	-3.082	0.002059	C	A	22/679/6361	0.09615	0.09714	0.3896	0.04912	FN1
rs2577290	2	216007795	0.7186	-3.082	0.002059	G	A	22/679/6361	0.09615	0.09714	0.3896	0.04912	FN1
rs1250247	2	216007874	0.7186	-3.082	0.002059	C	G	22/679/6361	0.09615	0.09714	0.3896	0.04912	FN1
rs6714280	2	217886714	1.372	2.836	0.004566	C	G	4/417/6859	0.05728	0.05668	0.5313	0.03079	
rs4672884	2	218890725	1.146	3.107	0.001893	A	G	1103/3258/2343	0.486	0.4829	0.613	0.4121	PNKD
rs4552182	2	218893116	1.139	3.004	0.002661	G	T	1103/3258/2410	0.4812	0.4814	0.9799	0.408	PNKD
rs4672886	2	218893789	1.139	3.004	0.002661	G	A	1103/3258/2410	0.4812	0.4814	0.9799	0.408	PNKD
rs3731861	2	218899500	1.139	3.004	0.002661	C	T	1103/3258/2410	0.4812	0.4814	0.9799	0.408	PNKD
rs10932774	2	218899813	1.137	3.056	0.00224	A	G	1099/3394/2780	0.4667	0.4733	0.2344	0.389	PNKD
rs897877	2	218900999	1.135	2.937	0.003311	A	G	1099/3293/2445	0.4816	0.4806	0.88	0.4059	PNKD
rs3770155	2	222046054	1.159	3.28	0.001039	C	T	1160/3551/2068	0.5238	0.491	3.96E-08	0.439	EPHA4
rs3770150	2	222047655	1.136	3.065	0.002179	T	C	1569/3636/2074	0.4995	0.4976	0.7594	0.4708	EPHA4
rs13009679	2	222048281	1.128	2.904	0.00369	A	G	1604/3588/2084	0.4931	0.4978	0.4233	0.4723	EPHA4
rs12463452	2	222048483	1.135	3.036	0.002401	T	C	1565/3644/2068	0.5008	0.4976	0.6043	0.4709	EPHA4
rs7563304	2	222050284	1.14	3.087	0.002019	A	T	1485/3550/2065	0.5	0.4967	0.5826	0.4648	EPHA4
rs7566787	2	222051049	1.132	2.958	0.003092	A	T	1519/3515/2081	0.494	0.4969	0.6332	0.466	EPHA4
rs3770149	2	222051136	1.132	2.958	0.003092	C	T	1519/3515/2081	0.494	0.4969	0.6332	0.466	EPHA4
rs1864462	2	222052067	1.132	2.958	0.003092	G	C	1519/3515/2081	0.494	0.4969	0.6332	0.4659	EPHA4
rs3770146	2	222052440	1.14	3.087	0.002019	G	A	1485/3550/2065	0.5	0.4967	0.5826	0.4648	EPHA4
rs3770145	2	222053391	1.14	3.087	0.002019	T	C	1485/3550/2065	0.5	0.4967	0.5826	0.4648	EPHA4
rs1864461	2	222055859	1.132	2.958	0.003092	A	G	1519/3515/2081	0.494	0.4969	0.6332	0.466	EPHA4
rs11692482	2	222060649	1.148	3.192	0.001412	C	T	1411/3425/1991	0.5017	0.4964	0.3934	0.4635	EPHA4
rs10498113	2	222060873	1.143	3.18	0.001472	C	T	1489/3655/2124	0.5029	0.4962	0.2565	0.4622	EPHA4
rs13026659	2	222061258	1.148	3.193	0.001407	C	T	1418/3427/1997	0.5009	0.4964	0.4651	0.4636	EPHA4
rs1367241	2	222061657	1.148	3.193	0.001407	G	A	1418/3427/1997	0.5009	0.4964	0.4651	0.4636	EPHA4
rs7573758	2	222065570	1.137	2.942	0.003257	G	T	1352/3427/1997	0.5058	0.4955	0.0907	0.4579	EPHA4
rs9288570	2	222087238	1.136	2.914	0.003565	A	G	801/3302/3177	0.4536	0.4467	0.1986	0.342	EPHA4
rs412120	2	225556634	0.8595	-2.941	0.003268	A	T	390/2580/4310	0.3544	0.355	0.8689	0.2269	
rs4305263	2	228295368	0.8711	-2.822	0.004771	A	T	484/2734/4057	0.3758	0.3794	0.4217	0.2495	
rs1527667	2	229423197	0.4403	-3.181	0.001467	T	C	0/187/7077	0.02574	0.02541	0.6346	0.01161	
rs6705605	2	231077141	1.278	3.078	0.002081	T	G	22/840/6418	0.1154	0.1141	0.3559	0.06314	SP100
rs17275036	2	231114314	1.294	3.212	0.001317	A	G	22/817/6441	0.1122	0.1113	0.5278	0.06163	
rs10183022	2	237146708	0.8736	-3.038	0.002383	A	G	938/3398/2943	0.4668	0.4621	0.3885	0.3581	CXCR7
rs4633874	2	237340720	1.141	2.894	0.003808	G	A	558/2951/3771	0.4054	0.4026	0.5802	0.2833	
rs7603207	2	239512773	0.8806	-2.82	0.004795	A	C	833/3290/3157	0.4519	0.449	0.6017	0.336	FLJ43879
rs3755325	2	241822589	0.8229	-3.153	0.001618	A	G	175/1784/5229	0.2482	0.2528	0.1236	0.1456	HDLBP
rs9876432	3	4736185	0.7917	-2.818	0.00484	A	G	54/1050/5910	0.1497	0.1515	0.3434	0.08062	ITPR1

rs11714280	3	8953972	1.708	3.22	0.001281	C	T	1/158/7110	0.02174	0.02177	0.5872	0.01245	RAD18
rs1145158	3	9220529	1.132	2.81	0.004947	T	C	722/3047/3507	0.4188	0.4267	0.1111	0.3136	SRGAP3
rs2648559	3	9329353	1.288	2.861	0.004223	G	A	16/686/6578	0.09423	0.09376	0.8022	0.05116	
rs713024	3	10156240	1.18	2.967	0.003005	A	G	177/1771/5319	0.2437	0.2497	0.0433	0.1496	
rs28113	3	10375643	0.2109	-3.026	0.002482	G	A	0/94/7144	0.01299	0.0129	1	0.0058	ATP2B2
rs1710873	3	11007829	0.8861	-2.836	0.004572	G	C	1306/3548/2419	0.4878	0.4883	0.9426	0.4184	SLC6A1
rs1728818	3	11007992	0.8854	-2.84	0.004517	G	C	1293/3542/2390	0.4902	0.4885	0.7726	0.4194	
rs2697148	3	11011580	0.8863	-2.83	0.004654	C	A	1298/3562/2420	0.4893	0.4881	0.8477	0.4183	SLC6A1
rs2601126	3	11011624	0.8854	-2.84	0.004517	T	C	1293/3542/2390	0.4902	0.4885	0.7726	0.4194	SLC6A1
rs1710898	3	11012760	0.8854	-2.84	0.004517	A	C	1293/3542/2390	0.4902	0.4885	0.7726	0.4194	SLC6A1
rs9832078	3	12777109	0.8276	-2.817	0.004846	G	A	111/1601/5297	0.2284	0.2263	0.4598	0.127	
rs9863496	3	18773852	0.8648	-2.827	0.004704	C	T	390/2745/3900	0.3902	0.3755	0.001069	0.2477	
rs9825093	3	18775247	0.865	-2.822	0.004765	A	C	390/2744/3900	0.3901	0.3755	0.00107	0.2476	
rs4858270	3	20831543	0.7901	-3.365	0.0007663	A	G	114/1456/5709	0.2	0.2046	0.05864	0.1119	
rs1499004	3	20855876	1.263	3.108	0.001881	C	G	33/976/6271	0.1341	0.1329	0.5363	0.07407	
rs6807580	3	20887126	0.7042	-2.808	0.004992	A	G	12/527/6741	0.07239	0.07282	0.6268	0.03633	
rs4549291	3	20894924	0.7077	-2.813	0.004904	T	G	12/542/6726	0.07445	0.07472	0.7523	0.03734	
rs2132145	3	23277653	1.14	2.948	0.003194	G	A	706/3181/3393	0.437	0.4319	0.3286	0.3199	UBE2E2
rs1863616	3	25957135	1.386	2.809	0.004972	A	G	2/384/6882	0.05283	0.05196	0.2506	0.02866	
rs9874450	3	25957891	1.386	2.809	0.004972	A	G	2/384/6882	0.05283	0.05196	0.2506	0.02866	
rs2114456	3	25959888	1.386	2.809	0.004972	C	T	2/384/6882	0.05283	0.05196	0.2506	0.02866	
rs9310860	3	28038792	1.594	3.386	0.0007098	T	C	4/228/6909	0.03193	0.0325	0.1294	0.01843	
rs17029872	3	30745291	0.8589	-2.948	0.003193	A	G	363/2655/4257	0.3649	0.3567	0.05247	0.2276	GADL1
rs1875688	3	30772129	0.8776	-2.866	0.004163	A	G	746/3196/3331	0.4394	0.4368	0.629	0.3171	GADL1
rs839588	3	31071883	0.7284	-3.031	0.002439	A	G	12/743/6525	0.1021	0.09981	0.05908	0.05122	
rs1353327	3	31477964	0.8562	-2.913	0.003578	T	C	319/2253/4707	0.3095	0.3183	0.02029	0.1952	
rs9828566	3	31500384	0.8464	-3.401	0.0006712	T	G	497/2684/4094	0.3689	0.3778	0.04699	0.2482	
rs9863982	3	31509158	0.8466	-3.586	0.0003363	C	T	685/3018/3576	0.4146	0.4211	0.1908	0.2964	
rs9854271	3	31510764	0.8581	-2.954	0.003134	A	G	367/2395/4518	0.329	0.3374	0.03407	0.2114	
rs11705838	3	33958201	0.8532	-3.581	0.0003423	G	A	940/3483/2856	0.4785	0.4654	0.01672	0.3624	
rs1112837	3	33960372	0.8807	-3.036	0.0024	C	T	1834/3681/1765	0.5056	0.5	0.3366	0.4995	
rs11714393	3	34225115	0.8199	-2.972	0.002955	C	A	118/1610/5550	0.2212	0.2215	0.9157	0.1237	
rs12638795	3	35089225	1.228	3.01	0.002615	A	G	55/1215/6005	0.167	0.1655	0.4794	0.09434	
rs2555233	3	37586339	0.851	-3.885	0.0001022	C	T	1871/3610/1798	0.4959	0.4999	0.4966	0.4988	ITGA9
rs12491609	3	37614425	1.155	2.815	0.004877	G	A	261/2478/4418	0.3462	0.3313	0.0001326	0.2131	ITGA9
rs2685104	3	37782039	1.141	3.095	0.001968	T	G	1106/3502/2672	0.481	0.4769	0.461	0.3973	ITGA9
rs3845948	3	37798704	0.8855	-2.866	0.004158	C	T	1384/3648/2248	0.5011	0.493	0.1608	0.4365	ITGA9
rs7639220	3	40260149	1.144	2.956	0.003113	G	T	584/2846/3638	0.4027	0.4066	0.4128	0.2893	YRIP FLJ3306
rs7639223	3	40260164	1.144	2.956	0.003113	G	A	584/2846/3638	0.4027	0.4066	0.4128	0.2893	YRIP FLJ3306
rs4618168	3	40261021	1.144	2.956	0.003113	T	C	584/2846/3638	0.4027	0.4066	0.4128	0.2892	YRIP FLJ3306

rs9831041	3	40267499	1.144	2.956	0.003113	C	G	584/2846/3638	0.4027	0.4066	0.4128	0.2893	YRIP FLJ3306
rs1356875	3	46643693	1.129	2.813	0.004903	C	T	900/3407/2973	0.468	0.4595	0.1197	0.3635	
rs13064588	3	54607587	0.7911	-3.039	0.002371	A	T	73/1216/5991	0.167	0.1696	0.2129	0.09052	CACNA2D3
rs4955928	3	55090549	0.8664	-2.835	0.004583	A	G	386/2592/4302	0.356	0.3553	0.895	0.2278	
rs358001	3	55093495	0.8664	-2.835	0.004583	T	C	386/2592/4302	0.356	0.3553	0.895	0.2278	
rs17257847	3	55094184	0.8664	-2.835	0.004583	A	G	386/2592/4302	0.356	0.3553	0.895	0.228	
rs358018	3	55105358	1.381	2.826	0.00472	A	C	7/384/6789	0.05348	0.0539	0.5055	0.02904	
rs9830116	3	55405696	1.148	2.98	0.002878	T	C	570/2746/3455	0.4056	0.4092	0.4579	0.2923	
rs1402292	3	55406449	1.148	3.003	0.002677	G	T	557/2832/3656	0.402	0.4032	0.7903	0.2853	
rs1658343	3	57984058	1.339	2.892	0.003833	T	C	5/492/6632	0.06901	0.06794	0.2219	0.03792	FLNB
rs1718459	3	57985340	1.339	2.892	0.003833	C	T	5/492/6632	0.06901	0.06794	0.2219	0.03792	FLNB
rs1658351	3	57988613	1.339	2.892	0.003833	C	T	5/492/6632	0.06901	0.06794	0.2219	0.03792	FLNB
rs1658397	3	57993147	1.339	2.892	0.003833	G	C	5/492/6632	0.06901	0.06794	0.2219	0.03793	FLNB
rs839237	3	57993996	1.339	2.892	0.003833	C	T	5/492/6632	0.06901	0.06794	0.2219	0.03792	FLNB
rs839235	3	57994487	1.339	2.892	0.003833	T	C	5/492/6632	0.06901	0.06794	0.2219	0.03792	FLNB
rs1718483	3	57995035	1.339	2.892	0.003833	C	T	5/492/6632	0.06901	0.06794	0.2219	0.03792	FLNB
rs839233	3	57995640	1.339	2.892	0.003833	C	T	5/492/6632	0.06901	0.06794	0.2219	0.03792	FLNB
rs1823242	3	60482851	0.873	-2.985	0.002837	G	T	753/3102/3408	0.4271	0.4332	0.2333	0.3124	FHIT
rs504739	3	62703563	0.8062	-3.043	0.002341	T	C	96/1439/5745	0.1977	0.1989	0.5958	0.1104	CADPS
rs526005	3	62706363	0.8113	-2.966	0.003021	G	A	89/1467/5722	0.2016	0.2005	0.6825	0.1106	CADPS
rs17069000	3	63500328	0.7198	-3.203	0.00136	A	C	29/732/6519	0.1005	0.1026	0.08632	0.05223	SYNPR
rs17048062	3	68985141	1.469	2.915	0.003561	C	T	5/269/6989	0.03704	0.03768	0.1946	0.02076	FAM19A4
rs17048087	3	68989002	1.453	2.885	0.003917	T	A	5/283/6992	0.03887	0.03944	0.2207	0.02161	FAM19A4
rs4855535	3	69017124	1.126	2.849	0.004392	G	T	1497/3622/2160	0.4976	0.4959	0.7767	0.4589	FAM19A4
rs4855347	3	69069904	0.8726	-2.964	0.003037	C	T	893/3366/2574	0.4926	0.4697	5.86E-05	0.3731	
rs17048187	3	69075830	0.8726	-2.964	0.003037	A	G	893/3366/2574	0.4926	0.4697	5.86E-05	0.3731	
rs6787753	3	69078627	0.8726	-2.964	0.003037	A	C	893/3366/2574	0.4926	0.4697	5.86E-05	0.3731	
rs6803374	3	69079328	0.8726	-2.964	0.003037	G	A	893/3366/2574	0.4926	0.4697	5.86E-05	0.3731	
rs2575762	3	87210145	1.151	2.97	0.002976	A	G	461/2698/3904	0.382	0.3812	0.876	0.2609	
rs12636460	3	87234560	1.151	2.97	0.002976	G	C	461/2698/3904	0.382	0.3812	0.876	0.261	
rs9310048	3	87235371	1.151	2.97	0.002976	C	T	461/2698/3904	0.382	0.3812	0.876	0.261	
rs16840752	3	100406740	0.7691	-3.069	0.002147	T	C	44/1025/6209	0.1408	0.1412	0.8032	0.07352	
rs1589346	3	100412110	0.7534	-3.106	0.001894	T	C	34/925/6272	0.1279	0.1279	1	0.06591	
rs6803675	3	100417202	0.7675	-3.07	0.002143	G	A	42/1015/6223	0.1394	0.1396	0.933	0.07255	
rs4856089	3	105090228	1.129	2.866	0.004163	A	G	1662/3656/1907	0.506	0.4994	0.2683	0.4876	
rs1921336	3	105095547	1.128	2.883	0.003939	T	C	1709/3663/1907	0.5032	0.4996	0.5575	0.491	
rs4856090	3	105101013	1.128	2.85	0.004371	T	C	1663/3657/1906	0.5061	0.4994	0.2683	0.4878	
rs6799166	3	105105383	1.162	3.513	0.0004425	A	T	1644/3533/1861	0.502	0.4995	0.6851	0.4905	
rs9881358	3	105113157	1.159	3.505	0.0004573	A	G	1686/3668/1896	0.5059	0.4996	0.2901	0.4912	
rs9833794	3	105119604	1.157	3.428	0.0006074	C	T	1644/3467/1846	0.4983	0.4996	0.8477	0.4913	

rs4856091	3	105123848	1.161	3.522	0.0004281	A	C	1644/3656/1898	0.5079	0.4994	0.15	0.4882		
rs6797420	3	105126210	0.8658	-3.409	0.0006513	A	G	1469/3635/2176	0.4993	0.4953	0.4927	0.446		
rs6797644	3	105126435	0.8657	-3.41	0.000649	A	G	1468/3636/2176	0.4995	0.4953	0.4778	0.4459		
rs9288768	3	105130355	1.137	3.09	0.002004	A	G	1713/3583/1984	0.4922	0.4993	0.2223	0.4866		
rs9858971	3	105138574	1.137	3.09	0.002004	A	G	1713/3583/1984	0.4922	0.4993	0.2223	0.4866		
rs9288772	3	105148699	1.137	3.09	0.002004	A	G	1713/3583/1984	0.4922	0.4993	0.2223	0.4866		
rs6779354	3	105157710	1.136	3.064	0.002185	T	C	1575/3582/2115	0.4926	0.4972	0.4227	0.4677		
rs9834400	3	105159194	1.129	2.888	0.003883	A	C	1597/3612/2018	0.4998	0.4983	0.8134	0.4752		
rs6786263	3	105159688	1.131	2.936	0.003329	G	C	1590/3612/2018	0.5003	0.4982	0.7409	0.4748		
rs7634768	3	105160823	0.8732	-3.228	0.001248	C	T	1608/3637/2035	0.4996	0.4983	0.8324	0.4659		
rs6437479	3	105161421	1.134	2.995	0.002747	G	A	1722/3602/1897	0.4988	0.4997	0.8876	0.4926		
rs6782375	3	105164123	1.129	2.888	0.003883	T	G	1597/3612/2018	0.4998	0.4983	0.8134	0.4752		
rs9810308	3	105164656	1.137	3.06	0.002216	C	T	1719/3658/1903	0.5025	0.4997	0.6391	0.4922		
rs9853755	3	105167776	0.8711	-3.285	0.001019	G	T	1600/3633/2047	0.499	0.4981	0.8877	0.4643		
rs1900684	3	105171999	1.131	2.93	0.003386	G	A	1593/3657/2022	0.5029	0.4983	0.4374	0.4749		
rs7630679	3	105172123	1.131	2.93	0.003386	T	A	1593/3657/2022	0.5029	0.4983	0.4374	0.4749		
rs736328	3	109941108	0.8847	-2.877	0.00401	T	C	1237/3457/2517	0.4794	0.4842	0.3948	0.4069		
rs2715694	3	110023833	0.8823	-2.944	0.003236	T	C	1238/3481/2560	0.4782	0.4835	0.3569	0.4044		TRAT1
rs4855735	3	110584473	0.88	-2.874	0.004057	C	T	1027/3153/2589	0.4658	0.4734	0.1904	0.3796		
rs6806987	3	110591487	0.8321	-3.31	0.0009318	T	A	303/2214/4237	0.3278	0.3304	0.5312	0.2035		
rs769486	3	110596160	0.8416	-3.178	0.001483	C	G	323/2333/4182	0.3412	0.3408	0.9434	0.2128		
rs769505	3	110598647	0.8416	-3.178	0.001483	G	A	323/2333/4182	0.3412	0.3408	0.9434	0.2128		
rs769501	3	110601443	0.8417	-3.175	0.001497	G	C	324/2330/4181	0.3409	0.3408	1	0.2128		
rs769498	3	110603087	0.8448	-3.161	0.001572	C	T	326/2389/4516	0.3304	0.3321	0.6455	0.2056		
rs769495	3	110603872	0.8444	-3.171	0.001519	G	A	325/2392/4520	0.3305	0.332	0.697	0.2054		
rs808802	3	110611382	0.8392	-3.318	0.000907	C	T	344/2413/4519	0.3316	0.3354	0.3452	0.2082		
rs6778096	3	110625114	0.8798	-3.038	0.002383	G	A	1514/3601/2122	0.4976	0.4965	0.8684	0.4525		
rs1905351	3	110644456	0.8683	-3.257	0.001128	C	T	1365/3443/2122	0.4968	0.494	0.6442	0.4395		FLJ25363
rs1462309	3	112009941	0.8628	-3.454	0.000552	T	G	1274/3551/2431	0.4894	0.4873	0.7359	0.4142		
rs2895378	3	112215362	0.8684	-3.322	0.0008932	G	C	1307/3563/2410	0.4894	0.4885	0.8856	0.4181		
rs1499901	3	114387968	1.136	2.829	0.004677	C	T	609/2893/3778	0.3974	0.4053	0.09925	0.2857		
rs2614192	3	114796700	0.882	-2.967	0.003005	C	T	1514/3509/2104	0.4924	0.4966	0.4743	0.4537		SIDT1
rs1499952	3	116649867	0.7637	-2.847	0.004417	A	T	28/868/6381	0.1193	0.1189	0.9213	0.06132		
rs7611371	3	118281770	1.164	3.224	0.001263	A	T	440/2748/4040	0.3802	0.376	0.3481	0.2558		
rs817510	3	119232698	1.152	2.999	0.002707	A	G	420/2748/4094	0.3784	0.372	0.1469	0.2518		
rs1580823	3	120062845	1.162	3.001	0.002688	G	T	310/2213/4757	0.304	0.3134	0.01096	0.199		
rs6764143	3	120080686	1.175	3.26	0.001112	A	T	333/2247/4697	0.3088	0.3202	0.002667	0.2049		
rs6789125	3	120080981	1.175	3.26	0.001112	G	T	333/2247/4697	0.3088	0.3202	0.002667	0.2049		
rs1521292	3	120081244	1.175	3.268	0.001082	A	G	333/2250/4697	0.3091	0.3203	0.003024	0.205		
rs962115	3	120082765	1.176	3.31	0.0009328	A	C	341/2240/4699	0.3077	0.3208	0.0005881	0.2054		

rs1938	3	120694557	1.142	2.992	0.002771	C	G	683/3094/3465	0.4272	0.4262	0.8686	0.3124	KTELC1
rs4688011	3	120709937	1.134	2.82	0.004805	A	G	664/3093/3523	0.4249	0.4229	0.6981	0.3079	C3orf1
rs4447803	3	120718667	1.134	2.818	0.00484	A	C	663/3096/3521	0.4253	0.4229	0.6575	0.3079	C3orf1
rs4461452	3	120718707	1.142	3.006	0.002649	T	G	687/3115/3478	0.4279	0.4265	0.8047	0.3128	C3orf1
rs2049502	3	120737075	0.8688	-3.049	0.002295	G	A	707/3207/3366	0.4405	0.4333	0.1596	0.3126	CD80
rs1720151	3	120748517	1.136	2.923	0.003467	G	A	833/3278/3164	0.4506	0.4487	0.7341	0.3435	CD80
rs7610229	3	130444913	0.7917	-3.137	0.001706	C	G	84/1285/5911	0.1765	0.1797	0.1335	0.09615	
rs6807994	3	130468271	0.8064	-2.909	0.003621	T	C	76/1318/5882	0.1811	0.1816	0.7964	0.09757	COPG
rs9784321	3	130470735	0.8104	-2.82	0.004799	G	A	67/1326/5807	0.1842	0.1822	0.4004	0.09819	COPG
rs6439163	3	130473107	0.7902	-3.144	0.001664	G	A	74/1301/5904	0.1787	0.1793	0.7936	0.09591	COPG
rs6771809	3	130473688	0.8104	-2.82	0.004799	C	T	67/1326/5807	0.1842	0.1822	0.4004	0.09819	COPG
rs6439164	3	130475065	0.8104	-2.82	0.004799	G	A	67/1326/5807	0.1842	0.1822	0.4004	0.09819	COPG
rs7634523	3	130485855	0.8104	-2.82	0.004799	C	A	67/1326/5807	0.1842	0.1822	0.4004	0.09819	C3orf37
rs7646495	3	130489286	0.8104	-2.82	0.004799	G	A	67/1326/5807	0.1842	0.1822	0.4004	0.09819	C3orf37
rs4075182	3	130490993	0.8104	-2.82	0.004799	C	T	67/1326/5807	0.1842	0.1822	0.4004	0.09819	C3orf37
rs11922581	3	132939357	0.8818	-2.868	0.004131	T	G	974/3309/2992	0.4548	0.4615	0.2227	0.357	CPNE4
rs10934975	3	132939505	0.8819	-2.867	0.004143	T	C	976/3312/2992	0.4549	0.4617	0.2136	0.3572	CPNE4
rs13073658	3	136048124	1.217	2.852	0.004339	A	G	71/1130/5915	0.1588	0.1628	0.04136	0.09286	EPHB1
rs12632781	3	138242420	1.133	2.957	0.003104	C	T	1146/3461/2661	0.4762	0.4783	0.713	0.4012	
rs1124328	3	144909884	1.365	3.409	0.0006519	T	C	15/609/6656	0.08365	0.08392	0.779	0.04624	SLC9A9
rs1124329	3	144910129	1.364	3.401	0.0006715	G	T	15/610/6655	0.08379	0.08405	0.7794	0.04629	SLC9A9
rs10513221	3	144914329	1.135	2.89	0.003847	C	G	806/3288/3186	0.4516	0.4466	0.3447	0.341	SLC9A9
rs9289667	3	144914613	1.135	2.884	0.003921	T	C	808/3300/3172	0.4533	0.4473	0.2599	0.3421	SLC9A9
rs1470445	3	145354795	1.133	2.996	0.002739	G	T	1381/3498/2364	0.4829	0.4908	0.1725	0.4369	
rs6774908	3	145357834	1.143	3.239	0.001201	T	C	1629/3559/2092	0.4889	0.498	0.1203	0.4734	
rs2370160	3	145360403	1.133	2.996	0.002739	G	A	1381/3498/2364	0.4829	0.4908	0.1725	0.4369	
rs6440221	3	145360763	1.133	2.996	0.002739	T	A	1381/3498/2364	0.4829	0.4908	0.1725	0.4369	
rs7615096	3	145363101	1.137	3.086	0.002026	C	A	1381/3518/2381	0.4832	0.4906	0.2054	0.4362	
rs9854202	3	145364468	1.139	3.12	0.001808	A	G	1359/3532/2389	0.4852	0.49	0.4025	0.4344	
rs6440222	3	145367382	1.134	3.025	0.002489	A	C	1381/3500/2364	0.4831	0.4908	0.1802	0.437	
rs1020376	3	145374114	1.134	3.025	0.002489	C	A	1381/3500/2364	0.4831	0.4908	0.1802	0.437	
rs2375134	3	145399350	1.132	2.92	0.003503	A	G	1354/3560/2198	0.5006	0.493	0.2023	0.4452	
rs2055529	3	145417689	1.133	2.954	0.003135	A	G	1358/3622/2294	0.4979	0.4917	0.2941	0.4402	
rs9289673	3	145441553	1.132	2.933	0.003354	G	A	1358/3621/2292	0.498	0.4917	0.2833	0.4404	
rs2375143	3	145446850	1.132	2.933	0.003354	C	T	1358/3621/2292	0.498	0.4917	0.2833	0.4404	
rs9870027	3	145448209	1.141	3.104	0.00191	T	C	1320/3511/2252	0.4957	0.4913	0.4682	0.4391	
rs1503333	3	145448734	1.132	2.933	0.003354	G	A	1358/3621/2292	0.498	0.4917	0.2833	0.4404	
rs9844107	3	149784442	1.418	2.914	0.003568	T	C	0/348/6932	0.0478	0.04666	0.0228	0.02556	
rs2638360	3	149911046	1.286	2.837	0.004555	G	A	26/632/6379	0.08981	0.09248	0.01989	0.05169	AGTR1
rs931490	3	149913457	1.286	2.837	0.004555	G	A	26/632/6379	0.08981	0.09248	0.01989	0.05169	AGTR1

rs1492103	3	149915654	1.288	2.912	0.003597	G	A	29/640/6495	0.08934	0.09269	0.004549	0.05186	AGTR1
rs3772591	3	150056374	1.35	3.213	0.001314	G	C	9/606/6665	0.08324	0.08204	0.2528	0.04512	CPB1
rs3772590	3	150057029	1.349	3.139	0.001694	G	A	8/579/6661	0.07988	0.07872	0.293	0.04333	CPB1
rs3772588	3	150057287	1.349	3.139	0.001694	G	T	8/579/6661	0.07988	0.07872	0.293	0.04333	CPB1
rs1516527	3	150092394	1.31	2.872	0.004073	T	C	8/611/6661	0.08393	0.08242	0.1523	0.04517	CPA3
rs2723359	3	153109592	1.13	2.809	0.004975	A	G	893/3435/2948	0.4721	0.4601	0.02667	0.3642	
rs3773904	3	156288098	0.7739	-2.985	0.002838	T	C	52/983/6064	0.1385	0.1414	0.09235	0.07422	MME
rs2122418	3	157633355	1.168	3.452	0.0005569	A	C	596/2953/3606	0.4127	0.4115	0.8183	0.2948	KCNAB1
rs3755632	3	157659035	0.8141	-3.097	0.001953	G	A	129/1617/5534	0.2221	0.2244	0.3749	0.1252	KCNAB1
rs3796168	3	157664734	0.7975	-3.377	0.0007341	A	G	121/1648/5509	0.2264	0.226	0.9173	0.1257	KCNAB1
rs3755633	3	157665282	0.7987	-3.339	0.0008413	A	T	122/1611/5547	0.2213	0.2223	0.6733	0.1234	KCNAB1
rs1357434	3	157665851	0.7948	-3.406	0.0006593	G	A	126/1597/5557	0.2194	0.2217	0.3687	0.1229	KCNAB1
rs16827940	3	158815195	1.16	3.163	0.001559	G	A	444/2758/4063	0.3796	0.3759	0.4171	0.2551	
rs1499787	3	158818812	1.166	3.284	0.001022	C	A	448/2764/4068	0.3797	0.3764	0.4738	0.2557	
rs16827950	3	158819160	1.16	3.163	0.001559	T	C	444/2758/4063	0.3796	0.3759	0.4171	0.2551	
rs16827960	3	158822570	1.164	3.241	0.001193	G	C	444/2755/4062	0.3794	0.3759	0.4351	0.2551	
rs16827971	3	158824362	1.164	3.241	0.001193	G	A	444/2755/4062	0.3794	0.3759	0.4351	0.2551	
rs16828010	3	158840654	1.164	3.241	0.001193	G	A	444/2755/4062	0.3794	0.3759	0.4351	0.2551	
rs2722373	3	158841357	0.8692	-3.298	0.0009747	C	T	1563/3653/1979	0.5077	0.4983	0.113	0.4663	
rs16828014	3	158841436	1.164	3.241	0.001193	A	G	444/2755/4062	0.3794	0.3759	0.4351	0.2551	
rs2649733	3	158841494	0.8692	-3.298	0.0009747	G	C	1563/3653/1979	0.5077	0.4983	0.113	0.4664	
rs1353890	3	158843091	1.17	3.316	0.0009127	C	T	444/2713/3925	0.3831	0.3792	0.3976	0.2587	
rs1392187	3	158848015	0.8775	-3.092	0.001989	A	G	1576/3705/1997	0.5091	0.4983	0.07009	0.4667	
rs1488194	3	158851711	0.8692	-3.298	0.0009747	G	T	1563/3653/1979	0.5077	0.4983	0.113	0.4663	
rs9881258	3	158854585	0.8692	-3.298	0.0009747	G	A	1563/3653/1979	0.5077	0.4983	0.113	0.4664	
rs12634684	3	158855745	1.164	3.241	0.001193	T	C	444/2755/4062	0.3794	0.3759	0.4351	0.2549	
rs2047548	3	158862059	1.177	3.408	0.0006543	T	C	439/2643/3773	0.3856	0.3817	0.4289	0.2616	
rs10804781	3	158864880	1.177	3.408	0.0006543	T	G	439/2643/3773	0.3856	0.3817	0.4289	0.2616	
rs16828114	3	158879093	1.177	3.408	0.0006543	C	T	439/2643/3773	0.3856	0.3817	0.4289	0.2616	
rs16828118	3	158881229	1.157	3.162	0.00157	G	A	508/2778/3944	0.3842	0.3871	0.5434	0.2667	
rs9290002	3	158957137	0.879	-2.992	0.002767	G	A	1151/3383/2678	0.4691	0.4776	0.1326	0.3887	
rs792742	3	158957795	0.879	-2.992	0.002767	A	G	1151/3383/2678	0.4691	0.4776	0.1326	0.3887	
rs703175	3	158960900	0.879	-2.992	0.002767	C	A	1151/3383/2678	0.4691	0.4776	0.1326	0.3886	
rs1488197	3	158962852	0.879	-2.992	0.002767	T	C	1151/3383/2678	0.4691	0.4776	0.1326	0.3886	
rs1488196	3	158962957	0.879	-2.992	0.002767	T	C	1151/3383/2678	0.4691	0.4776	0.1326	0.3886	
rs1488195	3	158963099	0.879	-2.992	0.002767	T	C	1151/3383/2678	0.4691	0.4776	0.1326	0.3887	
rs792735	3	158965740	0.879	-2.992	0.002767	A	G	1151/3383/2678	0.4691	0.4776	0.1326	0.3886	
rs703170	3	158967929	0.874	-3.135	0.00172	A	C	1154/3407/2698	0.4693	0.4774	0.1539	0.3879	
rs703172	3	158971419	0.879	-2.992	0.002767	G	A	1151/3383/2678	0.4691	0.4776	0.1326	0.3886	
rs4856714	3	162444518	1.161	2.863	0.00419	T	C	229/2225/4813	0.3062	0.301	0.1495	0.1882	NMD3

rs4290817	3	162450048	1.161	2.863	0.00419	G	A	229/2225/4813	0.3062	0.301	0.1495	0.1882	NMD3
rs7652267	3	162481191	1.16	2.858	0.004263	A	G	230/2227/4823	0.3059	0.301	0.1728	0.1881	
rs4856767	3	162487073	1.163	2.864	0.004185	C	T	223/2111/4820	0.2951	0.2935	0.6872	0.1823	
rs13083229	3	162490819	1.163	2.864	0.004185	G	A	223/2111/4820	0.2951	0.2935	0.6872	0.1823	
rs6791213	3	162516415	1.166	2.908	0.003634	A	G	223/2106/4820	0.2946	0.2933	0.7469	0.1821	
rs7618391	3	162630884	1.404	3.319	0.0009048	G	A	9/475/6741	0.06574	0.06591	0.7226	0.03597	
rs1150433	3	165135904	0.6153	-3.475	0.0005099	T	C	4/473/6802	0.06498	0.0639	0.1957	0.03124	
rs1176943	3	165141277	0.6154	-3.474	0.000512	A	G	4/473/6803	0.06497	0.06389	0.1957	0.03124	
rs11915754	3	170889680	0.8823	-2.81	0.004947	G	C	829/3288/3162	0.4517	0.4486	0.5656	0.3364	
rs9854434	3	176172614	0.8673	-3.363	0.0007703	C	G	1786/3691/1778	0.5088	0.5	0.1391	0.4966	NAALADL2
rs7613353	3	178330282	1.157	3.465	0.0005299	G	A	1236/3584/2459	0.4924	0.4859	0.2671	0.4213	TBL1XR1
rs7426456	3	178342138	1.132	2.815	0.004881	T	C	1016/3479/2554	0.4935	0.4762	0.002278	0.3952	TBL1XR1
rs12054257	3	178349813	1.132	2.836	0.004573	A	G	1249/3331/2088	0.4996	0.4921	0.2227	0.4413	TBL1XR1
rs17826680	3	183169291	0.7845	-3.141	0.001686	G	A	57/1258/5965	0.1728	0.1707	0.336	0.09148	
rs2339521	3	183173990	0.7467	-3.548	0.0003887	A	G	45/1171/6064	0.1609	0.1582	0.1817	0.08352	
rs17543305	3	184686802	0.6688	-3.269	0.001078	T	C	15/557/6708	0.07651	0.07738	0.3601	0.03801	KLHL6
rs17543340	3	184687617	0.6743	-2.884	0.003925	A	G	7/464/6745	0.0643	0.06405	1	0.03122	
rs13098866	3	187822401	0.8732	-3.147	0.001651	A	G	1345/3190/2214	0.4727	0.4917	0.001529	0.4297	
rs4634107	3	187824087	0.8786	-3.078	0.002085	G	T	1345/3443/2486	0.4733	0.4877	0.0124	0.4161	
rs17607589	3	189885280	1.466	3.202	0.001363	T	C	5/348/6834	0.04842	0.04857	0.805	0.02652	LPP
rs11709806	3	189886267	1.466	3.202	0.001363	T	C	5/348/6834	0.04842	0.04857	0.805	0.02652	LPP
rs7623253	3	189888174	1.466	3.202	0.001363	C	T	5/348/6834	0.04842	0.04857	0.805	0.02653	LPP
rs7631239	3	192168305	1.161	3.293	0.0009923	A	T	570/2931/3779	0.4026	0.4028	0.9536	0.2841	
rs1587584	3	192181578	1.143	2.919	0.003516	A	G	537/2817/3926	0.387	0.3916	0.3089	0.2711	
rs7645099	3	192203182	1.15	3.025	0.002487	A	C	498/2823/3959	0.3878	0.387	0.8797	0.2664	
rs9816805	3	194920203	0.766	-2.836	0.004568	A	G	54/782/6117	0.1125	0.1198	3.04E-06	0.06163	
rs11717055	3	194934451	0.8652	-2.89	0.003856	G	A	429/2655/4196	0.3647	0.3661	0.7489	0.2376	
rs6794037	3	196270452	0.8689	-3.087	0.002019	T	C	754/3238/3287	0.4448	0.4395	0.3108	0.3223	C3orf21
rs1389726	3	196280291	0.8695	-2.928	0.003414	G	A	558/2985/3737	0.41	0.4047	0.271	0.2781	C3orf21
rs7670059	4	4442549	0.8871	-2.86	0.004238	T	G	1598/3554/2083	0.4912	0.4978	0.267	0.4614	D4S234E
rs13128917	4	4446605	1.141	2.828	0.004678	A	G	589/2972/3293	0.4336	0.4222	0.02564	0.3075	D4S234E
rs17475461	4	9896775	1.291	3.013	0.002587	C	T	27/696/6348	0.09843	0.1004	0.09652	0.05591	
rs41496445	4	12557772	1.158	3.099	0.001943	T	C	422/2641/4207	0.3633	0.3645	0.7722	0.2443	
rs16869987	4	20414863	0.8878	-2.81	0.004948	A	C	1634/3654/1926	0.5065	0.4992	0.22	0.475	KCNIP4
rs7676498	4	21708853	1.313	3.061	0.002208	T	C	19/684/6566	0.0941	0.09439	0.803	0.05123	
rs16867660	4	21711952	1.286	2.879	0.003991	A	T	23/712/6529	0.09802	0.09891	0.4071	0.05379	
rs9996455	4	21723360	1.285	2.863	0.004197	C	T	23/713/6529	0.09814	0.09902	0.4082	0.05384	
rs6856464	4	26432096	0.6923	-3.386	0.0007105	G	A	23/692/6558	0.09515	0.09632	0.274	0.04785	
rs1486770	4	30290763	1.44	2.898	0.003758	C	G	8/280/6992	0.03846	0.03983	0.01019	0.02194	
rs10025805	4	30942714	0.8865	-2.825	0.004731	C	T	1230/3485/2564	0.4788	0.4832	0.4376	0.4027	

rs4832912	4	37195425	0.8626	-3.126	0.001771	C	T	595/3107/3558	0.428	0.4167	0.02252	0.2921	
rs17577082	4	37206372	0.8688	-2.827	0.004695	T	C	441/2736/4102	0.3759	0.3735	0.6156	0.2451	
rs17604878	4	37207327	0.8641	-2.927	0.00342	C	T	439/2729/4102	0.3754	0.3731	0.615	0.2444	C4orf19
rs6833071	4	37696208	1.129	2.874	0.004058	G	C	1007/3289/2980	0.452	0.4632	0.04036	0.3686	TBC1D1
rs933820	4	37702617	1.138	2.957	0.003102	G	A	841/3249/3182	0.4468	0.4482	0.7936	0.3429	TBC1D1
rs6820715	4	37708185	1.169	3.52	0.0004309	A	T	678/3029/3573	0.4161	0.4209	0.3298	0.3059	TBC1D1
rs10018836	4	37708322	1.165	3.445	0.00057	C	T	682/3030/3568	0.4162	0.4214	0.2907	0.3064	TBC1D1
rs6818409	4	37714120	1.169	3.52	0.0004309	T	C	678/3029/3573	0.4161	0.4209	0.3298	0.3059	TBC1D1
rs10023902	4	37717009	1.146	3.119	0.001814	G	C	812/3170/3289	0.436	0.442	0.2539	0.334	TBC1D1
rs4832981	4	37719161	1.169	3.52	0.0004309	T	C	678/3029/3573	0.4161	0.4209	0.3298	0.3059	TBC1D1
rs10018389	4	37722964	1.165	3.445	0.00057	T	C	682/3030/3568	0.4162	0.4214	0.2907	0.3063	TBC1D1
rs6845120	4	37723940	1.153	3.27	0.001074	A	G	799/3168/3313	0.4352	0.4404	0.3119	0.3322	TBC1D1
rs7674408	4	37729055	1.155	3.323	0.0008898	G	T	819/3141/3295	0.4329	0.4418	0.089	0.3342	TBC1D1
rs9991524	4	37729601	1.154	3.269	0.001079	C	T	790/3165/3313	0.4355	0.4397	0.4083	0.3313	TBC1D1
rs6823014	4	37730977	1.169	3.52	0.0004309	A	G	678/3029/3573	0.4161	0.4209	0.3298	0.3059	TBC1D1
rs10031594	4	37734938	1.155	3.247	0.001165	G	T	694/3042/3544	0.4179	0.4234	0.2682	0.3085	TBC1D1
rs2052694	4	37735221	1.165	3.445	0.00057	T	C	682/3030/3568	0.4162	0.4214	0.2907	0.3064	TBC1D1
rs1863328	4	37736691	1.155	3.323	0.0008898	A	G	819/3141/3295	0.4329	0.4418	0.089	0.3342	TBC1D1
rs1863329	4	37736880	1.142	2.971	0.002969	T	C	726/3168/3313	0.4396	0.4356	0.4489	0.3247	TBC1D1
rs933824	4	37741087	1.169	3.52	0.0004309	A	G	678/3029/3573	0.4161	0.4209	0.3298	0.3059	TBC1D1
rs2303420	4	37745472	1.169	3.52	0.0004309	C	A	678/3029/3573	0.4161	0.4209	0.3298	0.3059	TBC1D1
rs2052693	4	37747001	1.153	3.273	0.001065	A	T	819/3166/3295	0.4349	0.4422	0.1601	0.3347	TBC1D1
rs4832984	4	37747334	1.15	3.218	0.00129	T	A	820/3171/3289	0.4356	0.4425	0.1854	0.335	TBC1D1
rs1364952	4	37749028	1.163	3.399	0.000677	G	A	695/3046/3539	0.4184	0.4237	0.2932	0.3091	TBC1D1
rs4832986	4	37760209	1.163	3.399	0.000677	A	C	695/3046/3539	0.4184	0.4237	0.2932	0.309	TBC1D1
rs1863325	4	37763500	1.159	3.335	0.0008522	T	C	690/3044/3546	0.4181	0.423	0.3187	0.3082	TBC1D1
rs9306956	4	37764200	1.169	3.52	0.0004309	C	T	678/3029/3573	0.4161	0.4209	0.3298	0.3059	TBC1D1
rs715344	4	37774349	1.169	3.52	0.0004309	T	C	678/3029/3573	0.4161	0.4209	0.3298	0.3059	TBC1D1
rs6813350	4	42657750	0.498	-3.057	0.002239	G	A	0/227/7053	0.03118	0.0307	0.4252	0.01419	LOC389207
rs6447287	4	43315984	0.7678	-3.003	0.002677	A	G	34/997/6235	0.1372	0.1358	0.4368	0.07116	
rs6852448	4	46981441	1.425	2.816	0.004866	A	T	10/285/6912	0.03954	0.04142	0.001375	0.02284	GABRB1
rs2078610	4	46983811	1.368	3.019	0.002539	G	T	15/436/6829	0.05989	0.06196	0.01161	0.03398	GABRB1
rs733873	4	46985469	1.425	2.843	0.004466	T	C	10/290/6900	0.04028	0.04213	0.00177	0.02326	GABRB1
rs9683412	4	46997541	1.518	3.015	0.002573	T	A	5/232/7031	0.03192	0.03274	0.0502	0.01823	GABRB1
rs4235146	4	46999617	1.518	3.015	0.002573	G	T	5/232/7031	0.03192	0.03274	0.0502	0.01823	GABRB1
rs4518219	4	46999789	1.518	3.015	0.002573	C	T	5/232/7031	0.03192	0.03274	0.0502	0.01823	GABRB1
rs11929757	4	47003606	1.541	3.531	0.0004142	G	A	6/301/6973	0.04135	0.04207	0.1517	0.0234	GABRB1
rs13129278	4	47016325	1.588	3.575	0.0003505	A	G	4/264/7003	0.03631	0.03671	0.3235	0.02063	GABRB1
rs13108343	4	47037983	1.588	3.575	0.0003505	C	G	4/264/7003	0.03631	0.03671	0.3235	0.02063	GABRB1
rs12503495	4	47038399	1.589	3.579	0.0003443	C	A	4/264/7003	0.03631	0.03671	0.3235	0.02063	GABRB1

rs17461709	4	47042260	1.547	3.561	0.0003693	A	G	7/292/6980	0.04012	0.04116	0.04139	0.02301	GABRB1
rs2898669	4	52836126	1.14	2.874	0.004054	G	T	605/2946/3558	0.4144	0.4137	0.9087	0.2959	
rs10780093	4	52847040	1.14	2.874	0.004054	T	C	605/2946/3558	0.4144	0.4137	0.9087	0.2959	
rs7658023	4	52847160	1.14	2.874	0.004054	A	G	605/2946/3558	0.4144	0.4137	0.9087	0.2959	
rs7677189	4	52847239	1.14	2.874	0.004054	G	A	605/2946/3558	0.4144	0.4137	0.9087	0.2959	
rs7683334	4	52847986	1.14	2.874	0.004054	G	A	605/2946/3558	0.4144	0.4137	0.9087	0.2959	
rs8180165	4	52848663	1.14	2.874	0.004054	A	C	605/2946/3558	0.4144	0.4137	0.9087	0.2959	
rs967344	4	52913877	1.141	2.854	0.004318	T	C	524/2818/3938	0.3871	0.39	0.528	0.269	
rs17051547	4	53115929	1.262	3.026	0.002475	A	C	38/924/6318	0.1269	0.1279	0.5205	0.07104	
rs17051548	4	53116582	1.271	3.037	0.002392	A	G	35/862/6330	0.1193	0.1206	0.3289	0.06685	
rs17051550	4	53120700	1.272	3.137	0.001707	G	C	37/923/6320	0.1268	0.1276	0.5812	0.07092	
rs17051554	4	53123605	1.272	3.086	0.002028	T	C	37/882/6323	0.1218	0.1233	0.2939	0.06842	
rs11936731	4	53125509	1.272	3.086	0.002028	A	G	37/882/6323	0.1218	0.1233	0.2939	0.06848	
rs346009	4	53128345	1.189	2.888	0.003878	T	C	123/1644/5512	0.2259	0.2259	0.9586	0.1326	
rs346010	4	53128445	1.191	2.908	0.003637	C	A	122/1617/5540	0.2221	0.223	0.7524	0.1307	
rs17051563	4	53138698	1.186	2.929	0.003405	A	C	140/1726/5411	0.2372	0.2377	0.8437	0.1409	
rs13434881	4	53149005	1.174	2.872	0.004073	T	G	175/1863/5241	0.2559	0.2578	0.5247	0.1549	
rs6832794	4	53160037	1.211	2.842	0.004489	C	T	71/1255/5418	0.1861	0.1857	0.9477	0.1068	USP46
rs10517263	4	53193426	1.233	2.968	0.002999	C	G	56/1120/6104	0.1538	0.1549	0.5449	0.08693	USP46
rs17051640	4	53196288	1.247	2.992	0.00277	T	A	41/1031/6063	0.1445	0.1438	0.7424	0.08064	USP46
rs7691678	4	53208526	1.243	2.834	0.004594	A	G	36/939/6166	0.1315	0.1316	0.9284	0.07312	USP46
rs11939490	4	53218589	1.224	2.864	0.004186	G	A	55/1118/6107	0.1536	0.1545	0.5957	0.08652	P46 LOC6437
rs7699089	4	53231790	1.171	2.824	0.004744	C	T	175/1857/5248	0.2551	0.2572	0.4942	0.1544	
rs11946547	4	53233472	1.243	2.828	0.004687	A	G	36/940/6161	0.1317	0.1317	0.9285	0.07326	
rs12513298	4	53234449	1.173	2.852	0.004349	T	G	173/1858/5249	0.2552	0.2569	0.584	0.1542	
rs746687	4	53238171	1.175	2.836	0.00457	A	G	164/1803/5240	0.2502	0.252	0.5433	0.1507	
rs12505070	4	53243407	1.173	2.849	0.004388	A	C	173/1858/5248	0.2553	0.2569	0.584	0.1542	
rs10517266	4	53244400	1.243	2.834	0.004594	C	T	36/939/6166	0.1315	0.1316	0.9284	0.07317	
rs12500617	4	53247384	1.243	2.828	0.004687	A	G	36/940/6161	0.1317	0.1317	0.9285	0.07326	
rs7674000	4	53252435	1.243	2.828	0.004687	A	C	36/940/6161	0.1317	0.1317	0.9285	0.07322	
rs2411261	4	53266955	1.243	2.834	0.004594	G	C	36/939/6166	0.1315	0.1316	0.9284	0.07317	
rs10028122	4	53272766	1.17	2.814	0.004888	A	G	180/1891/5145	0.2621	0.2633	0.6874	0.1588	
rs6817996	4	53293825	1.172	2.877	0.00401	A	G	192/1951/5050	0.2712	0.2719	0.8284	0.1657	
rs4865407	4	53313898	1.184	2.913	0.003581	C	G	147/1774/4827	0.2629	0.2595	0.302	0.1573	
rs10009096	4	53318494	1.177	2.81	0.00496	T	C	147/1769/4826	0.2624	0.2592	0.3239	0.1569	
rs7697631	4	53318876	1.177	2.81	0.00496	T	C	147/1769/4826	0.2624	0.2592	0.3239	0.1569	
rs7660838	4	53319145	1.177	2.81	0.00496	G	A	147/1769/4826	0.2624	0.2592	0.3239	0.1569	
rs4864679	4	53392503	1.146	2.815	0.004881	A	G	369/2421/4490	0.3326	0.3398	0.0728	0.2218	
rs2279334	4	53427202	1.147	2.841	0.004498	T	C	368/2428/4484	0.3335	0.3402	0.09811	0.2222	RASL11B
rs2062253	4	53427815	1.152	2.933	0.003354	C	G	368/2434/4478	0.3343	0.3406	0.1136	0.2227	RASL11B

rs2166020	4	54935698	1.627	3.066	0.002172	C	T	1/174/7065	0.02403	0.02401	1	0.01362	
rs6844962	4	60289250	1.548	2.955	0.003128	C	T	2/213/6999	0.02953	0.02963	0.6781	0.01631	
rs6855997	4	60289374	1.548	2.955	0.003128	G	A	2/213/6999	0.02953	0.02963	0.6781	0.01631	
rs13138017	4	65717253	0.5308	-2.944	0.003239	C	T	1/237/7026	0.03263	0.03236	1	0.01526	
rs13118169	4	65754311	0.5308	-2.944	0.003239	T	C	1/237/7026	0.03263	0.03236	1	0.01526	
rs13151731	4	65764069	0.5308	-2.944	0.003239	T	C	1/237/7026	0.03263	0.03236	1	0.01526	
rs13151338	4	65771813	0.5308	-2.944	0.003239	C	T	1/237/7026	0.03263	0.03236	1	0.01526	
rs2292574	4	65772673	0.5308	-2.944	0.003239	T	C	1/237/7026	0.03263	0.03236	1	0.01526	
rs13149941	4	65781847	0.5308	-2.944	0.003239	C	T	1/237/7026	0.03263	0.03236	1	0.01526	
rs13144545	4	65781968	0.5308	-2.944	0.003239	C	A	1/237/7026	0.03263	0.03236	1	0.01526	
rs9991142	4	69967176	1.278	2.902	0.003708	C	T	26/696/6551	0.0957	0.09756	0.1163	0.05406	
rs7680341	4	69981889	1.302	2.851	0.004363	G	A	15/597/6616	0.0826	0.08298	0.6695	0.04565	
rs7435335	4	70005924	1.277	2.883	0.003936	A	G	25/696/6555	0.09566	0.09727	0.1486	0.05388	UGT2B7
rs11249529	4	70032222	1.277	2.883	0.003936	C	A	25/696/6555	0.09566	0.09727	0.1486	0.05388	
rs1826684	4	70350594	1.136	2.839	0.00452	G	A	690/3062/3519	0.4211	0.4243	0.525	0.3095	
rs1826685	4	70354696	1.135	2.825	0.004727	C	T	683/3049/3501	0.4215	0.4241	0.6177	0.3092	
rs2642859	4	70355118	1.135	2.825	0.004727	G	A	683/3049/3501	0.4215	0.4241	0.6177	0.3093	
rs2642861	4	70356560	1.135	2.825	0.004727	G	A	683/3049/3501	0.4215	0.4241	0.6177	0.3092	
rs2736471	4	70356658	1.135	2.825	0.004727	T	C	683/3049/3501	0.4215	0.4241	0.6177	0.3092	
rs2736466	4	70361097	1.135	2.825	0.004727	A	T	683/3049/3501	0.4215	0.4241	0.6177	0.3092	
rs1353280	4	70362629	1.135	2.825	0.004727	C	T	683/3049/3501	0.4215	0.4241	0.6177	0.3091	
rs6600937	4	70368350	1.135	2.825	0.004727	G	A	683/3049/3501	0.4215	0.4241	0.6177	0.3093	
rs903446	4	70371090	1.135	2.825	0.004727	A	G	683/3049/3501	0.4215	0.4241	0.6177	0.3092	
rs2010468	4	70371820	1.135	2.825	0.004727	C	T	683/3049/3501	0.4215	0.4241	0.6177	0.3093	
rs6816748	4	70421904	0.5769	-2.839	0.004527	G	A	2/260/7011	0.03575	0.03564	1	0.01681	
rs7681169	4	73347021	0.8789	-2.941	0.003274	A	G	988/3338/2954	0.4585	0.4635	0.3627	0.3595	
rs10433664	4	73347736	0.8832	-2.824	0.004739	A	G	978/3349/2951	0.4602	0.4633	0.5777	0.3592	
rs16848920	4	74019167	1.153	2.85	0.004368	A	G	332/2550/4168	0.3617	0.352	0.02135	0.2324	
rs2366703	4	74021287	1.154	2.866	0.00416	G	A	330/2550/4168	0.3618	0.3517	0.0162	0.2322	
rs10938085	4	74615443	1.152	2.935	0.003337	T	C	380/2540/4354	0.3492	0.3508	0.7131	0.2316	
rs1247668	4	74665660	1.21	2.943	0.00325	A	G	69/1396/5715	0.1944	0.1908	0.1217	0.1105	RASSF6
rs1247670	4	74666316	1.217	2.944	0.003236	C	T	58/1308/5891	0.1802	0.177	0.1268	0.1018	RASSF6
rs1247671	4	74666374	1.217	2.944	0.003236	A	C	58/1308/5891	0.1802	0.177	0.1268	0.1017	RASSF6
rs2008288	4	74666664	1.21	2.943	0.00325	G	A	69/1396/5715	0.1944	0.1908	0.1217	0.1105	RASSF6
rs16849628	4	74667180	1.21	2.943	0.00325	T	A	69/1396/5715	0.1944	0.1908	0.1217	0.1105	RASSF6
rs7682201	4	74669694	1.197	2.837	0.00456	A	G	77/1474/5729	0.2025	0.1986	0.1107	0.1153	RASSF6
rs12507775	4	74669937	1.204	2.926	0.003434	G	A	74/1458/5748	0.2003	0.1963	0.08345	0.114	ASSF6 RASSF
rs12503916	4	74670123	1.206	2.95	0.003178	T	C	71/1458/5750	0.2003	0.1957	0.04773	0.1136	RASSF6
rs16849677	4	74671052	1.209	2.952	0.003159	C	T	69/1417/5749	0.1959	0.1918	0.07563	0.1112	RASSF6
rs1528917	4	74671366	1.209	2.952	0.003159	C	G	69/1417/5749	0.1959	0.1918	0.07563	0.1111	RASSF6

rs10019146	4	74672520	1.209	2.952	0.003159	A	G	69/1417/5749	0.1959	0.1918	0.07563	0.1112	RASSF6
rs13144296	4	74672852	1.209	2.952	0.003159	G	A	69/1417/5749	0.1959	0.1918	0.07563	0.1111	RASSF6
rs9999939	4	74672868	1.209	2.952	0.003159	T	C	69/1417/5749	0.1959	0.1918	0.07563	0.1112	RASSF6
rs17192413	4	74678842	1.216	2.936	0.003328	T	A	58/1310/5891	0.1805	0.1772	0.127	0.1019	RASSF6
rs16849727	4	74679608	1.209	2.952	0.003159	A	C	69/1417/5749	0.1959	0.1918	0.07563	0.1112	RASSF6
rs12500338	4	76412721	1.252	3.501	0.0004633	G	C	81/1328/5863	0.1826	0.1839	0.5658	0.1059	LOC643100
rs12641387	4	87449625	1.179	3.454	0.0005529	T	A	396/2601/4275	0.3577	0.3577	1	0.2367	MAPK10
rs17449485	4	87452841	1.17	3.273	0.001065	A	G	401/2519/4257	0.351	0.3557	0.2734	0.2354	MAPK10
rs17409478	4	87463858	0.8625	-2.821	0.004789	A	G	344/2414/4510	0.3321	0.3357	0.3637	0.2107	MAPK10
rs4693141	4	87471006	0.8526	-3.794	0.0001482	G	A	1566/3627/2072	0.4992	0.4976	0.7953	0.4605	MAPK10
rs17409904	4	87475230	0.8526	-3.794	0.0001482	T	C	1566/3627/2072	0.4992	0.4976	0.7953	0.4606	MAPK10
rs4611890	4	87478030	0.8505	-3.859	0.000114	C	T	1569/3633/2078	0.499	0.4976	0.8138	0.4603	MAPK10
rs4608765	4	87478292	0.8526	-3.794	0.0001482	T	C	1566/3627/2072	0.4992	0.4976	0.7953	0.4606	MAPK10
rs7688651	4	87478829	0.8625	-2.821	0.004789	T	C	344/2414/4510	0.3321	0.3357	0.3637	0.2108	MAPK10
rs11932806	4	87532478	0.8635	-3.489	0.0004848	T	C	1533/3630/2102	0.4997	0.4969	0.6537	0.4567	MAPK10
rs4597789	4	87541401	0.8635	-3.489	0.0004848	A	G	1533/3630/2102	0.4997	0.4969	0.6537	0.4569	MAPK10
rs6823664	4	87545748	0.8635	-3.489	0.0004848	T	C	1533/3630/2102	0.4997	0.4969	0.6537	0.4569	MAPK10
rs12639691	4	91489527	0.8487	-2.857	0.004274	G	C	224/2102/4861	0.2925	0.2919	0.9035	0.1734	MGC48628
rs10008329	4	98500923	1.127	2.85	0.004366	C	T	1570/3477/2032	0.4912	0.4979	0.2619	0.4721	
rs11097560	4	98505949	1.127	2.85	0.004366	A	G	1570/3477/2032	0.4912	0.4979	0.2619	0.4722	
rs7441186	4	98506195	1.127	2.85	0.004366	A	G	1570/3477/2032	0.4912	0.4979	0.2619	0.4722	
rs1350971	4	98510402	1.136	3.067	0.002162	C	T	1569/3614/2095	0.4966	0.4974	0.8876	0.4689	
rs1350970	4	98510697	1.127	2.85	0.004366	C	A	1570/3477/2032	0.4912	0.4979	0.2619	0.4721	
rs10010255	4	98515553	1.127	2.85	0.004366	A	G	1570/3477/2032	0.4912	0.4979	0.2619	0.4722	
rs7682461	4	98516722	1.127	2.85	0.004366	T	G	1570/3477/2032	0.4912	0.4979	0.2619	0.4722	
rs4699559	4	98518159	1.136	3.075	0.002108	G	A	1569/3615/2096	0.4966	0.4974	0.8876	0.4689	
rs7668790	4	98521978	1.131	2.959	0.003086	G	A	1590/3624/2066	0.4978	0.4979	1	0.4722	
rs7669443	4	98522277	1.131	2.959	0.003086	G	A	1590/3624/2066	0.4978	0.4979	1	0.4722	
rs2089930	4	98523158	1.131	2.959	0.003086	A	G	1590/3624/2066	0.4978	0.4979	1	0.4721	
rs986825	4	98526951	1.131	2.959	0.003086	G	A	1590/3624/2066	0.4978	0.4979	1	0.4721	
rs4699562	4	98530533	1.131	2.959	0.003086	T	G	1590/3624/2066	0.4978	0.4979	1	0.4722	
rs4699563	4	98530848	1.131	2.959	0.003086	C	A	1590/3624/2066	0.4978	0.4979	1	0.4722	
rs233815	4	103426830	1.272	2.963	0.003051	T	G	29/815/6204	0.1156	0.1162	0.6812	0.06469	SLC39A8
rs233809	4	103431453	1.272	2.963	0.003051	T	G	29/815/6204	0.1156	0.1162	0.6812	0.06469	SLC39A8
rs233808	4	103431479	1.231	2.837	0.004547	T	C	40/1056/6180	0.1451	0.1439	0.5681	0.08076	SLC39A8
rs2522474	4	105965267	1.137	3.068	0.002154	G	A	1123/3374/2783	0.4635	0.474	0.0602	0.3925	
rs2725771	4	105978188	1.129	2.923	0.003468	G	T	1233/3395/2652	0.4663	0.481	0.009791	0.4088	
rs2725770	4	105978874	1.143	3.192	0.00141	A	T	1116/3346/2818	0.4596	0.4727	0.0185	0.3897	
rs2046880	4	106590265	0.8798	-2.931	0.003374	T	A	1587/3502/1644	0.5201	0.5	0.0009984	0.4922	PPA2
rs2023840	4	106592037	0.8798	-2.931	0.003374	A	G	1587/3502/1644	0.5201	0.5	0.0009984	0.4922	PPA2

rs4699176	4	106608151	1.129	2.928	0.003411	T	G	1616/3544/2041	0.4922	0.4983	0.2981	0.4749	PPA2
rs2298732	4	106610542	1.129	2.928	0.003411	A	G	1616/3544/2041	0.4922	0.4983	0.2981	0.4749	PPA2
rs2866883	4	106610814	1.131	2.961	0.003063	C	T	1616/3499/2005	0.4914	0.4985	0.2346	0.4773	PPA2
rs7678401	4	106611329	1.129	2.928	0.003411	A	G	1616/3544/2041	0.4922	0.4983	0.2981	0.4749	PPA2
rs6533199	4	106615593	1.129	2.928	0.003411	A	G	1616/3544/2041	0.4922	0.4983	0.2981	0.4749	
rs1125254	4	106624072	1.129	2.928	0.003411	A	G	1616/3544/2041	0.4922	0.4983	0.2981	0.4749	
rs3864225	4	106627928	1.131	2.961	0.003063	G	T	1616/3499/2005	0.4914	0.4985	0.2346	0.4773	
rs3855585	4	106628127	1.131	2.961	0.003063	G	A	1616/3499/2005	0.4914	0.4985	0.2346	0.4773	
rs11097891	4	106628375	1.131	2.961	0.003063	T	C	1616/3499/2005	0.4914	0.4985	0.2346	0.4772	
rs13110343	4	106628645	1.131	2.961	0.003063	G	A	1616/3499/2005	0.4914	0.4985	0.2346	0.4773	
rs2866806	4	106628936	1.131	2.961	0.003063	C	T	1616/3499/2005	0.4914	0.4985	0.2346	0.4773	
rs765220	4	106631208	1.129	2.928	0.003411	T	G	1616/3544/2041	0.4922	0.4983	0.2981	0.4748	
rs757176	4	106631978	1.129	2.928	0.003411	A	C	1616/3544/2041	0.4922	0.4983	0.2981	0.4749	
rs4699182	4	106633149	1.13	2.958	0.003098	A	C	1658/3576/2043	0.4914	0.4986	0.2215	0.4779	
rs6825111	4	106638219	1.129	2.928	0.003411	G	A	1616/3544/2041	0.4922	0.4983	0.2981	0.4749	
rs6855572	4	106638387	1.129	2.928	0.003411	T	A	1616/3544/2041	0.4922	0.4983	0.2981	0.4749	
rs6831138	4	106639029	1.129	2.928	0.003411	A	G	1616/3544/2041	0.4922	0.4983	0.2981	0.4749	
rs4698941	4	106641985	1.129	2.928	0.003411	G	A	1616/3544/2041	0.4922	0.4983	0.2981	0.4749	
rs9999896	4	106643206	1.131	2.961	0.003063	C	T	1616/3499/2005	0.4914	0.4985	0.2346	0.4773	
rs6816997	4	106644038	1.131	2.961	0.003063	A	T	1616/3499/2005	0.4914	0.4985	0.2346	0.4772	
rs763289	4	106645725	1.129	2.928	0.003411	A	G	1616/3544/2041	0.4922	0.4983	0.2981	0.4749	
rs10020998	4	106647903	1.129	2.928	0.003411	A	G	1616/3544/2041	0.4922	0.4983	0.2981	0.4749	
rs10024331	4	106649118	1.132	2.975	0.002933	A	C	1616/3493/1990	0.492	0.4986	0.2735	0.4782	
rs3796941	4	111149662	1.184	3.126	0.001773	G	A	218/1841/4558	0.2782	0.2849	0.0576	0.1752	EGF
rs7684808	4	113940777	1.337	3.184	0.001453	T	C	13/621/6646	0.0853	0.08492	0.8897	0.04702	
rs7685179	4	113940935	1.337	3.184	0.001453	T	G	13/621/6646	0.0853	0.08492	0.8897	0.04702	
rs4626206	4	113947581	1.329	3.194	0.001404	C	A	23/612/6645	0.08407	0.0863	0.0397	0.04786	
rs1550952	4	113955448	1.341	3.206	0.001348	A	G	13/615/6644	0.08457	0.08426	0.8892	0.04669	
rs1979086	4	113956986	1.341	3.206	0.001348	A	T	13/615/6644	0.08457	0.08426	0.8892	0.04669	
rs10026837	4	113959159	1.341	3.206	0.001348	G	C	13/615/6644	0.08457	0.08426	0.8892	0.04668	ANK2
rs7681443	4	113967241	1.349	3.279	0.00104	G	A	13/615/6644	0.08457	0.08426	0.8892	0.04674	ANK2
rs6844637	4	113967887	1.349	3.279	0.00104	A	G	13/615/6644	0.08457	0.08426	0.8892	0.04674	ANK2
rs6822647	4	113971506	1.349	3.279	0.00104	A	G	13/615/6644	0.08457	0.08426	0.8892	0.04674	ANK2
rs6533657	4	113973128	1.354	3.326	0.0008816	T	C	13/616/6649	0.08464	0.08432	0.8893	0.04681	
rs4834307	4	113973913	1.343	3.236	0.001212	T	C	13/622/6645	0.08544	0.08505	0.8899	0.04713	
rs1550953	4	113975455	1.348	3.266	0.001092	T	G	13/616/6644	0.0847	0.08438	0.8893	0.04679	ANK2
rs7686651	4	123247981	1.144	3.094	0.001972	G	A	853/3302/3125	0.4536	0.4513	0.6778	0.3475	
rs7692155	4	123248494	1.142	3.052	0.002275	G	A	835/3315/3130	0.4554	0.4503	0.3488	0.3459	
rs17013253	4	129530662	1.212	3.115	0.001842	C	G	91/1522/5666	0.2091	0.2067	0.364	0.1202	
rs17013256	4	129532392	1.211	3.102	0.001924	A	G	91/1523/5664	0.2093	0.2068	0.3355	0.1203	

rs10518530	4	129534699	1.201	2.961	0.003066	C	A	88/1555/5619	0.2141	0.21	0.09363	0.122	
rs17013275	4	129559075	1.208	3.078	0.002083	A	G	90/1568/5617	0.2155	0.2114	0.1075	0.1232	
rs10049881	4	129565455	1.207	3.249	0.001158	C	G	126/1817/5315	0.2503	0.2444	0.04353	0.146	
rs1106860	4	129567235	1.176	3.368	0.0007563	A	T	382/2648/4245	0.364	0.359	0.253	0.2387	
rs4000688	4	129580449	1.229	3.652	0.0002607	C	T	147/1817/5315	0.2496	0.248	0.6031	0.1489	
rs11933959	4	129581675	1.176	3.368	0.0007563	C	G	382/2648/4245	0.364	0.359	0.253	0.2387	
rs4572893	4	129589323	1.211	3.454	0.0005533	A	T	173/1820/5287	0.25	0.2533	0.2669	0.1595	
rs4246727	4	129589968	1.169	3.236	0.001211	A	G	378/2650/4245	0.3644	0.3587	0.1802	0.2381	
rs17013315	4	129592875	1.229	3.654	0.0002583	T	C	147/1817/5316	0.2496	0.2479	0.6031	0.149	
rs3956594	4	129595154	1.229	3.654	0.0002583	C	G	147/1817/5316	0.2496	0.2479	0.6031	0.1489	
rs9307607	4	129595331	1.229	3.654	0.0002583	T	C	147/1817/5316	0.2496	0.2479	0.6031	0.149	
rs4975231	4	129598021	1.175	3.359	0.0007822	G	A	382/2650/4245	0.3642	0.3591	0.2399	0.2388	
rs7442153	4	129598471	1.169	3.236	0.001211	A	G	378/2650/4245	0.3644	0.3587	0.1802	0.2382	
rs4498144	4	129600289	1.175	3.359	0.0007822	C	T	382/2650/4245	0.3642	0.3591	0.2399	0.2388	
rs17613752	4	135147623	1.809	2.955	0.003122	T	C	0/107/7126	0.01479	0.01468	1	0.00817	
rs1390009	4	135151781	1.809	2.955	0.003122	G	A	0/107/7126	0.01479	0.01468	1	0.00817	
rs17614004	4	135162481	1.809	2.955	0.003122	G	C	0/107/7126	0.01479	0.01468	1	0.00817	
rs7659803	4	135163038	1.809	2.955	0.003122	C	A	0/107/7126	0.01479	0.01468	1	0.00817	
rs17614300	4	135185863	1.757	2.821	0.004784	G	A	0/110/7130	0.01519	0.01508	1	0.00833	
rs1509260	4	138904549	0.8068	-2.853	0.004337	G	A	60/1315/5904	0.1807	0.1777	0.1662	0.09601	
rs2162337	4	139596509	0.7972	-2.934	0.003343	T	A	59/1261/5809	0.1769	0.1747	0.3422	0.09432	
rs1834624	4	139597211	0.7972	-2.934	0.003343	G	T	59/1261/5809	0.1769	0.1747	0.3422	0.09428	
rs2291713	4	141764847	1.212	3.268	0.001083	C	A	124/1624/5532	0.2231	0.2241	0.7142	0.1321	TBC1D9
rs4956463	4	141765995	1.21	3.237	0.001209	G	C	124/1627/5529	0.2235	0.2244	0.7148	0.1322	TBC1D9
rs2115990	4	141772950	1.193	3.009	0.002624	A	G	130/1610/5540	0.2212	0.2239	0.2952	0.1317	TBC1D9
rs6813507	4	141780803	1.223	3.439	0.0005832	T	G	128/1597/5555	0.2194	0.2221	0.2911	0.1309	TBC1D9
rs2139383	4	142764901	0.8195	-2.958	0.003095	C	T	92/1667/5521	0.229	0.2219	0.00601	0.1238	
rs7349640	4	142870351	0.7578	-2.96	0.003077	G	A	24/914/6243	0.1273	0.125	0.1308	0.06461	IL15
rs6842797	4	144736564	1.228	3.033	0.002423	A	T	1/1655/5378	0.2353	0.2078	1.27E-46	0.1213	FREM3
rs1497609	4	144774271	1.248	2.907	0.003644	C	T	36/968/6184	0.1347	0.1342	0.8604	0.07496	FREM3
rs13131507	4	144780398	1.254	2.966	0.003012	A	T	36/961/6208	0.1334	0.1331	0.9295	0.07435	FREM3
rs1826927	4	144793589	1.225	2.821	0.004786	G	T	58/1008/6213	0.1385	0.1425	0.02083	0.07989	FREM3
rs6537170	4	144832350	1.242	2.842	0.004478	A	T	38/934/6301	0.1284	0.1292	0.5857	0.07213	FREM3
rs1545437	4	144837292	1.264	3.03	0.002441	T	G	37/887/6320	0.1224	0.1239	0.3417	0.06926	FREM3
rs4835333	4	144843777	1.297	2.994	0.002753	G	T	22/682/6575	0.09369	0.09477	0.3216	0.05246	FREM3
rs11728121	4	144847682	1.287	2.914	0.003572	C	T	22/692/6566	0.09505	0.09599	0.3918	0.05307	
rs6835827	4	144853406	1.291	2.943	0.00325	T	C	22/691/6567	0.09492	0.09586	0.3908	0.05302	
rs1450250	4	144872476	1.299	3.012	0.002596	A	G	22/681/6577	0.09354	0.09463	0.3205	0.0523	
rs4350958	4	144885259	1.331	3.425	0.0006153	T	C	24/741/6510	0.1019	0.1026	0.5664	0.0573	
rs9685306	4	144889446	1.373	2.837	0.004551	A	G	6/401/6575	0.05743	0.0574	1	0.03151	

rs13129993	4	144903679	1.373	2.837	0.004551	A	T	6/401/6575	0.05743	0.0574	1	0.03151	
rs1580002	4	144907110	1.373	2.837	0.004551	A	G	6/401/6575	0.05743	0.0574	1	0.03151	
rs1450235	4	144923530	1.288	2.924	0.003461	T	C	22/688/6570	0.09451	0.09549	0.3883	0.05291	
rs1822841	4	145062088	1.289	2.92	0.0035	G	T	22/683/6575	0.09382	0.09488	0.3227	0.05276	
rs3856989	4	145062355	1.29	2.933	0.003362	T	C	22/682/6576	0.09368	0.09475	0.3216	0.05251	
rs7693772	4	145106217	1.282	2.838	0.004541	C	G	29/629/6619	0.08644	0.08995	0.002351	0.04984	
rs4585249	4	145121776	1.285	2.849	0.004389	G	A	27/632/6619	0.08684	0.08981	0.008327	0.04979	
rs4305468	4	145127918	1.291	2.872	0.004078	G	C	19/653/6608	0.0897	0.09041	0.515	0.05016	
rs11731381	4	154201274	1.279	3.032	0.002427	A	G	36/778/6466	0.1069	0.1099	0.02428	0.06024	
rs17033402	4	156859787	0.8343	-3.207	0.001342	G	A	237/2186/4857	0.3003	0.2986	0.666	0.1782	GUCY1A3
rs4691073	4	156891338	0.8414	-3.391	0.0006965	A	G	401/2623/4221	0.362	0.361	0.8199	0.2313	GUCY1B3
rs7654841	4	158270785	0.5196	-2.856	0.004285	T	C	0/211/7069	0.02898	0.02856	0.4083	0.01335	GLRB
rs17357485	4	161736564	0.4408	-3.093	0.001979	A	G	0/184/7096	0.02527	0.02496	0.6323	0.01148	
rs7675062	4	167165316	0.8848	-2.859	0.00425	A	G	1200/3528/2535	0.4857	0.4831	0.662	0.4036	TLL1
rs13122019	4	169894890	0.8796	-2.894	0.003804	A	G	894/3356/3030	0.461	0.457	0.4571	0.3491	PALLD
rs7676725	4	169897266	0.88	-2.883	0.003935	G	A	892/3355/3030	0.461	0.4568	0.4415	0.3489	PALLD
rs2320079	4	169899705	0.88	-2.883	0.003935	T	C	892/3355/3030	0.461	0.4568	0.4415	0.3488	PALLD
rs6811238	4	169900190	0.8797	-2.891	0.003846	T	G	897/3369/3011	0.463	0.4578	0.3435	0.3504	PALLD
rs955911	4	169903754	0.8797	-2.891	0.003846	T	G	897/3369/3011	0.463	0.4578	0.3435	0.3503	PALLD
rs6857155	4	169904341	0.8797	-2.891	0.003846	C	T	897/3369/3011	0.463	0.4578	0.3435	0.3504	PALLD
rs1392756	4	169905396	0.8797	-2.891	0.003846	A	G	897/3369/3011	0.463	0.4578	0.3435	0.3504	PALLD
rs11132458	4	169905936	0.88	-2.883	0.003935	C	A	892/3355/3030	0.461	0.4568	0.4415	0.3488	PALLD
rs9654305	4	169906466	0.8797	-2.891	0.003846	C	T	897/3369/3011	0.463	0.4578	0.3435	0.3505	PALLD
rs6854697	4	169909064	0.8793	-2.901	0.003718	C	A	899/3370/3011	0.4629	0.4579	0.357	0.3506	PALLD
rs4438725	4	171099752	1.167	3.042	0.002351	T	C	328/2201/4201	0.327	0.3344	0.074	0.2163	
rs4692771	4	171103916	1.167	3.042	0.002351	T	C	328/2201/4201	0.327	0.3344	0.074	0.2163	
rs17711139	4	171105519	1.167	3.042	0.002351	G	T	328/2201/4201	0.327	0.3344	0.074	0.2163	
rs11726547	4	171107412	1.142	2.824	0.00475	G	C	441/2664/4175	0.3659	0.3685	0.5669	0.2475	
rs10008105	4	171108801	1.149	2.932	0.003366	T	C	433/2646/4201	0.3635	0.3661	0.543	0.2453	
rs1522605	4	171346678	1.285	3.036	0.002394	A	G	35/709/6536	0.09739	0.1013	0.002356	0.05609	
rs1717096	4	171354173	1.285	2.869	0.004121	A	C	22/671/6570	0.09239	0.0936	0.2585	0.05143	
rs1717097	4	171354544	1.285	2.869	0.004121	T	A	22/671/6570	0.09239	0.0936	0.2585	0.05143	
rs1718803	4	171354857	1.285	2.869	0.004121	G	A	22/671/6570	0.09239	0.0936	0.2585	0.05143	
rs1717098	4	171356751	1.285	2.869	0.004121	T	G	22/671/6570	0.09239	0.0936	0.2585	0.05143	
rs1718802	4	171357908	1.269	2.835	0.004581	T	A	25/739/6516	0.1015	0.1025	0.4219	0.05671	
rs6853808	4	171359497	1.285	2.869	0.004121	C	G	22/671/6570	0.09239	0.0936	0.2585	0.05143	
rs1581421	4	171360691	1.285	2.869	0.004121	A	C	22/671/6570	0.09239	0.0936	0.2585	0.05143	
rs1403231	4	171363908	1.285	2.869	0.004121	C	G	22/671/6570	0.09239	0.0936	0.2585	0.05143	
rs1718800	4	171364331	1.285	2.869	0.004121	C	T	22/671/6570	0.09239	0.0936	0.2585	0.05144	
rs4490423	4	171365420	1.285	2.869	0.004121	A	G	22/671/6570	0.09239	0.0936	0.2585	0.05143	

rs2090958	4	171373018	1.285	2.869	0.004121	T	C	22/671/6570	0.09239	0.0936	0.2585	0.05143	
rs9790679	4	171373410	1.285	2.869	0.004121	C	T	22/671/6570	0.09239	0.0936	0.2585	0.05143	
rs1357327	4	171374271	1.285	2.869	0.004121	T	C	22/671/6570	0.09239	0.0936	0.2585	0.05143	
rs1403226	4	171375505	1.285	2.869	0.004121	G	C	22/671/6570	0.09239	0.0936	0.2585	0.05143	
rs1403227	4	171375613	1.285	2.869	0.004121	A	G	22/671/6570	0.09239	0.0936	0.2585	0.05144	
rs6825128	4	171376342	1.285	2.869	0.004121	A	G	22/671/6570	0.09239	0.0936	0.2585	0.05144	
rs6831236	4	171376697	1.285	2.869	0.004121	T	C	22/671/6570	0.09239	0.0936	0.2585	0.05143	
rs7677730	4	171381604	1.286	2.872	0.004078	C	T	22/671/6567	0.09242	0.09364	0.2587	0.05145	
rs6553505	4	171381880	1.286	2.872	0.004078	G	A	22/671/6567	0.09242	0.09364	0.2587	0.05145	
rs7696514	4	171384920	1.286	2.872	0.004078	G	A	22/671/6567	0.09242	0.09364	0.2587	0.05145	
rs1718789	4	171385012	1.286	2.872	0.004078	G	T	22/671/6567	0.09242	0.09364	0.2587	0.05145	
rs7668436	4	171385199	1.286	2.872	0.004078	C	A	22/671/6567	0.09242	0.09364	0.2587	0.05145	
rs7693573	4	176415278	1.169	3.765	0.0001668	A	G	1444/3500/2279	0.4846	0.4933	0.1329	0.4484	
rs4696018	4	176424237	1.145	3.221	0.001277	A	G	1588/3696/1995	0.5078	0.4984	0.1151	0.4777	
rs1462505	4	179239055	0.7033	-3.215	0.001305	C	T	19/688/6551	0.09479	0.09502	0.8045	0.04769	
rs17069773	4	181717703	0.787	-2.955	0.003122	C	T	55/1132/6093	0.1555	0.1561	0.7636	0.08274	
rs7669125	4	181735182	0.7421	-2.899	0.003743	C	T	24/738/6345	0.1038	0.1045	0.5703	0.05342	
rs6851295	4	181736824	0.7421	-2.899	0.003743	C	G	24/738/6345	0.1038	0.1045	0.5703	0.05342	
rs17283383	4	182964476	1.206	2.967	0.00301	A	C	98/1387/5789	0.1907	0.1939	0.1471	0.1109	
rs2597125	4	183233787	0.8746	-2.887	0.003889	G	T	637/3034/3608	0.4168	0.4167	1	0.2917	
rs7696796	4	183403463	0.7708	-2.979	0.002888	A	G	49/967/5843	0.141	0.1432	0.2052	0.07504	
rs7656126	4	183403974	0.7708	-2.979	0.002888	T	G	49/967/5843	0.141	0.1432	0.2052	0.07504	
rs10021308	4	186953978	0.879	-2.896	0.00378	A	G	878/3255/3137	0.4477	0.4517	0.4515	0.3395	SORBS2
rs10004789	4	191167888	1.13	2.839	0.004528	G	A	919/3417/2943	0.4694	0.4613	0.1406	0.3664	
rs7722355	5	2196119	1.204	2.921	0.003493	A	G	90/1357/5820	0.1867	0.1891	0.2913	0.109	
rs10039198	5	3255947	0.8548	-3.184	0.001453	T	G	479/2951/3586	0.4206	0.4019	9.90E-05	0.2734	
rs433274	5	3256444	0.8548	-3.184	0.001453	T	C	479/2951/3586	0.4206	0.4019	9.90E-05	0.2734	
rs1234961	5	3256892	0.8548	-3.184	0.001453	T	C	479/2951/3586	0.4206	0.4019	9.90E-05	0.2734	
rs1236962	5	3256973	0.8652	-3.078	0.002082	G	A	611/2965/3586	0.414	0.4137	0.9772	0.2869	
rs1215697	5	3257163	0.8548	-3.184	0.001453	G	A	479/2951/3586	0.4206	0.4019	9.90E-05	0.2734	
rs1215698	5	3257334	0.866	-3.084	0.002039	C	G	628/3026/3586	0.418	0.4165	0.7995	0.2903	
rs7723199	5	3414181	1.126	2.825	0.004727	G	A	1340/3619/2319	0.4973	0.491	0.2826	0.4377	
rs11749036	5	6178330	0.6704	-3.954	7.70E-05	T	G	21/850/6409	0.1168	0.115	0.2215	0.05844	
rs371869	5	6740557	1.232	2.974	0.002939	A	G	61/1088/6129	0.1495	0.1524	0.1058	0.08583	
rs27842	5	9711904	0.8719	-2.928	0.003412	C	T	619/2974/3676	0.4091	0.4116	0.6081	0.2861	
rs7734914	5	10085335	1.128	2.824	0.004742	A	G	972/3396/2911	0.4665	0.4645	0.7239	0.3721	
rs41502749	5	14587618	1.327	2.869	0.004112	C	T	10/535/6735	0.07349	0.07333	1	0.04015	
rs10073096	5	15652713	1.14	3.166	0.001547	G	C	1594/3587/2094	0.4931	0.4976	0.4369	0.4709	FBXL7
rs1835128	5	15675075	0.8818	-2.984	0.002846	G	C	1483/3626/2170	0.4981	0.4955	0.6703	0.4476	FBXL7
rs17703964	5	15703140	0.884	-2.909	0.00362	A	G	1394/3631/2243	0.4996	0.4932	0.274	0.4368	FBXL7

rs17659916	5	17928445	0.8576	-2.886	0.003908	G	A	320/2462/4475	0.3393	0.3361	0.4423	0.2096	
rs17710915	5	17929313	0.8579	-2.865	0.004168	T	C	315/2432/4475	0.3367	0.3341	0.5261	0.2077	
rs10520853	5	17947976	0.8579	-2.865	0.004168	C	T	315/2432/4475	0.3367	0.3341	0.5261	0.2079	
rs17661265	5	18251688	1.149	3.043	0.002339	C	T	525/2811/3928	0.387	0.3903	0.4707	0.2697	
rs979297	5	21162664	0.7782	-2.815	0.004871	A	G	39/940/6263	0.1298	0.1307	0.5295	0.06797	
rs4552670	5	21236292	0.7689	-2.897	0.003762	C	A	41/906/6290	0.1252	0.1272	0.1946	0.06589	
rs4866265	5	21242341	0.782	-2.81	0.00496	T	G	41/969/6270	0.1331	0.1339	0.599	0.06981	
rs1396166	5	25268721	1.133	2.818	0.004838	T	C	727/3225/3328	0.443	0.4362	0.188	0.3255	
rs1604096	5	26638617	0.7677	-3.025	0.002484	G	A	32/1061/6166	0.1462	0.143	0.05878	0.07453	
rs7733099	5	26640572	0.7805	-2.862	0.004212	A	T	32/1067/6166	0.1469	0.1436	0.0497	0.07496	
rs6450848	5	31554669	1.49	3.231	0.001233	T	G	0/335/6945	0.04602	0.04496	0.03428	0.02437	RNASEN
rs422672	5	31737903	0.8325	-3.149	0.00164	T	C	214/2006/5060	0.2755	0.2784	0.3767	0.164	
rs7709525	5	33369268	0.5122	-2.98	0.002878	C	T	2/199/7079	0.02734	0.0275	0.6527	0.01282	
rs3849683	5	33477603	0.659	-2.971	0.002967	A	C	6/424/6849	0.05825	0.0581	1	0.02833	TARS
rs7705177	5	33482813	0.6551	-3.035	0.002406	T	C	6/431/6843	0.0592	0.059	1	0.02878	TARS
rs6880616	5	33491319	0.5233	-3.235	0.001218	A	C	1/253/6927	0.03523	0.03488	0.7292	0.01651	TARS
rs3765144	5	33491967	0.5233	-3.235	0.001218	G	A	1/253/6927	0.03523	0.03488	0.7292	0.0165	TARS
rs9686398	5	33499401	0.5233	-3.235	0.001218	C	T	1/253/6927	0.03523	0.03488	0.7292	0.0165	TARS
rs925210	5	33511557	0.5233	-3.235	0.001218	C	T	1/253/6927	0.03523	0.03488	0.7292	0.0165	
rs2939378	5	40078002	0.8608	-2.835	0.004579	G	A	342/2418/4486	0.3337	0.3365	0.485	0.2103	
rs10045484	5	50861674	0.4518	-2.893	0.003817	T	C	0/167/7113	0.02294	0.02268	1	0.01046	
rs2221387	5	53109429	0.8148	-2.967	0.003012	T	A	95/1526/5656	0.2097	0.208	0.5351	0.1141	
rs16881743	5	53116604	0.8192	-2.85	0.004375	G	A	87/1486/5657	0.2055	0.2032	0.3549	0.1112	
rs6450167	5	53129319	0.8623	-2.857	0.004273	T	G	340/2618/4321	0.3597	0.3504	0.02504	0.2216	
rs16881757	5	53147091	0.8628	-2.846	0.004429	G	A	340/2618/4313	0.3601	0.3507	0.02501	0.2219	
rs13436696	5	54233248	1.17	3.035	0.002405	T	C	239/2209/4832	0.3034	0.301	0.508	0.1881	
rs10491367	5	54239231	1.164	2.937	0.003313	T	C	243/2207/4830	0.3032	0.3015	0.6689	0.1885	
rs4865891	5	54269390	1.136	2.878	0.004	G	A	693/3177/3403	0.4368	0.4306	0.2205	0.3184	
rs6895344	5	55607343	0.8773	-2.957	0.003107	G	A	894/3253/3133	0.4468	0.4527	0.2769	0.3429	
rs1997522	5	55687334	1.187	3.017	0.002549	T	A	161/1773/5346	0.2435	0.2464	0.3181	0.1464	
rs6892034	5	55689646	1.193	3.11	0.001869	G	A	160/1774/5346	0.2437	0.2463	0.3659	0.1464	
rs12517669	5	55689902	1.191	3.1	0.001932	G	A	168/1772/5340	0.2434	0.2476	0.1425	0.1474	
rs17416494	5	55998913	0.8104	-2.89	0.003854	A	C	90/1316/5874	0.1808	0.1844	0.09815	0.1003	
rs990453	5	56912727	1.38	3.806	0.0001411	A	T	28/703/6257	0.1006	0.1027	0.1011	0.05698	
rs16887120	5	56920661	1.303	3.396	0.0006836	T	C	30/896/6340	0.1233	0.1229	0.8489	0.06814	
rs6868521	5	64129537	0.8851	-2.952	0.003159	G	A	1731/3522/2027	0.4838	0.4992	0.008551	0.4749	SDCCAG10
rs6449760	5	64131926	0.8817	-3.05	0.002286	T	G	1744/3507/2028	0.4818	0.4992	0.002868	0.4756	SDCCAG10
rs1368704	5	64150760	0.8791	-3.119	0.001813	G	T	1790/3528/1962	0.4846	0.4997	0.009889	0.483	SDCCAG10
rs1433602	5	64175169	0.8793	-3.115	0.00184	G	A	1789/3528/1962	0.4847	0.4997	0.01056	0.483	CWC27
rs6872595	5	64193337	0.8789	-3.125	0.001776	G	A	1791/3531/1958	0.485	0.4997	0.01209	0.4834	SDCCAG10

rs7733752	5	64202638	0.8817	-3.047	0.002309	T	G	1739/3516/2025	0.483	0.4992	0.005598	0.4757	SDCCAG10
rs7700722	5	64224425	1.127	2.868	0.004125	T	C	1781/3524/1854	0.4922	0.4999	0.1936	0.4993	CWC27
rs975081	5	64232425	1.127	2.872	0.004076	A	G	1784/3524/1854	0.492	0.5	0.1856	0.4994	CWC27
rs10940034	5	64764831	0.7914	-2.866	0.004152	T	C	47/1126/6106	0.1547	0.1536	0.5929	0.08107	ADAMTS6
rs4547878	5	65871632	0.7134	-2.818	0.004837	C	T	23/537/6719	0.07377	0.07689	0.001844	0.03807	FLJ46010
rs2017888	5	71523557	0.6689	-2.93	0.003386	C	T	10/450/6820	0.06181	0.06248	0.345	0.03068	MAP1B
rs4703877	5	71761668	0.6753	-2.838	0.004541	G	A	9/445/6826	0.06113	0.06158	0.4494	0.02995	
rs10043295	5	71778780	0.6641	-2.889	0.00386	G	C	7/433/6840	0.05948	0.05952	0.8442	0.02888	ZNF366
rs10068109	5	71779955	0.6712	-2.834	0.004594	A	G	7/436/6837	0.05989	0.0599	0.8462	0.02911	ZNF366
rs10060299	5	71779998	0.6656	-2.872	0.004073	C	T	7/432/6841	0.05934	0.05939	0.8436	0.02883	ZNF366
rs10942300	5	71781168	0.6659	-2.848	0.004399	C	T	7/427/6842	0.05869	0.05877	0.8408	0.02851	ZNF366
rs10942301	5	71781213	0.6659	-2.848	0.004399	G	A	7/427/6842	0.05869	0.05877	0.8408	0.02851	ZNF366
rs12517661	5	71781472	0.6659	-2.848	0.004399	C	T	7/427/6842	0.05869	0.05877	0.8408	0.02851	ZNF366
rs4703886	5	71781741	0.6659	-2.848	0.004399	C	T	7/427/6842	0.05869	0.05877	0.8408	0.02851	ZNF366
rs9293306	5	71782488	0.6659	-2.848	0.004399	A	G	7/427/6842	0.05869	0.05877	0.8408	0.02851	ZNF366
rs9293307	5	71782523	0.6659	-2.848	0.004399	A	C	7/427/6842	0.05869	0.05877	0.8408	0.02851	ZNF366
rs9293308	5	71782538	0.6659	-2.848	0.004399	C	G	7/427/6842	0.05869	0.05877	0.8408	0.02851	ZNF366
rs9293309	5	71782578	0.6659	-2.848	0.004399	A	G	7/427/6842	0.05869	0.05877	0.8408	0.02851	ZNF366
rs12697709	5	71782839	0.6659	-2.848	0.004399	A	G	7/427/6842	0.05869	0.05877	0.8408	0.02851	ZNF366
rs12697710	5	71783001	0.6593	-2.941	0.003268	T	A	7/436/6837	0.05989	0.0599	0.8462	0.02905	ZNF366
rs4703889	5	71783662	0.6659	-2.848	0.004399	G	C	7/427/6842	0.05869	0.05877	0.8408	0.02851	ZNF366
rs2879093	5	71783675	0.6659	-2.848	0.004399	A	G	7/427/6842	0.05869	0.05877	0.8408	0.02851	ZNF366
rs4703890	5	71784380	0.6656	-2.872	0.004073	G	A	7/432/6841	0.05934	0.05939	0.8436	0.02883	ZNF366
rs2338021	5	71784850	0.6656	-2.872	0.004073	T	C	7/432/6841	0.05934	0.05939	0.8436	0.02883	ZNF366
rs3112482	5	71786656	0.6593	-2.941	0.003268	T	C	7/436/6837	0.05989	0.0599	0.8462	0.02906	ZNF366
rs3112483	5	71786660	0.6593	-2.941	0.003268	T	C	7/436/6837	0.05989	0.0599	0.8462	0.02905	ZNF366
rs11741285	5	76436374	1.212	3.033	0.002424	C	T	79/1473/5728	0.2023	0.1989	0.1573	0.1149	LOC728723
rs9293710	5	76486642	0.8364	-2.917	0.003535	T	C	168/1884/5226	0.2589	0.2585	0.9638	0.1491	
rs182042	5	79207143	0.8389	-2.889	0.003867	G	A	162/1942/5172	0.2669	0.2629	0.2118	0.1515	
rs11742720	5	80022904	0.6779	-3.036	0.002401	T	G	10/530/6527	0.075	0.0748	1	0.03662	MSH3
rs10036264	5	80046730	0.6779	-3.036	0.002401	G	C	10/530/6527	0.075	0.0748	1	0.03662	MSH3
rs579492	5	80377212	0.8479	-2.948	0.003197	T	C	253/2185/4842	0.3001	0.3013	0.7264	0.1815	RASGRF2
rs565408	5	80378580	0.8395	-3.151	0.00163	G	A	253/2375/4535	0.3316	0.3213	0.007234	0.1972	RASGRF2
rs516343	5	80379504	0.8386	-3.187	0.001437	C	A	253/2392/4634	0.3286	0.3189	0.009049	0.1953	RASGRF2
rs486683	5	80380953	0.8391	-3.174	0.001502	T	C	252/2392/4634	0.3287	0.3187	0.008073	0.1952	RASGRF2
rs567914	5	80381358	0.8415	-3.067	0.002159	T	C	255/2147/4878	0.2949	0.2984	0.3261	0.1791	RASGRF2
rs4704697	5	80383994	0.8384	-3.13	0.001748	T	C	255/2151/4874	0.2955	0.2987	0.3466	0.1792	RASGRF2
rs580004	5	80384225	0.8477	-2.948	0.003198	G	A	255/2166/4858	0.2976	0.3001	0.482	0.1805	RASGRF2
rs540149	5	80394369	0.8428	-3.007	0.002639	T	A	243/2098/4876	0.2907	0.2939	0.3569	0.1754	RASGRF2
rs2113025	5	82080310	0.8566	-3.293	0.0009897	T	C	658/3079/3543	0.4229	0.4215	0.781	0.297	

rs4383715	5	82213613	1.152	2.887	0.003893	A	G	360/2493/4426	0.3425	0.344	0.7078	0.2246	
rs2656989	5	82226475	1.153	2.902	0.003703	T	C	359/2490/4426	0.3423	0.3437	0.7076	0.2243	
rs9293379	5	83951854	0.8745	-2.819	0.004824	T	C	597/3009/3461	0.4258	0.4179	0.1174	0.2915	
rs11952128	5	91185109	1.135	3.007	0.002637	A	G	1284/3513/2463	0.4839	0.4868	0.6127	0.4242	
rs4367317	5	95586549	0.8662	-2.896	0.003775	T	C	508/2665/3729	0.3861	0.3911	0.2953	0.2629	
rs10213951	5	95594533	0.8672	-2.876	0.004026	C	T	508/2665/3729	0.3861	0.3911	0.2953	0.263	
rs4379224	5	95604991	0.8662	-2.896	0.003775	T	A	508/2665/3729	0.3861	0.3911	0.2953	0.2629	
rs4077816	5	95608250	0.8672	-2.876	0.004026	G	A	508/2665/3729	0.3861	0.3911	0.2953	0.263	
rs7708068	5	95611822	0.8662	-2.896	0.003775	A	G	508/2665/3729	0.3861	0.3911	0.2953	0.2629	
rs7447610	5	95630870	0.8681	-2.843	0.004466	T	C	493/2660/3790	0.3831	0.3873	0.369	0.259	
rs6895341	5	95642851	0.8675	-2.912	0.003589	A	G	511/2772/3983	0.3815	0.3858	0.3459	0.2574	
rs6885381	5	95642994	0.8701	-2.862	0.004215	C	T	515/2782/3983	0.3821	0.3865	0.332	0.2582	
rs6556913	5	95643979	0.8868	-2.851	0.004357	A	G	1774/3617/1843	0.5	0.5	1	0.4909	
rs4502854	5	95644897	0.8675	-2.912	0.003589	A	G	511/2772/3983	0.3815	0.3858	0.3459	0.2574	
rs12518754	5	95646586	0.8675	-2.912	0.003589	T	C	511/2772/3983	0.3815	0.3858	0.3459	0.2574	
rs6881916	5	95646973	0.8675	-2.912	0.003589	T	G	511/2772/3983	0.3815	0.3858	0.3459	0.2574	
rs6868724	5	95647481	0.8889	-2.809	0.004975	C	T	1805/3636/1839	0.4995	0.5	0.9253	0.4934	
rs7733672	5	95649000	0.8868	-2.851	0.004357	T	C	1774/3617/1843	0.5	0.5	1	0.4909	
rs12522310	5	95651304	0.8868	-2.851	0.004357	T	C	1774/3617/1843	0.5	0.5	1	0.4909	
rs10476666	5	95651999	0.8868	-2.851	0.004357	G	A	1774/3617/1843	0.5	0.5	1	0.4909	
rs4869279	5	95654674	0.8675	-2.912	0.003589	A	G	511/2772/3983	0.3815	0.3858	0.3459	0.2574	
rs11950062	5	95656898	0.8868	-2.851	0.004357	A	G	1774/3617/1843	0.5	0.5	1	0.4909	
rs10043904	5	95673735	0.8675	-2.912	0.003589	C	G	511/2772/3983	0.3815	0.3858	0.3459	0.2573	
rs6899303	5	95676731	0.8863	-2.863	0.004192	A	C	1775/3617/1843	0.4999	0.5	1	0.4909	
rs10070093	5	95678368	0.8675	-2.912	0.003589	T	C	511/2772/3983	0.3815	0.3858	0.3459	0.2574	
rs6893547	5	95691636	0.8669	-2.924	0.003454	A	C	506/2780/3969	0.3832	0.3861	0.5231	0.2579	
rs271913	5	95694553	0.8637	-3.009	0.002617	T	C	514/2796/3970	0.3841	0.3873	0.4678	0.2591	
rs1432810	5	109270027	1.59	2.979	0.002889	C	A	3/182/7073	0.02508	0.02557	0.1206	0.01421	
rs11954836	5	113175733	0.5645	-3.178	0.001483	T	C	9/294/6804	0.04137	0.04294	0.007052	0.0202	
rs1422069	5	114498969	1.143	3.06	0.002213	A	G	785/3291/3203	0.4521	0.4448	0.1706	0.3389	TRIM36
rs9285891	5	119948619	1.221	2.878	0.003999	G	A	53/1158/5704	0.1675	0.1661	0.5621	0.09397	PRR16
rs9327151	5	119953076	1.225	2.915	0.003561	G	A	51/1157/5719	0.167	0.1652	0.4233	0.09346	PRR16
rs6862435	5	119953751	1.252	3.427	0.0006112	T	C	75/1247/5578	0.1807	0.182	0.5518	0.1043	PRR16
rs1037568	5	119954330	1.225	3.16	0.001579	C	A	80/1331/5595	0.19	0.1902	0.9001	0.1091	PRR16
rs2045187	5	119955919	1.158	2.888	0.003874	G	A	275/2251/4753	0.3092	0.3108	0.6782	0.1955	PRR16
rs9285892	5	119956346	1.195	2.86	0.004233	C	T	98/1449/5580	0.2033	0.2042	0.7277	0.1176	PRR16
rs1524563	5	119956528	1.193	2.839	0.004529	G	T	101/1449/5589	0.203	0.2045	0.5242	0.118	PRR16
rs9327153	5	119959673	1.195	2.86	0.004233	G	A	98/1449/5580	0.2033	0.2042	0.7277	0.1176	PRR16
rs9327154	5	119962926	1.203	2.984	0.002846	C	A	101/1433/5602	0.2008	0.2029	0.3816	0.1169	PRR16
rs716815	5	120025308	1.154	2.881	0.003968	A	G	318/2468/4493	0.3391	0.3355	0.3825	0.2166	PRR16

rs17151068	5	123365484	1.181	2.819	0.004813	A	G	128/1616/5536	0.222	0.2241	0.4325	0.1313	
rs245314	5	127192354	1.128	2.921	0.003493	T	C	1619/3576/2084	0.4913	0.498	0.2585	0.4723	
rs1421750	5	127240127	1.123	2.821	0.004791	C	A	1636/3558/2057	0.4907	0.4983	0.1949	0.475	
rs2544780	5	127242062	1.123	2.814	0.004899	T	C	1636/3580/2057	0.4922	0.4983	0.3005	0.475	
rs245237	5	127247204	1.124	2.842	0.00449	A	G	1650/3588/2042	0.4929	0.4986	0.3351	0.4772	
rs151855	5	127250007	1.123	2.814	0.004899	C	A	1636/3580/2057	0.4922	0.4983	0.3005	0.4751	
rs245173	5	127252052	1.123	2.814	0.004899	C	G	1636/3580/2057	0.4922	0.4983	0.3005	0.4751	
rs122734	5	127252221	1.123	2.814	0.004899	T	C	1636/3580/2057	0.4922	0.4983	0.3005	0.475	
rs151853	5	127255083	1.123	2.814	0.004899	G	A	1636/3580/2057	0.4922	0.4983	0.3005	0.4751	
rs245167	5	127257094	1.123	2.814	0.004899	T	C	1636/3580/2057	0.4922	0.4983	0.3005	0.4751	
rs2409033	5	127262473	1.123	2.814	0.004899	T	C	1636/3580/2057	0.4922	0.4983	0.3005	0.4751	
rs12659117	5	132925691	0.8542	-3.085	0.002036	C	G	419/2568/4075	0.3636	0.366	0.5806	0.237	FSTL4
rs10479046	5	132945455	0.8744	-2.885	0.00391	T	C	655/3144/3481	0.4319	0.4247	0.1513	0.3016	FSTL4
rs10078111	5	133211769	0.7396	-3.102	0.001924	A	G	24/836/6420	0.1148	0.1141	0.6804	0.0585	
rs3891191	5	133233125	0.5367	-2.85	0.004375	A	G	1/216/7062	0.02967	0.0295	1	0.01411	
rs7712481	5	136046639	0.8781	-2.929	0.003396	T	C	942/3407/2931	0.468	0.4627	0.3358	0.3593	
rs4374753	5	136049449	0.8791	-2.904	0.003688	A	G	943/3405/2932	0.4677	0.4627	0.3618	0.3626	
rs17099072	5	141960255	0.8231	-2.911	0.003602	T	A	126/1560/5585	0.2146	0.2182	0.162	0.1216	FGF1
rs258754	5	142603303	0.7832	-2.912	0.003588	C	G	42/1065/6173	0.1463	0.1454	0.6865	0.07669	
rs258753	5	142603664	0.7855	-2.877	0.004016	T	C	42/1062/6176	0.1459	0.145	0.6861	0.07652	
rs853176	5	142616649	0.764	-2.941	0.003275	T	C	34/897/6349	0.1232	0.1238	0.7043	0.06405	
rs2963149	5	142705277	0.7948	-2.828	0.004687	T	A	49/1113/6118	0.1529	0.1525	0.9387	0.08884	NR3C1
rs2963154	5	142722730	0.7513	-3.104	0.001908	C	T	34/894/6347	0.1229	0.1235	0.6354	0.0638	NR3C1
rs2918415	5	142725156	0.7367	-3.278	0.001045	C	A	32/890/6347	0.1224	0.1226	0.8484	0.0632	NR3C1
rs6861962	5	142730494	0.7367	-3.278	0.001045	A	C	32/890/6347	0.1224	0.1226	0.8484	0.0632	NR3C1
rs1438732	5	142736459	0.7367	-3.278	0.001045	C	G	32/890/6347	0.1224	0.1226	0.8484	0.0632	NR3C1
rs1866388	5	142739978	0.7367	-3.278	0.001045	G	A	32/890/6347	0.1224	0.1226	0.8484	0.0632	NR3C1
rs9324918	5	142747353	0.7367	-3.278	0.001045	C	T	32/890/6347	0.1224	0.1226	0.8484	0.0632	NR3C1
rs4912921	5	142838964	1.133	2.939	0.003291	C	G	1005/3426/2849	0.4706	0.4679	0.6342	0.3786	
rs12054921	5	142839424	1.133	2.939	0.003291	A	G	1005/3426/2849	0.4706	0.4679	0.6342	0.3786	
rs7723926	5	142839612	1.133	2.939	0.003291	G	T	1005/3426/2849	0.4706	0.4679	0.6342	0.3787	
rs7721458	5	142843433	1.133	2.939	0.003291	T	C	1005/3426/2849	0.4706	0.4679	0.6342	0.3786	
rs7700636	5	142860961	1.133	2.939	0.003291	C	A	1005/3426/2849	0.4706	0.4679	0.6342	0.3787	
rs4912922	5	142861882	1.133	2.939	0.003291	A	G	1005/3426/2849	0.4706	0.4679	0.6342	0.3786	
rs17100362	5	142863491	1.133	2.939	0.003291	C	T	1005/3426/2849	0.4706	0.4679	0.6342	0.3786	
rs11167817	5	142863907	1.133	2.939	0.003291	C	T	1005/3426/2849	0.4706	0.4679	0.6342	0.3786	
rs6887579	5	142867368	1.133	2.939	0.003291	G	C	1005/3426/2849	0.4706	0.4679	0.6342	0.3787	
rs13354365	5	142867502	1.133	2.939	0.003291	C	T	1005/3426/2849	0.4706	0.4679	0.6342	0.3786	
rs6580285	5	142869361	1.133	2.939	0.003291	G	A	1005/3426/2849	0.4706	0.4679	0.6342	0.3786	
rs1422682	5	142870503	1.133	2.939	0.003291	C	T	1005/3426/2849	0.4706	0.4679	0.6342	0.3787	

rs1024998	5	142871862	1.133	2.939	0.003291	G	C	1005/3426/2849	0.4706	0.4679	0.6342	0.3786	
rs13340393	5	146084645	0.8851	-2.815	0.004885	T	G	1043/3422/2812	0.4702	0.4705	0.9801	0.3742	PPP2R2B
rs17721604	5	148169619	1.195	3.172	0.001515	T	C	147/1925/5208	0.2644	0.2584	0.04591	0.157	
rs17114703	5	152881609	1.731	3.013	0.002583	C	G	1/128/7151	0.01758	0.0177	0.4407	0.01001	GRIA1
rs10052189	5	153535357	1.308	3.555	0.0003776	T	C	30/930/6292	0.1282	0.1272	0.579	0.07178	
rs2351228	5	153537783	1.272	3.138	0.0017	A	G	31/910/6294	0.1258	0.1253	0.8511	0.07028	
rs890794	5	153538100	1.272	3.138	0.0017	T	G	31/910/6294	0.1258	0.1253	0.8511	0.07026	
rs4958705	5	153544302	1.265	3.867	0.0001104	T	C	94/1462/5724	0.2008	0.201	0.9535	0.1183	
rs752580	5	153546240	1.306	3.391	0.0006959	C	A	29/841/6294	0.1174	0.1176	0.8407	0.06604	
rs7719182	5	153546572	1.27	3.118	0.001821	C	T	31/910/6339	0.125	0.1246	0.8511	0.06964	
rs6860445	5	153557714	1.301	3.344	0.000826	T	C	29/841/6294	0.1174	0.1176	0.8407	0.06599	GALNT10
rs10515706	5	153558711	1.247	3.608	0.0003088	C	T	92/1460/5728	0.2005	0.2003	1	0.1176	GALNT10
rs1465416	5	153562192	1.275	3.107	0.00189	G	C	29/882/6294	0.1224	0.122	0.8467	0.06829	GALNT10
rs17628118	5	153627454	2.4	2.855	0.004305	G	C	0/40/7231	0.0055	0.00549	1	0.00336	GALNT10
rs17552495	5	153629307	2.4	2.855	0.004305	G	A	0/40/7231	0.0055	0.00549	1	0.00336	GALNT10
rs17552639	5	153631652	2.4	2.855	0.004305	G	A	0/40/7231	0.0055	0.00549	1	0.00336	GALNT10
rs6881721	5	153631760	2.4	2.855	0.004305	G	A	0/40/7231	0.0055	0.00549	1	0.00336	GALNT10
rs6882166	5	153631796	2.4	2.855	0.004305	T	G	0/40/7231	0.0055	0.00549	1	0.00336	GALNT10
rs1516648	5	160262795	1.126	2.809	0.004971	A	G	1171/3471/2540	0.4833	0.4818	0.8066	0.409	
rs17058799	5	160479251	1.162	2.868	0.004128	G	A	232/2168/4863	0.2985	0.2967	0.6352	0.1848	
rs4868962	5	162585754	0.8864	-2.833	0.00461	C	G	1288/3515/2420	0.4866	0.4877	0.847	0.4176	
rs17066996	5	165839745	0.7713	-3.12	0.001807	T	A	54/1098/6128	0.1508	0.1519	0.5367	0.07988	
rs17068505	5	166819940	1.167	2.943	0.003249	G	A	254/2228/4320	0.3276	0.3213	0.1131	0.205	ODZ2
rs17068513	5	166826027	1.152	2.88	0.003972	T	C	356/2524/4237	0.3546	0.3513	0.4378	0.2312	ODZ2
rs17068525	5	166826122	1.157	3.053	0.002265	C	T	398/2659/4223	0.3652	0.362	0.4565	0.2413	
rs12655128	5	166827026	1.158	3.084	0.00204	G	A	401/2641/4236	0.3629	0.3612	0.7211	0.2406	
rs11134467	5	167033739	0.7847	-2.825	0.004731	G	A	47/1008/6222	0.1385	0.14	0.3573	0.07313	ODZ2
rs17732939	5	168124390	1.127	2.839	0.00452	C	T	1195/3517/2567	0.4832	0.4822	0.8841	0.4102	SLIT3
rs264875	5	169007204	1.127	2.811	0.004944	T	C	1232/3528/2422	0.4912	0.4863	0.3958	0.4216	DOCK2
rs264835	5	169013087	1.143	3.003	0.00267	A	G	1232/3372/2059	0.5061	0.4923	0.02353	0.4427	DOCK2
rs2382120	5	175164553	1.233	2.823	0.004763	C	G	44/991/6050	0.1399	0.1407	0.6121	0.07865	CPLX2
rs4868662	5	175950155	1.381	2.978	0.002898	T	C	5/432/6799	0.0597	0.05922	0.689	0.03249	CDHR2
rs11134985	5	175954562	1.381	2.978	0.002898	T	C	5/432/6799	0.0597	0.05922	0.689	0.03249	CDHR2
rs4958945	5	177310254	1.134	2.86	0.004232	G	T	753/3134/3393	0.4305	0.4342	0.4662	0.3244	
rs9502950	6	1435103	0.8787	-2.947	0.00321	G	T	1034/3404/2743	0.474	0.4717	0.6889	0.3775	
rs9378834	6	3912843	0.8791	-2.949	0.003187	T	A	1486/3549/1866	0.5143	0.4985	0.0091	0.4667	-DRB1 HLA-D
rs4416719	6	6109207	1.179	3.067	0.002161	A	T	198/2039/5043	0.2801	0.2785	0.6739	0.1716	F13A1
rs9328348	6	6220931	0.8757	-2.852	0.004349	C	T	665/3052/3521	0.4217	0.4222	0.9334	0.2985	F13A1
rs9502429	6	6229124	0.8702	-3.11	0.001869	T	C	915/3148/3025	0.4441	0.4557	0.03259	0.3466	F13A1
rs760680	6	11400275	1.176	2.979	0.002893	A	G	194/2010/5076	0.2761	0.2751	0.7983	0.1673	

rs17764876	6	11582618	0.8359	-2.829	0.004676	A	G	135/1801/5344	0.2474	0.244	0.2492	0.1385	
rs12195374	6	11696549	0.8052	-2.908	0.003634	G	A	75/1322/5882	0.1816	0.1818	0.9486	0.09775	
rs1409286	6	12125051	0.8673	-3.345	0.0008218	C	G	1373/3548/2345	0.4883	0.4911	0.6328	0.428	HIVEP1
rs4714119	6	12127340	0.8714	-3.229	0.001243	T	A	1355/3577/2348	0.4913	0.4907	0.9239	0.4272	HIVEP1
rs9462377	6	12129525	0.8676	-3.348	0.0008136	T	C	1374/3541/2365	0.4864	0.4907	0.4591	0.4269	HIVEP1
rs17765290	6	12134526	0.8668	-3.366	0.0007638	T	A	1373/3554/2353	0.4882	0.4909	0.6331	0.4277	HIVEP1
rs17697699	6	12135388	0.8547	-3.651	0.0002616	G	A	1231/3505/2543	0.4815	0.4838	0.6983	0.4044	HIVEP1
rs2327506	6	12137410	0.8674	-3.345	0.0008243	C	T	1373/3547/2345	0.4882	0.491	0.6328	0.4281	HIVEP1
rs1570987	6	12146962	0.8612	-3.227	0.001251	A	C	930/3152/2526	0.477	0.4708	0.296	0.3745	HIVEP1
rs557767	6	12331413	0.8747	-2.928	0.003406	T	C	751/3175/3354	0.4361	0.4361	1	0.3164	
rs2180030	6	12331615	0.8763	-2.89	0.00385	G	A	751/3176/3353	0.4363	0.4361	1	0.3166	
rs17592421	6	12331908	0.8757	-2.903	0.003695	A	G	751/3182/3347	0.4371	0.4364	0.9145	0.3169	
rs17592533	6	12333687	0.8763	-2.887	0.003891	G	A	750/3179/3347	0.4369	0.4363	0.9144	0.3168	
rs4714300	6	12334056	0.8763	-2.887	0.003891	C	T	750/3179/3347	0.4369	0.4363	0.9144	0.3169	
rs6458132	6	12334404	0.8744	-2.935	0.003336	G	T	751/3176/3353	0.4363	0.4361	1	0.3165	
rs6458136	6	12335502	0.8744	-2.935	0.003336	C	T	751/3176/3353	0.4363	0.4361	1	0.3166	
rs6458137	6	12335694	0.8744	-2.935	0.003336	G	A	751/3176/3353	0.4363	0.4361	1	0.3166	
rs9349138	6	12336189	0.8744	-2.935	0.003336	G	A	751/3176/3353	0.4363	0.4361	1	0.3166	
rs1028715	6	12338741	0.8697	-2.959	0.003091	C	T	612/3123/3528	0.43	0.4194	0.03345	0.2946	
rs484690	6	12342584	0.8697	-2.959	0.003091	G	A	612/3123/3528	0.43	0.4194	0.03345	0.2946	
rs4490660	6	12343185	0.8697	-2.959	0.003091	C	T	612/3123/3528	0.43	0.4194	0.03345	0.2947	
rs9357318	6	12345980	0.8697	-2.959	0.003091	G	A	612/3123/3528	0.43	0.4194	0.03345	0.2946	
rs553182	6	12346913	0.8697	-2.959	0.003091	G	A	612/3123/3528	0.43	0.4194	0.03345	0.2946	
rs2137873	6	16567620	1.145	3.231	0.001233	T	C	1184/3449/2644	0.474	0.4799	0.2936	0.4045	ATXN1
rs1144695	6	16850744	0.8438	-2.846	0.004433	C	T	168/2025/5087	0.2782	0.2717	0.04711	0.1591	ATXN1
rs9461064	6	24873527	1.554	2.893	0.003811	T	C	2/206/7028	0.02847	0.0286	0.6648	0.01583	
rs2524057	6	31359874	1.168	3.032	0.00243	G	A	265/2226/4789	0.3058	0.3069	0.76	0.1931	
rs2524049	6	31363932	1.171	3.081	0.002064	G	C	264/2220/4786	0.3054	0.3066	0.7307	0.1926	
rs2524044	6	31364732	1.171	3.081	0.002064	G	T	264/2220/4786	0.3054	0.3066	0.7307	0.1929	
rs2524042	6	31365139	1.171	3.081	0.002064	G	C	264/2220/4786	0.3054	0.3066	0.7307	0.1929	
rs2524152	6	31369908	1.171	3.081	0.002064	T	C	264/2220/4786	0.3054	0.3066	0.7307	0.1929	
rs2524136	6	31372741	1.171	3.081	0.002064	A	G	264/2220/4786	0.3054	0.3066	0.7307	0.1929	
rs2524131	6	31373066	1.171	3.081	0.002064	T	A	264/2220/4786	0.3054	0.3066	0.7307	0.1929	
rs2507976	6	31459866	0.8617	-3.278	0.001045	A	C	791/3290/3198	0.452	0.4453	0.2065	0.3278	
rs2523497	6	31484907	0.863	-3.392	0.0006943	C	T	1669/3539/1730	0.5101	0.5	0.09285	0.4881	MICA
rs2072633	6	32027557	1.126	2.852	0.004338	A	G	1516/3554/2189	0.4896	0.4957	0.2975	0.4591	CFB RDBP
rs2071482	6	32924678	1.171	2.823	0.004755	A	C	170/1856/5167	0.258	0.2587	0.8198	0.1556	TAP1
rs12529313	6	32925108	1.167	2.895	0.003793	G	A	209/2102/4969	0.2887	0.2862	0.4863	0.1762	TAP1
rs1937780	6	38782428	1.238	2.849	0.004392	A	G	35/981/6234	0.1353	0.1345	0.6622	0.0747	
rs2183818	6	40860439	1.478	2.999	0.002704	T	C	3/278/6849	0.03899	0.03904	0.7606	0.02155	

rs6458182	6	40862869	1.45	3.396	0.0006838	G	C	4/414/6862	0.05687	0.05629	0.53	0.03096	
rs966888	6	40863696	1.45	3.396	0.0006838	A	G	4/414/6862	0.05687	0.05629	0.53	0.03102	
rs2496655	6	41602345	1.127	2.847	0.004411	A	G	1110/3411/2759	0.4685	0.4743	0.2994	0.3911	
rs2495243	6	41603269	1.139	3.075	0.002105	G	A	1114/3478/2688	0.4777	0.4766	0.8633	0.3966	
rs2495244	6	41603317	1.139	3.084	0.002045	A	G	1112/3477/2691	0.4776	0.4765	0.8633	0.3963	
rs1891455	6	41799577	1.235	2.934	0.003349	A	C	34/1115/6127	0.1532	0.1494	0.0276	0.08408	TFEB
rs9462772	6	42192494	1.147	2.933	0.003356	C	T	466/2759/4055	0.379	0.3785	0.926	0.258	
rs4579355	6	42193203	1.142	2.836	0.004564	T	C	474/2752/4054	0.378	0.3791	0.8047	0.2585	
rs4400215	6	42193991	1.143	2.821	0.004793	C	G	474/2752/3816	0.3908	0.3874	0.4792	0.2672	C6orf132
rs394754	6	42767957	1.151	3.076	0.002098	G	T	522/2875/3882	0.395	0.3935	0.7658	0.2743	
rs423137	6	42768633	1.144	2.931	0.003375	A	G	519/2873/3882	0.395	0.3931	0.6983	0.2737	
rs376389	6	42770552	1.144	2.931	0.003375	A	C	519/2873/3882	0.395	0.3931	0.6983	0.2738	
rs9369380	6	42784489	1.139	2.897	0.003769	T	C	986/3244/2340	0.4938	0.4788	0.01156	0.4034	PRPH2
rs6458302	6	42789233	1.132	2.91	0.003615	T	C	1239/3575/2340	0.4997	0.4882	0.04703	0.4292	PRPH2
rs3846893	6	42791039	1.129	2.855	0.004298	C	T	1237/3574/2343	0.4996	0.488	0.047	0.4286	PRPH2
rs7752711	6	42794980	1.132	2.91	0.003615	C	G	1239/3575/2340	0.4997	0.4882	0.04703	0.4291	PRPH2
rs4714621	6	42799255	1.139	3.089	0.002008	T	C	1240/3606/2433	0.4954	0.4866	0.1232	0.4244	
rs9381214	6	42801937	1.136	3.007	0.002635	T	C	1238/3604/2432	0.4955	0.4865	0.123	0.4241	
rs860741	6	44916413	0.6654	-2.988	0.002805	G	A	10/476/6612	0.06706	0.06744	0.5957	0.03314	SUPT3H
rs2038765	6	45184472	0.6535	-2.91	0.003614	G	A	9/418/6797	0.05786	0.05853	0.3111	0.02854	SUPT3H
rs12198982	6	45185892	0.6513	-2.934	0.003351	A	C	9/419/6797	0.05799	0.05866	0.3128	0.02859	SUPT3H
rs10948201	6	45201472	0.6513	-2.934	0.003351	A	C	9/419/6797	0.05799	0.05866	0.3128	0.02859	SUPT3H
rs12193720	6	45211340	0.6763	-2.864	0.004181	G	A	9/465/6728	0.06457	0.06482	0.7142	0.03177	SUPT3H
rs12209161	6	45212299	0.6763	-2.864	0.004181	A	G	9/465/6728	0.06457	0.06482	0.7142	0.03177	SUPT3H
rs1324536	6	45224239	0.6872	-2.824	0.00475	C	T	9/489/6777	0.06722	0.06726	0.8618	0.03305	SUPT3H
rs220669	6	46953432	0.8638	-3.48	0.0005006	T	C	1698/3561/1912	0.4966	0.4996	0.6196	0.4791	GPR116
rs9463275	6	47057796	0.8443	-3.116	0.001836	G	A	279/2398/4601	0.3295	0.3237	0.1284	0.1989	
rs4714980	6	47062497	0.8291	-3.216	0.0013	A	G	210/2084/4907	0.2894	0.2873	0.5657	0.1701	
rs4714981	6	47062830	0.8291	-3.216	0.0013	T	A	210/2084/4907	0.2894	0.2873	0.5657	0.1701	
rs9463276	6	47062945	0.8307	-3.202	0.001365	A	G	210/2111/4954	0.2902	0.2874	0.4382	0.1701	
rs1226498	6	47065584	0.8763	-2.953	0.003151	A	G	855/3280/3145	0.4505	0.4505	1	0.3382	
rs1226500	6	47069911	0.8778	-2.902	0.003704	A	C	841/3269/3141	0.4508	0.4497	0.8549	0.337	
rs574909	6	49397814	0.8248	-2.917	0.003538	C	T	122/1576/5580	0.2165	0.2188	0.3911	0.1225	
rs9369884	6	49402170	0.822	-2.963	0.00305	T	C	123/1566/5579	0.2155	0.2182	0.2822	0.122	
rs500811	6	49402252	0.822	-2.963	0.00305	T	A	123/1566/5579	0.2155	0.2182	0.2822	0.122	
rs563271	6	49403189	0.822	-2.963	0.00305	T	C	123/1566/5579	0.2155	0.2182	0.2822	0.122	
rs482930	6	49405479	0.8118	-3.146	0.001657	A	C	124/1576/5557	0.2172	0.2198	0.3103	0.1228	
rs474897	6	49405960	0.8118	-3.146	0.001657	C	T	124/1576/5557	0.2172	0.2198	0.3103	0.1228	
rs474021	6	49406029	0.8118	-3.146	0.001657	C	T	124/1576/5557	0.2172	0.2198	0.3103	0.1228	
rs472313	6	49406203	0.822	-2.963	0.00305	C	T	123/1566/5579	0.2155	0.2182	0.2822	0.122	

rs548644	6	49407664	0.822	-2.963	0.00305	C	T	123/1566/5579	0.2155	0.2182	0.2822	0.122
rs556807	6	49410241	0.8272	-2.889	0.003869	A	G	122/1608/5523	0.2217	0.2227	0.6734	0.1246
rs512497	6	49410776	0.8118	-3.146	0.001657	G	A	124/1576/5557	0.2172	0.2198	0.3103	0.1228
rs478550	6	49412130	0.8118	-3.146	0.001657	T	C	124/1576/5557	0.2172	0.2198	0.3103	0.1228
rs475757	6	49412439	0.825	-2.913	0.003581	T	C	122/1576/5582	0.2165	0.2188	0.3628	0.1224
rs562369	6	49412748	0.8118	-3.146	0.001657	C	T	124/1576/5557	0.2172	0.2198	0.3103	0.1228
rs529464	6	49414029	0.8143	-3.092	0.001985	C	A	121/1571/5557	0.2167	0.2188	0.4205	0.1222
rs1924982	6	49414269	0.8207	-2.993	0.002761	G	A	125/1574/5581	0.2162	0.2192	0.2611	0.1226
rs549464	6	49416010	0.8238	-2.935	0.003332	A	G	124/1569/5587	0.2155	0.2184	0.2596	0.1223
rs577530	6	49416200	0.822	-2.963	0.00305	C	T	123/1566/5579	0.2155	0.2182	0.2822	0.122
rs9357610	6	49416272	0.822	-2.963	0.00305	T	C	123/1566/5579	0.2155	0.2182	0.2822	0.122
rs485976	6	49417892	0.8118	-3.146	0.001657	G	C	124/1576/5557	0.2172	0.2198	0.3103	0.1227
rs501835	6	49418281	0.822	-2.963	0.00305	A	G	123/1566/5579	0.2155	0.2182	0.2822	0.122
rs9349485	6	49420226	0.811	-3.837	0.0001247	T	C	298/2314/4662	0.3181	0.32	0.608	0.196
rs532017	6	49420368	0.8204	-2.992	0.002773	T	C	123/1572/5585	0.2159	0.2185	0.3081	0.1223
rs530156	6	49420560	0.822	-2.963	0.00305	G	A	123/1566/5579	0.2155	0.2182	0.2822	0.122
rs16879241	6	49420834	0.8203	-2.997	0.002724	C	T	123/1578/5578	0.2168	0.2192	0.3631	0.1227
rs543305	6	49423303	0.8203	-2.997	0.002724	T	A	123/1578/5578	0.2168	0.2192	0.3631	0.1227
rs575461	6	49424393	0.8203	-2.997	0.002724	T	A	123/1578/5578	0.2168	0.2192	0.3631	0.1227
rs520470	6	49424426	0.8203	-2.997	0.002724	T	G	123/1578/5578	0.2168	0.2192	0.3631	0.1227
rs496864	6	49424700	0.8203	-2.997	0.002724	A	G	123/1578/5578	0.2168	0.2192	0.3631	0.1227
rs556711	6	49426133	0.8143	-3.092	0.001985	T	C	121/1571/5557	0.2167	0.2188	0.4205	0.1222
rs545472	6	49426849	0.8233	-2.984	0.002846	A	G	132/1611/5537	0.2213	0.2244	0.2502	0.1261
rs543805	6	49426984	0.8203	-2.997	0.002724	G	T	123/1578/5578	0.2168	0.2192	0.3631	0.1227
rs9381767	6	49428098	0.8203	-2.997	0.002724	G	A	123/1578/5578	0.2168	0.2192	0.3631	0.1227
rs538016	6	49429189	0.8054	-3.26	0.001113	G	A	124/1576/5505	0.2187	0.2211	0.3649	0.1236
rs568369	6	49430248	0.8172	-3.047	0.002315	G	A	121/1578/5581	0.2168	0.2188	0.422	0.1224
rs9357612	6	49433084	0.8118	-3.146	0.001657	T	C	124/1576/5557	0.2172	0.2198	0.3103	0.1228
rs520599	6	49433861	0.8118	-3.146	0.001657	C	A	124/1576/5557	0.2172	0.2198	0.3103	0.1228
rs471220	6	49434529	0.8118	-3.146	0.001657	T	C	124/1576/5557	0.2172	0.2198	0.3103	0.1228
rs471332	6	49434563	0.8118	-3.146	0.001657	A	C	124/1576/5557	0.2172	0.2198	0.3103	0.1228
rs495303	6	49434850	0.8118	-3.146	0.001657	T	C	124/1576/5557	0.2172	0.2198	0.3103	0.1228
rs483192	6	49435591	0.8118	-3.146	0.001657	C	T	124/1576/5557	0.2172	0.2198	0.3103	0.1228
rs478991	6	49436845	0.8203	-2.997	0.002724	A	C	123/1578/5578	0.2168	0.2192	0.3631	0.1227
rs539427	6	49436933	0.8203	-2.997	0.002724	A	G	123/1578/5578	0.2168	0.2192	0.3631	0.1227
rs476131	6	49437817	0.8233	-2.984	0.002846	A	G	132/1611/5537	0.2213	0.2244	0.2502	0.1259
rs510455	6	49437939	0.8203	-2.997	0.002724	T	C	123/1578/5578	0.2168	0.2192	0.3631	0.1227
rs9381770	6	49438095	0.8203	-2.997	0.002724	G	A	123/1578/5578	0.2168	0.2192	0.3631	0.1227
rs513031	6	49438222	0.8118	-3.146	0.001657	G	A	124/1576/5557	0.2172	0.2198	0.3103	0.1228
rs514138	6	49438376	0.8118	-3.146	0.001657	C	T	124/1576/5557	0.2172	0.2198	0.3103	0.1228

rs539754	6	49438851	0.8118	-3.146	0.001657	C	A	124/1576/5557	0.2172	0.2198	0.3103	0.1228	
rs544263	6	49439359	0.807	-3.247	0.001166	C	A	124/1602/5554	0.2201	0.2218	0.4921	0.1241	
rs568143	6	49439658	0.807	-3.247	0.001166	A	C	124/1602/5554	0.2201	0.2218	0.4921	0.1241	
rs575897	6	49441864	0.8007	-3.358	0.0007862	A	G	124/1601/5503	0.2215	0.2231	0.5272	0.1249	
rs570439	6	49442453	0.8007	-3.358	0.0007862	C	G	124/1601/5503	0.2215	0.2231	0.5272	0.1249	
rs1748796	6	49444536	0.8007	-3.358	0.0007862	A	T	124/1601/5503	0.2215	0.2231	0.5272	0.1249	
rs1748797	6	49444556	0.8007	-3.358	0.0007862	C	T	124/1601/5503	0.2215	0.2231	0.5272	0.1249	
rs500592	6	49444819	0.8007	-3.358	0.0007862	C	A	124/1601/5503	0.2215	0.2231	0.5272	0.1249	
rs484133	6	49444937	0.8034	-3.299	0.0009706	G	A	121/1595/5503	0.2209	0.2221	0.6715	0.1243	
rs565340	6	49445792	0.8007	-3.358	0.0007862	T	C	124/1601/5503	0.2215	0.2231	0.5272	0.1249	
rs557770	6	49446672	0.8007	-3.358	0.0007862	G	A	124/1601/5503	0.2215	0.2231	0.5272	0.1249	
rs9381772	6	49446703	0.8007	-3.358	0.0007862	C	G	124/1601/5503	0.2215	0.2231	0.5272	0.1249	
rs12055724	6	49461821	0.8439	-3.103	0.001913	T	G	293/2222/4755	0.3056	0.3117	0.1055	0.1898	
rs10807409	6	49475831	0.8449	-3.081	0.002066	A	G	292/2222/4757	0.3056	0.3115	0.1137	0.1897	
rs2182011	6	49479846	0.8447	-3.089	0.002005	C	G	293/2226/4761	0.3058	0.3117	0.106	0.1899	
rs9296615	6	49481662	0.8409	-3.152	0.001621	A	T	293/2213/4642	0.3096	0.3149	0.1536	0.1924	
rs9395490	6	49486568	0.8477	-3.025	0.002483	G	A	294/2207/4779	0.3032	0.3102	0.05395	0.1888	
rs9367354	6	49489411	0.8411	-3.148	0.001641	A	G	293/2211/4642	0.3094	0.3148	0.1532	0.1923	
rs9381786	6	49520234	0.8415	-3.141	0.001684	G	T	293/2214/4642	0.3097	0.315	0.1646	0.1925	MUT
rs1327268	6	51280428	1.138	2.861	0.004222	G	C	600/3061/3615	0.4207	0.4141	0.1834	0.2971	
rs9370418	6	55374584	1.164	3.011	0.002608	T	C	271/2331/4675	0.3203	0.3169	0.3746	0.2013	GFRAL GFRAL
rs9404741	6	58638567	1.144	3.099	0.001943	T	C	817/3073/3179	0.4347	0.4442	0.07285	0.3378	
rs9391403	6	58640125	1.144	3.099	0.001943	A	G	817/3073/3179	0.4347	0.4442	0.07285	0.3378	
rs9377813	6	58644486	1.144	3.099	0.001943	T	A	817/3073/3179	0.4347	0.4442	0.07285	0.3379	
rs2398394	6	58650186	1.14	3.008	0.002629	A	C	812/3283/3185	0.451	0.4469	0.4469	0.3416	
rs9404626	6	58651076	1.144	3.099	0.001943	T	G	817/3073/3179	0.4347	0.4442	0.07285	0.3379	
rs7769931	6	58669386	1.137	2.945	0.003225	A	C	822/3283/3164	0.4516	0.4481	0.5129	0.3434	
rs4928329	6	58679994	1.144	3.099	0.001943	T	C	817/3073/3179	0.4347	0.4442	0.07285	0.3379	
rs4928452	6	58689965	1.144	3.099	0.001943	T	A	817/3073/3179	0.4347	0.4442	0.07285	0.3379	
rs9377714	6	58707701	1.144	3.099	0.001943	A	T	817/3073/3179	0.4347	0.4442	0.07285	0.3378	
rs1400600	6	58714934	1.146	3.136	0.001715	A	G	809/3080/3180	0.4357	0.4438	0.1266	0.3371	
rs9391294	6	58717970	1.144	3.099	0.001943	A	G	817/3073/3179	0.4347	0.4442	0.07285	0.3378	
rs4478437	6	58723059	1.144	3.099	0.001943	A	G	817/3073/3179	0.4347	0.4442	0.07285	0.3379	
rs1517230	6	58723487	1.144	3.099	0.001943	A	C	817/3073/3179	0.4347	0.4442	0.07285	0.3378	
rs9391295	6	58735426	1.144	3.099	0.001943	T	C	817/3073/3179	0.4347	0.4442	0.07285	0.3379	
rs9391296	6	58737880	1.144	3.099	0.001943	G	A	817/3073/3179	0.4347	0.4442	0.07285	0.3379	
rs4928463	6	58760396	1.14	2.994	0.00275	C	G	812/3017/3174	0.4308	0.4431	0.02039	0.3361	
rs9377831	6	58761209	1.136	2.933	0.003354	G	A	826/3290/3164	0.4519	0.4484	0.5136	0.3438	
rs9404641	6	58761241	1.142	3.057	0.002237	A	G	820/3246/3202	0.4466	0.4463	0.9581	0.3406	
rs9377722	6	58762701	1.137	2.937	0.003315	T	C	810/3281/3185	0.4509	0.4467	0.4312	0.3411	

rs9377723	6	58765669	1.144	3.099	0.001943	G	A	817/3073/3179	0.4347	0.4442	0.07285	0.3378	
rs6932736	6	58766585	1.144	3.099	0.001943	A	G	817/3073/3179	0.4347	0.4442	0.07285	0.3379	
rs4928464	6	58766872	1.144	3.099	0.001943	G	A	817/3073/3179	0.4347	0.4442	0.07285	0.3379	
rs9404770	6	58768284	1.144	3.099	0.001943	C	A	817/3073/3179	0.4347	0.4442	0.07285	0.3379	
rs6901936	6	58770602	1.144	3.099	0.001943	C	T	817/3073/3179	0.4347	0.4442	0.07285	0.3379	
rs3880512	6	58772329	1.144	3.099	0.001943	G	A	817/3073/3179	0.4347	0.4442	0.07285	0.3379	
rs4108706	6	58778083	1.144	3.099	0.001943	A	G	817/3073/3179	0.4347	0.4442	0.07285	0.3379	
rs4928465	6	58782389	1.139	2.998	0.002721	T	C	837/3255/3188	0.4471	0.4479	0.8959	0.3432	
rs4928466	6	58783908	1.137	2.937	0.003315	C	T	810/3281/3185	0.4509	0.4467	0.4312	0.3413	
rs4928467	6	58784521	1.137	2.937	0.003315	T	C	810/3281/3185	0.4509	0.4467	0.4312	0.3413	
rs9377833	6	58788363	1.137	2.937	0.003315	C	T	810/3281/3185	0.4509	0.4467	0.4312	0.3413	
rs9377834	6	58788893	1.137	2.937	0.003315	T	C	810/3281/3185	0.4509	0.4467	0.4312	0.3413	
rs4039185	6	58789882	1.134	2.897	0.00377	C	G	846/3239/3158	0.4472	0.4491	0.7337	0.3446	
rs4928468	6	58794010	1.137	2.937	0.003315	G	A	810/3281/3185	0.4509	0.4467	0.4312	0.3411	
rs9391300	6	58798050	1.137	2.937	0.003315	A	T	810/3281/3185	0.4509	0.4467	0.4312	0.3412	
rs9363384	6	66291458	1.152	3.001	0.002691	T	A	452/2773/4055	0.3809	0.3775	0.4563	0.2577	EGFL11
rs1929330	6	66303331	1.146	2.845	0.004437	T	C	452/2603/3831	0.378	0.3796	0.727	0.2598	EYS
rs1411850	6	66305145	1.146	2.845	0.004437	C	T	452/2603/3831	0.378	0.3796	0.727	0.2598	EYS
rs1331200	6	66306545	1.146	2.845	0.004437	A	C	452/2603/3831	0.378	0.3796	0.727	0.2598	EYS
rs9363388	6	66308721	1.146	2.845	0.004437	C	T	452/2603/3831	0.378	0.3796	0.727	0.2597	EYS
rs9345648	6	66313144	1.146	2.845	0.004437	A	C	452/2603/3831	0.378	0.3796	0.727	0.2598	EYS
rs1411840	6	66314688	1.146	2.845	0.004437	A	G	452/2603/3831	0.378	0.3796	0.727	0.2597	EYS
rs9351510	6	66340521	1.149	3	0.002702	T	C	496/2832/3952	0.389	0.3873	0.7392	0.2677	EGFL11
rs1929338	6	66345904	1.141	2.817	0.004851	C	T	488/2822/3948	0.3888	0.3864	0.6056	0.2665	EYS
rs11970254	6	67705937	0.8588	-2.819	0.004823	T	G	320/2347/4030	0.3505	0.3466	0.378	0.2197	
rs1852705	6	72932233	0.8232	-3.138	0.001703	G	T	169/1822/5289	0.2503	0.2527	0.4041	0.1449	RIMS1
rs1830335	6	72959509	0.8181	-3.231	0.001235	G	A	169/1824/5287	0.2505	0.2529	0.4307	0.1449	RIMS1
rs2250128	6	72967122	0.8274	-2.938	0.003299	C	T	142/1672/5465	0.2297	0.2326	0.2898	0.1312	RIMS1
rs2254147	6	72977664	0.8244	-3.142	0.00168	C	G	181/1831/5268	0.2515	0.2559	0.1554	0.1472	RIMS1
rs4569931	6	75809165	1.206	2.978	0.002905	A	G	98/1396/5763	0.1924	0.1953	0.2065	0.1129	
rs7752899	6	84344431	0.8793	-2.845	0.004441	T	C	969/2897/2718	0.44	0.4647	1.73E-05	0.3631	SNAP91
rs9444177	6	85203828	1.138	2.865	0.004175	T	C	658/3239/3375	0.4454	0.4302	0.002708	0.3175	
rs6902106	6	89982459	1.283	3.231	0.001232	C	A	26/910/6229	0.127	0.1253	0.2582	0.06988	GABRR1
rs1321354	6	90007608	1.278	3.18	0.001474	A	G	26/914/6222	0.1276	0.1258	0.2587	0.07014	
rs282123	6	90016941	1.341	3.273	0.001066	T	C	17/624/6639	0.08571	0.0863	0.4989	0.04764	
rs9452078	6	93445241	0.6716	-2.916	0.003543	A	T	12/442/6748	0.06137	0.06261	0.1266	0.03061	
rs1341994	6	93456741	0.6966	-2.842	0.004479	T	C	12/498/6756	0.06854	0.06926	0.3929	0.03411	
rs10485382	6	97519974	0.7304	-2.867	0.004141	C	T	21/639/6520	0.089	0.09035	0.1919	0.04612	KLHL32
rs2572109	6	99547090	0.8771	-2.833	0.004611	A	G	641/3040/3599	0.4176	0.4175	1	0.292	
rs6937414	6	106576584	0.8549	-2.824	0.004737	A	C	272/2256/4485	0.3217	0.3196	0.6009	0.1961	

rs484621	6	106841101	1.141	3.156	0.001602	T	C	1608/3689/1983	0.5067	0.4987	0.1729	0.4799	ATG5
rs694433	6	106891244	0.8808	-2.822	0.00477	C	T	828/3286/3166	0.4514	0.4484	0.5832	0.3347	
rs9386625	6	107648780	1.803	3.215	0.001305	G	A	0/136/7144	0.01868	0.01851	1	0.01018	PDSS2
rs579257	6	108641709	1.186	3.194	0.001402	G	T	200/2065/5013	0.2837	0.2813	0.5049	0.1739	SNX3
rs217136	6	108658902	1.186	3.194	0.001402	G	C	200/2065/5013	0.2837	0.2813	0.5049	0.1739	SNX3
rs597731	6	108672887	1.186	3.194	0.001402	T	C	200/2065/5013	0.2837	0.2813	0.5049	0.1739	SNX3
rs635051	6	108678357	1.186	3.194	0.001402	C	T	200/2065/5013	0.2837	0.2813	0.5049	0.1739	SNX3
rs534848	6	108684482	1.186	3.194	0.001402	C	T	200/2065/5013	0.2837	0.2813	0.5049	0.1739	SNX3
rs9398166	6	108686901	1.185	3.179	0.001479	C	A	200/2067/5013	0.2839	0.2815	0.479	0.174	SNX3
rs7773293	6	114070787	1.129	2.844	0.004449	C	T	1406/3651/2151	0.5065	0.4947	0.04299	0.4533	
rs4945963	6	114074325	1.131	2.902	0.003712	G	T	1287/3590/2392	0.4939	0.4884	0.3491	0.4284	
rs9488235	6	114077350	1.13	2.893	0.00381	A	G	1290/3593/2395	0.4937	0.4885	0.3746	0.4285	
rs9374579	6	116275968	0.8723	-3.083	0.00205	A	C	919/3448/2912	0.4737	0.4625	0.04013	0.3578	
rs9400883	6	116412806	0.8775	-3.087	0.002021	G	A	1176/3287/2760	0.4551	0.476	0.000208	0.3852	FRK
rs1850629	6	118200284	1.645	3.176	0.001492	T	C	2/170/7108	0.02335	0.02362	0.2768	0.01349	
rs2516102	6	118209787	1.87	2.813	0.004916	A	G	0/80/7188	0.01101	0.01095	1	0.00651	
rs667994	6	118213730	1.87	2.813	0.004916	A	G	0/80/7188	0.01101	0.01095	1	0.00651	
rs1016083	6	118356188	1.892	3.311	0.000931	G	A	1/107/7137	0.01477	0.01493	0.3359	0.00867	SLC35F1
rs17820580	6	118360868	1.892	3.311	0.000931	A	G	1/107/7137	0.01477	0.01493	0.3359	0.00866	SLC35F1
rs9489288	6	118378098	1.9	3.192	0.001414	T	C	1/95/7165	0.01308	0.01327	0.2759	0.00775	SLC35F1
rs6931818	6	120186547	0.8336	-2.888	0.003882	T	A	148/1809/5297	0.2494	0.2481	0.7049	0.141	
rs4946445	6	120198896	0.8336	-2.888	0.003882	G	A	148/1809/5297	0.2494	0.2481	0.7049	0.1411	
rs4403287	6	120201866	0.835	-2.863	0.004194	C	T	148/1810/5295	0.2496	0.2482	0.6704	0.1412	
rs12530313	6	120202750	0.8309	-2.859	0.004246	T	C	148/1690/4996	0.2473	0.2484	0.6971	0.1414	
rs6917690	6	120210314	0.835	-2.863	0.004194	G	T	148/1810/5295	0.2496	0.2482	0.6704	0.1412	
rs13200922	6	120211115	0.8336	-2.888	0.003882	G	A	148/1809/5297	0.2494	0.2481	0.7049	0.1411	
rs12527104	6	120211810	0.8336	-2.888	0.003882	C	T	148/1809/5297	0.2494	0.2481	0.7049	0.1411	
rs4946447	6	120213981	0.836	-2.869	0.004117	A	G	153/1832/5295	0.2516	0.2506	0.7434	0.1428	
rs4946448	6	120216675	0.8355	-2.874	0.00405	G	T	152/1830/5297	0.2514	0.2502	0.7079	0.1426	
rs2148820	6	120308807	0.8717	-2.853	0.004325	G	A	530/2900/3849	0.3984	0.396	0.6358	0.2665	
rs4945705	6	123060689	1.137	3.102	0.00192	G	A	1598/3620/2061	0.4973	0.498	0.9063	0.4727	PKIB
rs9388119	6	123062311	1.139	3.025	0.002485	A	T	1422/3371/2029	0.4941	0.496	0.7511	0.4602	PKIB
rs17084752	6	123065944	1.146	3.129	0.001752	A	G	1432/3272/1935	0.4928	0.4971	0.4894	0.467	PKIB
rs9401575	6	123068776	1.139	3.132	0.001736	A	G	1588/3630/2061	0.4987	0.4979	0.9063	0.4722	PKIB
rs9375249	6	123710046	0.8334	-2.954	0.003133	A	G	162/1851/5238	0.2553	0.255	0.9633	0.1463	TRDN
rs2060065	6	123710384	0.8314	-3	0.002695	A	C	162/1872/5238	0.2574	0.2564	0.7837	0.1473	TRDN
rs6569421	6	125740012	1.324	3.049	0.002298	A	G	13/623/6405	0.08848	0.08793	0.6852	0.04854	
rs6569422	6	125741530	1.324	3.049	0.002298	A	G	13/623/6405	0.08848	0.08793	0.6852	0.04854	
rs1931004	6	132304007	0.8705	-3.125	0.00178	A	G	908/3398/2974	0.4668	0.4597	0.2024	0.3547	
rs12197573	6	133504378	0.8323	-2.819	0.004813	T	C	139/1617/5198	0.2325	0.2354	0.3083	0.1326	

rs7750279	6	133511207	0.8323	-2.819	0.004813	A	C	139/1617/5198	0.2325	0.2354	0.3083	0.1326	
rs7754486	6	133511278	0.8269	-2.919	0.003509	T	G	139/1628/5209	0.2334	0.2359	0.3612	0.1327	
rs12201315	6	133512201	0.8269	-2.919	0.003509	T	C	139/1628/5209	0.2334	0.2359	0.3612	0.1328	
rs1832392	6	133516366	0.8323	-2.819	0.004813	G	T	139/1617/5198	0.2325	0.2354	0.3083	0.1326	
rs1135205	6	135323488	1.127	2.842	0.004478	C	T	1605/3584/2068	0.4939	0.498	0.4942	0.4729	HBS1L
rs9389248	6	135324349	1.126	2.836	0.004572	T	C	1604/3541/2070	0.4908	0.4979	0.2279	0.4724	HBS1L
rs4475342	6	135325041	1.128	2.879	0.003986	T	C	1606/3580/2068	0.4935	0.498	0.4506	0.4729	HBS1L
rs9399129	6	135326841	1.127	2.842	0.004478	C	A	1605/3584/2068	0.4939	0.498	0.4942	0.4729	HBS1L
rs13199205	6	135328198	1.127	2.842	0.004478	T	C	1605/3584/2068	0.4939	0.498	0.4942	0.4729	HBS1L
rs9376074	6	135333811	1.128	2.887	0.003889	C	T	1606/3578/2076	0.4928	0.4979	0.3832	0.4724	HBS1L
rs4895434	6	135335058	1.127	2.842	0.004478	G	A	1605/3584/2068	0.4939	0.498	0.4942	0.4729	HBS1L
rs4896119	6	135335084	1.128	2.879	0.003986	C	T	1606/3580/2068	0.4935	0.498	0.4506	0.473	HBS1L
rs4895435	6	135335156	1.128	2.879	0.003986	C	A	1606/3580/2068	0.4935	0.498	0.4506	0.473	HBS1L
rs4896120	6	135335205	1.128	2.879	0.003986	C	G	1606/3580/2068	0.4935	0.498	0.4506	0.473	HBS1L
rs4896121	6	135335338	1.128	2.878	0.003999	T	C	1606/3540/2074	0.4903	0.4979	0.1937	0.4725	HBS1L
rs7755680	6	135335952	1.128	2.879	0.003986	A	G	1606/3580/2068	0.4935	0.498	0.4506	0.473	HBS1L
rs4895436	6	135336694	1.126	2.836	0.004572	G	A	1604/3541/2070	0.4908	0.4979	0.2279	0.4726	HBS1L
rs4896123	6	135337103	1.128	2.879	0.003986	T	G	1606/3580/2068	0.4935	0.498	0.4506	0.473	HBS1L
rs9376075	6	135338656	1.127	2.842	0.004478	A	G	1605/3584/2068	0.4939	0.498	0.4942	0.4729	HBS1L
rs4637662	6	135339851	1.131	2.929	0.003403	T	C	1602/3575/2069	0.4934	0.4979	0.4363	0.4726	HBS1L
rs13218642	6	135351633	1.129	2.889	0.003864	A	G	1600/3578/2061	0.4943	0.498	0.524	0.4729	HBS1L
rs4451151	6	135352239	1.128	2.879	0.003986	C	T	1606/3580/2068	0.4935	0.498	0.4506	0.473	HBS1L
rs6902954	6	135355687	1.135	3.017	0.002555	C	A	1598/3580/2066	0.4942	0.4979	0.5241	0.4728	HBS1L
rs6909975	6	135357263	1.127	2.842	0.004478	C	T	1605/3584/2068	0.4939	0.498	0.4942	0.4729	HBS1L
rs4895438	6	135359358	1.127	2.842	0.004478	G	T	1605/3584/2068	0.4939	0.498	0.4942	0.4729	HBS1L
rs7773126	6	135360083	1.135	3.017	0.002555	A	G	1598/3580/2066	0.4942	0.4979	0.5241	0.4728	HBS1L
rs4895439	6	135360547	1.128	2.878	0.003999	A	T	1606/3540/2074	0.4903	0.4979	0.1937	0.4725	HBS1L
rs12526055	6	135367611	1.128	2.879	0.003986	C	T	1606/3580/2068	0.4935	0.498	0.4506	0.473	HBS1L
rs6929661	6	135370485	1.131	2.929	0.003403	G	A	1602/3575/2069	0.4934	0.4979	0.4363	0.4726	HBS1L
rs2327578	6	135371662	1.127	2.842	0.004478	C	T	1605/3584/2068	0.4939	0.498	0.4942	0.4729	HBS1L
rs9389256	6	135372947	1.135	3.017	0.002555	A	C	1598/3580/2066	0.4942	0.4979	0.5241	0.4728	HBS1L
rs1014021	6	135376293	1.13	2.928	0.003413	A	G	1611/3587/2081	0.4928	0.4979	0.3838	0.4726	HBS1L
rs6923765	6	135376869	1.13	2.915	0.003552	G	C	1611/3594/2073	0.4938	0.498	0.4801	0.4731	HBS1L
rs6923827	6	135377075	1.135	3.018	0.002546	C	T	1598/3576/2066	0.4939	0.4979	0.4937	0.4728	HBS1L
rs6902438	6	135378280	1.129	2.892	0.003823	C	T	1605/3577/2069	0.4933	0.498	0.4364	0.4728	HBS1L
rs6908681	6	135379426	1.135	3.018	0.002546	A	G	1598/3576/2066	0.4939	0.4979	0.4937	0.4728	HBS1L
rs3756799	6	135382704	1.128	2.879	0.003986	A	G	1606/3536/2074	0.49	0.4979	0.1778	0.4725	HBS1L
rs4896125	6	135384262	1.131	2.93	0.003392	C	T	1602/3571/2069	0.4931	0.4979	0.4089	0.4725	HBS1L
rs949547	6	135385192	1.129	2.892	0.003823	C	G	1605/3577/2069	0.4933	0.498	0.4364	0.4728	HBS1L
rs9376082	6	135388082	1.135	3.018	0.002546	T	A	1598/3576/2066	0.4939	0.4979	0.4937	0.4728	HBS1L

rs11756988	6	135389288	1.135	3.018	0.002546	A	G	1598/3576/2066	0.4939	0.4979	0.4937	0.4728	HBS1L
rs12661423	6	135391263	1.13	2.921	0.003492	C	T	1611/3593/2076	0.4935	0.498	0.4514	0.4729	HBS1L
rs987690	6	135392072	1.131	2.93	0.003392	A	G	1602/3571/2069	0.4931	0.4979	0.4089	0.4725	HBS1L
rs7750300	6	135392643	1.14	3.135	0.001718	C	G	1603/3603/2073	0.495	0.4979	0.6211	0.473	HBS1L
rs6569988	6	135393996	1.127	2.846	0.004432	C	A	1605/3537/2068	0.4906	0.4979	0.21	0.4728	HBS1L
rs6919862	6	135394136	1.127	2.846	0.004432	C	A	1605/3537/2068	0.4906	0.4979	0.21	0.4727	HBS1L
rs10872427	6	135395145	1.127	2.846	0.004432	T	C	1605/3537/2068	0.4906	0.4979	0.21	0.4727	HBS1L
rs7766189	6	135395544	1.127	2.846	0.004432	A	C	1605/3537/2068	0.4906	0.4979	0.21	0.4727	HBS1L
rs4134030	6	135395787	1.127	2.846	0.004432	G	T	1605/3537/2068	0.4906	0.4979	0.21	0.4727	HBS1L
rs4896130	6	135396811	1.128	2.888	0.003876	C	T	1606/3574/2076	0.4926	0.4979	0.3579	0.4724	HBS1L
rs11754265	6	135397909	1.13	2.918	0.003525	G	C	1612/3592/2074	0.4935	0.498	0.4513	0.4731	HBS1L
rs7742542	6	135400260	1.129	2.892	0.003823	T	G	1605/3577/2069	0.4933	0.498	0.4364	0.4728	HBS1L
rs6569990	6	135407511	1.128	2.888	0.003876	C	T	1606/3574/2076	0.4926	0.4979	0.3579	0.4724	HBS1L
rs9399135	6	135410007	1.127	2.844	0.004459	G	A	1605/3579/2068	0.4935	0.498	0.4505	0.4729	HBS1L
rs6915770	6	135410907	1.129	2.892	0.003823	G	A	1605/3577/2069	0.4933	0.498	0.4364	0.4728	HBS1L
rs11755229	6	135411606	1.129	2.892	0.003823	A	G	1605/3577/2069	0.4933	0.498	0.4364	0.4728	HBS1L
rs9402677	6	135412401	1.129	2.892	0.003823	A	T	1605/3577/2069	0.4933	0.498	0.4364	0.4729	HBS1L
rs9389261	6	135412841	1.127	2.846	0.004432	C	G	1605/3537/2068	0.4906	0.4979	0.21	0.4727	HBS1L
rs9376085	6	135412917	1.127	2.846	0.004432	T	C	1605/3537/2068	0.4906	0.4979	0.21	0.4727	HBS1L
rs9376086	6	135413022	1.127	2.846	0.004432	T	C	1605/3537/2068	0.4906	0.4979	0.21	0.4727	HBS1L
rs1041480	6	135414769	1.129	2.892	0.003823	G	T	1605/3577/2069	0.4933	0.498	0.4364	0.4728	HBS1L
rs9373122	6	135415227	1.129	2.892	0.003823	G	A	1605/3577/2069	0.4933	0.498	0.4364	0.4729	HBS1L
rs7741515	6	135416061	1.13	2.917	0.003539	C	G	1611/3594/2075	0.4937	0.498	0.4656	0.473	HBS1L
rs2150681	6	135416925	1.13	2.915	0.003554	C	T	1610/3597/2073	0.4941	0.498	0.5099	0.4731	HBS1L
rs2297338	6	135417455	1.129	2.892	0.003823	G	A	1605/3577/2069	0.4933	0.498	0.4364	0.4728	HBS1L
rs2297339	6	135417684	1.127	2.846	0.004432	G	A	1605/3537/2068	0.4906	0.4979	0.21	0.4727	HBS1L
rs2183709	6	135418784	1.127	2.846	0.004432	C	T	1605/3537/2068	0.4906	0.4979	0.21	0.4727	
rs4142299	6	135418879	1.129	2.892	0.003823	G	T	1605/3577/2069	0.4933	0.498	0.4364	0.4728	
rs2327582	6	135425688	1.141	2.997	0.002728	A	G	1327/3537/2068	0.5102	0.4943	0.007519	0.4517	
rs11154829	6	136289621	0.8801	-2.84	0.004507	T	C	801/3150/3327	0.4328	0.4398	0.1826	0.3209	PDE7B
rs1628976	6	138430627	0.7242	-2.995	0.002745	T	C	59/563/6655	0.07737	0.0892	2.28E-19	0.05387	
rs2077836	6	142963754	1.146	3.077	0.002091	C	T	984/3168/2576	0.4709	0.472	0.8565	0.3863	LOC153910
rs263130	6	142969988	1.154	3.202	0.001363	G	A	936/3114/2576	0.47	0.4694	0.9374	0.3813	LOC153910
rs13202642	6	143001883	1.153	3.055	0.00225	C	G	575/2584/3394	0.3943	0.4075	0.009131	0.2903	
rs9496433	6	143005102	1.153	3.055	0.00225	A	T	575/2584/3394	0.3943	0.4075	0.009131	0.2903	
rs6928084	6	143006732	1.153	3.055	0.00225	G	A	575/2584/3394	0.3943	0.4075	0.009131	0.2903	
rs6924380	6	143007373	1.133	2.88	0.003974	C	T	770/3111/3399	0.4273	0.4348	0.1455	0.3238	
rs6902754	6	143007773	1.153	3.055	0.00225	A	G	575/2584/3394	0.3943	0.4075	0.009131	0.2903	
rs7740440	6	143014610	1.158	3.232	0.001231	A	G	745/2916/3026	0.4361	0.4418	0.2929	0.3346	
rs17071978	6	143102133	1.22	3.348	0.0008148	A	C	119/1588/5573	0.2181	0.2194	0.6306	0.1295	

rs9373401	6	144089574	0.8473	-3.158	0.001588	C	T	341/2470/4457	0.3398	0.3396	1	0.2131	PHACTR2
rs9373575	6	149165785	1.165	3.677	0.0002359	G	A	1344/3515/2421	0.4828	0.4891	0.2809	0.4321	UST
rs9403986	6	149166776	0.8756	-3.171	0.00152	G	A	1436/3553/2291	0.488	0.4931	0.3793	0.4357	UST
rs9403990	6	149209898	0.8688	-2.966	0.003022	T	G	595/3025/3660	0.4155	0.4114	0.4087	0.2857	UST
rs9498341	6	149742566	1.312	3.004	0.002666	T	A	13/669/6565	0.09231	0.0913	0.4386	0.04998	TAB2
rs9498342	6	149742635	1.303	2.927	0.003424	G	C	13/670/6566	0.09243	0.0914	0.439	0.04997	TAB2
rs12191079	6	149743162	1.303	2.927	0.003424	T	C	13/670/6566	0.09243	0.0914	0.439	0.04997	TAB2
rs12204461	6	149743228	1.296	2.863	0.0042	T	A	13/670/6566	0.09243	0.0914	0.439	0.04992	TAB2
rs11155647	6	149747328	1.303	2.927	0.003424	C	T	13/670/6566	0.09243	0.0914	0.439	0.04997	TAB2
rs2248861	6	151687262	0.8636	-3.321	0.0008967	T	C	905/3182/3163	0.4389	0.4515	0.01792	0.3387	KAP12 AKAP1
rs3900024	6	151748263	0.8868	-2.849	0.004379	A	G	1386/3513/2381	0.4826	0.4907	0.1588	0.4261	ZBTB2
rs6928370	6	153263843	1.376	2.807	0.004999	G	A	4/401/6801	0.05565	0.05515	0.6657	0.03029	
rs9479432	6	153265706	1.376	2.807	0.004999	T	C	4/401/6801	0.05565	0.05515	0.6657	0.03029	
rs9397778	6	155470624	0.8767	-3.039	0.002374	G	A	1060/3334/2886	0.458	0.4685	0.05412	0.3702	TIAM2
rs41487150	6	157339651	0.5625	-2.847	0.004413	G	A	0/234/7046	0.03214	0.03163	0.2667	0.01504	ARID1B
rs2025641	6	158318990	1.182	3.283	0.001027	G	A	264/2379/4636	0.3268	0.3196	0.0565	0.2027	
rs4263608	6	158319437	1.273	3.895	9.81E-05	A	G	94/1446/5734	0.1988	0.1994	0.7689	0.116	
rs10447366	6	158889165	1.146	3.249	0.001159	A	G	1470/3649/2158	0.5014	0.4955	0.3204	0.4567	TMEM181
rs2293289	6	161471429	1.158	2.849	0.004387	A	G	246/2243/4791	0.3081	0.3051	0.42	0.1914	AGPAT4
rs12525451	6	161977790	1.185	2.959	0.003083	T	A	150/1773/5357	0.2435	0.2442	0.8104	0.1457	PARK2
rs10945761	6	161985357	1.182	2.913	0.003574	C	T	149/1772/5359	0.2434	0.2439	0.8476	0.1453	PARK2
rs6932653	6	163586793	1.125	2.83	0.00466	T	C	1231/3453/2594	0.4744	0.4825	0.1588	0.4114	PACRG
rs10945953	6	164427516	0.6587	-3.355	0.0007937	A	G	18/535/6604	0.07475	0.0766	0.06106	0.03822	
rs12333017	6	164429440	0.6947	-3.185	0.001448	C	A	18/636/6558	0.08819	0.08884	0.5066	0.04463	
rs10484511	6	164435755	0.691	-3.457	0.0005453	A	G	26/712/6542	0.0978	0.09944	0.1569	0.05016	LOC728275
rs10945955	6	164438308	0.7487	-2.886	0.003896	T	C	25/771/6447	0.1064	0.1069	0.6604	0.0545	
rs9456927	6	164443836	0.7487	-2.886	0.003896	C	T	25/771/6447	0.1064	0.1069	0.6604	0.05451	
rs9458985	6	164444839	0.7487	-2.886	0.003896	G	A	25/771/6447	0.1064	0.1069	0.6604	0.0545	
rs13205991	6	164445957	0.7487	-2.886	0.003896	A	G	25/771/6447	0.1064	0.1069	0.6604	0.05451	
rs206701	6	164448085	0.7385	-3.085	0.002038	A	C	28/806/6443	0.1108	0.1114	0.5981	0.05679	LOC728275
rs9347065	6	165377638	1.155	3.452	0.0005566	T	C	1323/3534/2403	0.4868	0.4889	0.7188	0.4313	
rs3008010	6	165930969	0.6316	-2.996	0.002734	G	A	11/371/6872	0.05114	0.05271	0.02151	0.02596	PDE10A
rs2983505	6	165931057	0.6316	-2.996	0.002734	T	C	11/371/6872	0.05114	0.05271	0.02151	0.02596	PDE10A
rs2983508	6	165931203	0.6316	-2.996	0.002734	G	C	11/371/6872	0.05114	0.05271	0.02151	0.02596	PDE10A
rs713084	6	165931419	0.6316	-2.996	0.002734	T	C	11/371/6872	0.05114	0.05271	0.02151	0.02596	PDE10A
rs2983512	6	165932010	0.6316	-2.996	0.002734	T	C	11/371/6872	0.05114	0.05271	0.02151	0.02596	PDE10A
rs308092	7	5850048	0.8864	-2.881	0.003966	T	C	1572/3623/2072	0.4986	0.4976	0.8875	0.4611	ZNF815
rs7812038	7	9614151	0.8661	-3.096	0.001962	T	G	679/3201/3400	0.4397	0.4302	0.06012	0.308	
rs6463932	7	9615649	0.8688	-3.026	0.002479	C	T	677/3228/3375	0.4434	0.4313	0.01806	0.3098	
rs4487658	7	9618385	0.8632	-3.135	0.001718	A	G	651/3137/3400	0.4364	0.4269	0.06025	0.3037	

rs1532852	7	9618398	0.8632	-3.135	0.001718	C	G	651/3137/3400	0.4364	0.4269	0.06025	0.3038	
rs1032873	7	9618805	0.8632	-3.135	0.001718	C	T	651/3137/3400	0.4364	0.4269	0.06025	0.3038	
rs6953287	7	11180765	0.821	-3.454	0.0005514	C	A	236/2154/4890	0.2959	0.2957	0.9684	0.1763	
rs6944741	7	11183909	0.8115	-3.636	0.0002768	C	G	229/2177/4871	0.2992	0.2965	0.4767	0.1765	
rs6460904	7	12244674	1.229	3.057	0.002238	A	G	55/1238/5951	0.1709	0.1688	0.3292	0.09683	
rs11978669	7	13537710	0.7145	-3.357	0.0007868	T	C	37/788/6126	0.1134	0.1163	0.0387	0.05885	
rs1918265	7	16238160	0.878	-2.994	0.002757	C	T	1072/3457/2750	0.4749	0.4734	0.8045	0.3789	
rs17714316	7	17596179	1.456	2.94	0.003281	T	G	2/298/6980	0.04093	0.04062	0.772	0.02228	
rs11761610	7	17683751	1.14	3.058	0.002231	C	G	927/3330/3023	0.4574	0.4586	0.838	0.3603	
rs2075085	7	17686787	1.151	3.286	0.001015	T	C	920/3332/3028	0.4577	0.4581	0.9388	0.3601	
rs4721647	7	17688494	1.14	3.062	0.002201	T	G	958/3351/2971	0.4603	0.4618	0.7996	0.366	
rs12700260	7	21161896	1.15	2.894	0.003799	T	C	480/2808/3368	0.4219	0.4059	0.001362	0.2886	
rs10239005	7	21193234	1.141	2.836	0.004568	A	T	479/2809/3970	0.387	0.3843	0.5618	0.2647	
rs12154561	7	21193868	1.141	2.836	0.004568	G	T	479/2809/3970	0.387	0.3843	0.5618	0.2646	
rs10260685	7	21194963	1.141	2.836	0.004568	G	A	479/2809/3970	0.387	0.3843	0.5618	0.2647	
rs10241111	7	21197853	1.141	2.836	0.004568	A	G	479/2809/3970	0.387	0.3843	0.5618	0.2647	
rs7810780	7	21199399	1.144	2.898	0.003751	C	T	481/2812/3970	0.3872	0.3846	0.583	0.2652	
rs7791933	7	21199562	1.141	2.833	0.004613	G	A	479/2810/3970	0.3871	0.3844	0.5618	0.2647	
rs17150652	7	24978580	0.8325	-3.512	0.0004456	T	C	363/2398/4519	0.3294	0.337	0.05574	0.2091	OSBPL3
rs16873726	7	24993184	0.8314	-3.642	0.0002708	T	C	431/2631/4214	0.3616	0.3648	0.4598	0.2342	
rs17150701	7	24995311	0.8314	-3.642	0.0002708	T	G	431/2631/4214	0.3616	0.3648	0.4598	0.2343	
rs17150775	7	25021867	0.8314	-3.642	0.0002708	A	G	431/2631/4214	0.3616	0.3648	0.4598	0.2343	
rs17150777	7	25022073	0.8369	-3.524	0.0004258	T	C	433/2633/4214	0.3617	0.3651	0.4223	0.2347	
rs12674177	7	28333441	0.8681	-2.862	0.004209	C	G	456/2825/3999	0.388	0.3816	0.1576	0.252	CREB5
rs1014137	7	31590271	1.159	3.557	0.0003757	T	C	1651/3661/1968	0.5029	0.4991	0.526	0.4843	CCDC129
rs12701126	7	31599796	1.162	3.62	0.000294	T	C	1570/3663/2047	0.5032	0.4979	0.3711	0.4731	CCDC129
rs7780772	7	31618335	1.137	2.957	0.003109	C	T	1448/3507/1861	0.5145	0.4982	0.006959	0.475	CCDC129
rs38332	7	31618853	1.137	3.088	0.002014	G	A	1642/3643/1995	0.5004	0.4988	0.7961	0.4806	CCDC129
rs7457833	7	33521559	1.389	3.296	0.000982	G	A	10/493/6777	0.06772	0.06798	0.7283	0.03767	BBS9
rs1451017	7	33567284	0.8541	-2.967	0.003003	A	G	353/2383/4029	0.3523	0.3524	0.9725	0.2255	BBS9
rs1451018	7	33567464	0.8657	-3.267	0.001087	T	C	939/3297/2979	0.457	0.46	0.5735	0.3551	BBS9
rs2329388	7	39349615	0.8493	-3.089	0.00201	C	T	353/2499/4088	0.3601	0.3552	0.2647	0.2261	POU6F2
rs2329390	7	39349848	0.8493	-3.089	0.00201	A	G	353/2499/4088	0.3601	0.3552	0.2647	0.2261	POU6F2
rs2329391	7	39349983	0.8644	-2.946	0.003216	T	C	472/2697/4111	0.3705	0.3751	0.3024	0.2451	POU6F2
rs6962623	7	39351355	0.8478	-3.122	0.001799	T	C	353/2511/4062	0.3625	0.3566	0.1777	0.2274	POU6F2
rs2237411	7	39353331	0.8559	-3.151	0.001628	A	G	481/2717/4082	0.3732	0.3777	0.3206	0.2475	POU6F2
rs1558159	7	39353666	0.8485	-3.108	0.001886	C	A	353/2508/4088	0.3609	0.3556	0.2244	0.2264	POU6F2
rs740509	7	39355472	0.8485	-3.108	0.001886	G	C	353/2508/4088	0.3609	0.3556	0.2244	0.2264	POU6F2
rs2024100	7	39423177	0.8671	-2.987	0.002815	T	C	621/2994/3284	0.434	0.4255	0.1008	0.3012	POU6F2
rs7792157	7	39424149	0.8689	-2.944	0.003238	T	G	620/2995/3285	0.4341	0.4254	0.095	0.301	POU6F2

rs17619647	7	39424239	0.8689	-2.944	0.003238	T	C	620/2995/3285	0.4341	0.4254	0.095	0.3011	POU6F2
rs1203739	7	40715175	1.133	2.994	0.00275	G	A	1517/3543/2220	0.4867	0.4953	0.1361	0.4575	C7orf10
rs4720569	7	47013623	1.135	2.972	0.002955	T	C	1500/3704/2038	0.5115	0.4972	0.01592	0.4677	
rs2881492	7	47029525	1.13	2.888	0.003879	C	T	1504/3713/2041	0.5116	0.4973	0.01504	0.4679	
rs4724529	7	47031133	1.13	2.888	0.003879	C	G	1504/3713/2041	0.5116	0.4973	0.01504	0.4679	
rs10949887	7	63389515	0.7837	-2.837	0.004555	C	T	47/985/6248	0.1353	0.1372	0.2313	0.0716	
rs10949888	7	63404158	0.7807	-2.833	0.004614	G	A	41/966/6268	0.1328	0.1337	0.5395	0.0699	
rs1879873	7	68376008	1.187	3.004	0.002665	C	T	154/1697/5427	0.2332	0.2375	0.1145	0.1424	
rs12669838	7	68386968	1.199	2.973	0.002951	G	T	111/1503/5630	0.2075	0.2098	0.3413	0.1229	
rs10250757	7	68387271	1.201	2.999	0.002709	C	T	111/1503/5648	0.207	0.2093	0.3404	0.1227	
rs10258649	7	68389024	1.206	3.073	0.002121	C	T	111/1504/5648	0.2071	0.2094	0.3408	0.1229	
rs10255614	7	68389086	1.206	3.073	0.002121	C	A	111/1504/5648	0.2071	0.2094	0.3408	0.1229	
rs16869061	7	68390119	1.205	3.064	0.002182	A	T	111/1505/5648	0.2072	0.2095	0.3412	0.1229	
rs10249177	7	68391365	1.205	3.064	0.002182	G	C	111/1505/5648	0.2072	0.2095	0.3412	0.1229	
rs10276060	7	68393490	1.205	3.064	0.002182	G	T	111/1505/5648	0.2072	0.2095	0.3412	0.1229	
rs1574044	7	68394744	1.205	3.064	0.002182	G	A	111/1505/5648	0.2072	0.2095	0.3412	0.1229	
rs12669407	7	68396003	1.205	3.064	0.002182	A	C	111/1505/5648	0.2072	0.2095	0.3412	0.1229	
rs4718891	7	68545197	0.8715	-2.863	0.004197	G	C	525/2819/3935	0.3873	0.3903	0.5089	0.2618	
rs2158867	7	75653077	1.153	3.076	0.002098	C	A	655/3083/3041	0.4548	0.4381	0.001727	0.3293	
rs1860148	7	75844750	1.166	3.43	0.0006045	A	T	604/2976/3683	0.4097	0.4101	0.9316	0.2919	
rs999096	7	75856951	1.167	3.435	0.0005929	T	C	590/2967/3683	0.4098	0.4087	0.8405	0.2904	SRCRB4D
rs7808180	7	76885718	0.8849	-2.848	0.004401	C	A	1126/3417/2737	0.4694	0.4755	0.2675	0.3851	
rs2065198	7	78305153	1.203	3.744	0.0001809	A	C	304/2472/4504	0.3396	0.3336	0.1309	0.2167	MAGI2
rs514951	7	78333273	1.199	3.632	0.0002814	C	T	289/2398/4593	0.3294	0.3252	0.2959	0.2095	MAGI2
rs514025	7	78333403	1.199	3.632	0.0002813	G	A	289/2398/4593	0.3294	0.3252	0.2959	0.2095	MAGI2
rs488478	7	78333849	1.199	3.632	0.0002811	C	T	290/2396/4594	0.3291	0.3252	0.3305	0.2096	MAGI2
rs1799014	7	78363746	1.158	2.884	0.003927	A	G	272/2291/4717	0.3147	0.3136	0.7936	0.1992	MAGI2
rs7811465	7	82934635	1.155	2.962	0.003057	C	T	364/2567/4349	0.3526	0.3502	0.5695	0.2316	SEMA3E
rs2249189	7	82935593	1.134	2.923	0.003465	C	G	876/3251/3153	0.4466	0.4511	0.3913	0.3496	SEMA3E
rs2249188	7	82935623	1.132	2.879	0.003988	A	G	885/3235/3160	0.4444	0.4512	0.203	0.3498	SEMA3E
rs1476443	7	91110480	1.207	2.947	0.003211	T	C	92/1345/5807	0.1857	0.1888	0.1705	0.1092	
rs12672352	7	100900791	0.8109	-4.236	2.28E-05	T	A	512/2796/3952	0.3851	0.3877	0.5653	0.2578	EMID2
rs870966	7	100903877	0.8296	-3.978	6.96E-05	G	A	677/2946/3544	0.4111	0.42	0.07188	0.2951	EMID2
rs28881	7	106051908	1.147	3.278	0.001045	G	A	1427/3591/2261	0.4933	0.4934	1	0.4487	
rs41835	7	106052801	1.144	3.173	0.001508	G	C	1359/3490/2261	0.4909	0.492	0.8471	0.4424	
rs12535046	7	114797859	0.5856	-3.116	0.001831	T	C	7/325/6879	0.04507	0.04591	0.12	0.02227	
rs12535099	7	114798059	0.5856	-3.116	0.001831	G	C	7/325/6879	0.04507	0.04591	0.12	0.02228	
rs12534695	7	114813143	0.5856	-3.116	0.001831	A	C	7/325/6879	0.04507	0.04591	0.12	0.02227	
rs6973066	7	124775795	0.7537	-3.252	0.001147	T	C	47/997/6212	0.1374	0.1391	0.3105	0.07217	
rs10234507	7	124776546	0.7302	-3.414	0.0006412	T	G	34/937/6266	0.1295	0.1292	1	0.06636	

rs10238745	7	124777794	0.7833	-2.84	0.004512	A	C	48/984/6201	0.136	0.1382	0.2009	0.07199	
rs11974366	7	124777895	0.6751	-3.9	9.60E-05	G	C	24/849/6292	0.1185	0.1174	0.4806	0.05929	
rs10500106	7	124779451	0.772	-3.066	0.00217	T	C	49/1045/6186	0.1435	0.1447	0.5164	0.07552	
rs10500108	7	124779737	0.7658	-3.057	0.002232	C	T	46/985/6209	0.136	0.1377	0.3054	0.07148	
rs6971838	7	124781115	0.7739	-3.036	0.002394	G	A	49/1043/6186	0.1433	0.1445	0.4655	0.07545	
rs12706649	7	124781659	0.7658	-3.057	0.002232	A	G	46/985/6209	0.136	0.1377	0.3054	0.07148	
rs12706650	7	124782413	0.7681	-3.117	0.001827	C	T	48/1050/6182	0.1442	0.145	0.6276	0.07563	
rs11981796	7	124784023	0.6642	-3.993	6.53E-05	G	A	20/839/6357	0.1163	0.1144	0.181	0.05767	
rs1896471	7	124785869	0.6663	-3.98	6.88E-05	G	T	21/844/6353	0.1169	0.1152	0.2597	0.05812	
rs1427574	7	124790024	0.66	-4.058	4.95E-05	A	T	21/841/6349	0.1166	0.115	0.2588	0.05796	
rs1593754	7	124795241	0.66	-4.058	4.95E-05	T	C	21/841/6349	0.1166	0.115	0.2588	0.05796	
rs1427572	7	124801579	0.6629	-4.031	5.56E-05	T	C	21/848/6343	0.1176	0.1158	0.2215	0.05839	
rs934028	7	124804219	0.8119	-3.197	0.001389	T	C	110/1862/4851	0.2729	0.2586	2.66E-06	0.1482	
rs934027	7	124804347	0.8166	-3.115	0.001839	G	A	110/1869/4888	0.2722	0.2579	2.69E-06	0.1478	
rs1427571	7	124806315	0.6629	-4.031	5.56E-05	T	G	21/848/6343	0.1176	0.1158	0.2215	0.05839	
rs6943219	7	127095622	0.7951	-3.251	0.001149	G	A	93/1513/5512	0.2126	0.2102	0.3671	0.1165	SND1
rs6943385	7	127095664	0.8025	-3.127	0.001765	G	C	91/1508/5681	0.2071	0.2052	0.4576	0.1134	SND1
rs6979634	7	127111201	0.8054	-3.077	0.002089	A	C	91/1505/5684	0.2067	0.2049	0.4922	0.1132	SND1
rs6953134	7	127137804	0.7928	-3.34	0.0008363	T	C	97/1562/5620	0.2146	0.2121	0.3477	0.1176	SND1
rs7778413	7	127144952	0.7897	-3.322	0.0008936	C	T	90/1509/5319	0.2181	0.2143	0.1605	0.119	SND1
rs7783102	7	127302906	0.7965	-3.149	0.001641	G	A	80/1435/5765	0.1971	0.1951	0.4006	0.1069	SND1
rs3757758	7	127482649	0.7921	-3.304	0.0009546	C	G	90/1521/5666	0.209	0.2064	0.3067	0.1138	SND1
rs322790	7	127536236	0.8095	-3.416	0.0006354	T	A	166/1915/5198	0.2631	0.261	0.5297	0.1507	
rs322740	7	127570634	0.819	-3.398	0.0006796	T	G	211/2077/4992	0.2853	0.2844	0.8047	0.1679	
rs17169137	7	136741634	1.214	2.988	0.002806	C	T	59/1414/5724	0.1965	0.1902	0.004332	0.11	DGKI
rs10235324	7	137341717	1.17	3.197	0.001388	G	T	332/2468/4437	0.341	0.3391	0.6523	0.2207	
rs17169503	7	137362154	1.182	3.29	0.001003	A	G	269/2308/4703	0.317	0.3145	0.5264	0.2	
rs451054	7	138086355	1.131	2.939	0.003298	A	C	1584/3601/2005	0.5008	0.4983	0.6702	0.4753	ATP6V0A4
rs228578	7	144039366	1.172	3.208	0.001335	T	C	309/2365/4605	0.3249	0.3258	0.8012	0.2101	TPK1
rs757797	7	144768973	0.8459	-2.953	0.003144	G	C	234/2193/4853	0.3012	0.2987	0.5047	0.1776	
rs850377	7	144772709	0.8237	-3.48	0.0005023	T	C	276/2202/4802	0.3025	0.3067	0.2362	0.1836	
rs1976891	7	149742558	1.128	2.816	0.004863	C	T	974/3268/3038	0.4489	0.4598	0.04403	0.3615	
rs6978639	7	149754864	1.159	3.466	0.0005274	C	T	1083/3400/2797	0.467	0.4723	0.3457	0.3865	
rs6960838	7	149754931	1.158	3.44	0.0005807	T	A	1084/3397/2799	0.4666	0.4723	0.309	0.3864	
rs4725898	7	149759950	1.163	3.641	0.0002714	T	C	1799/3568/1913	0.4901	0.4999	0.096	0.4974	
rs11770221	7	149762417	1.164	3.647	0.000265	G	A	1803/3569/1908	0.4902	0.4999	0.1008	0.4979	
rs6959816	7	153657371	1.265	2.837	0.004554	T	C	21/803/6456	0.1103	0.1093	0.5201	0.06029	LOC653748
rs10236495	7	154218726	0.8692	-2.882	0.003958	G	A	503/2782/3991	0.3824	0.3851	0.5427	0.2561	DPP6
rs2316527	7	154253390	0.812	-3.511	0.0004466	A	G	192/2063/4779	0.2933	0.2874	0.08885	0.1704	DPP6
rs11243320	7	154263043	0.8462	-3.083	0.002052	G	A	288/2355/4637	0.3235	0.3216	0.6356	0.1981	DPP6

rs12719629	7	154263593	0.8135	-3.689	0.0002248	T	A	257/2244/4779	0.3082	0.3071	0.7893	0.1856	DPP6
rs6597431	7	154274070	0.815	-3.767	0.000165	C	T	308/2330/4642	0.3201	0.3228	0.4678	0.1979	DPP6
rs3807275	7	154280197	0.8648	-2.809	0.004971	A	G	356/2497/4427	0.343	0.3436	0.8646	0.2176	DPP6
rs10254936	7	154290362	1.945	3.022	0.002515	T	C	1/76/7185	0.01047	0.01068	0.1877	0.00623	DPP6
rs10243586	7	154290475	1.945	3.022	0.002515	A	G	1/76/7185	0.01047	0.01068	0.1877	0.00623	DPP6
rs10264311	7	154290904	1.945	3.022	0.002515	G	A	1/76/7185	0.01047	0.01068	0.1877	0.00623	DPP6
rs9719268	7	154729571	0.7838	-3.641	0.0002715	T	G	135/1594/5487	0.2209	0.225	0.129	0.1246	INSIG1
rs10258075	7	154730440	0.8021	-3.369	0.0007556	T	A	138/1646/5483	0.2265	0.2295	0.2609	0.128	INSIG1
rs6459879	7	157904553	0.8356	-3.214	0.001307	G	C	258/2228/4794	0.306	0.3059	1	0.1843	PTPRN2
rs2335167	7	157906709	0.873	-2.844	0.00446	T	G	551/3014/3715	0.414	0.4056	0.07793	0.2785	PTPRN2
rs1377374	7	157908408	0.8394	-3.123	0.001788	T	G	252/2198/4830	0.3019	0.3023	0.9074	0.1814	PTPRN2
rs1476201	7	157959207	0.8488	-3.246	0.001172	A	G	413/2622/4244	0.3602	0.3615	0.7704	0.2317	PTPRN2
rs1670339	7	157963900	0.8661	-2.81	0.004947	A	G	377/2507/4395	0.3444	0.3476	0.438	0.2198	PTPRN2
rs17740671	8	994334	1.658	2.896	0.00378	G	C	2/134/7141	0.01841	0.01878	0.1373	0.01086	
rs10093402	8	1280468	0.8774	-2.947	0.003211	A	C	939/3413/2928	0.4688	0.4627	0.2651	0.3601	
rs13257864	8	1457826	1.137	3.06	0.002215	A	G	1719/3632/1856	0.504	0.4998	0.4943	0.4941	DLGAP2
rs13268384	8	1458328	0.8659	-3.389	0.0007027	C	T	1712/3563/1800	0.5036	0.4999	0.5522	0.4896	DLGAP2
rs7009907	8	1458511	0.8671	-3.356	0.0007907	A	G	1712/3563/1806	0.5032	0.4999	0.601	0.4892	DLGAP2
rs6558478	8	1458717	0.8653	-3.361	0.0007754	C	T	1632/3563/1800	0.5094	0.4997	0.1089	0.4839	DLGAP2
rs6558479	8	1458796	0.8645	-3.468	0.0005236	T	C	1801/3563/1806	0.4969	0.5	0.6034	0.4955	DLGAP2
rs7826386	8	1459557	0.8701	-3.339	0.0008418	C	T	1831/3617/1832	0.4968	0.5	0.5898	0.4959	DLGAP2
rs7012381	8	1462764	1.131	2.952	0.00316	T	C	1743/3655/1882	0.5021	0.4998	0.7076	0.494	DLGAP2
rs2404619	8	1463600	1.137	3.008	0.002633	C	G	1565/3600/1908	0.509	0.4988	0.09056	0.4795	DLGAP2
rs4876085	8	1469115	0.8795	-2.807	0.004997	G	C	1231/3512/1846	0.533	0.4956	9.60E-10	0.45	DLGAP2
rs2272611	8	1864038	1.187	3.275	0.001055	T	C	224/2088/4965	0.2869	0.2878	0.8069	0.179	GEF10 ARHGE
rs1822243	8	3193869	0.8869	-2.852	0.004343	T	C	1742/3682/1856	0.5058	0.4999	0.3248	0.4884	CSMD1
rs2551040	8	3197350	1.144	3.053	0.002264	C	G	1513/3430/1742	0.5131	0.4994	0.02586	0.4875	CSMD1
rs1104305	8	3199596	1.144	3.053	0.002264	A	G	1513/3430/1742	0.5131	0.4994	0.02586	0.4875	CSMD1
rs1096505	8	3200002	1.144	3.053	0.002264	T	C	1513/3430/1742	0.5131	0.4994	0.02586	0.4875	CSMD1
rs10098833	8	3200504	1.144	3.053	0.002264	C	G	1513/3430/1742	0.5131	0.4994	0.02586	0.4875	CSMD1
rs10102119	8	3657789	0.8669	-2.819	0.004824	G	A	398/2609/4272	0.3584	0.3584	1	0.2304	CSMD1
rs12677765	8	4098468	0.8822	-2.977	0.002912	T	C	1679/3551/1901	0.498	0.4995	0.7943	0.4796	CSMD1
rs779110	8	4098532	0.8822	-2.977	0.002912	A	G	1679/3551/1901	0.498	0.4995	0.7943	0.4797	CSMD1
rs6995607	8	4099262	0.8822	-2.977	0.002912	T	A	1679/3551/1901	0.498	0.4995	0.7943	0.4797	CSMD1
rs17069764	8	4100900	0.8808	-3.048	0.0023	G	T	1685/3601/1984	0.4953	0.4992	0.5107	0.4746	CSMD1
rs17063270	8	4432630	1.664	3.468	0.0005241	C	G	1/199/7080	0.02734	0.02723	1	0.01517	CSMD1
rs17070710	8	4436914	1.689	3.342	0.0008323	T	C	0/179/7101	0.02459	0.02429	0.6295	0.01349	CSMD1
rs7845237	8	6118080	0.8877	-2.825	0.004725	G	C	1400/3517/2325	0.4856	0.4918	0.2824	0.4313	
rs9325744	8	15091385	1.25	2.837	0.004548	G	A	30/889/6361	0.1221	0.1219	0.9236	0.0674	SGCZ
rs4831343	8	15506873	0.7899	-2.914	0.003573	C	T	57/1125/6049	0.1556	0.1567	0.548	0.08272	TUSC3

rs2029830	8	18399227	0.885	-2.824	0.004743	T	C	1065/3374/2841	0.4635	0.4702	0.222	0.3737	
rs2632839	8	18718981	1.235	3.093	0.001984	C	T	56/1198/6026	0.1646	0.1638	0.7207	0.09299	PSD3
rs2638608	8	18718994	1.304	2.837	0.004547	T	A	12/603/6665	0.08283	0.08242	0.7766	0.04524	PSD3
rs2638610	8	18719115	1.232	3.034	0.002417	G	A	56/1168/6055	0.1605	0.1604	1	0.09088	PSD3
rs2638611	8	18719176	1.232	3.015	0.002567	C	T	54/1161/6038	0.1601	0.1597	0.8831	0.09037	PSD3
rs6421416	8	20006362	1.158	3.409	0.0006522	A	G	891/3304/3085	0.4538	0.4546	0.8974	0.3552	
rs6421417	8	20008935	1.153	3.321	0.0008963	A	G	912/3305/3063	0.454	0.4563	0.6624	0.3579	
rs6421418	8	20014453	1.156	3.386	0.0007084	G	A	940/3296/3043	0.4528	0.4583	0.3185	0.3613	
rs4278155	8	23052724	1.136	3.06	0.002212	C	A	1608/3638/2032	0.4999	0.4983	0.7959	0.4754	TNFRSF10D
rs4242391	8	23056128	0.7939	-3.154	0.00161	T	C	73/1415/5792	0.1944	0.1914	0.2204	0.1043	TNFRSF10D
rs2941596	8	25198469	0.8583	-3.637	0.0002759	G	A	1714/3695/1870	0.5076	0.4998	0.189	0.4833	DOCK5
rs3739216	8	26683166	1.136	3.091	0.001993	G	C	1818/3567/1895	0.49	0.4999	0.09142	0.4993	DRA1A ADRA1
rs10109311	8	40186623	1.422	3.036	0.002398	G	A	9/345/6921	0.04742	0.04865	0.0467	0.02678	
rs10105368	8	40187793	1.422	3.095	0.001968	T	G	10/359/6905	0.04935	0.05075	0.03198	0.02785	
rs10504019	8	40190982	1.431	3.113	0.001855	A	G	9/350/6921	0.04808	0.04927	0.05124	0.02715	
rs41352947	8	40191717	1.432	3.153	0.001615	A	G	8/363/6909	0.04986	0.05071	0.1592	0.02793	
rs28655819	8	40241991	1.438	3.204	0.001354	A	G	10/359/6911	0.04931	0.05071	0.03188	0.02793	
rs10105630	8	40260199	1.438	3.202	0.001365	A	T	10/359/6909	0.04933	0.05072	0.03191	0.02789	
rs10102605	8	40269752	1.441	3.268	0.001083	A	G	12/362/6906	0.04973	0.05162	0.005004	0.02844	
rs10086031	8	49714525	1.153	2.969	0.002992	C	A	408/2560/4312	0.3516	0.3562	0.2776	0.2366	
rs10098811	8	49714558	1.15	2.917	0.003532	A	G	407/2554/4319	0.3508	0.3556	0.2488	0.236	
rs11994273	8	49716023	1.149	2.892	0.003832	C	T	405/2547/4319	0.3503	0.3551	0.2478	0.2356	
rs13275840	8	49717849	1.152	2.957	0.003108	A	G	407/2560/4313	0.3516	0.3561	0.2922	0.2365	
rs13251674	8	49717935	1.15	2.926	0.00343	A	T	409/2558/4313	0.3514	0.3562	0.2495	0.2366	
rs13248205	8	49718026	1.152	2.963	0.003049	A	G	411/2555/4314	0.351	0.3563	0.1997	0.2367	
rs4873230	8	53971201	0.8746	-3.028	0.002461	T	G	1427/3436/1827	0.5136	0.4982	0.01231	0.4653	
rs4873233	8	53989096	1.134	2.989	0.002803	A	T	1385/3601/2261	0.4969	0.4927	0.4745	0.445	
rs16918308	8	53990175	1.137	3.077	0.002091	G	T	1408/3608/2261	0.4958	0.4931	0.6516	0.4468	
rs4409393	8	57376926	0.8732	-2.945	0.003228	A	G	731/3175/3374	0.4361	0.4341	0.7055	0.3135	RDHE2
rs6983076	8	60787872	1.134	2.991	0.002785	T	C	1154/3458/2666	0.4751	0.4784	0.5566	0.3999	
rs10098842	8	62799895	0.8054	-2.929	0.003405	T	C	99/1212/5663	0.1738	0.1817	0.0004562	0.09865	
rs6990434	8	62806337	0.7814	-3.405	0.0006611	T	C	104/1323/5633	0.1874	0.1933	0.01149	0.1054	
rs7835761	8	62809032	0.7814	-3.405	0.0006611	T	C	104/1323/5633	0.1874	0.1933	0.01149	0.1053	
rs11785234	8	62811648	0.8261	-2.97	0.002977	T	C	120/1741/5418	0.2392	0.2351	0.1483	0.1329	
rs6989668	8	62811838	0.8345	-2.829	0.004669	T	C	128/1711/5439	0.2351	0.2337	0.6519	0.1322	
rs7014115	8	62812121	0.8308	-2.876	0.004033	G	T	117/1732/5422	0.2382	0.2338	0.1198	0.1321	
rs16927824	8	62813554	0.8262	-2.892	0.003824	C	T	116/1609/5537	0.2216	0.2214	1	0.124	
rs10504332	8	62815120	0.8262	-2.892	0.003824	C	T	116/1609/5537	0.2216	0.2214	1	0.124	
rs7015215	8	62816098	0.8308	-2.876	0.004033	T	A	117/1732/5422	0.2382	0.2338	0.1198	0.1321	
rs10096620	8	62816995	0.8191	-2.998	0.002718	A	G	109/1624/5515	0.2241	0.2218	0.427	0.1241	

rs6982384	8	62820477	0.8145	-3.073	0.002119	T	A	109/1616/5523	0.223	0.221	0.4896	0.1234	
rs1904571	8	63929984	1.255	3.355	0.0007946	T	C	45/1272/5963	0.1747	0.1696	0.008544	0.09718	FAM77D
rs10102831	8	63930295	1.256	3.364	0.0007681	A	G	45/1267/5968	0.174	0.169	0.01021	0.09684	FAM77D
rs10504356	8	63935531	1.252	3.309	0.0009353	G	C	45/1266/5969	0.1739	0.1689	0.01022	0.09673	FAM77D
rs10504357	8	63935647	1.261	3.426	0.0006126	G	C	45/1267/5968	0.174	0.169	0.01021	0.09691	FAM77D
rs10504358	8	63936341	1.255	3.353	0.0008007	G	A	46/1266/5968	0.1739	0.1691	0.01518	0.0969	FAM77D
rs10504359	8	63937197	1.249	3.269	0.001081	G	C	45/1262/5963	0.1736	0.1687	0.01216	0.0965	NKAIN3
rs17182014	8	63939640	1.249	3.269	0.001081	G	A	45/1262/5963	0.1736	0.1687	0.01216	0.09651	NKAIN3
rs4272421	8	66315092	1.357	2.906	0.003661	A	G	4/487/6650	0.0682	0.06692	0.1522	0.03669	
rs9886640	8	66464133	0.6014	-3.086	0.002031	A	C	5/348/6926	0.04781	0.04797	0.628	0.02301	
rs920529	8	70643888	1.13	2.945	0.003235	A	T	1612/3623/2045	0.4977	0.4982	0.925	0.4749	SULF1
rs723290	8	70647956	0.8839	-2.971	0.002967	T	C	1798/3649/1832	0.5013	0.5	0.8329	0.4938	SULF1
rs16937518	8	72296384	0.854	-3.047	0.002308	C	A	379/2527/4370	0.3473	0.3496	0.5915	0.2222	EYA1
rs3779755	8	73133558	1.654	3.406	0.0006597	C	T	1/207/7057	0.02849	0.02835	1	0.01593	TRPA1
rs7819749	8	73138355	1.654	3.406	0.0006597	T	G	1/207/7057	0.02849	0.02835	1	0.01593	TRPA1
rs920830	8	73140073	1.673	3.484	0.0004948	A	G	1/206/7051	0.02838	0.02825	1	0.01589	TRPA1
rs10104272	8	73140653	1.673	3.484	0.0004948	G	T	1/206/7051	0.02838	0.02825	1	0.01589	TRPA1
rs2383846	8	73142272	1.654	3.406	0.0006597	G	A	1/207/7057	0.02849	0.02835	1	0.01593	TRPA1
rs1811457	8	73143881	1.636	3.522	0.0004281	A	G	3/222/7024	0.03062	0.03096	0.2644	0.01737	TRPA1
rs7010969	8	73144919	1.673	3.484	0.0004948	A	C	1/206/7051	0.02838	0.02825	1	0.01589	TRPA1
rs13269625	8	73150449	1.673	3.484	0.0004948	G	C	1/206/7051	0.02838	0.02825	1	0.01589	
rs17741463	8	73163435	1.462	2.852	0.004342	A	G	3/273/7004	0.0375	0.03759	0.75	0.02077	
rs17741483	8	73163792	1.504	2.896	0.00378	G	T	0/248/7009	0.03417	0.03359	0.2772	0.01865	
rs10504527	8	73163936	1.503	2.893	0.003818	A	T	0/249/7024	0.03424	0.03365	0.2781	0.01872	
rs11784301	8	73185007	1.695	3.474	0.000513	G	T	2/187/7040	0.02587	0.02607	0.3584	0.01461	
rs7815632	8	73194043	1.56	3.375	0.0007383	T	C	3/264/7013	0.03626	0.0364	0.7398	0.02027	
rs2980221	8	76470294	0.8828	-2.843	0.004462	A	C	1003/3393/2884	0.4661	0.4666	0.92	0.366	
rs3779741	8	76504632	1.173	2.898	0.00375	C	G	206/1713/5305	0.2371	0.2509	6.34E-06	0.1578	
rs1533366	8	76540720	0.8375	-3.202	0.001363	T	G	283/2265/4249	0.3332	0.3298	0.3977	0.2037	
rs2672862	8	76546365	0.8375	-3.202	0.001363	A	C	283/2265/4249	0.3332	0.3298	0.3977	0.2037	
rs2672853	8	76554872	0.8375	-3.202	0.001363	A	T	283/2265/4249	0.3332	0.3298	0.3977	0.2037	
rs830780	8	76564486	0.8375	-3.202	0.001363	G	A	283/2265/4249	0.3332	0.3298	0.3977	0.2037	
rs878088	8	76596138	0.8663	-3.076	0.002096	A	C	615/3041/3621	0.4179	0.4147	0.5157	0.2891	
rs16939134	8	76778407	1.247	2.896	0.003781	T	C	34/940/6234	0.1304	0.1301	0.9278	0.07233	
rs16919400	8	76783549	1.262	3.112	0.001861	A	G	39/958/6281	0.1316	0.1322	0.6582	0.0737	
rs16939153	8	76802912	1.262	3.092	0.001986	G	A	39/944/6267	0.1302	0.131	0.5902	0.07303	
rs2920923	8	76866654	0.8641	-2.817	0.004853	G	A	350/2446/4479	0.3362	0.3389	0.4892	0.2131	
rs2920922	8	76870632	0.8641	-2.817	0.004846	C	T	350/2446/4484	0.336	0.3388	0.489	0.213	
rs2920917	8	76875964	0.8641	-2.817	0.004853	A	C	350/2446/4479	0.3362	0.3389	0.4892	0.2131	
rs2977326	8	76876064	0.8641	-2.817	0.004853	T	C	350/2446/4479	0.3362	0.3389	0.4892	0.2131	

rs2920913	8	76887651	0.8641	-2.817	0.004853	G	T	350/2446/4479	0.3362	0.3389	0.4892	0.2131	
rs2977344	8	76888978	0.8641	-2.817	0.004853	T	C	350/2446/4479	0.3362	0.3389	0.4892	0.2131	
rs2977342	8	76889956	0.8641	-2.817	0.004853	A	G	350/2446/4479	0.3362	0.3389	0.4892	0.2131	
rs7828571	8	76895992	1.273	3.163	0.00156	T	C	38/914/6328	0.1255	0.1267	0.4052	0.07065	
rs11991462	8	76997004	1.321	3.356	0.0007921	G	A	25/754/6491	0.1037	0.1045	0.5007	0.0575	
rs4735708	8	77014472	1.33	3.465	0.0005311	G	T	25/766/6480	0.1054	0.1059	0.657	0.05841	
rs10504599	8	77019515	1.33	3.465	0.0005311	A	G	25/766/6480	0.1054	0.1059	0.657	0.05841	
rs16939206	8	77028391	1.33	3.465	0.0005311	T	C	25/766/6480	0.1054	0.1059	0.657	0.0584	
rs16939212	8	77046164	1.33	3.465	0.0005311	G	A	25/766/6480	0.1054	0.1059	0.657	0.0584	
rs17424935	8	77300088	1.213	3.314	0.0009211	T	C	130/1653/5494	0.2272	0.2283	0.6442	0.1344	
rs4961201	8	87662367	1.135	2.902	0.003705	G	C	726/3093/3460	0.4249	0.4295	0.3678	0.3158	CNGB3
rs7012943	8	87675164	1.141	3.01	0.002614	T	G	728/3110/3440	0.4273	0.4306	0.5136	0.3176	CNGB3
rs2107070	8	91343230	1.21	3.201	0.001369	T	C	128/1527/5622	0.2098	0.215	0.0435	0.1257	
rs16895317	8	98301311	1.23	3.192	0.001414	T	C	79/1309/5892	0.1798	0.1812	0.5175	0.1036	
rs4593512	8	98395328	1.607	4.01	6.06E-05	T	C	3/345/6903	0.04758	0.04724	0.8015	0.02614	
rs11996689	8	98395590	1.607	4.01	6.06E-05	A	G	3/345/6903	0.04758	0.04724	0.8015	0.02614	
rs11986181	8	98395725	1.607	4.01	6.06E-05	G	T	3/345/6903	0.04758	0.04724	0.8015	0.02614	
rs16895513	8	98396149	1.585	4.02	5.81E-05	T	C	3/374/6903	0.05137	0.05084	0.4912	0.0281	
rs6468544	8	98398941	1.603	4.13	3.62E-05	G	C	3/373/6904	0.05124	0.05071	0.4911	0.0281	
rs16895523	8	98399910	1.604	4.17	3.05E-05	T	C	3/379/6898	0.05206	0.05149	0.4922	0.02855	
rs17741363	8	98405252	1.579	3.837	0.0001247	T	C	3/343/6934	0.04712	0.04679	0.8008	0.02586	
rs17741775	8	98431539	1.425	3.078	0.002086	A	G	3/399/6876	0.05482	0.0541	0.3811	0.02928	
rs1871316	8	98579208	0.8123	-2.837	0.004548	T	G	95/1297/5767	0.1812	0.1861	0.02615	0.1008	
rs4734378	8	98985002	1.273	3.333	0.0008598	G	A	44/1090/6070	0.1513	0.1502	0.5825	0.08383	MATN2
rs2513816	8	98986022	1.154	2.923	0.003468	C	G	364/2584/4331	0.355	0.3515	0.4046	0.2298	MATN2
rs4735508	8	98986987	1.225	2.907	0.003646	A	G	63/1148/6062	0.1578	0.1598	0.3037	0.08932	MATN2
rs2514344	8	99170315	0.8863	-2.863	0.0042	G	T	1824/3623/1788	0.5008	0.5	0.9064	0.4994	C8orf47
rs4735611	8	100413689	0.6389	-3.133	0.001733	T	C	5/445/6608	0.06305	0.06239	0.5632	0.03095	VPS13B
rs4735612	8	100438342	0.6389	-3.133	0.001733	T	A	5/445/6608	0.06305	0.06239	0.5632	0.03096	VPS13B
rs10504988	8	100456378	0.6389	-3.133	0.001733	T	C	5/445/6608	0.06305	0.06239	0.5632	0.03095	VPS13B
rs11989337	8	100465360	0.6752	-2.825	0.004725	T	C	5/450/6617	0.06363	0.06293	0.45	0.0314	VPS13B
rs16897325	8	100469467	0.6752	-2.825	0.004725	C	T	5/450/6617	0.06363	0.06293	0.45	0.0314	VPS13B
rs16897326	8	100469958	0.6459	-3.177	0.001486	A	G	5/478/6633	0.06717	0.06623	0.2832	0.03293	VPS13B
rs11989163	8	100491723	0.6752	-2.825	0.004725	C	T	5/450/6617	0.06363	0.06293	0.45	0.0314	VPS13B
rs7008833	8	100516412	0.6752	-2.825	0.004725	A	G	5/450/6617	0.06363	0.06293	0.45	0.0314	VPS13B
rs7008629	8	100516594	0.6756	-2.821	0.004783	T	A	5/450/6620	0.0636	0.0629	0.45	0.03128	VPS13B
rs11992424	8	100556457	0.6752	-2.825	0.004725	G	A	5/450/6617	0.06363	0.06293	0.45	0.0314	VPS13B
rs11988133	8	100564710	0.6752	-2.825	0.004725	T	C	5/450/6617	0.06363	0.06293	0.45	0.03139	VPS13B
rs9643005	8	105263940	0.8667	-3.176	0.001494	A	G	1076/3238/2369	0.4845	0.4813	0.5936	0.3985	RIMS2
rs1374525	8	105268804	0.8667	-3.176	0.001494	A	G	1076/3238/2369	0.4845	0.4813	0.5936	0.3986	RIMS2

rs1348887	8	105270536	0.8667	-3.176	0.001494	A	G	1076/3238/2369	0.4845	0.4813	0.5936	0.3985	RIMS2
rs1900523	8	105279773	0.8687	-3.086	0.002027	C	G	1028/3226/2368	0.4872	0.4795	0.2001	0.394	RIMS2
rs2511549	8	105285497	0.8683	-3.125	0.001776	C	A	1066/3227/2368	0.4845	0.4809	0.5579	0.3976	RIMS2
rs2441909	8	105286277	0.8689	-3.11	0.001869	G	T	1064/3227/2368	0.4846	0.4808	0.5407	0.3974	RIMS2
rs2511550	8	105289275	0.8689	-3.11	0.001869	G	A	1064/3227/2368	0.4846	0.4808	0.5407	0.3974	RIMS2
rs2511552	8	105291081	0.8683	-3.096	0.00196	C	T	1029/3226/2368	0.4871	0.4796	0.2093	0.3941	RIMS2
rs10955344	8	105292449	0.8689	-3.11	0.001869	C	G	1064/3227/2368	0.4846	0.4808	0.5407	0.3974	RIMS2
rs4734749	8	105292950	0.8683	-3.125	0.001776	C	T	1066/3227/2368	0.4845	0.4809	0.5579	0.3976	RIMS2
rs7005827	8	105293748	0.8683	-3.125	0.001776	G	C	1066/3227/2368	0.4845	0.4809	0.5579	0.3976	RIMS2
rs2511557	8	105293888	0.8689	-3.086	0.002027	G	A	1029/3271/2368	0.4906	0.4798	0.07002	0.3947	RIMS2
rs2028944	8	105312929	0.8707	-3.213	0.001312	A	T	1331/3594/2205	0.5041	0.4925	0.04864	0.4338	RIMS2
rs2441816	8	105332149	0.8512	-3.699	0.0002168	A	G	1089/3523/2660	0.4845	0.4767	0.1683	0.3865	RIMS2
rs2252152	8	105332927	0.8518	-3.449	0.0005627	T	A	846/3062/2667	0.4657	0.4616	0.4874	0.3562	RIMS2
rs10461	8	105333155	0.867	-3.332	0.000863	C	T	1316/3610/2290	0.5003	0.4909	0.1081	0.4273	RIMS2
rs10088658	8	105341376	0.8689	-3.278	0.001047	A	G	1310/3591/2290	0.4994	0.4907	0.1363	0.4267	
rs1374518	8	105343467	0.8712	-3.156	0.001602	G	A	1049/3528/2702	0.4847	0.4742	0.06375	0.3814	
rs1562399	8	105344003	0.867	-3.348	0.0008139	G	A	1323/3629/2328	0.4985	0.4905	0.1658	0.4257	
rs4734751	8	105345053	0.8687	-3.282	0.001029	G	A	1310/3591/2291	0.4993	0.4907	0.1427	0.4267	
rs978669	8	105347322	0.8687	-3.282	0.001029	A	T	1310/3591/2291	0.4993	0.4907	0.1427	0.4265	
rs4734754	8	105347978	0.8687	-3.282	0.001029	T	G	1310/3591/2291	0.4993	0.4907	0.1427	0.4266	
rs4734093	8	105352516	0.8618	-3.278	0.001044	C	T	820/3169/3040	0.4508	0.4501	0.9156	0.3372	
rs4734757	8	105355266	0.8672	-3.328	0.000876	G	A	1305/3591/2308	0.4985	0.4903	0.1634	0.425	
rs1447083	8	105359794	0.8686	-3.198	0.001384	C	T	1168/3494/2290	0.5026	0.487	0.007817	0.4141	
rs2100103	8	105360306	0.8686	-3.198	0.001384	G	A	1168/3494/2290	0.5026	0.487	0.007817	0.4141	
rs10106504	8	105361339	0.8672	-3.239	0.0012	A	G	1163/3494/2308	0.5017	0.4865	0.009704	0.4123	
rs2084280	8	105363284	0.8815	-2.841	0.004499	T	A	913/3334/2917	0.4654	0.4609	0.4122	0.3556	
rs4734761	8	105364522	0.8815	-2.841	0.004499	C	T	913/3334/2917	0.4654	0.4609	0.4122	0.3556	
rs2511606	8	105364871	0.8772	-2.977	0.002914	T	A	951/3407/2921	0.4681	0.4634	0.4039	0.3595	
rs2514672	8	105365087	0.8818	-2.836	0.004573	C	T	914/3336/2918	0.4654	0.4609	0.4269	0.3556	
rs1026054	8	105386455	0.8488	-3.42	0.0006256	G	A	684/2887/3026	0.4376	0.437	0.9326	0.3175	
rs7829028	8	110178706	0.7003	-3.028	0.002461	T	C	14/584/6682	0.08022	0.08053	0.6633	0.04002	TRHR
rs16879277	8	110372169	0.7661	-2.863	0.004196	G	C	32/861/6385	0.1183	0.119	0.6216	0.06122	NUDCD1
rs2468155	8	119196379	0.8619	-2.872	0.004085	T	C	405/2322/4261	0.3323	0.3478	0.0002308	0.22	
rs2514760	8	119197131	0.8619	-2.872	0.004085	A	C	405/2322/4261	0.3323	0.3478	0.0002308	0.22	
rs2451143	8	119197236	0.8619	-2.872	0.004085	A	G	405/2322/4261	0.3323	0.3478	0.0002308	0.22	
rs2445923	8	119197340	0.8619	-2.872	0.004085	G	C	405/2322/4261	0.3323	0.3478	0.0002308	0.22	
rs2468154	8	119197364	0.8599	-2.902	0.003711	A	G	405/2262/4261	0.3265	0.3451	9.60E-06	0.2176	
rs2514761	8	119197880	0.8619	-2.872	0.004085	G	A	405/2322/4261	0.3323	0.3478	0.0002308	0.22	
rs1563564	8	119198041	0.8619	-2.872	0.004085	G	A	405/2322/4261	0.3323	0.3478	0.0002308	0.22	
rs2514762	8	119199892	0.8619	-2.872	0.004085	C	T	405/2322/4261	0.3323	0.3478	0.0002308	0.22	

rs6997530	8	124278338	1.136	2.847	0.004417	T	C	641/2953/3686	0.4056	0.4125	0.1555	0.294	FAM83A
rs6997816	8	124278366	1.138	2.892	0.003831	A	G	641/2938/3701	0.4036	0.4117	0.09307	0.2931	FAM83A
rs6996389	8	124635537	0.8511	-2.996	0.002736	C	T	293/2353/4633	0.3233	0.3223	0.8272	0.1987	
rs2958692	8	134203855	0.8815	-2.998	0.002721	T	C	1439/3549/2290	0.4876	0.4932	0.3418	0.4361	TG
rs11774190	8	136051484	1.347	2.809	0.004963	G	C	15/424/6812	0.05847	0.06065	0.005506	0.03327	
rs7013584	8	136052384	1.347	2.809	0.004963	G	T	15/424/6812	0.05847	0.06065	0.005506	0.03327	
rs6986888	8	138838930	1.138	3.084	0.00204	T	C	1299/3547/2433	0.4873	0.4879	0.9234	0.4269	
rs4909718	8	138992328	0.8369	-2.81	0.00496	T	C	136/1765/5186	0.249	0.2461	0.3345	0.1407	
rs1511832	8	138992699	0.8369	-2.81	0.00496	T	C	136/1765/5186	0.249	0.2461	0.3345	0.1407	
rs1519375	8	139446901	1.159	2.835	0.004589	C	T	253/2044/4802	0.2879	0.2947	0.05327	0.1839	FAM135B
rs2043422	8	142305519	1.135	3.007	0.002642	A	G	1031/3270/2892	0.4546	0.4665	0.03168	0.3768	SLC45A4
rs1998437	9	867032	1.155	2.938	0.003303	T	C	364/2490/4425	0.3421	0.3444	0.563	0.2245	DMRT1
rs1340009	9	1319052	1.317	2.915	0.003552	T	C	9/590/6632	0.08159	0.08055	0.3781	0.04406	
rs1796904	9	1524536	1.219	2.952	0.003155	A	C	84/1074/5880	0.1526	0.1609	4.16E-05	0.09121	
rs3793511	9	2160512	0.8838	-2.958	0.003092	G	C	1806/3600/1865	0.4951	0.5	0.4117	0.491	SMARCA2
rs4740884	9	7363868	0.8833	-2.823	0.004752	C	A	934/3174/3163	0.4365	0.453	0.002061	0.3433	
rs1905760	9	7702066	0.8812	-2.874	0.004058	T	C	948/3338/2994	0.4585	0.4605	0.7216	0.3561	
rs1905761	9	7702116	0.8828	-2.905	0.00367	C	T	1224/3498/2558	0.4805	0.4832	0.6277	0.4049	
rs6474736	9	12808600	0.8439	-3.354	0.0007965	C	G	392/2660/4228	0.3654	0.3612	0.3304	0.2326	C9orf150
rs7875268	9	13977524	1.208	3.994	6.51E-05	T	C	425/2690/4143	0.3706	0.3688	0.7025	0.2501	
rs7859777	9	13998509	1.155	3.33	0.0008683	C	T	855/3282/3138	0.4511	0.4508	0.9585	0.3486	
rs263655	9	16999481	1.134	3.022	0.002514	G	A	1574/3605/2097	0.4955	0.4974	0.7414	0.469	
rs10156461	9	18808890	0.8388	-3.748	0.0001779	T	C	656/3073/3551	0.4221	0.4209	0.8238	0.2946	
rs10963784	9	18815527	0.8308	-3.914	9.09E-05	C	G	658/2969/3427	0.4209	0.423	0.6935	0.2968	ADAMTSL1
rs10963786	9	18816610	0.8348	-3.827	0.0001296	A	G	630/2980/3668	0.4095	0.4129	0.4779	0.2848	
rs10811043	9	18822659	0.8445	-3.596	0.0003233	A	G	732/3011/3132	0.438	0.4391	0.8475	0.3188	ADAMTSL1
rs7874702	9	18825003	0.8276	-3.921	8.81E-05	T	A	666/2977/3173	0.4368	0.4324	0.4165	0.309	ADAMTSL1
rs7039790	9	18853560	0.8253	-2.847	0.004408	A	C	105/1605/5570	0.2205	0.2182	0.4203	0.1215	
rs10125884	9	19148229	0.8605	-2.946	0.003221	C	G	391/2689/4200	0.3694	0.3631	0.1465	0.2337	
rs1007907	9	19750153	1.151	2.856	0.004287	C	T	334/2580/4218	0.3617	0.3517	0.01679	0.2318	SLC24A2
rs16937885	9	20089091	0.8678	-3.24	0.001195	C	G	975/3352/2952	0.4605	0.4631	0.6306	0.3596	
rs10965077	9	21585144	1.185	2.864	0.004187	A	T	131/1608/5406	0.2251	0.2275	0.3764	0.1345	
rs2383197	9	21589233	1.185	2.864	0.004187	T	C	131/1608/5406	0.2251	0.2275	0.3764	0.1345	
rs16938475	9	21590813	1.185	2.864	0.004187	C	G	131/1608/5406	0.2251	0.2275	0.3764	0.1345	
rs16938476	9	21591758	1.19	2.93	0.003388	T	C	126/1599/5412	0.224	0.2257	0.5291	0.1334	
rs12683126	9	21596530	1.189	2.929	0.003397	C	T	127/1610/5543	0.2212	0.2233	0.4308	0.1316	
rs10123003	9	21610510	1.203	3.178	0.001483	T	G	141/1654/5485	0.2272	0.2306	0.2222	0.1366	
rs10965256	9	22141465	0.6603	-3.622	0.0002928	A	G	30/614/6528	0.08561	0.08956	0.0007926	0.04472	
rs10965258	9	22143663	0.6945	-3.282	0.001032	G	A	29/632/6567	0.08744	0.09091	0.002612	0.04566	
rs10965267	9	22151828	0.7332	-2.967	0.003004	G	T	35/677/6567	0.09301	0.09736	0.0004228	0.04927	

rs828580	9	22158464	0.7117	-3.028	0.002463	T	A	25/602/6653	0.08269	0.08555	0.008377	0.04322	
rs1981047	9	22163499	0.7152	-2.861	0.004227	G	T	22/546/6662	0.07552	0.07827	0.005987	0.03951	
rs10511735	9	23637858	0.8299	-2.831	0.004637	A	T	104/1648/5528	0.2264	0.2224	0.1401	0.1251	
rs16907489	9	23646538	0.8095	-2.819	0.004815	T	C	65/1291/5868	0.1787	0.1774	0.5509	0.09608	
rs1332682	9	24701970	2.075	2.901	0.003726	A	G	0/58/7192	0.008	0.00797	1	0.00472	
rs944637	9	25534991	0.8365	-3.01	0.002612	G	T	190/1994/5088	0.2742	0.2732	0.7967	0.1599	
rs7034229	9	26099419	1.281	2.917	0.003534	G	A	23/724/6533	0.09945	0.1002	0.4841	0.05498	
rs7049079	9	26178638	1.338	3.133	0.001729	G	C	23/558/6689	0.07675	0.07963	0.004516	0.04328	
rs11525092	9	26180855	1.36	3.379	0.0007268	C	G	18/602/6660	0.08269	0.0838	0.261	0.04579	
rs10812362	9	26181311	1.338	3.133	0.001729	T	C	23/558/6689	0.07675	0.07963	0.004516	0.04328	
rs10812364	9	26181574	1.345	3.295	0.0009835	A	G	26/589/6665	0.08091	0.08417	0.002958	0.0459	
rs7860531	9	26199816	1.299	2.813	0.004902	A	G	17/603/6660	0.08283	0.08367	0.3981	0.04529	
rs13285728	9	26207742	1.312	2.85	0.004366	T	C	15/582/6656	0.08024	0.08082	0.5589	0.04372	
rs2618011	9	26319422	0.8386	-2.902	0.003706	G	T	172/1874/4841	0.2721	0.2702	0.5925	0.1579	
rs2785092	9	26343893	0.8324	-3.305	0.0009486	G	A	254/2192/4832	0.3012	0.3022	0.786	0.1818	
rs2618018	9	26371609	0.8419	-3.096	0.001958	G	C	247/2183/4850	0.2999	0.3001	0.9378	0.1805	
rs694150	9	26374371	0.8432	-3.056	0.00224	G	A	242/2168/4841	0.299	0.2989	1	0.1796	
rs404134	9	26375912	0.8358	-3.202	0.001366	G	C	241/2157/4882	0.2963	0.2968	0.8745	0.1779	
rs7030013	9	26427223	0.8117	-3.216	0.001298	C	G	131/1665/5356	0.2328	0.2331	0.8792	0.1318	
rs7034296	9	26428146	0.8117	-3.216	0.001298	T	C	131/1665/5356	0.2328	0.2331	0.8792	0.1318	
rs10114322	9	26429023	0.8286	-2.925	0.003448	G	A	127/1695/4850	0.254	0.2495	0.1409	0.1435	
rs10967337	9	26431276	0.8286	-2.925	0.003448	C	T	127/1695/4850	0.254	0.2495	0.1409	0.1435	
rs16910463	9	26434299	0.8057	-3.407	0.0006557	A	C	145/1757/5362	0.2419	0.2421	0.9228	0.1376	
rs17835770	9	27474575	0.8594	-2.959	0.003086	T	C	374/2586/4319	0.3553	0.3531	0.6187	0.2251	MOBKL2B
rs4878428	9	30294445	1.166	2.86	0.004234	T	C	224/1979/4661	0.2883	0.2911	0.4308	0.1801	
rs7022276	9	30301083	1.166	2.86	0.004234	A	G	224/1979/4661	0.2883	0.2911	0.4308	0.1802	
rs10969623	9	30328809	1.166	2.85	0.004376	C	T	207/2061/4696	0.296	0.2922	0.3053	0.181	
rs12339053	9	30337683	1.166	2.85	0.004376	T	C	207/2061/4696	0.296	0.2922	0.3053	0.181	
rs10969635	9	30339300	1.166	2.85	0.004376	G	A	207/2061/4696	0.296	0.2922	0.3053	0.1811	
rs3802428	9	34557200	1.967	3.263	0.001101	A	C	1/85/7176	0.0117	0.01191	0.2283	0.00701	CNTFR
rs7047619	9	34560354	1.967	3.263	0.001101	C	T	1/85/7176	0.0117	0.01191	0.2283	0.00701	TFR LOC4150
rs3763614	9	34574135	1.951	3.317	0.0009104	A	G	1/90/7165	0.0124	0.0126	0.2519	0.00741	CNTFR
rs12001648	9	34575385	1.951	3.317	0.0009104	A	G	1/90/7165	0.0124	0.0126	0.2519	0.00741	CNTFR
rs17269330	9	34592628	1.977	3.424	0.0006175	T	G	1/92/7164	0.01268	0.01287	0.2615	0.00758	
rs12352811	9	34604681	1.977	3.424	0.0006175	C	G	1/92/7164	0.01268	0.01287	0.2615	0.00758	DCTN3
rs2812365	9	34653138	1.171	3.158	0.001588	C	T	304/2356/4620	0.3236	0.3243	0.8566	0.2078	
rs928148	9	34658074	1.171	3.158	0.001588	G	A	304/2356/4620	0.3236	0.3243	0.8566	0.2079	
rs928149	9	34658159	1.171	3.158	0.001588	G	C	304/2356/4620	0.3236	0.3243	0.8566	0.2079	
rs2772547	9	34658878	1.171	3.158	0.001588	G	T	304/2356/4620	0.3236	0.3243	0.8566	0.2078	
rs2812347	9	34659171	1.158	2.893	0.00382	T	C	304/2326/4404	0.3307	0.3301	0.9137	0.2126	

rs2812346	9	34660791	1.164	3.039	0.002373	G	A	305/2362/4613	0.3245	0.3249	0.9138	0.2083	
rs2772548	9	34660988	1.171	3.158	0.001588	T	G	304/2356/4620	0.3236	0.3243	0.8566	0.2079	
rs2256480	9	34664332	1.171	3.158	0.001588	A	C	304/2356/4620	0.3236	0.3243	0.8566	0.2079	
rs2812351	9	34665471	1.171	3.158	0.001588	A	G	304/2356/4620	0.3236	0.3243	0.8566	0.2079	
rs2772558	9	34673790	1.171	3.158	0.001588	C	G	304/2356/4620	0.3236	0.3243	0.8566	0.2079	
rs2772559	9	34676157	1.149	2.838	0.004541	C	T	356/2467/4456	0.3389	0.3414	0.5366	0.2226	
rs10814134	9	34688396	1.149	2.838	0.004541	T	A	356/2467/4456	0.3389	0.3414	0.5366	0.2226	
rs10972659	9	35968321	0.8836	-2.887	0.003885	C	A	1311/3595/2374	0.4938	0.4893	0.4435	0.4221	
rs10814339	9	36144678	0.8106	-2.954	0.003134	C	A	81/1413/5786	0.1941	0.1929	0.6706	0.1061	C9orf19
rs2889334	9	36887133	0.7385	-2.904	0.00369	A	G	14/743/6523	0.1021	0.1003	0.1595	0.05067	PAX5
rs17056164	9	72799873	1.18	3.084	0.002043	A	C	215/1918/5063	0.2665	0.2731	0.04691	0.1674	TRPM3
rs6560173	9	72845897	1.17	2.878	0.004006	G	A	203/1909/5057	0.2663	0.2708	0.1627	0.1654	TRPM3
rs7873024	9	72886514	1.157	3.037	0.002391	T	C	390/2545/4345	0.3496	0.3524	0.4851	0.2331	TRPM3
rs12551523	9	72888767	1.173	2.912	0.003586	T	A	199/1893/5015	0.2664	0.2704	0.2035	0.1653	TRPM3
rs972387	9	72911842	1.148	2.929	0.003399	T	A	444/2465/4371	0.3386	0.3545	0.0001619	0.235	TRPM3
rs17579544	9	74039290	1.499	2.916	0.003547	A	G	2/239/7039	0.03283	0.03282	1	0.01808	GDA
rs4130867	9	74047810	1.486	2.943	0.003254	G	T	2/257/7021	0.0353	0.03521	1	0.01943	GDA
rs2258131	9	78121713	1.193	3.127	0.001764	A	G	153/1778/5349	0.2442	0.2453	0.7023	0.1469	
rs11138606	9	82128141	0.7339	-2.832	0.004628	C	T	14/657/6609	0.09025	0.08967	0.6946	0.04501	
rs11138648	9	82202312	0.8714	-3.189	0.001425	T	G	1601/3331/1884	0.4887	0.4991	0.08491	0.4727	
rs10867626	9	82648284	0.8596	-3.446	0.0005682	G	C	1014/3420/2779	0.4741	0.4701	0.4677	0.3716	
rs10867627	9	82648298	0.8595	-3.478	0.0005043	A	T	1051/3450/2779	0.4739	0.4718	0.728	0.3751	
rs11138869	9	82650117	0.8632	-3.349	0.0008107	G	A	1096/3389/2598	0.4785	0.4775	0.8813	0.3884	
rs11138870	9	82650217	0.8632	-3.349	0.0008107	C	T	1096/3389/2598	0.4785	0.4775	0.8813	0.3883	
rs1041039	9	82652320	0.8727	-3.051	0.002279	T	C	1014/3347/2571	0.4828	0.4748	0.1641	0.3822	
rs1547137	9	82662468	0.8666	-3.236	0.001214	T	C	982/3383/2781	0.4734	0.4683	0.3632	0.3689	
rs10867632	9	82666898	0.8618	-3.408	0.0006554	G	C	1028/3446/2799	0.4738	0.4704	0.5496	0.3726	
rs10868031	9	85409860	1.127	2.875	0.004046	A	G	1691/3594/1968	0.4955	0.4993	0.5255	0.4853	
rs13288052	9	86112597	1.262	3.25	0.001156	G	A	50/1071/5596	0.1594	0.1591	0.9389	0.09051	SLC28A3
rs11140508	9	86116712	1.225	3.433	0.0005966	C	T	123/1561/5596	0.2144	0.2174	0.2358	0.1282	SLC28A3
rs11140510	9	86117953	1.238	3.556	0.0003765	T	C	115/1500/5596	0.208	0.2111	0.2195	0.1243	SLC28A3
rs13291737	9	86118446	1.235	3.505	0.0004571	G	A	115/1487/5384	0.2129	0.2156	0.2916	0.1274	SLC28A3
rs4744515	9	91813131	1.193	3.101	0.001929	A	G	137/1909/5217	0.2628	0.2554	0.01304	0.1532	
rs10992124	9	93624273	0.8839	-2.879	0.003995	A	C	1200/3443/2606	0.475	0.4812	0.2721	0.3969	ROR2
rs7855417	9	93634447	0.8812	-2.951	0.003164	C	G	1211/3463/2606	0.4757	0.4816	0.2954	0.3979	ROR2
rs7867707	9	93637467	0.8839	-2.879	0.003986	G	A	1200/3444/2606	0.475	0.4812	0.2828	0.397	ROR2
rs10992128	9	93639788	0.8839	-2.879	0.003986	C	T	1200/3444/2606	0.475	0.4812	0.2828	0.397	ROR2
rs10992129	9	93640777	0.8839	-2.879	0.003986	C	G	1200/3444/2606	0.475	0.4812	0.2828	0.397	ROR2
rs7863061	9	93641293	0.8839	-2.879	0.003986	T	C	1200/3444/2606	0.475	0.4812	0.2828	0.397	ROR2
rs16907887	9	93647115	0.8783	-3.02	0.002525	A	G	1231/3355/2550	0.4702	0.4829	0.02571	0.4014	ROR2

rs6479376	9	93648851	0.8803	-2.998	0.002716	A	G	1290/3478/2512	0.4777	0.4859	0.1547	0.4101	ROR2
rs10992130	9	93651211	0.8796	-3.007	0.002638	A	C	1257/3472/2551	0.4769	0.4842	0.1996	0.405	ROR2
rs10992132	9	93654447	0.8794	-2.991	0.00278	G	A	1232/3349/2550	0.4696	0.4829	0.02115	0.4014	ROR2
rs1881392	9	93655851	0.8775	-3.061	0.002206	A	G	1256/3471/2552	0.4769	0.4841	0.1995	0.4047	ROR2
rs7039406	9	93656919	0.88	-2.976	0.002922	A	T	1231/3349/2550	0.4697	0.4829	0.02116	0.4014	ROR2
rs10992137	9	93658924	0.8777	-3.055	0.002249	A	G	1257/3473/2550	0.4771	0.4842	0.2082	0.405	ROR2
rs10992139	9	93659619	0.8782	-3.041	0.002355	A	G	1254/3466/2550	0.4768	0.4841	0.1991	0.4046	ROR2
rs10821046	9	94748013	0.8679	-3.17	0.001525	T	C	1037/3377/2583	0.4826	0.4756	0.2182	0.3845	
rs10992552	9	94751135	1.191	3.124	0.001782	T	C	166/1833/5281	0.2518	0.2532	0.6434	0.1527	
rs3936098	9	94751183	0.8679	-3.164	0.001554	T	C	1037/3259/2582	0.4738	0.4748	0.8789	0.3825	FGD3
rs4237217	9	94756785	0.8746	-3.071	0.002134	C	G	1037/3377/2866	0.4639	0.4684	0.409	0.3693	FGD3
rs7021090	9	94762234	0.8746	-3.071	0.002134	T	C	1037/3377/2866	0.4639	0.4684	0.409	0.3693	FGD3
rs7026624	9	94764371	0.8746	-3.071	0.002134	A	T	1037/3377/2866	0.4639	0.4684	0.409	0.3693	
rs7034581	9	99252224	1.148	2.934	0.003351	C	G	461/2827/3991	0.3884	0.3824	0.1876	0.2627	TDRD7
rs10988690	9	100851318	1.152	2.933	0.003355	C	G	396/2500/4359	0.3446	0.3508	0.1321	0.2305	COL15A1
rs379029	9	101119491	1.164	3.287	0.001012	G	A	474/2751/4055	0.3779	0.379	0.8046	0.2592	
rs162201	9	101120239	1.159	3.16	0.00158	A	G	448/2711/4121	0.3724	0.3727	0.9499	0.2523	
rs10988775	9	101148797	1.161	3.449	0.0005627	T	C	1222/3164/2225	0.4786	0.4885	0.1017	0.431	
rs411102	9	101156790	1.197	2.81	0.004947	T	C	92/1281/5780	0.1791	0.1838	0.03349	0.1052	
rs323721	9	101163567	1.185	3.771	0.0001627	C	T	910/3345/2502	0.495	0.4722	7.27E-05	0.3895	
rs10988788	9	101188139	1.163	3.431	0.0006014	C	G	1045/3169/2369	0.4814	0.4798	0.7973	0.4062	
rs10988793	9	101192162	1.154	3.321	0.0008968	C	G	1236/3244/2254	0.4817	0.4886	0.2513	0.4307	
rs10988803	9	101202126	1.214	3.88	0.0001043	T	A	298/2198/4703	0.3053	0.3128	0.04573	0.1992	
rs10512277	9	102527621	0.8751	-3.04	0.002364	C	T	999/3437/2844	0.4721	0.4679	0.4524	0.3681	
rs4743420	9	102527910	0.8746	-3.052	0.002271	A	C	1000/3446/2834	0.4734	0.4683	0.3676	0.3688	
rs10521052	9	102528411	1.144	3.181	0.001469	G	T	1192/3549/2539	0.4875	0.4829	0.4232	0.413	
rs7022939	9	102528615	0.8777	-2.978	0.002898	G	C	1004/3444/2832	0.4731	0.4685	0.4091	0.3693	
rs10512276	9	102528929	1.144	3.184	0.001453	C	G	1188/3555/2537	0.4883	0.4828	0.3438	0.4128	
rs9299341	9	102530705	0.8757	-3.025	0.002488	G	A	1003/3451/2826	0.474	0.4686	0.3419	0.3696	
rs4076912	9	102533572	0.8768	-3.092	0.001991	A	C	1358/3604/2308	0.4957	0.4915	0.474	0.4292	
rs10512288	9	103534637	0.6486	-3.39	0.0006996	T	C	9/524/6747	0.07198	0.07168	0.8701	0.03493	GRIN3A
rs10512290	9	103552744	0.6456	-3.401	0.000671	G	A	14/506/6760	0.06951	0.07066	0.1802	0.03437	
rs16920725	9	103572257	0.6366	-3.435	0.0005923	A	G	11/497/6765	0.06833	0.06881	0.4954	0.03339	
rs10989981	9	104110828	1.179	3.033	0.002425	A	G	196/1958/5126	0.269	0.2707	0.5737	0.1653	
rs10989983	9	104112415	1.173	2.954	0.00314	G	A	203/1935/5142	0.2658	0.2699	0.2077	0.1647	
rs9409370	9	104122739	1.167	2.896	0.003784	C	T	232/2101/4724	0.2977	0.2974	0.9681	0.1857	
rs10990003	9	104125693	1.187	3.154	0.001613	A	G	196/1946/5138	0.2673	0.2696	0.4601	0.1646	
rs1415686	9	105276045	0.85	-3.329	0.000873	A	C	511/2697/4056	0.3713	0.3809	0.0311	0.2514	
rs10816423	9	108515708	0.7609	-2.893	0.003813	A	T	25/866/6388	0.119	0.1179	0.55	0.06075	
rs10816424	9	108517207	0.7624	-2.837	0.004552	T	A	25/835/6420	0.1147	0.1142	0.837	0.05878	

rs10978606	9	108517848	0.7654	-2.814	0.0049	C	A	25/839/6416	0.1152	0.1147	0.7589	0.05911	
rs10121978	9	110165392	0.7393	-3.202	0.001365	T	C	31/884/6346	0.1217	0.1218	0.9233	0.06276	
rs17743418	9	114626301	1.404	3.148	0.001641	G	T	16/386/6817	0.05347	0.05623	0.000337	0.03065	SNX30
rs16917592	9	114749552	0.4421	-2.875	0.004045	C	T	0/160/7049	0.02219	0.02195	1	0.01017	
rs16917610	9	114756929	0.5252	-3.172	0.001516	A	G	2/262/7016	0.03599	0.03587	1	0.01691	
rs1858730	9	117270228	0.8836	-2.846	0.004425	A	G	1297/3472/2199	0.4983	0.4916	0.2624	0.4302	
rs2818321	9	120771649	1.183	3.014	0.002578	A	G	157/1898/5225	0.2607	0.2577	0.3394	0.1551	
rs2818306	9	120787951	1.177	2.912	0.003594	A	G	159/1897/5224	0.2606	0.258	0.4135	0.1551	
rs3861868	9	125875305	1.132	2.852	0.004338	G	A	759/3151/3370	0.4328	0.4357	0.5722	0.3242	
rs1571570	9	127788070	1.155	3.407	0.000657	C	G	1159/3492/2522	0.4868	0.4819	0.4049	0.4099	
rs492656	9	128122921	0.8273	-2.899	0.003741	G	A	129/1600/5551	0.2198	0.2227	0.269	0.1236	
rs2429922	9	128139361	0.8235	-2.832	0.004623	G	A	100/1493/5667	0.2056	0.206	0.8643	0.1131	FAM125B
rs554480	9	128147964	0.8235	-2.832	0.004623	G	A	100/1493/5667	0.2056	0.206	0.8643	0.1131	FAM125B
rs3814126	9	128305618	0.8508	-3.011	0.002607	C	G	300/2335/4644	0.3208	0.3219	0.7708	0.1966	FAM125B
rs11244207	9	132812917	0.8306	-3.815	0.0001364	C	A	524/2986/3770	0.4102	0.4006	0.0435	0.2723	
rs1892004	9	137196747	0.7081	-2.811	0.004934	T	C	16/522/6631	0.07281	0.07429	0.1083	0.03703	
rs4645616	9	137205413	0.6896	-3.25	0.001155	G	C	18/628/6634	0.08626	0.08705	0.4183	0.04345	
rs884524	9	137370325	0.8615	-2.846	0.004429	A	G	334/2516/4430	0.3456	0.3417	0.3544	0.2152	
rs11103001	9	137462785	0.801	-3.125	0.001778	G	A	85/1445/5750	0.1985	0.1972	0.6345	0.1086	
rs12238018	9	137505445	0.8826	-2.866	0.004152	G	C	1005/3374/2898	0.4637	0.4662	0.6508	0.3654	
rs4842080	9	138063844	0.8566	-3.162	0.001569	T	A	502/2798/3980	0.3843	0.3859	0.7383	0.2569	BTBD14A
rs2254143	9	138820929	0.8335	-2.885	0.003913	A	C	144/1794/5342	0.2464	0.2451	0.6671	0.1397	KIAA1984 KI[illegible]
rs2784098	9	138821487	0.8343	-2.878	0.004007	G	A	147/1795/5338	0.2466	0.2458	0.8117	0.1402	[illegible]orf86 KIAA198[illegible]
rs4545536	11	3216111	1.339	2.852	0.004343	T	C	7/488/6785	0.06703	0.06658	0.7239	0.03639	
rs12286769	11	4423445	0.8883	-2.811	0.004941	T	C	1545/3593/2142	0.4935	0.4966	0.6036	0.4547	
rs191353	11	4424214	0.8705	-3.202	0.001364	T	C	1174/3547/2541	0.4884	0.4823	0.2844	0.401	
rs2291842	11	5676243	1.126	2.813	0.004906	C	T	1069/3429/2771	0.4717	0.4726	0.8816	0.3861	RIM22 TRIM2[illegible]
rs7112649	11	6699239	1.141	2.952	0.003154	G	C	746/3280/3152	0.457	0.4438	0.01243	0.3374	GVIN1 GVIN1
rs7942744	11	6700466	1.133	2.808	0.00499	C	T	751/3322/3186	0.4576	0.4437	0.008156	0.337	GVIN1
rs7129133	11	6880837	1.126	2.864	0.00418	G	C	1637/3591/2052	0.4933	0.4984	0.3843	0.4772	
rs6578793	11	6881438	1.127	2.886	0.003905	C	T	1636/3594/2050	0.4937	0.4984	0.424	0.4774	
rs10839656	11	6890346	1.13	2.938	0.003302	C	T	1627/3601/2052	0.4946	0.4983	0.5408	0.4767	
rs11041095	11	6890377	1.129	2.924	0.003452	C	T	1636/3586/2058	0.4926	0.4983	0.3234	0.4768	
rs10769737	11	6890746	1.123	2.808	0.004984	G	T	1637/3580/2063	0.4918	0.4983	0.2689	0.4764	
rs1018002	11	6896370	1.126	2.866	0.004151	G	A	1623/3575/2054	0.493	0.4982	0.3705	0.476	
rs7925068	11	6897139	1.126	2.864	0.004178	C	A	1623/3575/2054	0.493	0.4982	0.3705	0.4761	
rs11605995	11	6899204	1.125	2.827	0.004694	A	C	1618/3528/2043	0.4907	0.4983	0.2012	0.4761	OR2D3
rs2035844	11	6899528	1.125	2.829	0.004676	T	C	1618/3528/2043	0.4907	0.4983	0.2012	0.4761	OR2D3
rs1388535	11	6899830	1.126	2.867	0.00415	C	T	1623/3588/2069	0.4929	0.4981	0.3713	0.4751	OR2D3
rs7479713	11	6900632	1.127	2.88	0.003975	T	G	1623/3587/2070	0.4927	0.4981	0.3588	0.475	

rs994710	11	6901105	1.125	2.841	0.004497	G	A	1621/3584/2075	0.4923	0.4981	0.3231	0.4746	
rs994709	11	6901217	1.126	2.861	0.004218	C	T	1623/3578/2053	0.4932	0.4982	0.3963	0.4761	
rs1491833	11	6902321	1.126	2.866	0.004157	C	G	1623/3577/2054	0.4931	0.4982	0.3833	0.476	
rs1844908	11	6902408	1.126	2.866	0.004157	C	G	1623/3577/2054	0.4931	0.4982	0.3833	0.476	ZNF215
rs1491832	11	6902551	1.125	2.835	0.004584	T	C	1621/3574/2048	0.4934	0.4983	0.4092	0.4763	
rs1466682	11	6904337	1.125	2.835	0.004584	A	T	1621/3574/2048	0.4934	0.4983	0.4092	0.4762	ZNF215
rs11041106	11	6907128	0.8871	-2.873	0.004065	A	G	1787/3380/1901	0.4782	0.4999	0.0002717	0.4862	ZNF215
rs4426121	11	6911115	0.8871	-2.873	0.004065	C	G	1787/3380/1901	0.4782	0.4999	0.0002717	0.4863	ZNF215
rs10839661	11	6914514	0.8871	-2.873	0.004065	C	T	1787/3380/1901	0.4782	0.4999	0.0002717	0.4863	ZNF215
rs1602569	11	6915170	0.8871	-2.873	0.004065	C	T	1787/3380/1901	0.4782	0.4999	0.0002717	0.4863	ZNF215
rs10839663	11	6919547	0.8839	-2.976	0.002921	T	C	1802/3439/1911	0.4808	0.4999	0.001294	0.4867	ZNF215
rs10765861	11	11482000	1.127	2.848	0.004399	A	G	1184/3482/2614	0.4783	0.4807	0.6785	0.4062	GALNTL4
rs1389549	11	12269126	0.878	-2.939	0.003289	T	C	884/3265/3127	0.4487	0.4525	0.4842	0.3417	
rs10741578	11	12271762	0.8807	-2.851	0.004365	G	A	871/3259/3097	0.4509	0.4526	0.7552	0.3419	MICALCL
rs16911154	11	12330256	1.448	3.361	0.0007773	C	A	6/397/6877	0.05453	0.0546	0.8291	0.03012	MICALCL
rs16911157	11	12330673	1.472	3.475	0.0005101	G	A	6/384/6827	0.05321	0.05337	0.6623	0.02952	MICALCL
rs16911159	11	12330765	1.453	3.376	0.0007348	C	T	6/393/6881	0.05398	0.05408	0.8267	0.02984	MICALCL
rs3816360	11	13324326	1.133	2.821	0.004783	C	T	694/3066/3520	0.4212	0.4247	0.4902	0.3103	ARNTL
rs11022968	11	13780338	1.168	3.147	0.001647	T	C	333/2491/4435	0.3432	0.3403	0.4905	0.2224	
rs10832116	11	13781377	1.169	3.173	0.001509	G	T	334/2489/4454	0.342	0.3397	0.5809	0.2219	
rs10832117	11	13781389	1.159	2.954	0.003137	G	A	333/2491/4141	0.3576	0.3505	0.09397	0.2315	
rs1583931	11	13782907	1.169	3.197	0.001387	G	A	345/2497/4436	0.3431	0.342	0.8104	0.2239	
rs980918	11	13784464	1.16	2.975	0.002933	T	A	333/2496/4142	0.3581	0.3507	0.08765	0.2317	
rs7941664	11	13809817	1.17	3.182	0.001465	G	A	332/2496/4436	0.3436	0.3404	0.4482	0.2224	
rs1104823	11	13810823	1.171	3.215	0.001305	G	A	328/2521/4431	0.3463	0.3412	0.2161	0.2232	LOC729147
rs2221342	11	13812941	1.171	3.204	0.001354	G	A	326/2490/4454	0.3425	0.3388	0.3681	0.2211	
rs7127844	11	13815019	1.17	3.182	0.001465	A	G	332/2496/4436	0.3436	0.3404	0.4482	0.2224	
rs1507534	11	13819980	1.171	3.204	0.001354	T	G	326/2490/4454	0.3425	0.3388	0.3681	0.2211	
rs4756770	11	13825796	1.199	3.575	0.0003503	A	T	261/2399/4504	0.3349	0.3246	0.007849	0.2095	LOC729147
rs7121237	11	13827769	1.199	3.575	0.0003503	G	A	261/2399/4504	0.3349	0.3246	0.007849	0.2095	
rs3849162	11	13834774	1.199	3.575	0.0003503	A	G	261/2399/4504	0.3349	0.3246	0.007849	0.2095	
rs7926524	11	13835928	1.199	3.575	0.0003503	C	T	261/2399/4504	0.3349	0.3246	0.007849	0.2095	
rs3849164	11	13836477	1.199	3.575	0.0003503	C	T	261/2399/4504	0.3349	0.3246	0.007849	0.2095	
rs3897973	11	13839228	1.168	3.08	0.002071	T	G	282/2467/4529	0.339	0.3297	0.01721	0.2131	LOC729147
rs1875713	11	13841440	1.184	3.31	0.0009333	A	G	250/2486/4504	0.3434	0.3274	2.62E-05	0.2115	
rs7122947	11	13845946	1.184	3.31	0.0009333	G	A	250/2486/4504	0.3434	0.3274	2.62E-05	0.2115	
rs2403701	11	13849020	1.184	3.31	0.0009333	A	G	250/2486/4504	0.3434	0.3274	2.62E-05	0.2115	
rs11023779	11	15893643	1.129	2.945	0.003229	T	C	1643/3562/2045	0.4913	0.4985	0.2206	0.4776	
rs10458876	11	15895656	1.125	2.866	0.004152	T	C	1665/3570/2045	0.4904	0.4986	0.1585	0.4792	
rs16932318	11	15898608	1.136	3.086	0.002031	G	A	1649/3576/2048	0.4917	0.4985	0.2491	0.4781	

rs11023787	11	15908870	1.134	3.048	0.002301	T	C	1656/3576/2048	0.4912	0.4986	0.2128	0.4787	
rs10832532	11	15915293	1.136	3.086	0.002031	G	T	1649/3576/2048	0.4917	0.4985	0.2491	0.4783	
rs7111730	11	15922928	1.143	3.195	0.0014	A	T	1649/3573/1951	0.4981	0.4991	0.8685	0.4849	
rs11023791	11	15925961	1.143	3.195	0.0014	A	G	1649/3573/1951	0.4981	0.4991	0.8685	0.4849	
rs11023792	11	15928474	1.143	3.195	0.0014	T	C	1649/3573/1951	0.4981	0.4991	0.8685	0.4849	
rs11023794	11	15930190	1.143	3.195	0.0014	C	T	1649/3573/1951	0.4981	0.4991	0.8685	0.4849	
rs12226315	11	15932395	1.135	3.05	0.002286	T	C	1637/3577/2048	0.4926	0.4984	0.3227	0.4773	
rs10832539	11	15961138	0.852	-3.361	0.0007776	C	A	588/2961/3731	0.4067	0.4068	1	0.2786	
rs10832542	11	15963049	0.8565	-3.299	0.0009707	C	T	632/3024/3621	0.4156	0.4156	0.9775	0.2891	SOX6
rs4757560	11	17646738	1.192	2.865	0.004173	C	G	102/1537/5641	0.2111	0.2106	0.8674	0.1227	
rs1483100	11	18882462	1.136	3.081	0.002066	C	G	1647/3554/2079	0.4882	0.4982	0.08595	0.4762	
rs958061	11	18886391	1.134	3.033	0.002418	G	A	1618/3578/2084	0.4915	0.498	0.2687	0.4737	
rs7121353	11	18889251	1.135	3.048	0.002302	G	A	1618/3570/2080	0.4912	0.498	0.2484	0.4741	
rs10741757	11	18891837	1.133	2.896	0.003785	G	A	1380/3580/2080	0.5085	0.4951	0.02363	0.4557	
rs3812784	11	18894043	1.134	3.035	0.002408	C	A	1618/3573/2084	0.4911	0.4979	0.2486	0.4738	
rs7130079	11	18938521	0.8103	-2.93	0.003387	G	C	92/1354/5611	0.1919	0.1942	0.3263	0.1057	
rs7111927	11	18946896	0.8077	-2.971	0.002967	G	C	92/1354/5611	0.1919	0.1942	0.3263	0.1057	
rs7929731	11	25092498	1.233	2.852	0.004343	A	G	42/1039/6112	0.1444	0.1439	0.8697	0.08086	
rs1564745	11	28248131	1.14	2.912	0.003595	A	G	585/2673/4022	0.3672	0.3886	3.34E-06	0.2682	METT5D1
rs7124975	11	36098490	1.134	3.037	0.002388	A	G	1456/3372/2243	0.4769	0.4938	0.004154	0.4495	LDLRAD3
rs7930554	11	36416657	0.8511	-3.685	0.0002286	C	T	1011/3385/2877	0.4654	0.4671	0.7632	0.3647	FLJ14213
rs7943771	11	36416737	0.8888	-2.815	0.004872	A	G	1463/3550/2267	0.4876	0.4939	0.2856	0.4399	FLJ14213
rs3758873	11	36543593	1.172	3.091	0.001996	C	A	256/2268/4756	0.3115	0.309	0.4948	0.1952	
rs10734444	11	36700402	1.126	2.81	0.004952	C	T	1282/3601/2397	0.4946	0.4883	0.2696	0.4287	
rs1401290	11	36701987	1.237	3.937	8.26E-05	T	C	191/1934/5155	0.2657	0.2675	0.5398	0.1641	
rs10501162	11	36703331	1.238	3.948	7.89E-05	A	C	190/1935/5155	0.2658	0.2674	0.599	0.164	
rs12271535	11	36705661	1.236	3.919	8.88E-05	A	G	192/1932/5156	0.2654	0.2675	0.4835	0.1641	
rs4756342	11	36707277	1.245	4.07	4.71E-05	A	G	190/1935/5155	0.2658	0.2674	0.599	0.1642	
rs1996536	11	36707589	1.238	3.948	7.89E-05	A	C	191/1933/5156	0.2655	0.2674	0.5395	0.164	
rs1996537	11	36707703	1.241	4.005	6.21E-05	T	C	192/1930/5158	0.2651	0.2673	0.4829	0.164	
rs4360677	11	36714487	1.149	3.056	0.002244	C	T	563/2936/3724	0.4065	0.4042	0.6624	0.2864	
rs1581278	11	36715633	1.149	3.056	0.002244	T	G	563/2936/3724	0.4065	0.4042	0.6624	0.2864	
rs7925895	11	36716839	1.159	3.023	0.002503	C	T	387/2940/3723	0.417	0.388	2.40E-10	0.2686	
rs1022367	11	36716901	1.149	3.056	0.002244	A	G	563/2936/3724	0.4065	0.4042	0.6624	0.2863	
rs7940927	11	36742363	1.138	2.996	0.002733	T	C	938/3385/2957	0.465	0.4615	0.5422	0.3673	
rs7117392	11	36754183	1.137	2.973	0.00295	T	C	934/3389/2957	0.4655	0.4614	0.4613	0.3669	
rs2035628	11	41157340	0.8842	-2.891	0.003838	T	G	1315/3563/2402	0.4894	0.4889	0.9427	0.4205	
rs12786091	11	41166076	0.8749	-2.968	0.003	T	C	1167/3262/2144	0.4963	0.489	0.2357	0.4198	
rs11823199	11	42679594	1.176	2.862	0.00421	C	T	135/1894/5144	0.264	0.2562	0.009771	0.1531	
rs7342195	11	42846760	1.145	3.21	0.001329	G	A	1208/3553/2519	0.488	0.4838	0.4674	0.4143	

rs10742654	11	42847172	1.149	3.295	0.0009852	T	C	1225/3551/2504	0.4878	0.4846	0.5781	0.4163	
rs7939308	11	42847622	1.154	3.409	0.000651	G	A	1263/3566/2449	0.49	0.4867	0.5797	0.4232	
rs7926119	11	42847843	1.154	3.408	0.0006533	T	C	1264/3565/2451	0.4897	0.4867	0.6131	0.4231	
rs10837954	11	42850230	0.8637	-3.344	0.0008257	T	G	1046/3382/2743	0.4716	0.472	0.9601	0.3774	
rs4545545	11	42851385	1.164	3.181	0.001469	A	G	404/2733/4143	0.3754	0.3681	0.09773	0.2463	
rs12576578	11	42864442	1.164	3.181	0.001469	G	T	404/2733/4143	0.3754	0.3681	0.09773	0.2464	
rs12577761	11	42864712	1.164	3.181	0.001469	G	A	404/2733/4143	0.3754	0.3681	0.09773	0.2464	
rs16937083	11	42878984	1.171	3.243	0.001184	C	T	440/2753/3595	0.4056	0.392	0.004382	0.2708	
rs16937084	11	42879076	1.164	3.181	0.001469	T	C	404/2733/4143	0.3754	0.3681	0.09773	0.2464	
rs12574104	11	42879363	1.164	3.181	0.001469	T	C	404/2733/4143	0.3754	0.3681	0.09773	0.2464	
rs964188	11	42881308	0.8742	-2.986	0.002827	A	G	985/3273/2607	0.4768	0.4721	0.428	0.378	
rs16937096	11	42881996	1.165	3.208	0.001336	G	A	424/2742/4114	0.3766	0.3715	0.2561	0.2498	
rs16937097	11	42882329	1.165	3.208	0.001336	T	C	424/2742/4114	0.3766	0.3715	0.2561	0.2497	
rs16937103	11	42887178	1.165	3.208	0.001336	G	A	424/2742/4114	0.3766	0.3715	0.2561	0.2498	
rs16937105	11	42887219	1.165	3.208	0.001336	T	C	424/2742/4114	0.3766	0.3715	0.2561	0.2498	
rs7942934	11	42889304	0.8636	-3.345	0.0008228	A	C	1046/3383/2743	0.4717	0.472	0.9601	0.3775	
rs16937110	11	42890710	1.165	3.208	0.001336	G	T	424/2742/4114	0.3766	0.3715	0.2561	0.2498	
rs34675564	11	42904365	1.151	2.985	0.002837	T	C	449/2684/4147	0.3687	0.371	0.5913	0.2539	
rs1387312	11	42905338	0.8754	-2.953	0.003149	T	C	978/3272/2603	0.4775	0.4719	0.3435	0.3778	
rs16937147	11	42908548	1.165	3.208	0.001336	C	T	424/2742/4114	0.3766	0.3715	0.2561	0.2498	
rs16937150	11	42910921	1.165	3.208	0.001336	G	A	424/2742/4114	0.3766	0.3715	0.2561	0.2498	
rs16937155	11	42911499	1.165	3.208	0.001336	G	T	424/2742/4114	0.3766	0.3715	0.2561	0.2498	
rs1489215	11	42912317	1.165	3.208	0.001336	T	A	424/2742/4114	0.3766	0.3715	0.2561	0.2497	
rs1489214	11	42912370	1.165	3.208	0.001336	C	A	424/2742/4114	0.3766	0.3715	0.2561	0.2498	
rs7117404	11	47083729	0.7569	-2.931	0.003378	G	A	43/797/6261	0.1122	0.1166	0.002942	0.06024	C11orf49
rs506223	11	56375612	0.8772	-2.926	0.003434	T	G	892/3175/2997	0.4495	0.4556	0.2615	0.3463	
rs596396	11	56375896	0.8772	-2.926	0.003434	A	G	892/3175/2997	0.4495	0.4556	0.2615	0.3463	
rs626464	11	56378041	0.8772	-2.926	0.003434	G	C	892/3175/2997	0.4495	0.4556	0.2615	0.3464	
rs554574	11	56381486	0.8729	-2.955	0.003128	A	T	905/2970/2673	0.4536	0.4635	0.08311	0.3599	
rs2581924	11	56965044	0.4816	-3.286	0.001015	A	G	9/218/7032	0.03003	0.03198	0.0001145	0.01493	
rs475145	11	59233198	1.275	2.849	0.00438	A	G	25/713/6283	0.1016	0.1028	0.2967	0.05639	
rs11235645	11	72441922	1.298	2.996	0.002732	T	C	41/576/6395	0.08214	0.08944	5.56E-09	0.04953	FCHSD2
rs11236208	11	74067969	0.8644	-3.117	0.001826	T	G	645/3057/3473	0.4261	0.4223	0.4674	0.2979	
rs2853066	11	74866805	1.358	2.982	0.002862	T	C	10/467/6801	0.06417	0.06468	0.4662	0.03505	GDPD5
rs682292	11	74877949	1.341	2.85	0.00437	G	A	8/481/6791	0.06607	0.06594	1	0.0356	GDPD5
rs504793	11	74878212	1.341	2.85	0.00437	T	C	8/481/6791	0.06607	0.06594	1	0.0356	GDPD5
rs549034	11	74880782	1.486	3.167	0.001542	T	C	5/289/6883	0.04027	0.04079	0.2412	0.02255	GDPD5
rs1540210	11	74887046	1.341	2.85	0.00437	G	T	8/481/6791	0.06607	0.06594	1	0.0356	GDPD5
rs1793397	11	74890155	1.589	2.829	0.004664	A	G	3/157/7027	0.02184	0.02242	0.06387	0.01255	GDPD5
rs1783559	11	74894676	1.451	2.897	0.003769	T	C	4/278/6959	0.03839	0.03872	0.367	0.02122	GDPD5

rs1320725	11	79167580	1.126	2.841	0.004497	C	T	1435/3595/2250	0.4938	0.4937	1	0.4479	
rs1944948	11	79167858	1.125	2.816	0.00487	C	T	1431/3599/2250	0.4944	0.4937	0.9243	0.4475	
rs675255	11	84348294	0.7771	-3.161	0.001573	C	T	44/1202/5980	0.1663	0.1626	0.05058	0.08655	DLG2
rs596556	11	84365369	0.79	-2.948	0.003199	A	G	43/1175/5993	0.1629	0.1596	0.07645	0.08506	DLG2
rs2509056	11	84366641	0.79	-2.948	0.003199	T	G	43/1175/5993	0.1629	0.1596	0.07645	0.08506	DLG2
rs670998	11	84379747	0.7951	-2.968	0.002995	G	A	63/1194/6023	0.164	0.1649	0.6193	0.08811	
rs688329	11	84389655	0.7886	-2.969	0.002986	G	A	43/1178/5993	0.1633	0.1599	0.07661	0.0852	DLG2
rs689226	11	84389856	0.7962	-2.939	0.003295	G	A	63/1178/5985	0.163	0.1642	0.5196	0.08765	DLG2
rs625495	11	84395787	0.8018	-2.929	0.003399	T	C	75/1209/5996	0.1661	0.1693	0.111	0.09085	
rs635032	11	84396529	0.7904	-2.982	0.002862	C	T	58/1156/6014	0.1599	0.1605	0.7692	0.08545	DLG2
rs1940122	11	84399229	0.7925	-3.038	0.002382	G	T	64/1224/5991	0.1682	0.1685	0.8348	0.09031	
rs1940123	11	84399449	0.7932	-3.017	0.002556	T	A	70/1183/5986	0.1634	0.1661	0.1784	0.08884	DLG2
rs621007	11	84400656	0.7516	-2.932	0.003363	A	G	24/794/6117	0.1145	0.114	0.8333	0.05894	DLG2
rs613315	11	84402052	0.7846	-3.234	0.001222	G	A	69/1290/5921	0.1772	0.1769	0.9472	0.09522	
rs7127990	11	84403723	0.7782	-3.296	0.000979	G	T	65/1266/5908	0.1749	0.1742	0.8395	0.09353	DLG2
rs685294	11	84413363	0.713	-3.546	0.0003908	C	T	28/861/5969	0.1255	0.1248	0.6984	0.06468	DLG2
rs10501586	11	84433725	0.7763	-3.02	0.002528	C	T	51/1033/6196	0.1419	0.1438	0.2884	0.07547	
rs568641	11	84437325	0.7809	-2.953	0.003148	G	A	46/1047/6183	0.1439	0.1443	0.8073	0.07577	
rs555259	11	84439577	0.7816	-2.95	0.00318	T	A	48/1039/6191	0.1428	0.1438	0.5146	0.07553	
rs641655	11	84452983	0.7773	-3.274	0.00106	C	T	68/1230/5981	0.169	0.1701	0.5812	0.0907	
rs1826613	11	84463780	0.7609	-3.223	0.001268	T	C	45/1041/6192	0.143	0.1433	0.8699	0.07498	
rs1377303	11	84477127	0.7997	-2.849	0.004387	A	G	64/1149/6011	0.1591	0.1611	0.273	0.08584	DLG2
rs9634018	11	84477522	0.7972	-2.937	0.003319	C	T	65/1201/6013	0.165	0.1661	0.5721	0.08874	
rs9634004	11	84477670	0.785	-2.953	0.003143	C	A	53/1080/6017	0.151	0.1521	0.534	0.08044	DLG2
rs560214	11	84478951	0.7788	-3.138	0.001703	G	C	60/1136/6084	0.156	0.1576	0.3725	0.08346	
rs595435	11	84479086	0.7627	-3.335	0.0008517	A	G	60/1106/6114	0.1519	0.1542	0.1966	0.08123	
rs1227842	11	84484544	0.785	-2.953	0.003143	C	T	53/1080/6017	0.151	0.1521	0.534	0.08044	DLG2
rs1227843	11	84484590	0.785	-2.953	0.003143	G	A	53/1080/6017	0.151	0.1521	0.534	0.08044	DLG2
rs1238728	11	84484609	0.785	-2.953	0.003143	C	G	53/1080/6017	0.151	0.1521	0.534	0.08044	DLG2
rs679745	11	84487202	0.785	-2.953	0.003143	C	T	53/1080/6017	0.151	0.1521	0.534	0.08044	DLG2
rs475639	11	85367433	0.8519	-3.701	0.0002143	T	C	1130/3425/2723	0.4706	0.476	0.3369	0.3851	PICALM
rs484154	11	85383124	0.8505	-3.744	0.0001813	T	A	1137/3422/2718	0.4702	0.4764	0.2683	0.3858	PICALM
rs677909	11	85435237	0.8459	-3.882	0.0001036	C	T	1149/3382/2749	0.4646	0.4758	0.04342	0.3843	PICALM
rs638509	11	85462627	0.8439	-4.063	4.85E-05	A	C	1740/3580/1949	0.4925	0.4996	0.2312	0.4796	
rs536841	11	85465472	0.8424	-4.107	4.02E-05	C	T	1740/3587/1953	0.4927	0.4996	0.2409	0.4792	
rs541458	11	85465999	0.8423	-4.112	3.92E-05	C	T	1799/3596/1885	0.494	0.4999	0.3134	0.4877	
rs604767	11	85467631	0.8431	-4.088	4.35E-05	C	G	1799/3593/1883	0.4939	0.4999	0.3022	0.488	
rs543293	11	85497725	0.8557	-3.612	0.0003033	A	G	1148/3410/2722	0.4684	0.4766	0.1402	0.3864	
rs659023	11	85502507	0.8572	-3.575	0.0003501	A	G	1171/3439/2670	0.4724	0.4788	0.2601	0.3915	
rs7110631	11	85533835	0.8336	-4.215	2.50E-05	C	G	1210/3506/2564	0.4816	0.4827	0.846	0.4003	

rs3851178	11	85536145	0.8353	-4.24	2.23E-05	G	T	1343/3482/2455	0.4783	0.4883	0.07979	0.4164	
rs10898439	11	85537131	0.8389	-4.085	4.41E-05	G	C	1237/3514/2529	0.4827	0.4843	0.7901	0.4044	
rs7113976	11	85547385	0.8344	-4.307	1.65E-05	C	T	1673/3601/2003	0.4948	0.499	0.481	0.4705	
rs7949528	11	86978269	1.359	2.832	0.004625	A	G	7/439/6673	0.06167	0.06161	1	0.03341	
rs7936294	11	86978601	1.359	2.832	0.004625	C	T	7/439/6673	0.06167	0.06161	1	0.03341	
rs7944286	11	86979793	1.359	2.832	0.004625	A	G	7/439/6673	0.06167	0.06161	1	0.03341	
rs11235221	11	86980506	1.359	2.832	0.004625	G	A	7/439/6673	0.06167	0.06161	1	0.03341	
rs7931538	11	86983643	1.359	2.832	0.004625	T	A	7/439/6673	0.06167	0.06161	1	0.03341	
rs7931854	11	86983712	1.359	2.832	0.004625	T	G	7/439/6673	0.06167	0.06161	1	0.03341	
rs6592394	11	86984050	1.359	2.832	0.004625	T	C	7/439/6673	0.06167	0.06161	1	0.03341	
rs7126228	11	86984334	1.359	2.832	0.004625	C	T	7/439/6673	0.06167	0.06161	1	0.03341	
rs11235224	11	86984958	1.359	2.832	0.004625	G	A	7/439/6673	0.06167	0.06161	1	0.03341	
rs1010309	11	86985302	1.359	2.832	0.004625	T	C	7/439/6673	0.06167	0.06161	1	0.03341	
rs7940053	11	86985829	1.359	2.832	0.004625	A	G	7/439/6673	0.06167	0.06161	1	0.03341	
rs16924603	11	93894907	1.142	2.825	0.004721	C	T	451/2738/4091	0.3761	0.375	0.8268	0.2535	LOC643037
rs16924599	11	93896079	1.147	2.908	0.003641	C	A	445/2730/4105	0.375	0.3736	0.7777	0.2524	LOC643037
rs11020812	11	93906765	1.147	2.908	0.003641	C	T	445/2730/4105	0.375	0.3736	0.7777	0.2524	
rs11020836	11	93948540	1.148	2.906	0.003664	A	G	416/2687/4177	0.3691	0.3666	0.5651	0.2456	PIWIL4
rs7342170	11	93954125	1.154	3.012	0.002594	T	A	419/2679/4182	0.368	0.3664	0.725	0.2456	PIWIL4
rs7126782	11	93963488	1.139	3.038	0.002378	G	T	1072/3521/2684	0.4839	0.4755	0.139	0.3936	PIWIL4
rs7102184	11	94862022	0.8752	-3.119	0.001812	A	G	1342/3668/2270	0.5038	0.4919	0.04043	0.4317	
rs11606101	11	94930169	1.172	3.013	0.002583	C	T	221/2236/4712	0.3119	0.3038	0.02411	0.1905	
rs4753176	11	94930502	1.183	3.733	0.000189	T	G	764/3246/3059	0.4592	0.4473	0.02729	0.3435	
rs16922192	11	94930943	1.16	2.824	0.004737	C	T	225/2275/4722	0.315	0.3061	0.01386	0.1921	
rs2597597	11	97227948	0.8334	-3.151	0.001627	C	T	223/2024/5033	0.278	0.2817	0.2614	0.1657	
rs2086589	11	97866791	1.294	2.874	0.004059	G	C	21/631/6532	0.08783	0.08929	0.1838	0.04898	
rs11605324	11	98903648	0.7437	-2.922	0.003479	T	C	26/736/6518	0.1011	0.1024	0.301	0.05223	CNTN5
rs470206	11	102176388	0.8426	-3.013	0.002589	T	C	231/2114/4935	0.2904	0.2912	0.8092	0.1733	
rs2510175	11	104937031	1.192	2.844	0.004461	A	T	98/1490/5524	0.2095	0.209	0.8649	0.1216	
rs7111410	11	112554497	0.8342	-2.94	0.00328	T	C	161/1878/5241	0.258	0.2565	0.6809	0.1477	NCAM1
rs2186798	11	112633271	1.148	3.139	0.001694	G	C	717/2868/3494	0.4051	0.4231	0.0003986	0.3085	NCAM1
rs17117479	11	114150388	0.8176	-2.953	0.003149	C	T	110/1508/5662	0.2071	0.2092	0.4006	0.1158	
rs4938130	11	114155209	0.8193	-2.91	0.00361	G	C	105/1502/5673	0.2063	0.2075	0.6113	0.1146	
rs1440192	11	115660765	0.741	-2.987	0.00282	C	T	24/772/6482	0.1061	0.1063	0.8251	0.05437	
rs11215993	11	115915905	0.6925	-2.878	0.004001	C	T	17/488/6474	0.06992	0.072	0.02802	0.03618	
rs11215996	11	115921363	0.6694	-2.942	0.003265	T	G	5/478/6797	0.06566	0.06479	0.3616	0.03184	
rs2276340	11	116897176	1.147	2.879	0.003992	A	G	424/2694/4118	0.3723	0.3697	0.5671	0.2486	DSCAML1
rs7941258	11	116898150	0.7699	-2.808	0.004984	T	C	18/922/6193	0.1293	0.1253	0.00589	0.06519	DSCAML1
rs7941460	11	116898197	0.7691	-2.867	0.004143	A	G	19/959/6183	0.1339	0.1295	0.003311	0.06757	DSCAML1
rs1940040	11	116898504	1.147	2.879	0.003992	C	G	424/2694/4118	0.3723	0.3697	0.5671	0.2485	DSCAML1

rs7944704	11	116898555	0.76	-2.982	0.002868	A	G	19/957/6183	0.1337	0.1293	0.003282	0.06741	DSCAML1
rs3016797	11	116915094	1.146	2.871	0.004096	C	G	424/2696/4121	0.3723	0.3697	0.5671	0.2485	DSCAML1
rs490111	11	116931753	1.143	3.066	0.002167	G	A	783/3220/3264	0.4431	0.4417	0.8111	0.334	DSCAML1
rs522742	11	116939175	1.173	3.274	0.00106	A	G	376/2473/4143	0.3537	0.3549	0.7875	0.2348	DSCAML1
rs479601	11	116945381	1.189	3.544	0.0003944	G	A	412/2460/3823	0.3674	0.3702	0.5523	0.25	DSCAML1
rs504935	11	116952155	1.187	3.641	0.0002715	C	T	432/2690/4084	0.3733	0.3716	0.7273	0.2512	DSCAML1
rs536031	11	116956304	1.174	3.428	0.0006075	A	T	456/2740/4084	0.3764	0.3758	0.9255	0.2549	DSCAML1
rs495420	11	116961570	1.172	3.403	0.0006656	C	G	465/2738/4077	0.3761	0.3769	0.852	0.2562	DSCAML1
rs680730	11	116980443	1.156	3.323	0.0008917	T	C	790/3237/3253	0.4446	0.4428	0.7308	0.3347	DSCAML1
rs527005	11	116992039	1.142	2.896	0.003774	G	C	543/2916/3821	0.4005	0.3986	0.7023	0.2789	DSCAML1
rs17123483	11	119293923	0.8444	-3.07	0.002137	A	C	274/2186/4820	0.3003	0.305	0.1913	0.1836	
rs657405	11	120494990	1.147	3.111	0.001867	T	C	716/3178/3383	0.4367	0.4328	0.4646	0.3219	TECTA
rs584329	11	120499848	1.145	3.066	0.002171	T	C	709/3173/3384	0.4367	0.4322	0.3852	0.321	TECTA
rs536069	11	120501502	1.142	2.996	0.002734	A	G	697/3190/3330	0.442	0.4334	0.09759	0.3224	TECTA
rs661189	11	120502179	1.143	3.035	0.002404	G	C	713/3173/3384	0.4365	0.4325	0.4477	0.3212	TECTA
rs643744	11	120505422	1.144	3.048	0.002306	T	G	704/3210/3365	0.441	0.4332	0.1297	0.3221	TECTA
rs586473	11	120505994	1.145	3.066	0.002171	T	C	709/3173/3384	0.4367	0.4322	0.3852	0.321	TECTA TECTA
rs616120	11	120508256	1.145	3.066	0.002171	A	G	709/3173/3384	0.4367	0.4322	0.3852	0.321	TECTA
rs479299	11	120509058	1.145	3.066	0.002171	A	G	709/3173/3384	0.4367	0.4322	0.3852	0.321	TECTA
rs11218742	11	122035458	0.8408	-2.957	0.003108	G	C	187/2047/5002	0.2829	0.2786	0.2055	0.1639	UBASH3B
rs7106417	11	122041146	0.8443	-2.887	0.00389	C	A	187/2045/5042	0.2811	0.2773	0.2534	0.1626	UBASH3B
rs12290043	11	122045738	0.846	-2.857	0.004278	T	G	187/2051/5042	0.2817	0.2776	0.2208	0.1633	STS-1
rs11218773	11	122096018	0.8169	-2.884	0.003924	G	C	92/1458/5730	0.2003	0.2001	1	0.1098	STS-1
rs12541	11	124128702	1.152	3.179	0.001479	C	T	710/3111/3459	0.4273	0.4287	0.7846	0.3149	ESAM
rs3802903	11	124140156	1.2	2.972	0.002958	A	G	105/1515/5646	0.2085	0.2092	0.7791	0.1214	C11orf61
rs3802902	11	124140245	0.8512	-3.791	0.0001502	T	C	1312/3568/2392	0.4906	0.489	0.7919	0.4216	C11orf61
rs3802901	11	124140354	1.154	3.18	0.001473	C	T	671/3002/3460	0.4209	0.4236	0.5953	0.3082	
rs7129148	11	124147884	1.194	2.865	0.004169	C	T	101/1477/5646	0.2045	0.2054	0.6884	0.1188	C11orf61
rs6590094	11	124149046	1.178	3.707	0.0002094	T	C	708/3111/3461	0.4273	0.4285	0.8268	0.3155	C11orf61
rs6590095	11	124149149	1.177	3.683	0.0002302	C	A	702/3111/3463	0.4276	0.428	0.9345	0.3148	C11orf61
rs7126463	11	124151373	1.154	3.18	0.001473	C	A	671/3002/3460	0.4209	0.4236	0.5953	0.3082	C11orf61
rs7934554	11	124153144	1.194	2.865	0.004169	A	G	101/1477/5646	0.2045	0.2054	0.6884	0.1188	C11orf61
rs7124686	11	124155694	1.154	3.18	0.001473	A	G	671/3002/3460	0.4209	0.4236	0.5953	0.3082	C11orf61
rs10466604	11	124159136	1.179	3.575	0.0003504	C	G	513/2833/3934	0.3891	0.3896	0.9281	0.2695	C11orf61
rs10894038	11	128823185	1.131	2.973	0.002949	T	G	1445/3548/2255	0.4895	0.4938	0.4754	0.4471	BARX2
rs329680	11	133347690	1.404	3.397	0.0006809	T	C	10/497/6773	0.06827	0.06849	0.731	0.03783	
rs11224140	11	134347740	1.148	2.904	0.003683	G	A	430/2676/4174	0.3676	0.3678	0.9746	0.2471	
rs12418711	11	134373604	1.146	2.856	0.004292	T	C	422/2651/4181	0.3655	0.3657	0.9488	0.2451	
rs1478746	11	134374817	0.8718	-3.186	0.001445	A	C	1234/3439/2500	0.4794	0.4844	0.3804	0.406	
rs7102094	11	134377514	0.8776	-2.845	0.004436	C	T	718/3147/3415	0.4323	0.4314	0.8705	0.3106	

rs7104706	11	134381320	0.8781	-2.835	0.004588	T	C	721/3144/3415	0.4319	0.4315	0.9567	0.3108	
rs10894947	11	134406258	0.8726	-3.168	0.001535	G	A	1234/3461/2500	0.481	0.4845	0.543	0.4064	
rs1478753	11	134407746	0.8675	-3.106	0.001896	G	A	740/3191/3344	0.4386	0.4359	0.6094	0.3167	
rs10750589	11	134408260	0.8776	-3.044	0.002334	A	G	1246/3436/2497	0.4786	0.4848	0.2841	0.4075	
rs1351189	11	134410875	0.8727	-3.165	0.001552	G	T	1234/3461/2501	0.481	0.4845	0.543	0.4063	
rs737574	11	134414033	0.8727	-3.165	0.001552	A	G	1234/3461/2501	0.481	0.4845	0.543	0.4064	
rs1564253	11	134429752	0.8587	-3.584	0.0003385	G	A	1405/3620/2255	0.4973	0.4932	0.4908	0.4352	
rs10894957	11	134438723	1.161	2.938	0.003302	C	T	332/2938/3406	0.4401	0.394	1.23E-22	0.275	
rs11062034	12	1939971	1.131	2.909	0.003625	C	T	1717/3543/1822	0.5003	0.4999	0.9621	0.4976	DCP1B
rs11062038	12	1959491	0.8875	-2.863	0.004197	C	T	1843/3630/1807	0.4986	0.5	0.8147	0.4975	DCP1B
rs4765870	12	1961137	1.131	2.909	0.00362	A	G	1717/3543/1822	0.5003	0.4999	0.9621	0.4976	DCP1B
rs11062040	12	1961518	1.132	2.952	0.003161	C	T	1735/3565/1843	0.4991	0.4999	0.9058	0.4974	DCP1B
rs4765871	12	1962321	1.131	2.909	0.00362	C	T	1717/3543/1822	0.5003	0.4999	0.9621	0.4976	DCP1B
rs11062041	12	1962806	1.131	2.909	0.00362	C	G	1717/3543/1822	0.5003	0.4999	0.9621	0.4976	DCP1B
rs17769793	12	3548512	1.627	3.6	0.0003187	G	A	4/250/7025	0.03435	0.03482	0.2918	0.01931	PRMT8
rs6416319	12	5038752	0.8659	-3.36	0.0007804	T	C	1227/3517/2534	0.4832	0.4839	0.9228	0.405	
rs4766335	12	5039110	0.8698	-3.259	0.001118	G	A	1231/3516/2531	0.4831	0.484	0.8654	0.4057	
rs680694	12	5055864	0.8721	-3.186	0.001441	C	T	1207/3517/2552	0.4834	0.4829	0.942	0.4026	
rs17219978	12	13541930	0.8764	-2.823	0.004764	G	A	662/3044/3472	0.4241	0.4234	0.9112	0.3001	
rs12813450	12	16482811	0.5819	-2.838	0.004542	C	T	3/267/7010	0.03668	0.0368	0.7429	0.01775	
rs2970797	12	22172193	0.8707	-2.821	0.004793	C	A	489/2693/4098	0.3699	0.3771	0.106	0.2483	
rs9783526	12	22202405	0.8713	-2.833	0.004615	T	A	521/2811/3948	0.3861	0.3892	0.5076	0.2606	
rs11182062	12	42100119	0.8803	-3.01	0.002609	T	C	1624/3696/1960	0.5077	0.4989	0.1389	0.4714	ADAMTS20
rs11182085	12	42133950	0.8768	-3.106	0.001895	G	A	1594/3678/2005	0.5054	0.4984	0.2396	0.4656	ADAMTS20
rs10880501	12	42147132	0.8775	-3.088	0.002014	G	A	1592/3677/2006	0.5054	0.4984	0.2303	0.4654	ADAMTS20
rs11182091	12	42150040	0.8774	-3.092	0.001991	T	C	1593/3677/2006	0.5054	0.4984	0.2396	0.4656	ADAMTS20
rs10506229	12	42150425	0.8775	-3.088	0.002014	C	A	1592/3677/2006	0.5054	0.4984	0.2303	0.4654	ADAMTS20
rs4768501	12	42177665	0.8775	-3.088	0.002014	G	A	1592/3677/2006	0.5054	0.4984	0.2303	0.4654	ADAMTS20
rs2471601	12	45234926	1.137	3.018	0.002548	A	C	1085/3465/2726	0.4762	0.4746	0.7858	0.3923	
rs1946175	12	45244704	1.137	3.018	0.002548	A	T	1085/3465/2726	0.4762	0.4746	0.7858	0.3924	
rs12300125	12	45284697	1.137	3.021	0.002518	A	G	1085/3468/2726	0.4764	0.4746	0.7482	0.3924	
rs7978888	12	60839052	1.192	2.852	0.004349	T	G	91/1597/5290	0.2289	0.2224	0.0154	0.1307	FAM19A2
rs10784645	12	66401556	0.8438	-4.025	5.69E-05	A	G	1491/3591/2116	0.4989	0.4962	0.669	0.4483	
rs7974944	12	66402124	0.8438	-4.025	5.69E-05	A	G	1491/3591/2116	0.4989	0.4962	0.669	0.4482	
rs10784646	12	66405588	0.8444	-4.047	5.19E-05	A	G	1549/3600/2117	0.4955	0.4969	0.7952	0.4526	
rs12579301	12	68910169	1.234	2.912	0.003597	C	T	44/1067/6094	0.1481	0.1475	0.8104	0.08266	
rs17226157	12	68966286	1.227	2.877	0.004011	T	G	52/1092/5943	0.1541	0.1545	0.8174	0.08704	CNOT2
rs17814121	12	68966327	1.235	2.925	0.003443	G	A	43/1098/5951	0.1548	0.153	0.3518	0.08606	CNOT2
rs12309057	12	68968081	1.225	2.846	0.004431	T	C	51/1089/5949	0.1536	0.1539	0.8772	0.08662	CNOT2
rs12297838	12	71034850	0.5288	-3.088	0.002012	A	G	2/251/7027	0.03448	0.03441	1	0.01606	TRHDE

rs2605342	12	73208990	1.256	2.837	0.004554	C	T	22/826/6427	0.1135	0.1124	0.4653	0.06277	
rs2126136	12	73216096	1.266	2.825	0.004732	T	G	18/772/6453	0.1066	0.1053	0.3715	0.05862	
rs590352	12	73218426	1.262	2.86	0.004233	G	C	21/801/6426	0.1105	0.1095	0.52	0.06128	ATXN7L3B
rs2605375	12	73226651	1.262	2.86	0.004233	G	T	21/801/6426	0.1105	0.1095	0.52	0.06128	
rs10778037	12	75065845	1.259	3.057	0.002238	A	G	36/991/6245	0.1363	0.1355	0.7288	0.07548	
rs10860855	12	75068158	1.34	3.682	0.0002313	T	C	27/830/6325	0.1156	0.1155	1	0.06451	
rs11114962	12	80569783	1.148	3.077	0.002094	C	G	661/3129/3490	0.4298	0.4245	0.2941	0.3101	PPFIA2
rs990932	12	82763589	0.8531	-3.095	0.001968	A	C	387/2554/4338	0.3509	0.3527	0.6657	0.225	
rs1841336	12	82766548	0.8531	-3.095	0.001968	T	C	387/2554/4338	0.3509	0.3527	0.6657	0.225	
rs1006187	12	82767644	0.8531	-3.095	0.001968	A	G	387/2554/4338	0.3509	0.3527	0.6657	0.225	
rs1006185	12	82768933	0.8532	-3.093	0.00198	A	G	387/2554/4339	0.3508	0.3527	0.6657	0.2249	
rs1676431	12	88172600	0.8425	-3.281	0.001034	G	A	353/2589/4281	0.3584	0.3521	0.1327	0.2234	
rs7956249	12	89156068	1.275	3.185	0.001446	G	A	39/886/6173	0.1248	0.1266	0.2593	0.07068	
rs17017578	12	89162425	1.26	3.033	0.002423	A	G	39/898/6167	0.1264	0.1279	0.3071	0.07132	
rs337680	12	91354607	1.256	2.946	0.003215	G	A	33/904/6248	0.1258	0.1259	0.9253	0.07032	
rs7314666	12	92948087	1.184	2.809	0.004962	T	C	120/1558/5549	0.2156	0.2178	0.3874	0.1269	
rs11108429	12	95052366	0.8814	-2.932	0.003367	C	G	1172/3561/2547	0.4891	0.4822	0.2242	0.4012	
rs10860187	12	96253131	1.465	3.341	0.0008362	T	G	7/371/6902	0.05096	0.05149	0.3572	0.02805	
rs10860188	12	96253413	1.452	3.27	0.001075	T	C	8/371/6901	0.05096	0.05175	0.1747	0.02816	
rs10860192	12	96260293	1.415	2.957	0.003106	G	A	6/363/6911	0.04986	0.05018	0.4851	0.02709	
rs7132969	12	99410974	1.144	3.027	0.002466	A	G	637/2945/3697	0.4046	0.4116	0.1464	0.2947	NR1H4
rs919213	12	99417767	1.145	3.032	0.002431	T	C	637/2943/3697	0.4044	0.4116	0.1386	0.2948	NR1H4
rs11110397	12	99422778	1.144	3.016	0.002565	A	G	638/2945/3697	0.4045	0.4117	0.1388	0.295	NR1H4
rs11110398	12	99423721	1.138	2.854	0.004314	T	C	564/2891/3825	0.3971	0.3997	0.5776	0.2807	NR1H4
rs2373354	12	99431791	1.14	2.899	0.003747	G	A	563/2887/3825	0.3968	0.3995	0.5771	0.2805	NR1H4
rs17030270	12	99442383	1.139	2.878	0.003996	G	A	564/2891/3825	0.3971	0.3997	0.5776	0.2807	NR1H4
rs12229407	12	99456093	1.14	2.894	0.003798	G	A	584/2919/3751	0.4024	0.4047	0.622	0.2865	NR1H4
rs17030306	12	99462915	1.137	2.858	0.004264	A	G	593/2936/3751	0.4033	0.4059	0.5833	0.2879	NR1H4
rs2607983	12	101423834	1.282	2.934	0.003349	C	T	18/751/6511	0.1032	0.1023	0.5655	0.0561	
rs703679	12	103595922	0.8692	-2.861	0.00423	C	T	486/2783/4011	0.3823	0.3828	0.9026	0.2536	CHST11
rs2694392	12	104467085	1.176	2.999	0.002706	A	G	193/1935/5106	0.2675	0.2694	0.5413	0.1639	
rs11833529	12	104686072	1.132	2.996	0.002732	C	T	1715/3588/1977	0.4929	0.4994	0.27	0.4866	
rs4609683	12	104686299	1.133	3.006	0.002651	A	T	1717/3586/1977	0.4926	0.4994	0.2501	0.4867	
rs4600310	12	104686535	1.129	2.92	0.003501	A	C	1724/3578/1977	0.4916	0.4994	0.181	0.4871	
rs4509866	12	104692300	1.146	3.284	0.001025	T	G	1692/3609/1979	0.4957	0.4992	0.5573	0.4852	
rs10732669	12	104692565	1.147	3.313	0.000923	G	A	1726/3571/1982	0.4906	0.4994	0.1331	0.4876	
rs11112736	12	104700067	1.145	3.263	0.001101	A	G	1692/3594/1991	0.4939	0.4992	0.3722	0.4844	
rs12314159	12	104702951	1.138	3.106	0.001899	T	G	1687/3609/1984	0.4957	0.4992	0.5573	0.4843	
rs11112739	12	104703792	1.138	3.106	0.001895	T	C	1689/3605/1985	0.4953	0.4992	0.5109	0.4844	
rs7979632	12	104707800	1.142	3.191	0.001417	A	G	1684/3613/1982	0.4964	0.4992	0.6386	0.4843	

rs4516058	12	104708342	1.139	3.138	0.001699	G	A	1687/3610/1983	0.4959	0.4992	0.5731	0.4844	
rs1477239	12	104711137	1.141	3.165	0.001549	C	G	1675/3617/1988	0.4968	0.4991	0.7071	0.4833	
rs4270016	12	104720049	1.134	3.032	0.002428	A	C	1546/3567/2167	0.49	0.4964	0.2774	0.4619	
rs7302776	12	104722165	1.141	3.166	0.001545	G	A	1523/3565/2192	0.4897	0.4958	0.2983	0.4589	
rs10778448	12	104729423	1.128	2.901	0.003714	C	T	1679/3615/1986	0.4966	0.4991	0.6725	0.4835	
rs10507200	12	104730648	1.134	3.016	0.002559	C	T	1675/3620/1985	0.4973	0.4991	0.7602	0.4835	
rs7970318	12	104735119	1.134	3.023	0.002503	G	A	1680/3612/1988	0.4962	0.4991	0.6219	0.4836	
rs10778450	12	104738423	1.146	3.256	0.001131	A	G	1165/3439/2676	0.4724	0.4785	0.2811	0.4009	
rs6539419	12	107127146	1.188	3.086	0.00203	G	A	187/1807/4822	0.2651	0.2688	0.2599	0.1645	WSCD2
rs4964662	12	107140674	1.171	2.872	0.004082	A	G	179/1954/5085	0.2707	0.269	0.6303	0.164	WSCD2
rs17320521	12	107142383	1.177	2.97	0.002981	A	G	178/1977/5125	0.2716	0.2691	0.4592	0.1643	WSCD2
rs724833	12	107142972	1.181	3.029	0.002451	A	G	178/1974/5126	0.2712	0.2689	0.4855	0.1642	WSCD2
rs1426365	12	107145548	1.171	2.872	0.004082	A	C	179/1954/5085	0.2707	0.269	0.6303	0.1641	WSCD2
rs4964664	12	107146222	1.179	3.025	0.002486	T	A	181/2002/5095	0.2751	0.2721	0.3656	0.1665	WSCD2
rs2374976	12	107155177	1.145	2.825	0.004724	G	A	523/2860/3238	0.432	0.4159	0.001733	0.2998	WSCD2
rs7302554	12	118014807	1.138	3.116	0.001833	G	A	1451/3503/2326	0.4812	0.4928	0.04572	0.4453	KIAA1853
rs12829158	12	118023771	1.14	3.173	0.001506	A	G	1444/3499/2337	0.4806	0.4925	0.04069	0.4442	KIAA1853
rs1243016	12	123663952	0.4488	-2.827	0.004692	C	G	0/158/7122	0.0217	0.02147	1	0.00973	
rs1355553	12	125751945	0.8279	-3.101	0.001928	G	A	168/1870/5242	0.2569	0.2571	0.9274	0.1477	
rs1355554	12	125752125	0.8279	-3.101	0.001928	G	A	168/1870/5242	0.2569	0.2571	0.9274	0.1477	
rs4480580	12	127090839	0.7649	-3.053	0.002265	G	C	42/972/6265	0.1335	0.1346	0.4865	0.07037	
rs11614634	12	127095258	0.7366	-2.898	0.003755	G	T	23/695/6299	0.09905	0.1	0.4023	0.05112	
rs11059862	12	127791702	1.217	3.058	0.00223	C	T	69/1398/5813	0.192	0.1887	0.1528	0.108	
rs11059865	12	127793208	1.248	3.265	0.001095	A	G	69/1107/5983	0.1546	0.1588	0.03059	0.0898	
rs11059866	12	127793341	1.249	3.253	0.001143	A	T	68/1092/5989	0.1527	0.157	0.02374	0.08859	
rs7302136	12	127793593	1.259	3.341	0.0008359	A	G	64/1087/5991	0.1522	0.1556	0.06754	0.08772	
rs7138984	12	127793872	1.248	3.265	0.001095	C	T	69/1107/5983	0.1546	0.1588	0.03059	0.0898	
rs12227382	12	127794827	1.248	3.373	0.0007437	C	T	70/1247/5959	0.1714	0.1725	0.5865	0.09817	
rs2398428	12	127801519	1.156	3.232	0.00123	G	A	616/2993/3669	0.4112	0.412	0.8645	0.2943	
rs10847765	12	128151066	0.8801	-2.94	0.003283	A	G	1042/3435/2803	0.4718	0.4707	0.8616	0.3743	TMEM132D
rs10773608	12	128243907	1.185	3.808	0.00014	T	C	634/3021/3625	0.415	0.4156	0.9102	0.3006	TMEM132D
rs2895125	12	128246898	1.157	3.397	0.0006821	T	G	903/3212/3136	0.443	0.4526	0.07336	0.352	TMEM132D
rs7959926	12	128505334	1.149	2.987	0.002813	T	C	493/2753/3990	0.3805	0.3832	0.5396	0.2628	TMEM132D
rs4128584	12	130326346	0.8831	-2.947	0.003204	T	G	1293/3461/2513	0.4763	0.4859	0.09106	0.4102	
rs12578896	12	130330327	0.8768	-3.129	0.001755	G	A	1408/3550/2322	0.4876	0.4921	0.446	0.4313	
rs2120220	13	18380972	1.16	2.9	0.003726	A	G	281/2151/4600	0.3059	0.3114	0.1455	0.1972	
rs966557	13	19647143	1.634	3.473	0.0005139	A	C	2/236/7042	0.03242	0.03242	1	0.01786	
rs4769970	13	19647841	1.539	3.06	0.002217	C	G	2/250/7028	0.03434	0.03428	1	0.01865	
rs7330666	13	19648106	1.519	2.969	0.002985	G	A	2/252/7026	0.03462	0.03455	1	0.01875	
rs2313476	13	19649019	1.528	3.012	0.002591	A	G	2/252/7026	0.03462	0.03455	1	0.01875	

rs4769188	13	21544210	1.264	2.814	0.0049	G	A	21/803/6456	0.1103	0.1093	0.5201	0.06023	
rs497691	13	22775923	1.205	2.926	0.003438	A	G	87/1363/5830	0.1872	0.1888	0.4564	0.1089	SGCG
rs1753096	13	22776547	1.2	2.857	0.004275	G	A	88/1363/5829	0.1872	0.1891	0.42	0.109	SGCG
rs1536365	13	22827095	1.229	3.348	0.0008139	C	T	90/1515/5635	0.2093	0.2067	0.3335	0.1209	SACS
rs2031640	13	22828055	1.24	3.543	0.000396	T	A	96/1549/5635	0.2128	0.2106	0.4036	0.1236	SACS SACS
rs2709235	13	22854140	1.185	2.824	0.004739	C	T	124/1561/5085	0.2306	0.2315	0.7527	0.1372	SACS
rs2182497	13	22855488	1.229	3.348	0.0008139	T	C	90/1515/5635	0.2093	0.2067	0.3335	0.1209	SACS
rs11619233	13	22857999	1.229	3.348	0.0008139	A	G	90/1515/5635	0.2093	0.2067	0.3335	0.1209	SACS
rs17331329	13	22989459	0.6689	-2.96	0.003076	A	G	8/460/6812	0.06319	0.06325	0.8527	0.03129	
rs17331447	13	22990328	0.6863	-2.845	0.004439	C	G	8/476/6796	0.06538	0.0653	1	0.03241	
rs9553150	13	23515611	1.141	3.124	0.001783	T	C	1103/3432/2745	0.4714	0.4746	0.5701	0.3924	
rs4769313	13	23521388	1.141	3.124	0.001783	G	A	1103/3432/2745	0.4714	0.4746	0.5701	0.3924	
rs976418	13	23545986	1.272	2.849	0.004383	A	C	16/782/6482	0.1074	0.1056	0.1487	0.05749	
rs3117848	13	25197213	1.129	2.859	0.004252	A	G	1091/3446/2724	0.4746	0.4747	0.9803	0.392	ATP8A2
rs3132364	13	25197814	1.131	2.89	0.003851	G	A	1093/3479/2708	0.4779	0.4754	0.6751	0.3925	ATP8A2
rs9553858	13	26036472	1.132	2.89	0.003853	C	T	1103/3594/2583	0.4937	0.4793	0.011	0.4027	WASF3
rs6490409	13	29002154	0.8542	-3.691	0.0002236	A	G	1272/3549/2459	0.4875	0.4867	0.9042	0.4122	SLC7A1
rs3783257	13	29004466	0.8579	-3.587	0.0003349	T	G	1258/3542/2480	0.4865	0.4859	0.9231	0.4099	SLC7A1
rs12428026	13	29005963	0.8541	-3.655	0.0002571	T	C	1143/3493/2644	0.4798	0.4787	0.8639	0.3906	SLC7A1
rs914362	13	29010971	0.8708	-3.223	0.001271	C	T	1186/3532/2562	0.4852	0.4821	0.6097	0.4001	SLC7A1
rs476506	13	29036036	0.8792	-2.819	0.004812	T	G	701/3161/3418	0.4342	0.4304	0.4621	0.3095	SLC7A1
rs1877463	13	29045596	0.8793	-2.975	0.002927	A	G	1089/3484/2707	0.4786	0.4753	0.5706	0.384	SLC7A1
rs280933	13	29052546	0.8801	-2.955	0.003126	C	G	1089/3484/2707	0.4786	0.4753	0.5706	0.3841	SLC7A1
rs280934	13	29053020	0.8812	-2.929	0.003402	T	A	1089/3480/2710	0.4781	0.4752	0.6218	0.3839	SLC7A1
rs280936	13	29054317	0.8779	-3.018	0.002549	G	A	1101/3478/2701	0.4777	0.4758	0.7488	0.3852	SLC7A1
rs166828	13	29055039	0.8834	-2.87	0.0041	C	G	1092/3487/2701	0.479	0.4756	0.5542	0.3849	SLC7A1
rs1556428	13	30032432	0.7073	-3.654	0.0002585	G	A	35/896/6346	0.1231	0.1239	0.5698	0.06335	
rs1556429	13	30032712	0.7109	-3.596	0.0003226	G	A	34/892/6348	0.1226	0.1233	0.6342	0.06303	
rs7989032	13	30033463	0.7109	-3.596	0.0003226	C	T	34/892/6348	0.1226	0.1233	0.6342	0.06303	
rs9506340	13	30033656	0.7109	-3.596	0.0003226	C	G	34/892/6348	0.1226	0.1233	0.6342	0.06303	
rs7320361	13	35239322	1.807	3.318	0.000907	C	T	2/123/7155	0.0169	0.01729	0.1043	0.01001	
rs4520715	13	36357113	1.217	3.03	0.002449	T	C	71/1378/5829	0.1893	0.187	0.3162	0.1072	SMAD9
rs12427600	13	36358648	1.215	3.01	0.002612	C	T	71/1380/5829	0.1896	0.1872	0.3163	0.1073	
rs7333607	13	36360010	1.214	2.997	0.00273	G	A	72/1379/5829	0.1894	0.1873	0.3807	0.1074	
rs2147167	13	36365404	1.209	2.928	0.003417	A	G	73/1382/5821	0.1899	0.188	0.4171	0.1078	SMAD9
rs12020203	13	36367287	1.223	3.015	0.002569	A	G	64/1277/5829	0.1781	0.1768	0.5482	0.1009	SMAD9
rs493248	13	36371386	1.211	2.964	0.003035	T	C	73/1384/5823	0.1901	0.1881	0.3832	0.1079	
rs9531993	13	36373197	1.208	2.915	0.003554	C	G	73/1384/5821	0.1902	0.1881	0.3832	0.1079	SMAD9
rs527929	13	36376506	1.208	2.915	0.003554	T	C	73/1384/5821	0.1902	0.1881	0.3832	0.1079	SMAD9
rs9536429	13	52958882	1.297	3.363	0.0007721	C	T	38/848/6394	0.1165	0.1189	0.0929	0.06639	

rs1413124	13	58108254	0.5759	-3.113	0.001852	C	G	3/311/6943	0.04286	0.04273	1	0.02055
rs9538150	13	58113709	0.5751	-3.121	0.001804	T	C	3/312/6943	0.04299	0.04285	1	0.02061
rs4886097	13	58141613	0.5452	-3.386	0.0007103	C	T	3/319/6943	0.04391	0.04373	1	0.02092
rs4886264	13	60200033	0.6044	-2.849	0.004386	G	C	6/299/6870	0.04167	0.04241	0.1522	0.02047
rs1355902	13	60201119	0.6087	-2.807	0.004995	A	G	6/296/6870	0.04127	0.04202	0.1466	0.02031
rs6562130	13	60202803	0.6044	-2.849	0.004386	C	A	6/299/6870	0.04167	0.04241	0.1522	0.02047
rs6562131	13	60202859	0.6044	-2.849	0.004386	C	G	6/299/6870	0.04167	0.04241	0.1522	0.02047
rs1514166	13	60203791	0.6044	-2.849	0.004386	C	T	6/299/6870	0.04167	0.04241	0.1522	0.02047
rs9592084	13	60493599	1.147	3.01	0.002612	G	A	552/2876/3852	0.3951	0.3973	0.6369	0.2789
rs1905393	13	60710761	1.145	2.883	0.003939	A	G	470/2785/3780	0.3959	0.3893	0.1681	0.2695
rs9539070	13	60711227	1.145	2.883	0.003939	C	T	470/2785/3780	0.3959	0.3893	0.1681	0.2695
rs7993782	13	60723719	1.14	2.814	0.004896	A	G	488/2790/3821	0.393	0.3898	0.5029	0.2698
rs17434465	13	64283635	0.7535	-3.005	0.002658	A	G	21/890/6362	0.1224	0.1199	0.09552	0.06197
rs1591358	13	68895483	0.7574	-3.108	0.001881	A	G	34/990/6256	0.136	0.1348	0.4869	0.06997
rs17609144	13	68926139	0.737	-3.448	0.0005651	T	C	37/1024/6219	0.1407	0.1395	0.5019	0.07244
rs1330538	13	68929072	0.76	-3.017	0.002552	C	G	35/935/6281	0.1289	0.129	0.9276	0.06684
rs1819422	13	68935761	0.7487	-3.195	0.001399	C	G	33/966/6278	0.1327	0.1318	0.5933	0.06832
rs1819541	13	68935859	0.7539	-3.104	0.001909	T	C	31/957/6285	0.1316	0.1303	0.4709	0.06751
rs17683235	13	68940840	0.7539	-3.103	0.001915	G	A	31/958/6289	0.1316	0.1303	0.4711	0.06754
rs17683319	13	68942853	0.7539	-3.103	0.001915	A	G	31/958/6289	0.1316	0.1303	0.4711	0.06754
rs17610390	13	68946100	0.7539	-3.103	0.001915	C	T	31/958/6289	0.1316	0.1303	0.4711	0.06754
rs3916210	13	68947388	0.7414	-3.331	0.0008661	T	C	40/972/6268	0.1335	0.1341	0.7262	0.06947
rs1041375	13	68948010	0.753	-3.156	0.001601	A	T	33/981/6266	0.1348	0.1335	0.4819	0.06931
rs1041374	13	68948153	0.7408	-3.385	0.000711	A	G	39/1010/6231	0.1387	0.1383	0.8653	0.07188
rs1854746	13	68948899	0.7518	-3.122	0.001795	C	T	30/958/6290	0.1316	0.1301	0.3669	0.06728
rs4468490	13	68949981	0.7552	-3.14	0.001692	C	T	41/968/6266	0.1331	0.1339	0.5988	0.06951
rs1964931	13	73749171	0.7604	-3.499	0.0004666	T	C	65/1238/5977	0.1701	0.1703	0.8906	0.09019
rs1412162	13	73755899	0.7626	-3.449	0.0005624	T	C	64/1223/5973	0.1685	0.1688	0.835	0.08937
rs9543558	13	73756948	0.7582	-3.536	0.0004059	A	G	65/1243/5972	0.1707	0.1708	0.9453	0.09046
rs1360499	13	73757635	0.7554	-3.172	0.001513	C	A	50/934/6171	0.1305	0.1341	0.03364	0.06931
rs1831540	13	73758477	0.7499	-3.254	0.001138	G	A	46/946/6128	0.1329	0.1352	0.1598	0.06989
rs2875679	13	73760424	0.7649	-3.442	0.0005773	C	T	69/1238/5973	0.1701	0.1711	0.5836	0.09081
rs1318725	13	73760773	0.7489	-3.271	0.001072	A	C	47/946/6128	0.1328	0.1354	0.1149	0.07
rs966081	13	73763006	0.7489	-3.271	0.001072	C	T	47/946/6128	0.1328	0.1354	0.1149	0.07
rs9544563	13	77130103	1.141	2.901	0.003718	G	A	608/2962/3623	0.4118	0.4122	0.9316	0.2943
rs9593251	13	77131233	1.134	3.019	0.002537	T	C	1334/3512/2434	0.4824	0.4886	0.2805	0.4301
rs1937381	13	77140041	1.13	2.911	0.003599	A	G	1327/3495/2396	0.4842	0.489	0.3997	0.4315
rs1343911	13	77151328	1.159	2.998	0.002717	T	C	342/2379/4484	0.3302	0.3348	0.2456	0.2164
rs7997533	13	77159020	1.166	3.087	0.002019	G	A	325/2327/4500	0.3254	0.3296	0.2818	0.212
rs7324678	13	77161073	1.165	3.062	0.002195	A	G	325/2324/4484	0.3258	0.33	0.2818	0.2123

rs17070073	13	78145736	1.148	3.164	0.001555	C	A	747/3030/3503	0.4162	0.4283	0.01604	0.316
rs4884102	13	78157087	1.153	3.2	0.001373	T	C	668/3070/3522	0.4229	0.4227	1	0.3086
rs9574271	13	78159807	1.149	3.085	0.002033	A	C	665/3043/3419	0.427	0.4253	0.7594	0.3119
rs4885562	13	78177775	1.166	3.359	0.000782	T	G	606/2917/3522	0.4141	0.4143	0.9541	0.2986
rs12585723	13	78178965	1.147	3.079	0.002079	A	G	690/3093/3480	0.4259	0.4262	0.9561	0.3129
rs12584019	13	78181027	1.147	3.079	0.002079	A	T	690/3093/3480	0.4259	0.4262	0.9561	0.3129
rs9530815	13	78187844	1.14	2.977	0.002912	A	G	739/3158/3383	0.4338	0.434	0.9569	0.3235
rs9574275	13	78188215	1.147	3.079	0.002079	C	A	690/3093/3480	0.4259	0.4262	0.9561	0.3129
rs9544871	13	78191091	1.136	2.899	0.003747	T	C	743/3161/3374	0.4343	0.4347	0.957	0.3243
rs9635020	13	78191699	1.147	3.094	0.001973	C	T	700/3062/3518	0.4206	0.4251	0.3776	0.3116
rs17070130	13	78193846	1.157	3.278	0.001044	C	A	677/3081/3522	0.4232	0.4236	0.9339	0.31
rs9565434	13	78194925	1.147	3.094	0.001973	A	G	700/3062/3518	0.4206	0.4251	0.3776	0.3116
rs2876733	13	78196840	1.149	3.085	0.002033	A	T	665/3043/3419	0.427	0.4253	0.7594	0.312
rs9574277	13	78199546	1.156	3.266	0.001092	T	C	692/3076/3501	0.4232	0.4253	0.6591	0.3122
rs1325636	13	78204351	1.135	2.878	0.004004	C	A	725/3133/3419	0.4305	0.4315	0.8492	0.3198
rs9574282	13	78204646	1.135	2.878	0.004004	G	A	725/3133/3419	0.4305	0.4315	0.8492	0.3199
rs12583065	13	78205531	1.135	2.878	0.004004	T	G	725/3133/3419	0.4305	0.4315	0.8492	0.3199
rs9565437	13	78205921	1.136	2.894	0.003806	C	T	743/3163/3374	0.4345	0.4347	0.9785	0.3242
rs7331176	13	78206503	1.135	2.878	0.004004	G	A	725/3133/3419	0.4305	0.4315	0.8492	0.3199
rs9574284	13	78207870	1.135	2.878	0.004004	T	A	725/3133/3419	0.4305	0.4315	0.8492	0.3199
rs4885569	13	78208669	1.136	2.894	0.003806	G	T	743/3163/3374	0.4345	0.4347	0.9785	0.3242
rs4143297	13	78208806	1.147	3.094	0.001973	A	C	700/3062/3518	0.4206	0.4251	0.3776	0.3116
rs4143298	13	78209067	1.135	2.878	0.004004	T	C	725/3133/3419	0.4305	0.4315	0.8492	0.3199
rs1325613	13	78209387	1.14	2.977	0.002912	C	T	739/3158/3383	0.4338	0.434	0.9569	0.3235
rs17070166	13	78210310	1.149	3.139	0.001695	A	C	691/3095/3494	0.4251	0.4259	0.8906	0.3126
rs4120394	13	78210792	1.147	3.094	0.001973	T	C	700/3062/3518	0.4206	0.4251	0.3776	0.3118
rs9574286	13	78211240	1.147	3.094	0.001973	G	A	700/3062/3518	0.4206	0.4251	0.3776	0.3116
rs4884106	13	78211808	1.156	3.268	0.001084	T	C	692/3080/3508	0.4231	0.4252	0.6793	0.312
rs1218194	13	78212120	1.136	2.884	0.003932	T	C	725/3134/3421	0.4305	0.4314	0.8492	0.3198
rs969249	13	78212989	1.136	2.894	0.003806	G	A	743/3163/3374	0.4345	0.4347	0.9785	0.3242
rs1228325	13	78213218	1.136	2.894	0.003806	A	G	743/3163/3374	0.4345	0.4347	0.9785	0.3242
rs1889217	13	78213556	1.14	2.977	0.002912	G	A	739/3158/3383	0.4338	0.434	0.9569	0.3235
rs1931038	13	78213755	1.136	2.894	0.003806	C	T	743/3163/3374	0.4345	0.4347	0.9785	0.3242
rs1409009	13	78214379	1.147	3.094	0.001973	T	A	700/3062/3518	0.4206	0.4251	0.3776	0.3116
rs2255186	13	78216213	1.14	2.977	0.002912	C	A	739/3158/3383	0.4338	0.434	0.9569	0.3235
rs11149107	13	78217071	1.15	3.164	0.001555	C	A	688/3056/3535	0.4198	0.4235	0.455	0.3096
rs9574287	13	78220454	1.147	3.096	0.001962	T	C	687/2957/3554	0.4108	0.4207	0.04669	0.3062
rs9574288	13	78220506	1.136	2.884	0.003932	C	T	725/3134/3421	0.4305	0.4314	0.8492	0.3197
rs9574289	13	78220799	1.147	3.096	0.001962	G	A	687/2957/3554	0.4108	0.4207	0.04669	0.3062
rs9574290	13	78221092	1.147	3.096	0.001962	A	G	687/2957/3554	0.4108	0.4207	0.04669	0.3062

rs17070210	13	78223028	1.15	3.147	0.001647	T	C	692/3053/3535	0.4194	0.4237	0.3762	0.3099	
rs12873802	13	78223550	1.147	3.096	0.001962	G	A	687/2957/3554	0.4108	0.4207	0.04669	0.3062	
rs17070216	13	78223739	1.148	3.127	0.001768	T	C	689/3033/3558	0.4166	0.4223	0.2552	0.3082	
rs1931040	13	78224622	1.136	2.884	0.003932	A	G	725/3134/3421	0.4305	0.4314	0.8492	0.3198	
rs1931041	13	78224653	1.15	3.147	0.001647	C	T	692/3053/3535	0.4194	0.4237	0.3762	0.3099	
rs9318580	13	78230056	1.15	3.163	0.001562	A	G	688/3057/3535	0.4199	0.4235	0.4718	0.3098	
rs4533152	13	78232093	1.14	2.977	0.002912	C	T	739/3158/3383	0.4338	0.434	0.9569	0.3235	
rs348055	13	79712005	0.8384	-3.409	0.0006516	C	T	387/2544/4212	0.3562	0.3566	0.9207	0.2281	
rs370787	13	79714566	0.8389	-3.415	0.0006373	T	C	387/2546/4324	0.3508	0.3528	0.6179	0.2245	
rs390195	13	79716417	0.839	-3.415	0.0006386	T	C	387/2562/4330	0.352	0.3533	0.7402	0.2249	
rs9574612	13	79716812	0.8437	-2.974	0.002939	A	G	246/2148/4212	0.3252	0.3198	0.1782	0.1959	
rs9574613	13	79717033	0.8437	-2.974	0.002939	T	G	246/2148/4212	0.3252	0.3198	0.1782	0.1959	
rs1146942	13	79717730	0.8389	-3.415	0.0006373	G	A	387/2546/4324	0.3508	0.3528	0.6179	0.2245	
rs9545880	13	81311370	0.7591	-3.635	0.0002781	C	T	79/1316/5885	0.1808	0.182	0.5621	0.09729	
rs1591581	13	81313378	0.7624	-3.7	0.0002153	G	A	108/1326/5844	0.1822	0.1894	0.001565	0.1017	
rs1999790	13	81313851	0.7666	-3.472	0.0005172	T	C	67/1308/5852	0.181	0.1796	0.5558	0.09606	
rs4242978	13	81320554	0.7666	-3.472	0.0005172	T	A	67/1308/5852	0.181	0.1796	0.5558	0.09606	
rs9545886	13	81332211	0.7666	-3.472	0.0005172	G	T	67/1308/5852	0.181	0.1796	0.5558	0.09606	
rs4456389	13	81378602	0.8173	-3.095	0.001968	G	A	139/1621/5519	0.2227	0.2269	0.121	0.1268	
rs4112704	13	81378826	0.8189	-3.057	0.002234	A	T	135/1626/5518	0.2234	0.2266	0.234	0.1266	
rs4112703	13	81378893	0.8173	-3.108	0.001887	A	C	143/1634/5503	0.2245	0.229	0.1011	0.1281	
rs7491779	13	81383535	0.8146	-3.177	0.001487	G	A	147/1657/5476	0.2276	0.2321	0.1059	0.1301	
rs7336437	13	81383702	0.8129	-3.207	0.001341	A	G	145/1664/5471	0.2286	0.2324	0.1581	0.1303	
rs2343057	13	85161441	0.819	-2.911	0.003608	A	G	90/1536/5601	0.2125	0.2093	0.196	0.1154	
rs11617680	13	85163519	0.8142	-2.983	0.002859	A	C	92/1527/5605	0.2114	0.2088	0.3107	0.1151	
rs9560792	13	86226273	0.8814	-2.833	0.004611	C	T	866/3281/3132	0.4507	0.4515	0.8763	0.3389	
rs693092	13	87858156	0.8876	-2.871	0.004088	G	A	1659/3530/2061	0.4869	0.4985	0.04783	0.4685	
rs3764127	13	93078438	0.6956	-2.978	0.0029	C	G	16/549/6715	0.07541	0.07662	0.1684	0.03863	GPC6
rs1557024	13	97208138	1.579	2.916	0.003542	G	C	0/188/7092	0.02582	0.02549	0.6353	0.01405	
rs7983469	13	102661160	1.149	2.908	0.003636	G	C	449/2686/3703	0.3928	0.3868	0.211	0.2656	
rs773312	13	104130457	0.8785	-3.034	0.002413	G	T	1278/3545/2453	0.4872	0.487	0.9808	0.4141	
rs2243644	13	105423049	1.63	3.052	0.002275	A	C	0/181/7099	0.02486	0.02455	0.6305	0.01357	
rs12864280	13	109033895	1.139	3.122	0.001794	T	C	1294/3501/2456	0.4828	0.4872	0.4549	0.4248	
rs914270	13	109041018	1.13	2.908	0.003639	G	C	1221/3558/2501	0.4887	0.4845	0.4681	0.4169	
rs942649	13	110752557	1.31	3.503	0.0004596	T	C	40/856/6344	0.1182	0.1209	0.0641	0.06774	ARHGEF7
rs1555751	13	110753466	1.305	3.446	0.0005683	T	C	40/856/6344	0.1182	0.1209	0.0641	0.06774	ARHGEF7
rs4773348	13	110761922	1.331	3.753	0.0001749	G	C	43/857/6363	0.118	0.1214	0.02006	0.06824	
rs6492353	13	110763053	1.331	3.753	0.0001749	A	G	43/857/6363	0.118	0.1214	0.02006	0.06823	
rs7333394	13	110763812	1.333	3.846	0.0001201	T	C	50/878/6352	0.1206	0.1253	0.002581	0.0706	
rs3737220	14	19827644	1.25	2.917	0.003539	A	C	30/972/6278	0.1335	0.1317	0.2848	0.07361	TTC5 TTC5

rs10134743	14	19831456	1.157	3.047	0.002315	G	A	407/2648/4225	0.3637	0.3625	0.7959	0.2412	TTC5
rs11623837	14	19831906	1.156	3.027	0.00247	G	A	407/2659/4214	0.3652	0.3633	0.6749	0.2419	TTC5
rs4981148	14	19833871	1.262	3.218	0.00129	T	C	43/1062/6175	0.1459	0.1453	0.8085	0.08206	TTC5
rs4981951	14	19833934	1.26	3.175	0.001499	C	G	42/1051/6187	0.1444	0.1438	0.8066	0.08113	TTC5
rs8004417	14	20299040	1.311	2.999	0.002711	C	G	23/597/6660	0.08201	0.08442	0.02469	0.04652	
rs11624594	14	20300012	1.301	2.828	0.004677	A	G	15/610/6655	0.08379	0.08405	0.7794	0.04613	
rs8013362	14	20326763	0.8305	-3.408	0.0006549	A	T	292/2314/4673	0.3179	0.3189	0.7969	0.1948	
rs10136056	14	20822363	0.8777	-2.95	0.003182	A	G	925/3344/3011	0.4593	0.4589	0.9593	0.3521	
rs4981415	14	21784072	0.873	-3.009	0.002619	C	G	785/3088/3407	0.4242	0.4351	0.03333	0.3153	
rs10483344	14	28548157	0.8847	-2.846	0.004422	C	G	1352/3668/2219	0.5067	0.4928	0.0171	0.4346	
rs1957468	14	28559642	0.8801	-2.971	0.002964	G	A	1369/3692/2217	0.5073	0.4932	0.01535	0.4361	
rs4981678	14	28560352	0.872	-3.186	0.001441	C	T	1400/3701/2178	0.5084	0.4943	0.01557	0.4405	
rs1957467	14	28567367	0.8797	-2.978	0.002901	A	G	1363/3681/2217	0.507	0.4931	0.01731	0.4355	
rs1957465	14	28568676	0.8799	-2.977	0.002915	T	C	1370/3692/2217	0.5072	0.4932	0.01638	0.4361	
rs17114423	14	28569442	0.8799	-2.977	0.002915	G	C	1370/3692/2217	0.5072	0.4932	0.01638	0.4362	
rs12882489	14	31996133	0.7053	-2.878	0.004001	T	C	15/546/6719	0.075	0.07599	0.2768	0.0379	AKAP6
rs11156798	14	33070254	0.8711	-2.9	0.003726	G	A	705/3360/2850	0.4859	0.4519	3.29E-10	0.3412	NPAS3
rs4981197	14	33070496	0.8711	-2.9	0.003726	T	G	705/3360/2850	0.4859	0.4519	3.29E-10	0.3411	NPAS3
rs8004056	14	33070798	0.8557	-3.55	0.0003854	G	C	1020/3473/2775	0.4778	0.4708	0.2129	0.3744	NPAS3
rs1957317	14	33071692	0.8486	-3.645	0.0002676	T	C	937/3319/2753	0.4735	0.4664	0.2096	0.3653	NPAS3
rs11156799	14	33072207	0.8543	-3.583	0.0003394	A	T	1030/3363/2753	0.4706	0.4709	0.9599	0.3745	NPAS3
rs10145123	14	33072340	0.8543	-3.583	0.0003394	T	C	1030/3363/2753	0.4706	0.4709	0.9599	0.3745	NPAS3
rs10134389	14	33072470	0.8485	-3.783	0.0001549	C	T	1082/3429/2767	0.4711	0.4732	0.7103	0.3789	NPAS3
rs10134498	14	33072608	0.8634	-3.394	0.0006886	C	T	1099/3455/2723	0.4748	0.4751	0.9606	0.3837	NPAS3
rs10147920	14	33072760	0.8527	-3.635	0.0002782	A	G	1034/3377/2767	0.4705	0.4709	0.9401	0.3743	NPAS3
rs10148152	14	33072996	0.8457	-3.852	0.000117	A	G	1068/3427/2782	0.4709	0.4723	0.8232	0.377	NPAS3
rs7154051	14	33073144	0.8498	-3.706	0.0002107	G	A	1022/3376/2782	0.4702	0.47	0.98	0.3725	NPAS3
rs7156402	14	33073620	0.8439	-3.848	0.0001192	C	T	1022/3347/2725	0.4718	0.4712	0.9197	0.3747	NPAS3
rs7159470	14	33073744	0.8498	-3.706	0.0002107	G	A	1022/3376/2782	0.4702	0.47	0.98	0.3725	NPAS3
rs1244367	14	33765460	0.8542	-3.699	0.0002167	G	T	1473/3656/2142	0.5028	0.4958	0.2278	0.4484	
rs1244362	14	33766662	1.139	3.03	0.002445	C	T	926/3405/2947	0.4678	0.4614	0.2426	0.3649	
rs1244359	14	33767129	1.139	3.023	0.002507	C	T	927/3406/2947	0.4679	0.4615	0.2531	0.365	
rs4982270	14	34950569	0.8744	-3.081	0.002062	C	G	1006/3430/2844	0.4712	0.4681	0.5991	0.3695	
rs7147379	14	37143888	1.198	2.892	0.003831	C	G	92/1457/5725	0.2003	0.2002	1	0.1159	C14orf25
rs41480647	14	51219584	0.8611	-3.167	0.001538	G	A	618/2939/3723	0.4037	0.409	0.2639	0.2808	FRMD6
rs4344631	14	51271126	0.8852	-2.858	0.004269	T	G	1603/3577/1932	0.503	0.4989	0.5058	0.4717	
rs12147302	14	51330913	1.146	2.809	0.004965	T	C	358/2608/4310	0.3584	0.3525	0.1528	0.2335	
rs8017705	14	51340221	1.149	2.812	0.004927	A	G	323/2531/4425	0.3477	0.3412	0.1065	0.2233	
rs17126688	14	53169552	0.7323	-2.864	0.004182	C	T	15/678/6587	0.09313	0.09252	0.7032	0.04697	
rs7142267	14	53172192	0.6624	-3.215	0.001304	T	C	13/531/6675	0.07356	0.07418	0.4277	0.03671	

rs6573114	14	56511239	1.169	3.362	0.0007747	C	T	497/2890/3893	0.397	0.3912	0.2193	0.2721	
rs17834803	14	62211048	0.7009	-2.948	0.003202	G	A	17/546/6716	0.07501	0.07651	0.122	0.03852	
rs4902352	14	64492549	1.169	3	0.002699	G	A	254/2183/4821	0.3008	0.302	0.7265	0.1891	RAB15
rs1561193	14	68388004	1.133	2.945	0.003229	A	G	1095/3551/2633	0.4878	0.4777	0.07324	0.3984	
rs17565772	14	69826086	1.162	3.345	0.0008236	G	A	576/2875/3829	0.3949	0.4002	0.2657	0.2803	
rs8017803	14	74178175	1.156	3.362	0.0007741	T	G	902/3358/3017	0.4615	0.4578	0.5055	0.3602	
rs2286910	14	74393096	1.14	2.836	0.004571	A	G	498/2803/3979	0.385	0.3857	0.8793	0.2659	PROX2
rs2300599	14	74429194	0.8642	-3.042	0.002351	C	T	559/2965/3756	0.4073	0.4036	0.4502	0.2745	DLST
rs17093914	14	74433394	0.868	-2.95	0.003176	T	C	557/2961/3762	0.4067	0.4031	0.4498	0.2741	DLST
rs732765	14	74435482	1.154	3.125	0.001781	G	A	532/2918/3830	0.4008	0.3974	0.479	0.2791	DLST
rs731952	14	74436200	0.8628	-3.077	0.002091	T	C	559/2962/3759	0.4069	0.4034	0.4856	0.2744	DLST
rs17808467	14	76239145	1.215	2.873	0.004072	A	G	56/1269/5948	0.1745	0.1719	0.2189	0.09754	
rs4243658	14	77158738	0.842	-3.298	0.0009725	A	G	362/2429/4488	0.3337	0.3393	0.1567	0.2126	
rs8017748	14	77174650	0.8391	-3.391	0.0006965	A	G	380/2473/4427	0.3397	0.3455	0.1542	0.218	
rs12050437	14	77178308	0.796	-3.925	8.69E-05	A	T	251/2005/4774	0.2852	0.293	0.02786	0.1738	
rs2288140	14	78339846	0.7903	-3.078	0.002086	A	G	74/1244/5961	0.1709	0.1729	0.3092	0.09209	IRXN3 NRXN3
rs2888002	14	79923089	1.147	2.923	0.003462	C	A	445/2713/4122	0.3727	0.3724	0.9749	0.252	
rs10131611	14	87838368	1.172	2.936	0.003329	G	A	185/2049/5043	0.2816	0.2772	0.1897	0.1697	KCNK10
rs2402110	14	91636736	1.198	2.938	0.003301	G	A	96/1539/5645	0.2114	0.2095	0.4673	0.1224	ATXN3
rs10150807	14	92559353	0.7536	-3.215	0.001303	A	T	34/1001/6240	0.1376	0.1361	0.4375	0.07136	ITPK1
rs4117	14	92588851	0.7237	-3.554	0.0003788	G	C	34/960/6259	0.1324	0.1317	0.7218	0.06818	ITPK1
rs749619	14	92593647	0.7494	-3.286	0.001015	T	C	36/1002/6241	0.1377	0.1367	0.6066	0.07138	ITPK1
rs879362	14	94099067	1.308	3.114	0.001844	G	T	24/699/6542	0.09621	0.09754	0.2298	0.05408	SERPINA4
rs7151768	14	94198943	0.8515	-2.907	0.003648	T	G	278/2233/4743	0.3078	0.3106	0.4497	0.1883	
rs11160203	14	94208985	0.8521	-2.893	0.003812	A	C	277/2232/4743	0.3078	0.3104	0.4723	0.1882	
rs17091245	14	94212197	0.8566	-2.814	0.004886	A	G	281/2255/4742	0.3098	0.3122	0.5233	0.1897	
rs11851941	14	95633577	1.42	3.009	0.002622	T	C	1/380/6899	0.0522	0.0511	0.06503	0.02816	
rs4905430	14	95634046	1.401	2.898	0.003755	G	T	1/384/6895	0.05275	0.05162	0.0655	0.02844	
rs4905431	14	95634303	1.568	3.367	0.0007605	C	T	0/266/6904	0.0371	0.03641	0.1812	0.0204	
rs4905433	14	95634563	1.559	3.324	0.000888	A	T	0/267/6904	0.03723	0.03654	0.1821	0.02046	
rs11849747	14	95635211	1.559	3.324	0.000888	A	G	0/267/6904	0.03723	0.03654	0.1821	0.02046	
rs8013607	14	97553768	0.7789	-3.043	0.002339	G	A	53/1084/6135	0.1491	0.1503	0.4825	0.07884	
rs1456991	14	97558657	0.7894	-2.9	0.003726	C	T	54/1086/6140	0.1492	0.1506	0.4358	0.0791	
rs12894262	14	97560262	0.7848	-2.882	0.003958	C	T	59/992/6153	0.1377	0.1422	0.009924	0.07418	
rs1456992	14	97563255	0.7848	-2.882	0.003958	A	G	59/992/6153	0.1377	0.1422	0.009924	0.07418	
rs8019853	14	97563832	0.7804	-3.061	0.002209	A	G	64/1083/6133	0.1488	0.1525	0.03799	0.08005	
rs12050445	14	97564321	0.7714	-3.162	0.001565	A	G	55/1091/6134	0.1499	0.1514	0.394	0.07932	
rs12892356	14	97566932	0.7646	-3.25	0.001156	T	G	61/1066/6137	0.1468	0.1502	0.06015	0.07851	
rs1547047	14	98335032	0.8045	-3.296	0.0009799	A	G	129/1607/5544	0.2207	0.2234	0.3186	0.1252	
rs8003680	14	98336062	0.8024	-3.337	0.0008465	C	T	131/1609/5540	0.221	0.224	0.2716	0.1256	

rs7174982	15	20517099	1.139	3.023	0.002502	T	C	968/3433/2878	0.4716	0.4656	0.279	0.3743	CYFIP1
rs12902543	15	21717227	1.152	3.041	0.002361	A	G	495/2947/3790	0.4075	0.3962	0.0162	0.2761	
rs12915353	15	21717493	1.152	3.041	0.002361	G	T	495/2947/3790	0.4075	0.3962	0.0162	0.2761	
rs1459961	15	21719148	1.148	3.003	0.002671	G	A	524/2966/3790	0.4074	0.3994	0.08873	0.2794	
rs12908771	15	21721225	1.152	3.041	0.002361	G	A	495/2947/3790	0.4075	0.3962	0.0162	0.2761	
rs17117842	15	21727934	1.172	2.898	0.00376	A	T	204/1828/5159	0.2542	0.2626	0.007995	0.158	
rs12905760	15	21732670	1.148	2.862	0.004212	G	A	431/2673/3795	0.3874	0.3811	0.1744	0.2596	
rs12908270	15	21736010	1.148	2.862	0.004212	C	T	431/2673/3795	0.3874	0.3811	0.1744	0.2596	
rs12900312	15	21737493	1.146	2.953	0.003144	A	G	534/2950/3795	0.4053	0.3996	0.2407	0.2793	
rs4778358	15	21740263	1.146	2.933	0.003352	A	G	508/2883/3795	0.4012	0.3954	0.2212	0.2748	
rs11161364	15	21742389	1.146	2.923	0.003469	A	G	508/2885/3795	0.4014	0.3954	0.2104	0.2749	
rs12916809	15	21742482	1.146	2.923	0.003469	C	T	508/2885/3795	0.4014	0.3954	0.2104	0.2748	
rs883483	15	23783912	1.138	2.84	0.004518	T	A	597/2923/3715	0.404	0.4071	0.5252	0.2886	
rs11635850	15	27553598	1.154	3.22	0.001281	T	C	653/3013/3614	0.4139	0.4173	0.4827	0.3016	
rs717941	15	32984170	1.135	2.826	0.004717	C	A	621/2977/3630	0.4119	0.4133	0.7543	0.2963	AQR
rs4343221	15	44529782	0.7928	-3.142	0.001681	T	C	75/1364/5839	0.1874	0.1864	0.7058	0.1008	
rs8040559	15	44530880	0.8029	-3.566	0.0003625	T	A	161/2025/4859	0.2874	0.2777	0.003038	0.162	
rs1431609	15	44531807	0.8224	-3.363	0.0007722	C	T	197/2202/4837	0.3043	0.2944	0.004024	0.1751	
rs1431608	15	44532959	0.8007	-3.618	0.0002969	A	G	162/2039/4865	0.2886	0.2785	0.002395	0.1626	
rs13380372	15	44534589	0.8479	-2.925	0.003441	C	A	231/2262/4741	0.3127	0.3057	0.05435	0.1841	
rs16955177	15	44534669	0.7813	-4.069	4.73E-05	G	A	179/2029/5043	0.2798	0.275	0.1464	0.1595	
rs6493221	15	44542978	0.7994	-3.316	0.0009122	G	C	108/1731/4750	0.2627	0.2518	0.0003493	0.1436	
rs7182652	15	44578692	0.7947	-2.809	0.004963	A	C	64/1055/5697	0.1548	0.1585	0.05586	0.08384	
rs4775493	15	44578904	0.7926	-3.562	0.0003674	C	G	91/1904/5068	0.2696	0.2517	4.75E-10	0.1433	
rs1898853	15	45877892	0.8522	-2.813	0.004906	C	T	224/2146/4910	0.2948	0.2928	0.6028	0.175	
rs689933	15	51255923	1.214	3.416	0.0006356	T	C	147/3227/3208	0.4903	0.3919	1.43E-107	0.2734	
rs10518725	15	51603044	1.172	2.908	0.003634	A	G	178/1923/5179	0.2641	0.264	1	0.1609	WDR72
rs17663138	15	51781418	0.8867	-2.86	0.004233	A	G	1663/3647/1963	0.5014	0.4991	0.707	0.4753	WDR72
rs1690363	15	53098119	0.8648	-3.39	0.0006984	G	A	1540/3483/1961	0.4987	0.4982	0.9426	0.4629	
rs1690365	15	53098549	0.8648	-3.39	0.0006984	T	C	1540/3483/1961	0.4987	0.4982	0.9426	0.4629	
rs1690367	15	53099066	0.8621	-3.52	0.000431	G	A	1542/3627/2110	0.4983	0.497	0.8319	0.4541	
rs1728873	15	53102462	0.8358	-4.195	2.73E-05	C	T	1267/3486/2527	0.4788	0.485	0.2769	0.4053	
rs7178963	15	53102791	0.8581	-3.512	0.0004445	A	T	1047/3393/2836	0.4663	0.4698	0.5327	0.3704	
rs1690370	15	53103234	0.8427	-3.921	8.80E-05	G	A	1267/3437/2351	0.4872	0.4882	0.8645	0.4155	
rs1690372	15	53103854	0.8427	-3.921	8.80E-05	G	A	1267/3437/2351	0.4872	0.4882	0.8645	0.4155	
rs2899576	15	53107909	0.8581	-3.659	0.0002534	C	T	1770/3632/1878	0.4989	0.4999	0.8696	0.4854	
rs1528472	15	53108420	0.8519	-3.837	0.0001245	C	A	1782/3606/1892	0.4953	0.4999	0.4391	0.4848	
rs17238122	15	53109188	0.8562	-3.73	0.0001916	G	A	1839/3606/1835	0.4953	0.5	0.4255	0.4931	
rs10431784	15	53123341	0.8523	-3.141	0.001686	T	C	404/2650/4226	0.364	0.3622	0.6978	0.2319	
rs538170	15	55878620	1.138	2.909	0.003625	C	T	700/3166/3414	0.4349	0.4305	0.3987	0.3189	

rs11071380	15	56497919	0.8231	-2.859	0.004252	A	C	98/1561/5592	0.2153	0.213	0.3776	0.1184	
rs11071586	15	59165378	0.8826	-2.818	0.00483	C	T	873/3348/3058	0.46	0.4549	0.3538	0.3452	RORA
rs16954052	15	68092291	0.8394	-3.109	0.001875	A	G	232/2114/4934	0.2904	0.2914	0.7478	0.174	
rs7178037	15	68760095	1.267	2.914	0.003565	T	C	29/810/6391	0.112	0.1128	0.531	0.06235	UACA
rs7168338	15	68767370	1.267	2.914	0.003565	C	T	29/810/6391	0.112	0.1128	0.531	0.06235	UACA
rs16954772	15	68821067	1.255	2.836	0.00457	C	T	30/837/6403	0.1151	0.1158	0.6122	0.06407	UACA
rs11852645	15	68821216	1.255	2.836	0.00457	G	T	30/837/6403	0.1151	0.1158	0.6122	0.06407	UACA
rs11855828	15	68821284	1.247	2.824	0.004739	A	C	31/895/6354	0.1229	0.1228	1	0.06819	UACA
rs11852677	15	68821323	1.256	2.855	0.004301	C	T	30/842/6407	0.1157	0.1162	0.686	0.06439	UACA
rs11855915	15	68821551	1.255	2.841	0.004491	T	C	30/843/6407	0.1158	0.1163	0.6864	0.0643	UACA
rs7177970	15	68832308	1.256	2.878	0.003998	G	A	30/863/6386	0.1186	0.1188	0.8436	0.06596	UACA
rs11852640	15	69432717	0.8604	-2.835	0.004585	C	G	340/2436/4010	0.359	0.3538	0.2299	0.2252	THSD4
rs723434	15	72130383	0.8811	-2.827	0.004703	G	A	886/3275/3001	0.4573	0.4564	0.897	0.3475	
rs12903518	15	77324324	1.291	2.871	0.004089	T	C	11/684/6585	0.09396	0.09228	0.1282	0.051	LOC729911
rs4779143	15	77814481	1.527	3.654	0.0002577	A	G	2/371/6907	0.05096	0.05018	0.2436	0.02765	
rs1020056	15	78271142	1.192	2.836	0.004562	G	T	99/1517/5664	0.2084	0.2078	0.8657	0.1198	
rs1865998	15	78271988	1.19	2.813	0.004914	A	G	103/1511/5666	0.2076	0.208	0.8218	0.12	
rs17312955	15	78398684	1.45	2.818	0.004838	G	A	5/267/7008	0.03668	0.03733	0.1912	0.02041	
rs3974426	15	78447776	1.136	2.884	0.003933	C	G	739/3120/3376	0.4312	0.4336	0.6449	0.3229	
rs12594845	15	78971344	1.146	3.259	0.001118	T	C	1362/3537/2381	0.4859	0.4902	0.4586	0.4354	KIAA1199
rs8028364	15	79270785	0.8656	-2.903	0.003695	G	C	505/2675/3635	0.3925	0.3945	0.6674	0.2666	
rs11072993	15	79292599	0.866	-2.891	0.003837	G	C	505/2671/3633	0.3923	0.3945	0.645	0.2666	IL16
rs1995830	15	79300099	0.8686	-2.834	0.004595	A	G	505/2672/3632	0.3924	0.3945	0.6672	0.2669	IL16
rs980618	15	79305264	0.863	-3.034	0.002414	G	A	513/2808/3946	0.3864	0.3884	0.6507	0.2601	IL16
rs11072994	15	79306368	0.8627	-3.04	0.002368	T	A	513/2809/3946	0.3865	0.3884	0.6725	0.2601	IL16
rs4778652	15	79894158	1.285	2.905	0.003667	T	G	21/723/6536	0.09931	0.09956	0.8134	0.05442	
rs7495437	15	83527294	0.5497	-2.882	0.003954	A	G	0/244/6990	0.03373	0.03316	0.2732	0.01554	
rs4635318	15	83527741	0.5497	-2.882	0.003954	C	A	0/244/6990	0.03373	0.03316	0.2732	0.01554	
rs2342120	15	83529824	0.5497	-2.882	0.003954	G	T	0/244/6990	0.03373	0.03316	0.2732	0.01554	
rs4614693	15	85867049	0.8822	-2.992	0.002774	C	G	1608/3634/1992	0.5024	0.4986	0.5398	0.4692	
rs12904008	15	85894985	1.136	3.016	0.002561	A	T	1414/3513/2125	0.4982	0.4949	0.5966	0.4535	
rs4146308	15	85908243	1.135	3.047	0.002312	A	G	1414/3614/2252	0.4964	0.4934	0.6179	0.4465	
rs4887301	15	85908338	1.136	3.025	0.002487	A	G	1414/3513/2126	0.4981	0.4949	0.5966	0.4535	
rs11073721	15	85908736	1.136	3.025	0.002487	C	G	1414/3513/2126	0.4981	0.4949	0.5966	0.4535	
rs7178330	15	85929233	1.134	2.836	0.004564	A	G	683/3186/3411	0.4376	0.4298	0.1267	0.3175	
rs10459693	15	85952837	0.8679	-2.846	0.004429	T	C	425/2841/4010	0.3905	0.3786	0.007738	0.2491	
rs11857638	15	86016574	0.8348	-3.457	0.0005459	A	G	361/2541/4340	0.3509	0.3491	0.6862	0.2212	
rs4319733	15	86020665	0.8404	-3.06	0.00221	A	G	246/2165/4463	0.315	0.3118	0.4169	0.1898	
rs13329210	15	86075188	0.8627	-2.821	0.004789	C	T	330/2584/4364	0.355	0.3464	0.03584	0.2197	
rs950540	15	87555868	0.8393	-3.134	0.001727	T	C	231/2329/4720	0.3199	0.3099	0.00574	0.1882	RLBP1

rs950541	15	87555958	0.8484	-2.927	0.00342	A	G	224/2288/4768	0.3143	0.3052	0.01123	0.1848	RLBP1
rs4482248	15	95099348	1.297	2.931	0.00338	A	G	24/656/6388	0.09281	0.09464	0.1024	0.05242	
rs2895446	15	95109592	1.362	3.77	0.000163	C	T	26/762/6492	0.1047	0.1056	0.4375	0.05917	
rs3812906	15	95126122	1.349	3.687	0.0002266	A	G	28/784/6390	0.1089	0.1098	0.4515	0.06163	
rs2047223	15	99853919	1.148	2.954	0.003141	C	T	497/2790/3977	0.3841	0.3852	0.8075	0.264	
rs9928320	16	5278276	1.14	2.984	0.002849	G	A	802/3210/3265	0.4411	0.4427	0.7508	0.3345	
rs2343519	16	6068521	1.182	3.552	0.0003816	G	C	426/2676/4178	0.3676	0.3672	0.9491	0.2465	A2BP1
rs6500751	16	6072811	1.169	3.508	0.0004515	C	T	645/3080/3555	0.4231	0.4201	0.558	0.3052	A2BP1
rs1352157	16	6077412	1.149	3.214	0.001309	G	A	872/3382/3016	0.4652	0.4565	0.1112	0.3577	A2BP1
rs9928993	16	7187313	1.335	2.977	0.002914	A	G	16/532/6699	0.07341	0.0748	0.1152	0.04071	A2BP1
rs40129	16	9346193	1.142	2.913	0.003577	G	T	539/2888/3853	0.3967	0.3964	0.9764	0.2763	
rs1900718	16	10114153	1.127	2.872	0.004077	G	A	1517/3627/2135	0.4983	0.4964	0.7589	0.4628	GRIN2A
rs6498116	16	10876783	1.127	2.865	0.004168	A	T	1443/3514/2260	0.4869	0.4936	0.2523	0.448	
rs12596540	16	10877977	1.124	2.824	0.004747	A	G	1466/3529/2285	0.4848	0.4937	0.1228	0.4483	CIITA
rs12928665	16	10878975	1.126	2.843	0.004468	A	G	1443/3509/2264	0.4863	0.4935	0.2149	0.4476	CIITA
rs8043545	16	10889846	1.139	3.143	0.00167	G	C	1715/3551/2010	0.488	0.4992	0.05715	0.4847	CIITA
rs4072865	16	10931606	0.8788	-2.948	0.003195	C	T	1008/3425/2847	0.4705	0.4681	0.6887	0.3694	DEXI
rs441349	16	11279260	1.318	2.81	0.00496	T	C	17/534/6701	0.07363	0.07526	0.0828	0.04085	
rs1794022	16	11283947	1.318	2.81	0.00496	G	A	17/534/6701	0.07363	0.07526	0.0828	0.0408	
rs12599908	16	11303416	1.318	2.821	0.004787	A	G	18/528/6726	0.07261	0.07455	0.0383	0.04057	
rs11645131	16	13457053	1.386	3.36	0.0007798	C	T	10/541/6687	0.07474	0.0745	1	0.04093	
rs3751877	16	15062399	0.8278	-2.832	0.004621	C	T	101/1631/5533	0.2245	0.2205	0.123	0.1233	RRN3
rs889201	16	19961872	0.8846	-2.816	0.004863	G	A	1092/3462/2726	0.4755	0.4748	0.9018	0.3827	GPR139
rs17842735	16	20437818	0.8537	-2.835	0.004585	C	A	244/2253/4774	0.3099	0.3059	0.2832	0.1844	
rs151220	16	20625331	1.23	2.944	0.003241	G	A	66/1021/6022	0.1436	0.149	0.003148	0.08403	
rs11860248	16	24485267	0.7075	-2.885	0.003913	G	T	25/530/6339	0.07688	0.08059	0.0007671	0.04059	RBBP6
rs8051759	16	24795643	0.883	-2.966	0.003021	A	G	1593/3661/2026	0.5029	0.4982	0.4376	0.4651	SLC5A11
rs16949000	16	49470302	1.206	3.159	0.001582	T	G	118/1616/5546	0.222	0.222	0.9579	0.1302	
rs8048121	16	49476092	1.193	3.234	0.001222	C	T	203/1959/4612	0.2892	0.2882	0.8004	0.1778	
rs8044493	16	49476584	1.196	3.307	0.0009434	T	A	203/2020/4608	0.2957	0.2921	0.3202	0.181	
rs1995066	16	49477102	1.195	3.353	0.0007982	A	G	207/2116/4957	0.2907	0.2871	0.3077	0.1772	
rs6500345	16	49477284	1.209	3.538	0.0004032	T	C	194/2064/5022	0.2835	0.2801	0.3152	0.172	
rs12447622	16	49477896	1.256	3.423	0.0006201	G	C	60/1280/5780	0.1798	0.1773	0.256	0.1012	
rs8053438	16	49478108	1.19	3.278	0.001046	A	G	207/2105/4966	0.2892	0.2862	0.3898	0.1763	
rs12446199	16	49478513	1.195	3.353	0.0007982	C	T	207/2116/4957	0.2907	0.2871	0.3077	0.1772	
rs11641277	16	50553334	1.244	2.895	0.003792	C	T	42/925/6313	0.1271	0.129	0.2027	0.07172	
rs9932063	16	50561058	1.257	2.959	0.003084	C	T	35/885/6306	0.1225	0.1234	0.5041	0.06838	
rs8058963	16	50572511	1.13	2.953	0.00315	C	T	1519/3550/2211	0.4876	0.4955	0.1776	0.4567	
rs9925629	16	50575131	1.133	3.029	0.002454	A	G	1516/3548/2216	0.4874	0.4954	0.17	0.4563	
rs9302703	16	50576021	1.257	2.959	0.003084	A	G	35/885/6306	0.1225	0.1234	0.5041	0.06839	

rs11076261	16	50584868	1.129	2.941	0.003272	G	A	1511/3551/2216	0.4879	0.4953	0.2014	0.456	
rs1118787	16	50585416	1.133	3.018	0.002546	G	C	1509/3561/2208	0.4893	0.4954	0.2978	0.4564	
rs2540749	16	50791134	0.7905	-3.077	0.00209	T	C	90/1186/5713	0.1697	0.1763	0.002222	0.09487	
rs16950923	16	50792303	0.8217	-2.942	0.003263	C	T	104/1629/5544	0.2239	0.2206	0.2215	0.1229	
rs1420201	16	50793801	0.7905	-3.077	0.00209	T	G	90/1186/5713	0.1697	0.1763	0.002222	0.09487	
rs3095553	16	50794531	0.7905	-3.077	0.00209	C	T	90/1186/5713	0.1697	0.1763	0.002222	0.09488	
rs7200614	16	50794864	0.7905	-3.077	0.00209	A	G	90/1186/5713	0.1697	0.1763	0.002222	0.09487	
rs2256155	16	50795120	0.7905	-3.077	0.00209	A	T	90/1186/5713	0.1697	0.1763	0.002222	0.09488	
rs12931674	16	50795321	0.7905	-3.077	0.00209	A	G	90/1186/5713	0.1697	0.1763	0.002222	0.09487	
rs2540697	16	50795441	0.7905	-3.077	0.00209	A	G	90/1186/5713	0.1697	0.1763	0.002222	0.09488	
rs4785133	16	50796095	0.7905	-3.077	0.00209	A	G	90/1186/5713	0.1697	0.1763	0.002222	0.09487	
rs11647049	16	50796256	0.7905	-3.077	0.00209	C	T	90/1186/5713	0.1697	0.1763	0.002222	0.09487	
rs10521320	16	54133662	1.147	3.256	0.001129	C	A	1171/3472/2636	0.477	0.4797	0.6251	0.4041	AYTL1
rs11866301	16	54552344	0.83	-2.829	0.004665	G	A	105/1688/5454	0.2329	0.2276	0.04968	0.1269	
rs17269270	16	54613776	0.8221	-2.883	0.003941	C	T	102/1580/5598	0.217	0.215	0.4458	0.1186	
rs2303785	16	56495289	1.233	2.857	0.004279	C	T	45/1024/6194	0.141	0.1416	0.6787	0.07972	NGB1 CNGB1
rs1030254	16	59254152	0.8628	-3.173	0.001507	C	G	673/3163/3444	0.4345	0.4276	0.1706	0.3054	
rs1030252	16	59254370	0.8631	-3.166	0.001543	C	T	673/3163/3444	0.4345	0.4276	0.1706	0.3054	
rs11860355	16	63054158	0.8255	-3.1	0.001933	G	A	166/1814/5241	0.2512	0.253	0.5454	0.1446	
rs9302565	16	63066166	1.125	2.834	0.004598	A	G	1617/3665/1998	0.5034	0.4986	0.4242	0.4782	
rs429065	16	63516143	1.15	2.898	0.003754	G	A	373/2704/4203	0.3714	0.3616	0.02135	0.241	
rs9936642	16	70767020	0.8726	-2.926	0.003429	T	C	665/2895/3643	0.4019	0.4145	0.0105	0.2889	
rs16943919	16	74753106	1.244	2.904	0.003681	G	A	47/938/6291	0.1289	0.1318	0.07429	0.0735	
rs10514402	16	74755057	1.245	2.881	0.00397	T	C	40/935/6302	0.1285	0.1298	0.4153	0.07225	
rs2068767	16	74772094	1.236	2.822	0.00478	A	G	47/939/6287	0.1291	0.1319	0.0747	0.07348	
rs7184877	16	75589006	1.376	3.167	0.001542	G	A	9/507/6746	0.06982	0.06968	1	0.03834	
rs7203528	16	75591957	1.38	3.192	0.001412	T	C	9/506/6746	0.06969	0.06956	1	0.03828	
rs7186276	16	75591992	1.38	3.192	0.001412	G	T	9/506/6746	0.06969	0.06956	1	0.03828	
rs8061371	16	75601178	1.344	2.986	0.002822	A	G	10/535/6735	0.07349	0.07333	1	0.0403	
rs9929146	16	75603974	1.348	3.012	0.002593	A	G	9/535/6732	0.07353	0.07312	0.7491	0.04022	
rs7190688	16	75605139	1.357	3.066	0.002173	C	T	9/529/6735	0.07273	0.07238	0.8706	0.03978	
rs12716822	16	75606315	1.357	3.066	0.002173	A	G	9/529/6735	0.07273	0.07238	0.8706	0.03978	
rs7197571	16	76980427	1.137	2.931	0.003384	C	T	763/3285/3230	0.4514	0.4426	0.09515	0.3351	WWOX
rs4502225	16	76982332	1.165	3.374	0.0007411	T	C	575/3007/3698	0.413	0.408	0.3011	0.2904	WWOX
rs7199370	16	76983291	1.162	3.304	0.0009519	T	G	538/3014/3728	0.414	0.404	0.0367	0.2858	WWOX
rs17777958	16	77148468	1.187	3.076	0.002098	G	C	165/1932/5072	0.2695	0.2657	0.2478	0.1618	WWOX
rs9923592	16	77149054	1.19	3.201	0.001371	G	A	184/2023/5072	0.2779	0.2745	0.3056	0.1685	
rs11642089	16	77159207	1.192	2.958	0.003101	A	G	111/1700/5435	0.2346	0.2301	0.102	0.1365	WWOX
rs11150090	16	77178874	1.187	2.988	0.002806	G	A	142/1787/5349	0.2455	0.2441	0.6313	0.1462	
rs4477688	16	82171973	0.8679	-3.326	0.0008813	C	T	1295/3513/2472	0.4826	0.4869	0.4413	0.4141	CDH13

rs3924280	16	83775291	1.182	2.886	0.003901	A	G	129/1717/5422	0.2362	0.2348	0.6529	0.1399	LOC123855
rs8062449	16	84607329	1.131	2.915	0.003562	G	A	1638/3531/1894	0.4999	0.4993	0.943	0.4861	
rs12598047	16	84610535	1.154	3.392	0.0006942	A	G	1088/3422/2770	0.4701	0.4733	0.569	0.3888	
rs6540246	16	84611335	1.136	2.955	0.003127	C	A	863/3233/3184	0.4441	0.4492	0.3344	0.3442	
rs13330844	16	84612387	1.132	2.863	0.004191	C	G	817/3216/3247	0.4418	0.4443	0.6349	0.3368	
rs1809844	16	84745512	1.2	3.84	0.000123	C	T	409/2730/4141	0.375	0.3686	0.1436	0.2488	
rs2086824	16	87998747	0.7663	-2.882	0.003947	C	A	39/877/6364	0.1205	0.1226	0.1504	0.06314	ANKRD11
rs4968165	17	417064	0.8738	-2.979	0.002889	A	C	785/3133/3348	0.4312	0.4378	0.1984	0.3192	VPS53
rs10521104	17	451209	0.8724	-3.014	0.002579	G	A	787/3144/3349	0.4319	0.4381	0.2286	0.3195	VPS53
rs434307	17	500830	0.8745	-2.96	0.003079	C	T	782/3136/3349	0.4315	0.4376	0.2382	0.3188	VPS53
rs1051322	17	1893253	0.6334	-3.154	0.001612	G	C	4/429/6766	0.05959	0.05886	0.4195	0.02865	DPH1 OVCA2
rs3744678	17	3582517	1.181	2.908	0.003632	C	A	153/1773/5354	0.2435	0.2448	0.6665	0.1459	ITGAE
rs11078711	17	7717939	1.158	3.392	0.0006934	T	G	1106/3380/2480	0.4852	0.4805	0.425	0.4079	
rs8074757	17	8462887	1.229	3.075	0.002108	C	G	63/1262/5954	0.1734	0.1725	0.7339	0.09758	MYH10
rs3785988	17	9065630	0.8457	-2.811	0.004945	A	G	185/1969/5115	0.2709	0.27	0.828	0.1575	NTN1
rs8082237	17	9832074	1.408	3.176	0.001493	G	C	7/430/6738	0.05993	0.05997	0.8444	0.03342	GAS7
rs1124150	17	9835846	1.464	3.807	0.0001406	T	C	9/488/6776	0.0671	0.06715	0.8613	0.03754	GAS7
rs4791946	17	10063979	1.225	3.481	0.0004999	A	G	140/1576/5312	0.2242	0.2292	0.07657	0.1369	
rs1013439	17	10068773	1.225	3.481	0.0004999	T	C	140/1576/5312	0.2242	0.2292	0.07657	0.1369	
rs9914160	17	10090243	1.196	3.194	0.001404	T	C	148/1872/5259	0.2572	0.2535	0.2292	0.1535	
rs11655347	17	12472876	0.7896	-4.443	8.86E-06	A	G	358/2519/4403	0.346	0.3456	0.9459	0.2161	
rs11655394	17	12472981	0.7915	-4.421	9.81E-06	A	G	368/2512/4400	0.3451	0.3466	0.7099	0.217	
rs734466	17	12528317	1.166	2.996	0.002735	T	G	316/2232/4113	0.3351	0.3375	0.5613	0.2191	MYOCD
rs7225754	17	12534949	1.134	2.829	0.00467	T	A	697/3081/3502	0.4232	0.4258	0.6202	0.3113	MYOCD
rs16950911	17	14893649	0.8037	-3.293	0.0009906	T	C	119/1629/5504	0.2246	0.2243	0.9582	0.1256	
rs2981611	17	16354334	1.144	3.007	0.002634	G	A	649/3111/3483	0.4295	0.4235	0.2328	0.3086	
rs4795837	17	29256668	1.141	2.827	0.004705	T	C	471/2720/4089	0.3736	0.3765	0.5134	0.2549	
rs11655712	17	36878558	1.184	3.461	0.0005377	C	A	342/2411/4527	0.3312	0.3348	0.3627	0.218	
rs6503633	17	36884664	1.14	2.852	0.004339	A	G	520/2771/3982	0.381	0.3867	0.2137	0.267	KRT35
rs10445383	17	46340442	1.56	2.926	0.00343	G	A	3/197/7073	0.02709	0.02752	0.1668	0.01518	
rs8068581	17	65309218	1.389	3.068	0.002156	A	G	6/432/6842	0.05934	0.05913	1	0.03247	
rs929647	17	66316366	0.8152	-2.846	0.004428	C	T	67/1480/5731	0.2034	0.1972	0.00735	0.1084	
rs10852739	17	67421373	0.717	-2.946	0.003223	A	G	13/643/6624	0.08832	0.08767	0.6871	0.04389	
rs7214582	17	67471558	0.7126	-3.115	0.001842	T	C	15/698/6567	0.09588	0.095	0.5361	0.04769	
rs11077586	17	67477030	0.7749	-2.995	0.002745	C	G	50/1024/6206	0.1407	0.1425	0.2841	0.07445	
rs11869595	17	67477604	0.7801	-2.896	0.003775	A	T	43/1031/6206	0.1416	0.1417	0.9342	0.07407	
rs8068400	17	67497311	0.7819	-2.876	0.004032	G	A	38/1044/6198	0.1434	0.142	0.4573	0.07434	
rs10512591	17	68391155	1.395	3.204	0.001357	G	A	10/437/6833	0.06003	0.0608	0.2472	0.03325	SLC39A11
rs667233	17	74027860	1.15	3.177	0.001489	A	G	1475/3284/1801	0.5006	0.4988	0.7854	0.4813	DNAH17
rs590040	17	74030083	1.138	3.013	0.002589	C	A	1464/3517/1970	0.506	0.4974	0.1548	0.4693	DNAH17

rs1077693	17	74782188	1.192	3.062	0.002202	T	C	150/1780/5175	0.2505	0.2499	0.8867	0.149	HRNBP3
rs9897213	17	75370959	1.173	3.034	0.002411	G	A	225/2213/4842	0.304	0.2989	0.1579	0.1862	CBX2 CBX2
rs9967286	18	804730	1.312	2.987	0.002815	G	C	19/612/6649	0.08407	0.0853	0.2147	0.04685	
rs9955849	18	3052581	0.8644	-2.965	0.003023	C	T	482/2787/4011	0.3828	0.3825	0.9756	0.2528	
rs11662443	18	3383892	1.159	3.24	0.001194	A	T	513/2888/3868	0.3973	0.3935	0.421	0.2763	
rs10853297	18	3389058	1.165	3.295	0.0009855	G	A	486/2772/3863	0.3893	0.3876	0.7366	0.2698	
rs9635855	18	3828381	0.8314	-2.939	0.003287	A	G	172/1698/5410	0.2332	0.2412	0.006424	0.1361	DLGAP1
rs9635857	18	3829680	0.8381	-2.958	0.003098	G	T	208/1878/5194	0.258	0.2655	0.01708	0.153	DLGAP1
rs3915772	18	3838826	0.832	-3.009	0.002617	C	T	195/1786/5299	0.2453	0.2542	0.003635	0.1448	DLGAP1
rs7238219	18	3842784	0.7674	-3.226	0.001255	G	A	97/975/5474	0.1489	0.1626	4.09E-10	0.08565	DLGAP1
rs11874767	18	3942917	0.7538	-2.964	0.003041	G	T	32/851/6397	0.1169	0.1178	0.4864	0.06018	
rs686307	18	6968671	0.8187	-2.822	0.004775	C	A	76/1478/5726	0.203	0.1988	0.07683	0.109	LAMA1
rs688648	18	6969240	0.8175	-2.835	0.004582	G	A	75/1463/5741	0.201	0.197	0.09553	0.1078	LAMA1
rs3852834	18	8806249	1.184	3.035	0.002402	T	C	173/1817/5290	0.2496	0.253	0.247	0.1533	KIAA0802
rs12965342	18	10416084	0.8694	-3.375	0.0007375	C	T	1904/3541/1832	0.4866	0.5	0.02294	0.4987	
rs9951929	18	12087264	1.151	2.868	0.004126	T	C	349/2450/4481	0.3365	0.3389	0.5566	0.2202	KFZp779B163
rs6505737	18	12251064	0.8818	-2.899	0.003748	T	C	1114/3491/2664	0.4803	0.4773	0.6058	0.3888	CIDEA
rs4800617	18	20886729	0.868	-2.831	0.004645	A	G	410/2704/4165	0.3715	0.3669	0.3067	0.2388	
rs9961549	18	20889347	0.8659	-2.888	0.00388	T	C	422/2694/4164	0.3701	0.3679	0.6328	0.2393	
rs12456829	18	21172006	1.158	3.278	0.001044	G	A	599/3007/3674	0.413	0.4108	0.6481	0.2936	ZNF521
rs9966413	18	24926894	1.591	2.96	0.003078	A	T	0/188/7081	0.02586	0.02553	0.6354	0.01418	
rs1966457	18	24929321	1.591	2.96	0.003078	C	A	0/188/7081	0.02586	0.02553	0.6354	0.01418	
rs2032726	18	24929659	1.591	2.96	0.003078	T	C	0/188/7081	0.02586	0.02553	0.6354	0.01418	
rs2032727	18	24929677	1.591	2.96	0.003078	A	G	0/188/7081	0.02586	0.02553	0.6354	0.01418	
rs2032728	18	24929779	1.591	2.96	0.003078	T	C	0/188/7081	0.02586	0.02553	0.6354	0.01418	
rs6506897	18	27049281	1.146	2.946	0.003218	A	G	494/2748/3964	0.3813	0.3841	0.56	0.2677	
rs6506901	18	27067565	1.159	3.085	0.002033	T	C	399/2700/4181	0.3709	0.3651	0.1776	0.2441	
rs8089598	18	27081704	1.183	3.231	0.001232	C	T	236/2175/4742	0.3041	0.3016	0.5052	0.1887	
rs7231460	18	27086185	1.183	3.231	0.001232	C	T	236/2175/4742	0.3041	0.3016	0.5052	0.1887	
rs4517864	18	27089306	1.183	3.231	0.001232	C	T	236/2175/4742	0.3041	0.3016	0.5052	0.1887	
rs4539673	18	27092767	1.183	3.231	0.001232	C	G	236/2175/4742	0.3041	0.3016	0.5052	0.1887	
rs8087103	18	27093292	1.183	3.231	0.001232	C	T	236/2175/4742	0.3041	0.3016	0.5052	0.1887	
rs4598984	18	27095395	1.183	3.231	0.001232	G	C	236/2175/4742	0.3041	0.3016	0.5052	0.1887	
rs11663924	18	27116963	1.189	3.298	0.0009752	G	A	227/2072/4967	0.2852	0.2872	0.5401	0.1776	
rs2298613	18	27311717	1.205	3.771	0.0001625	G	A	301/2360/4612	0.3245	0.3243	1	0.209	
rs1646613	18	31580770	1.135	2.984	0.002843	T	G	1484/3531/2082	0.4975	0.4965	0.8671	0.4624	
rs11872215	18	31590381	1.134	3.004	0.002661	G	A	1541/3645/2094	0.5007	0.4971	0.5556	0.4665	
rs4799808	18	31593609	1.156	3.142	0.00168	T	C	1041/3539/2083	0.5311	0.4878	3.79E-13	0.4269	
rs1942245	18	31601780	1.125	2.817	0.00485	C	T	1540/3619/2121	0.4971	0.4968	0.9812	0.4642	
rs948536	18	33379312	0.5084	-3.215	0.001303	G	A	2/234/7019	0.03225	0.03227	0.7215	0.01534	CELF4

rs9953231	18	33404524	0.5084	-3.215	0.001303	A	G	2/234/7019	0.03225	0.03227	0.7215	0.01534	
rs16971255	18	34785253	1.147	2.88	0.003974	C	G	421/2604/4255	0.3577	0.3613	0.399	0.2396	
rs16971305	18	34797900	1.148	2.909	0.003631	C	T	427/2597/4255	0.3568	0.3617	0.2435	0.24	
rs16971322	18	34809783	1.146	2.856	0.00429	T	G	421/2606/4253	0.358	0.3615	0.4176	0.2397	
rs10502716	18	34814065	1.145	2.843	0.004467	T	C	415/2602/4263	0.3574	0.3603	0.4946	0.2387	
rs17770824	18	40246400	0.786	-3.231	0.001234	T	A	73/1348/5805	0.1865	0.1854	0.6567	0.1	
rs12606539	18	40258615	0.7833	-3.271	0.001071	C	T	73/1348/5833	0.1858	0.1847	0.6566	0.09962	
rs12607379	18	40275267	0.7833	-3.271	0.001071	C	T	73/1348/5833	0.1858	0.1847	0.6566	0.09963	
rs1397988	18	40276800	0.7833	-3.271	0.001071	T	G	73/1348/5833	0.1858	0.1847	0.6566	0.09963	
rs10502829	18	40289840	0.786	-3.21	0.001327	A	C	69/1344/5813	0.186	0.1841	0.406	0.09927	
rs16978027	18	40324883	0.7872	-2.994	0.002754	C	T	63/1145/5816	0.163	0.1646	0.4248	0.08759	
rs17699979	18	40334259	0.7876	-3.008	0.00263	T	C	66/1149/5816	0.1634	0.1656	0.2795	0.08826	
rs954794	18	40335923	0.8275	-2.865	0.004176	A	G	125/1629/5238	0.233	0.2326	0.959	0.1306	
rs17700203	18	40336043	0.7876	-3.008	0.00263	G	A	66/1149/5816	0.1634	0.1656	0.2795	0.08826	
rs9946142	18	40341107	0.8233	-2.949	0.003187	A	G	126/1649/5240	0.2351	0.2343	0.8384	0.1317	
rs12326164	18	40930926	0.8751	-2.89	0.00385	G	A	726/3133/3254	0.4405	0.4368	0.4975	0.3171	
rs16978274	18	40935367	0.8751	-2.89	0.00385	G	A	726/3133/3254	0.4405	0.4368	0.4975	0.3171	
rs1450813	18	40951176	0.8774	-2.839	0.004529	G	T	723/3132/3254	0.4406	0.4366	0.4633	0.3171	
rs1450805	18	40963941	0.8774	-2.838	0.004544	G	A	723/3136/3249	0.4412	0.4369	0.4154	0.3174	
rs4890526	18	40974722	0.8732	-3.047	0.00231	G	A	973/3285/2675	0.4738	0.4699	0.4902	0.3724	
rs8096359	18	40975032	0.8688	-3.067	0.002162	G	T	865/3123/2675	0.4687	0.4631	0.3278	0.3595	
rs4890527	18	40977359	0.8732	-3.047	0.00231	C	T	973/3285/2675	0.4738	0.4699	0.4902	0.3725	
rs6507600	18	40977527	0.8732	-3.047	0.00231	T	C	973/3285/2675	0.4738	0.4699	0.4902	0.3724	
rs8088621	18	40984602	0.8732	-3.047	0.00231	A	G	973/3285/2675	0.4738	0.4699	0.4902	0.3723	
rs8089041	18	41007757	0.8753	-3.037	0.002387	A	G	975/3446/2853	0.4737	0.4667	0.2001	0.3661	
rs41471844	18	42545438	1.305	3	0.0027	A	G	11/685/6584	0.09409	0.0924	0.1281	0.05084	ST8SIA5
rs1075892	18	42547212	1.3	2.96	0.003079	T	C	13/679/6588	0.09327	0.09215	0.3721	0.05028	ST8SIA5
rs3819124	18	42681015	0.826	-2.884	0.003926	C	G	134/1563/5582	0.2147	0.2199	0.0484	0.122	PIAS2
rs10502882	18	43333631	0.8883	-2.81	0.004948	A	G	1565/3636/2076	0.4997	0.4975	0.7238	0.4605	
rs11082819	18	46144485	0.843	-2.868	0.004125	T	C	189/1969/4946	0.2772	0.2758	0.6988	0.1615	
rs7233973	18	46144874	0.843	-2.868	0.004125	T	G	189/1969/4946	0.2772	0.2758	0.6988	0.1615	
rs7407185	18	46144972	0.843	-2.868	0.004125	T	A	189/1969/4946	0.2772	0.2758	0.6988	0.1615	
rs9948601	18	46149791	0.843	-2.868	0.004125	T	C	189/1969/4946	0.2772	0.2758	0.6988	0.1615	
rs12607384	18	46150009	0.8435	-2.864	0.004178	C	G	191/1977/4938	0.2782	0.2769	0.7317	0.1623	
rs2000732	18	46150464	0.8435	-2.864	0.004178	G	A	191/1977/4938	0.2782	0.2769	0.7317	0.1624	
rs2000731	18	46150625	0.843	-2.868	0.004125	C	T	189/1969/4946	0.2772	0.2758	0.6988	0.1615	
rs4506980	18	48851652	1.406	3.154	0.00161	A	G	7/415/6858	0.05701	0.05719	0.682	0.03174	DCC
rs4368222	18	48865210	1.48	3.529	0.0004172	C	G	8/366/6867	0.05055	0.05136	0.1665	0.02886	DCC
rs4468701	18	48865278	1.48	3.529	0.0004172	T	C	8/366/6867	0.05055	0.05136	0.1665	0.02886	DCC
rs4321259	18	48877638	1.387	2.822	0.004775	A	G	7/356/6917	0.0489	0.04953	0.2375	0.02738	DCC

rs3794923	18	48981516	1.41	2.912	0.003594	T	C	7/339/6926	0.04662	0.04736	0.2047	0.02623	DCC
rs8098405	18	49006371	1.443	3.389	0.0007012	G	A	10/390/6879	0.05358	0.05474	0.08176	0.03057	DCC
rs8099160	18	49006608	1.393	3.058	0.002232	A	G	10/400/6867	0.05497	0.05605	0.136	0.03108	DCC
rs4456576	18	49016853	1.448	3.078	0.002081	G	C	7/322/6867	0.04475	0.0456	0.1149	0.02534	DCC
rs17411025	18	49046474	1.433	3.044	0.002336	G	C	7/335/6924	0.04611	0.04688	0.1996	0.02603	DCC
rs2133094	18	49718120	1.147	3.147	0.001652	T	C	1510/3533/1898	0.509	0.4984	0.07881	0.4769	
rs4131468	18	49727191	1.157	3.497	0.0004714	A	T	1750/3632/1898	0.4989	0.4998	0.8881	0.4949	
rs8088894	18	49798370	1.178	3.891	0.0001	C	A	1368/3607/2305	0.4955	0.4917	0.52	0.4417	
rs16957695	18	49802453	1.18	3.937	8.23E-05	A	T	1359/3595/2324	0.494	0.4912	0.6503	0.4398	
rs8095786	18	49803156	1.18	3.94	8.15E-05	T	C	1358/3602/2320	0.4948	0.4913	0.5509	0.44	
rs8096063	18	49803171	1.178	3.891	9.97E-05	T	G	1348/3600/2332	0.4945	0.4909	0.5348	0.4384	
rs13381484	18	49804357	1.182	3.977	6.98E-05	C	T	1348/3607/2325	0.4955	0.491	0.445	0.439	
rs2037861	18	49827692	1.135	2.947	0.003212	C	T	977/3416/2887	0.4692	0.4656	0.5128	0.3738	
rs1461533	18	49831455	1.153	3.288	0.001009	T	A	881/3333/3066	0.4578	0.455	0.6064	0.3554	
rs16957763	18	49833972	1.153	3.278	0.001047	G	T	880/3345/3054	0.4595	0.4554	0.4554	0.3561	
rs41332954	18	49841522	1.153	3.271	0.001071	A	C	880/3345/3053	0.4596	0.4554	0.4401	0.3561	
rs16957802	18	49855111	1.149	3.177	0.001487	G	A	867/3361/3052	0.4617	0.455	0.2163	0.3552	
rs1259830	18	49874899	1.133	2.895	0.003794	T	G	971/3426/2881	0.4707	0.4656	0.3516	0.3736	
rs1259836	18	49889523	1.137	2.984	0.002846	G	T	983/3452/2843	0.4743	0.4673	0.2098	0.3772	
rs2032712	18	56945896	1.262	3.337	0.0008473	T	C	54/1158/6062	0.1592	0.1589	0.9412	0.08936	
rs9961062	18	60691774	1.393	2.915	0.003563	A	G	4/386/6799	0.05369	0.0533	0.8226	0.02911	
rs11662608	18	60876992	1.163	3.4	0.0006735	G	T	851/3206/2884	0.4619	0.4571	0.4007	0.359	
rs528746	18	60899767	1.163	3.4	0.0006735	C	G	851/3206/2884	0.4619	0.4571	0.4007	0.3589	
rs597579	18	60900732	1.163	3.4	0.0006735	C	T	851/3206/2884	0.4619	0.4571	0.4007	0.3589	
rs661913	18	60902088	1.163	3.4	0.0006735	T	C	851/3206/2884	0.4619	0.4571	0.4007	0.3589	
rs3017381	18	60908588	1.157	3.308	0.0009393	C	T	881/3206/2884	0.4599	0.4587	0.855	0.3616	
rs448398	18	60915567	1.143	3.083	0.00205	C	A	937/3420/2895	0.4716	0.4636	0.1418	0.3697	
rs397581	18	60916684	1.143	3.066	0.002172	A	G	947/3298/2772	0.47	0.4662	0.5056	0.3745	
rs402144	18	60917778	1.143	3.066	0.002172	C	T	947/3298/2772	0.47	0.4662	0.5056	0.3745	
rs425251	18	60918588	1.143	3.066	0.002172	G	T	947/3298/2772	0.47	0.4662	0.5056	0.3745	
rs1108775	18	60918769	1.143	3.066	0.002172	G	A	947/3298/2772	0.47	0.4662	0.5056	0.3745	
rs176152	18	60921297	1.143	3.066	0.002172	C	T	947/3298/2772	0.47	0.4662	0.5056	0.3745	
rs176151	18	60922833	1.143	3.066	0.002172	C	T	947/3298/2772	0.47	0.4662	0.5056	0.3745	
rs176150	18	60923653	1.143	3.066	0.002172	G	A	947/3298/2772	0.47	0.4662	0.5056	0.3745	
rs176149	18	60923966	1.143	3.066	0.002172	C	T	947/3298/2772	0.47	0.4662	0.5056	0.3745	
rs176148	18	60924415	1.129	2.824	0.004749	T	C	1003/3471/2782	0.4784	0.4699	0.1339	0.3815	
rs176142	18	60927747	1.135	2.92	0.003496	T	C	950/3429/2786	0.4786	0.4672	0.04052	0.3762	
rs176140	18	60931033	1.133	2.88	0.003979	T	A	955/3429/2786	0.4782	0.4674	0.0517	0.3765	
rs176138	18	60933222	1.135	2.92	0.003496	A	T	950/3429/2786	0.4786	0.4672	0.04052	0.3762	
rs176137	18	60934033	0.8804	-2.998	0.002716	T	G	1757/3667/1769	0.5098	0.5	0.09883	0.4943	

rs176136	18	60936692	0.8805	-2.997	0.002727	T	C	1757/3651/1769	0.5087	0.5	0.1433	0.4944	
rs176125	18	60952812	0.8805	-2.997	0.002727	A	C	1757/3651/1769	0.5087	0.5	0.1433	0.4944	
rs1179815	18	60965747	0.8804	-2.998	0.002716	C	T	1757/3667/1769	0.5098	0.5	0.09883	0.4943	
rs1183856	18	60968869	0.8805	-2.997	0.002727	A	G	1757/3651/1769	0.5087	0.5	0.1433	0.4944	
rs1183855	18	60969780	0.8805	-2.997	0.002727	G	C	1757/3651/1769	0.5087	0.5	0.1433	0.4944	
rs1179812	18	60972807	0.8805	-2.997	0.002727	G	T	1757/3651/1769	0.5087	0.5	0.1433	0.4944	
rs1035208	18	60972934	0.8805	-2.997	0.002727	T	C	1757/3651/1769	0.5087	0.5	0.1433	0.4944	
rs1035207	18	60972984	0.8784	-3.072	0.002129	C	G	1778/3710/1792	0.5096	0.5	0.1058	0.4941	
rs1179809	18	60974730	0.8858	-2.891	0.003842	G	C	1810/3669/1800	0.5041	0.5	0.4967	0.4959	
rs1179808	18	60975338	1.125	2.818	0.00484	A	C	1724/3632/1920	0.4992	0.4996	0.9439	0.4914	
rs3862723	18	61007710	0.8851	-2.86	0.004234	A	T	1246/3490/2544	0.4794	0.4841	0.4104	0.4062	
rs12606922	18	68579567	1.134	2.933	0.003356	G	A	962/3189/2909	0.4517	0.462	0.06359	0.3681	NETO1
rs12606963	18	68579761	1.139	3.052	0.002275	G	A	968/3395/2917	0.4663	0.4642	0.7049	0.3721	NETO1
rs12607008	18	68579793	1.138	3.025	0.002488	A	G	967/3396/2917	0.4665	0.4641	0.6862	0.372	NETO1
rs12607048	18	68579970	1.139	3.046	0.002319	A	G	966/3387/2927	0.4652	0.4637	0.8005	0.3712	NETO1
rs2000727	18	68581327	1.139	3.043	0.002339	A	C	966/3378/2909	0.4657	0.4641	0.7808	0.372	NETO1
rs2000724	18	68581523	1.139	3.042	0.002352	A	G	972/3397/2911	0.4666	0.4645	0.7239	0.3727	NETO1
rs12966172	18	68582881	1.134	2.949	0.00319	A	G	964/3392/2917	0.4664	0.4639	0.6675	0.3716	NETO1
rs12959556	18	68588154	1.127	2.827	0.004692	A	G	1073/3489/2717	0.4793	0.4745	0.401	0.3922	NETO1
rs8083441	18	68593069	1.131	2.907	0.003652	G	A	1070/3495/2714	0.4801	0.4745	0.3231	0.3923	NETO1
rs7242528	18	68617024	1.158	3.357	0.0007879	C	G	750/3238/3292	0.4448	0.439	0.2737	0.3304	
rs7240422	18	68621700	1.154	3.286	0.001017	A	G	772/3257/3251	0.4474	0.442	0.3136	0.3346	NETO1
rs12961224	18	68627217	1.144	3.071	0.00213	C	T	750/3243/3287	0.4455	0.4393	0.2404	0.3306	NETO1
rs1529906	18	71205064	1.192	3.717	0.0002016	T	A	403/2486/4391	0.3415	0.35	0.04101	0.2317	
rs4891267	18	71205452	0.8795	-2.967	0.003008	C	A	1371/3601/2133	0.5068	0.4942	0.03268	0.4413	
rs1529905	18	71206301	1.189	3.557	0.0003746	A	G	336/2579/4365	0.3543	0.3469	0.07325	0.2285	
rs523453	20	868245	0.7805	-3.275	0.001055	C	T	71/1309/5878	0.1804	0.1799	0.8962	0.09598	
rs511582	20	868752	0.7805	-3.275	0.001055	G	A	71/1309/5878	0.1804	0.1799	0.8962	0.09599	
rs534498	20	868953	0.7727	-3.429	0.0006058	A	G	76/1324/5877	0.1819	0.1823	0.8472	0.09727	
rs6085056	20	5198158	1.342	2.898	0.00375	G	C	7/516/6568	0.07277	0.07195	0.4106	0.03882	
rs6053330	20	5274527	1.15	2.968	0.003002	G	C	435/2630/4215	0.3613	0.3652	0.3522	0.244	
rs6085118	20	5274616	1.149	2.959	0.003086	T	C	438/2626/4216	0.3607	0.3653	0.2756	0.2441	
rs6038855	20	7419576	0.8638	-3.38	0.0007243	T	C	1114/3406/2760	0.4679	0.4744	0.2358	0.3806	
rs6133429	20	7423468	0.8851	-2.853	0.004332	T	C	1193/3434/2653	0.4717	0.4799	0.1496	0.3943	
rs6086072	20	7427975	0.8667	-2.876	0.004027	C	G	454/2640/4186	0.3626	0.3686	0.1715	0.2387	
rs6056067	20	8686910	0.849	-3.546	0.0003914	T	A	715/3163/3402	0.4345	0.4319	0.6252	0.3105	PLCB1
rs6056072	20	8691803	0.877	-2.811	0.004935	A	T	635/3052/3593	0.4192	0.4175	0.7362	0.2932	PLCB1
rs737532	20	8694125	0.8501	-3.514	0.0004409	A	G	710/3154/3402	0.4341	0.4314	0.6054	0.3098	PLCB1
rs6077419	20	8696103	0.8501	-3.514	0.0004409	C	G	710/3154/3402	0.4341	0.4314	0.6054	0.3098	PLCB1
rs6077421	20	8696529	0.8501	-3.514	0.0004409	A	G	710/3154/3402	0.4341	0.4314	0.6054	0.3098	PLCB1

rs6056083	20	8697160	0.8501	-3.514	0.0004409	G	C	710/3154/3402	0.4341	0.4314	0.6054	0.3098	PLCB1
rs6056091	20	8700308	0.8501	-3.514	0.0004409	A	G	710/3154/3402	0.4341	0.4314	0.6054	0.3098	PLCB1
rs1040496	20	8708886	0.8392	-3.849	0.0001186	C	T	794/3260/3225	0.4479	0.4442	0.4928	0.3275	PLCB1
rs6056107	20	8710026	0.8397	-3.836	0.0001248	G	A	793/3260/3226	0.4479	0.4441	0.4926	0.3275	PLCB1
rs6086595	20	8712779	0.8271	-4.118	3.82E-05	T	C	738/3211/3331	0.4411	0.4366	0.3903	0.3163	PLCB1
rs718712	20	8714008	0.821	-4.275	1.91E-05	A	G	739/3204/3337	0.4401	0.4363	0.4684	0.3157	PLCB1
rs6039268	20	8714438	0.8859	-2.897	0.003771	T	C	1833/3637/1808	0.4997	0.5	0.9626	0.4961	PLCB1
rs2327088	20	8714882	0.8892	-2.82	0.004804	G	A	1846/3608/1826	0.4956	0.5	0.4533	0.496	PLCB1
rs6140722	20	8724110	0.8173	-4.38	1.19E-05	T	C	752/3208/3311	0.4412	0.4381	0.5559	0.3179	PLCB1
rs1474684	20	8724560	0.8508	-3.25	0.001156	G	A	465/2659/4135	0.3663	0.3722	0.1752	0.2436	PLCB1
rs6108193	20	8726246	0.8588	-3.115	0.00184	T	A	494/2846/3938	0.391	0.388	0.5259	0.2598	PLCB1
rs1474683	20	8729080	0.8582	-3.131	0.001742	A	G	495/2847/3938	0.3911	0.3882	0.5459	0.2598	PLCB1
rs852027	20	17030332	1.157	2.916	0.003543	G	C	288/2380/4609	0.3271	0.3237	0.4048	0.2068	
rs852072	20	17077341	0.8895	-2.816	0.004869	A	G	1776/3512/1914	0.4876	0.4998	0.04025	0.4857	
rs6081139	20	18365115	1.22	3.053	0.002267	C	G	8/1872/5155	0.2661	0.2324	6.41E-51	0.1428	C20orf12
rs6075341	20	18365508	1.22	3.053	0.002267	A	G	8/1872/5155	0.2661	0.2324	6.41E-51	0.1427	C20orf12
rs6046572	20	19981423	0.8018	-2.951	0.003166	A	G	74/1308/5516	0.1896	0.1888	0.7985	0.1024	RNKL1 C20orf2
rs7261425	20	20016635	0.8224	-2.888	0.003871	G	C	98/1565/5617	0.215	0.2126	0.3778	0.1179	C20orf26
rs4814948	20	20090889	0.7608	-3.698	0.0002176	A	G	100/1351/5140	0.205	0.2076	0.3129	0.1134	C20orf26
rs12625886	20	20958995	1.261	3.987	6.68E-05	T	C	124/1622/5534	0.2228	0.2239	0.6754	0.1326	
rs12626096	20	20959091	1.258	3.937	8.26E-05	A	G	124/1628/5528	0.2236	0.2245	0.754	0.1329	
rs12626090	20	20959236	1.265	4.043	5.28E-05	G	A	124/1627/5529	0.2235	0.2244	0.7148	0.133	
rs7262228	20	20960173	1.261	3.989	6.63E-05	T	C	124/1626/5530	0.2234	0.2243	0.7145	0.1329	
rs6082292	20	20967728	0.8701	-3.161	0.001573	T	G	1583/3267/1700	0.4988	0.4998	0.8626	0.486	
rs2184436	20	31377143	1.224	2.84	0.004513	G	A	34/1160/6086	0.1593	0.1545	0.006133	0.08745	
rs2746086	20	34185669	0.8039	-2.816	0.004859	T	G	58/1231/5945	0.1702	0.1689	0.5772	0.08979	EPB41L1
rs3887337	20	37594844	0.8817	-2.905	0.003673	T	C	1031/3394/2855	0.4662	0.4686	0.6707	0.3697	
rs292875	20	37774651	1.131	2.846	0.004433	G	A	1130/3330/2363	0.4881	0.4837	0.4679	0.4148	
rs273380	20	37785738	1.137	2.988	0.002806	A	G	1030/3471/2598	0.4889	0.4756	0.01899	0.3947	
rs11086760	20	37789942	1.137	2.988	0.002806	G	A	1030/3471/2598	0.4889	0.4756	0.01899	0.3946	
rs11906575	20	37795274	1.137	2.988	0.002806	G	A	1030/3471/2598	0.4889	0.4756	0.01899	0.3947	
rs2869898	20	37804431	1.138	3	0.0027	C	T	1030/3469/2599	0.4887	0.4756	0.02029	0.3946	
rs1408871	20	37804504	1.138	3	0.0027	T	G	1030/3469/2599	0.4887	0.4756	0.02029	0.3945	
rs4810273	20	37807091	1.138	3	0.0027	G	C	1030/3469/2599	0.4887	0.4756	0.02029	0.3946	
rs2869899	20	37808606	1.138	3	0.0027	G	A	1030/3469/2599	0.4887	0.4756	0.02029	0.3946	
rs1395368	20	37808625	1.138	3	0.0027	C	G	1030/3469/2599	0.4887	0.4756	0.02029	0.3946	
rs9784183	20	37810601	1.138	3	0.0027	G	T	1030/3469/2599	0.4887	0.4756	0.02029	0.3946	
rs12625111	20	37816220	1.138	3	0.0027	T	C	1030/3469/2599	0.4887	0.4756	0.02029	0.3946	
rs1325317	20	37821987	1.138	3	0.0027	A	G	1030/3469/2599	0.4887	0.4756	0.02029	0.3946	
rs7268285	20	37822369	1.138	3	0.0027	T	C	1030/3469/2599	0.4887	0.4756	0.02029	0.3946	

rs8121836	20	37822545	1.138	3	0.0027	A	C	1030/3469/2599	0.4887	0.4756	0.02029	0.3946	
rs716497	20	37823097	1.138	3	0.0027	G	A	1030/3469/2599	0.4887	0.4756	0.02029	0.3945	
rs12624644	20	37832268	1.138	3	0.0027	A	G	1030/3469/2599	0.4887	0.4756	0.02029	0.3947	
rs12625800	20	37832406	1.138	3	0.0027	C	T	1030/3469/2599	0.4887	0.4756	0.02029	0.3947	
rs159335	20	37840773	1.143	3.109	0.00188	C	G	1038/3583/2658	0.4922	0.4752	0.002413	0.394	
rs2076576	20	39146766	1.147	2.876	0.004034	A	C	503/2168/3898	0.33	0.3664	2.74E-15	0.2466	TOP1
rs6093729	20	40811517	0.8881	-2.844	0.004448	A	G	1781/3514/1848	0.492	0.5	0.1774	0.4897	PTPRT
rs2425789	20	44281223	1.143	2.849	0.004385	G	A	456/2699/4125	0.3707	0.373	0.6151	0.252	CDH22
rs2425792	20	44287080	1.148	2.952	0.003153	T	G	455/2695/4130	0.3702	0.3726	0.5928	0.2517	CDH22
rs16995730	20	49148516	1.777	3.147	0.00165	T	C	0/126/7149	0.01732	0.01717	1	0.00997	
rs6023096	20	52348135	1.197	3.211	0.001323	G	A	163/1801/5309	0.2476	0.2497	0.4811	0.1496	
rs331603	20	55052353	0.8281	-3.235	0.001215	G	A	202/2125/4950	0.292	0.2871	0.1531	0.1692	
rs331604	20	55052424	0.8282	-3.234	0.001221	A	G	202/2125/4951	0.292	0.2871	0.1531	0.1691	
rs6025887	20	55912403	0.8784	-2.993	0.002764	C	T	1353/3575/2229	0.4995	0.4925	0.2396	0.4329	
rs4811943	20	55915160	0.8847	-2.855	0.00431	T	C	1353/3575/2315	0.4936	0.4912	0.6843	0.4279	
rs437420	20	55916068	0.8847	-2.855	0.00431	C	T	1353/3575/2315	0.4936	0.4912	0.6843	0.4278	
rs6026574	20	56889890	0.8833	-2.813	0.004911	T	A	942/3354/2984	0.4607	0.4607	1	0.3545	GNAS
rs919197	20	56914328	0.8603	-3.469	0.000522	C	T	1118/3508/2646	0.4824	0.4779	0.4324	0.389	GNAS
rs6065006	20	58832173	1.201	3.095	0.001967	A	C	127/1579/5571	0.217	0.2202	0.2206	0.1294	
rs10485471	20	58864779	1.215	3.246	0.001172	C	G	119/1535/5605	0.2115	0.2144	0.25	0.1256	
rs2039369	20	61724265	0.8849	-2.899	0.003744	C	T	1326/3467/2487	0.4762	0.4873	0.05432	0.4156	
rs6062292	20	61734769	0.8781	-3.112	0.001861	G	T	1391/3398/2490	0.4668	0.4886	0.0001501	0.4193	
rs9980764	21	14858374	1.15	2.953	0.003149	T	C	483/2928/3798	0.4062	0.3943	0.01109	0.2732	
rs2822769	21	14860160	1.14	2.822	0.004778	C	G	532/2940/3798	0.4044	0.3991	0.2642	0.2784	
rs9980028	21	14861124	1.15	2.953	0.003149	G	C	483/2928/3798	0.4062	0.3943	0.01109	0.2732	
rs2822772	21	14861415	1.14	2.822	0.004778	A	G	532/2940/3798	0.4044	0.3991	0.2642	0.2784	
rs9983068	21	14861490	1.14	2.822	0.004778	G	T	532/2940/3798	0.4044	0.3991	0.2642	0.2784	
rs2178937	21	14864587	1.14	2.829	0.004671	A	G	532/2939/3799	0.4043	0.399	0.2768	0.2783	
rs1022446	21	14865880	1.14	2.829	0.004671	C	G	532/2939/3799	0.4043	0.399	0.2768	0.2783	
rs989938	21	14868699	1.141	2.857	0.004282	G	A	546/2958/3772	0.4065	0.4017	0.321	0.2813	
rs17241395	21	15988390	1.351	4.18	2.92E-05	G	A	40/1025/6093	0.1432	0.1425	0.7398	0.08088	
rs2823457	21	15998842	1.304	3.74	0.0001842	T	A	47/1071/6099	0.1484	0.1484	1	0.08384	
rs2823460	21	16008627	1.232	3.084	0.002042	G	A	65/1215/5999	0.1669	0.1677	0.6749	0.09492	
rs2823461	21	16009991	1.318	3.928	8.55E-05	C	T	54/1070/6064	0.1489	0.1505	0.3475	0.08518	
rs2823462	21	16011895	1.318	3.928	8.55E-05	C	T	54/1070/6064	0.1489	0.1505	0.3475	0.08518	
rs2823463	21	16012085	1.231	3.068	0.002151	C	T	67/1214/5997	0.1668	0.1681	0.5298	0.09508	
rs7283764	21	16017525	1.287	3.343	0.0008294	C	T	45/907/5930	0.1318	0.1344	0.1262	0.0752	
rs1543299	21	16019942	1.256	3.138	0.001704	A	G	48/1034/6144	0.1431	0.1442	0.5139	0.08042	
rs7277807	21	16021969	1.309	3.832	0.0001269	C	T	57/1053/6074	0.1466	0.1493	0.1327	0.08441	
rs2242676	21	16041122	1.272	3.054	0.002262	C	T	39/849/6227	0.1193	0.1218	0.09687	0.06751	USP25

rs17241416	21	16042262	1.23	3.033	0.002418	T	G	62/1201/5951	0.1665	0.1668	0.8325	0.09431	USP25
rs2823471	21	16042461	1.23	3.033	0.002418	C	T	62/1201/5951	0.1665	0.1668	0.8325	0.09431	USP25
rs17241423	21	16043366	1.23	3.048	0.002305	T	C	65/1212/5968	0.1673	0.1681	0.6749	0.0951	USP25
rs10482876	21	16043510	1.306	3.489	0.0004848	G	A	44/879/6170	0.1239	0.127	0.04877	0.07072	USP25
rs2823478	21	16063858	1.27	3.284	0.001022	T	C	43/1040/6122	0.1443	0.1441	0.9349	0.08053	USP25
rs2823481	21	16066443	1.238	3.148	0.001643	G	A	65/1207/5973	0.1666	0.1675	0.6235	0.09476	USP25
rs2823484	21	16067344	1.238	3.148	0.001643	G	A	65/1207/5973	0.1666	0.1675	0.6235	0.09476	USP25
rs2242677	21	16068199	1.27	3.284	0.001022	A	C	43/1040/6122	0.1443	0.1441	0.9349	0.08053	USP25
rs2823491	21	16076627	1.232	3.073	0.002123	C	T	65/1211/5971	0.1671	0.1679	0.6745	0.09496	USP25
rs3787577	21	16090250	1.27	3.284	0.001022	T	C	43/1040/6122	0.1443	0.1441	0.9349	0.08053	USP25
rs2404000	21	16107301	1.232	3.073	0.002123	G	A	65/1211/5971	0.1671	0.1679	0.6745	0.09496	USP25
rs17307190	21	16110715	1.274	3.312	0.0009247	C	G	42/1039/6073	0.1452	0.1447	0.8063	0.08103	USP25
rs17307197	21	16144364	1.312	3.848	0.0001191	G	A	52/1057/6091	0.1468	0.1482	0.426	0.08384	USP25
rs17307204	21	16148877	1.312	3.848	0.0001191	T	C	52/1057/6091	0.1468	0.1482	0.426	0.08384	USP25
rs8133079	21	16150021	1.214	2.856	0.004285	A	G	65/1229/5986	0.1688	0.1693	0.8353	0.09545	USP25
rs17241481	21	16162380	1.26	3.199	0.001381	G	A	51/1043/5965	0.1478	0.149	0.4718	0.08372	USP25
rs208928	21	17722590	1.966	3.306	0.0009472	C	T	0/93/7169	0.01281	0.01272	1	0.00758	
rs2824644	21	18426917	1.14	3.104	0.001911	T	G	1661/3594/1892	0.5029	0.4995	0.586	0.4887	
rs7275622	21	19999851	1.244	2.985	0.00284	C	T	43/1057/5812	0.1529	0.1517	0.5784	0.08457	
rs2826530	21	21108259	1.128	2.883	0.003945	T	A	1579/3529/2076	0.4912	0.4976	0.2755	0.4701	
rs17794389	21	21267784	1.156	2.928	0.003415	A	T	330/2573/4377	0.3534	0.3455	0.05309	0.2266	
rs2826631	21	21268319	1.199	3.981	6.85E-05	T	A	568/2986/3717	0.4107	0.4062	0.3557	0.2904	
rs2826642	21	21277621	1.16	2.933	0.003355	A	G	284/2458/4538	0.3376	0.3293	0.03264	0.2116	
rs4524185	21	23253300	0.8805	-2.927	0.003417	T	A	1108/3515/2656	0.4829	0.4774	0.3383	0.3894	
rs1015020	21	23255899	1.154	2.911	0.003606	C	A	348/2566/4366	0.3525	0.3477	0.2518	0.2277	
rs2827784	21	23261439	0.8614	-3.41	0.0006499	T	C	1079/3510/2690	0.4822	0.4755	0.2368	0.3842	
rs4816875	21	23268841	0.8693	-3.165	0.001549	T	C	1007/3485/2691	0.4852	0.4725	0.02461	0.378	
rs1448370	21	23276389	0.8663	-3.25	0.001153	C	T	1025/3485/2690	0.484	0.4733	0.0552	0.3794	
rs2026873	21	24048660	1.286	3.14	0.001689	C	T	28/831/6418	0.1142	0.1145	0.8374	0.0634	
rs1783016	21	26201909	1.142	2.877	0.004009	T	C	514/3001/3764	0.4123	0.4003	0.01086	0.281	APP
rs436587	21	26261150	1.144	3.22	0.001284	G	T	1379/3620/2281	0.4973	0.4923	0.4047	0.4443	APP
rs9305268	21	26261205	1.171	3.761	0.0001695	T	C	1599/3723/1958	0.5114	0.4988	0.03249	0.4825	APP
rs128648	21	26264863	0.8814	-3.018	0.002546	T	C	1700/3674/1906	0.5047	0.4996	0.3984	0.4802	APP
rs7283500	21	26265159	0.8847	-2.859	0.004255	C	T	1158/3470/2652	0.4766	0.4789	0.6954	0.3907	APP
rs2051174	21	26266108	0.8677	-3.329	0.0008728	C	A	1321/3625/2333	0.498	0.4903	0.1886	0.4226	APP
rs3737413	21	26270243	0.8668	-3.357	0.0007891	G	A	1339/3624/2317	0.4978	0.491	0.2421	0.425	APP
rs1783026	21	26275436	0.8823	-2.975	0.002931	T	C	1701/3741/1838	0.5139	0.4998	0.01679	0.4849	APP
rs4817076	21	26275931	0.8691	-3.299	0.0009713	C	T	1347/3634/2299	0.4992	0.4914	0.1896	0.4267	APP
rs4817077	21	26276090	0.8683	-3.319	0.0009034	A	G	1347/3641/2292	0.5001	0.4916	0.1394	0.4272	APP
rs9305274	21	26278851	0.8691	-3.297	0.0009775	T	A	1345/3638/2297	0.4997	0.4914	0.1594	0.4267	APP

rs2830003	21	26284227	0.8792	-2.923	0.003465	G	A	977/3409/2885	0.4688	0.4656	0.5624	0.3637	APP
rs9984732	21	34159219	0.7171	-2.835	0.004589	G	A	6/606/6609	0.08392	0.08192	0.04224	0.04121	ITSN1
rs9976801	21	34159478	0.7171	-2.835	0.004589	T	C	6/606/6609	0.08392	0.08192	0.04224	0.04121	ITSN1
rs2836485	21	38830283	0.8677	-3.303	0.000958	G	C	1548/3574/1891	0.5096	0.4988	0.07257	0.4693	ERG
rs9984425	21	38836178	1.145	3.013	0.002588	C	T	796/3069/2878	0.4551	0.4523	0.628	0.3508	ERG
rs4817950	21	38852353	0.8678	-3.39	0.0006984	T	C	1799/3681/1798	0.5058	0.5	0.3365	0.4942	ERG
rs457920	21	41716628	1.149	3.014	0.002577	A	G	506/2798/3975	0.3844	0.3864	0.6492	0.2662	
rs2058119	22	16102024	0.8807	-2.9	0.003732	G	A	1483/3651/1799	0.5266	0.499	4.26E-06	0.4729	
rs5749006	22	16140350	0.8589	-2.866	0.004157	A	G	334/2337/4571	0.3227	0.3289	0.1158	0.2038	
rs5752681	22	19104808	0.7329	-2.871	0.00409	A	G	21/679/6320	0.09672	0.09743	0.5389	0.04939	
rs4820621	22	23938548	1.256	3.251	0.001149	T	C	56/1121/6102	0.154	0.155	0.5455	0.08748	
rs762886	22	31478577	1.486	2.847	0.004411	A	G	2/246/7029	0.03381	0.03376	1	0.01888	SYN3
rs5998623	22	31488093	1.491	2.847	0.004415	T	C	2/240/7038	0.03297	0.03295	1	0.0183	SYN3
rs5998625	22	31489872	1.508	2.924	0.003451	T	C	2/237/7038	0.03257	0.03257	1	0.01809	SYN3
rs8136169	22	31492718	1.498	2.88	0.003977	G	T	2/239/7039	0.03283	0.03282	1	0.01825	SYN3
rs8142064	22	31504981	1.518	2.951	0.003163	T	C	2/234/7044	0.03214	0.03216	0.7206	0.01786	SYN3
rs10483165	22	31517342	1.566	3.041	0.002357	C	T	1/214/7059	0.02942	0.02925	1	0.01641	SYN3
rs7287772	22	31518307	1.567	3.044	0.002336	C	T	1/214/7056	0.02943	0.02927	1	0.01642	SYN3
rs738990	22	31519459	1.566	3.041	0.002357	A	T	1/214/7059	0.02942	0.02925	1	0.01641	SYN3
rs738944	22	33075676	1.172	2.838	0.004537	G	A	159/1874/5246	0.2575	0.2558	0.6142	0.1535	
rs204438	22	33127432	1.162	2.812	0.004918	C	G	207/2095/4978	0.2878	0.2853	0.4848	0.1756	
rs204444	22	33128028	1.164	2.854	0.004314	A	T	206/2096/4978	0.2879	0.2852	0.4348	0.1756	
rs204446	22	33128121	1.148	2.945	0.003226	A	G	464/2760/4053	0.3793	0.3784	0.8526	0.2578	
rs7287460	22	34309803	1.546	3.496	0.0004722	C	T	4/287/6989	0.03942	0.0397	0.5441	0.02211	
rs2239749	22	35661251	0.8476	-2.845	0.004444	A	G	200/2081/4998	0.2859	0.2828	0.3618	0.1672	CSF2RB
rs137978	22	38650704	1.137	2.81	0.004954	A	C	555/2781/3944	0.382	0.3916	0.03619	0.2711	GRAP2
rs5766573	22	44007730	1.14	3.097	0.001957	T	C	1142/3500/2636	0.4809	0.4789	0.7319	0.4028	C22orf9
rs5765226	22	44007924	0.84	-4.084	4.43E-05	G	A	1374/3608/2298	0.4956	0.4919	0.5357	0.4297	C22orf9
rs7288807	22	44008110	0.8364	-4.18	2.92E-05	T	G	1370/3613/2297	0.4963	0.4919	0.4601	0.4294	C22orf9
rs2294196	22	44009326	1.147	3.232	0.001231	T	C	1149/3539/2590	0.4863	0.4804	0.3055	0.4066	C22orf9
rs9615076	22	44009372	1.146	3.216	0.001298	T	C	1150/3542/2588	0.4865	0.4805	0.2942	0.4067	C22orf9
rs12169131	22	46833134	0.8444	-3.216	0.001299	G	A	340/2485/4455	0.3413	0.3402	0.8095	0.2128	
rs2688168	22	47998597	0.7849	-2.889	0.003861	G	C	44/1059/6146	0.1461	0.1457	0.9357	0.07647	
rs134461	22	48301819	0.8876	-2.846	0.004428	T	C	1481/3563/2236	0.4894	0.4946	0.368	0.4434	
rs138229	22	48899534	0.8578	-2.841	0.004498	G	A	279/2367/4634	0.3251	0.3211	0.2899	0.197	MOV10L1
