# Supplementary material for: Genetic and Metabolic Characterization of Insomnia
Source: PLoS One. 2011 Apr 6;6(4):e18455. doi: 10.1371/journal.pone.0018455 (PMC3071826; doi:10.1371/journal.pone.0018455)
Supplement: Table S2 — Functional analysis of the genes containing at least one of the SNPs as an output of the ‘Functional Clustering’ function of DAVID (http://david.abcc.ncifcrf.gov). (PDF) [file pone.0018455.s008.pdf]

Enrichment Score: 2.2980151378864107

Term	Count	PValue	Genes	Fold Enrichment
GO:0045202~synapse	11	5.45E-05	SCAMP1,DLGAP1,APP,GRIK3,SYN3,MAP1B,CYFIP1,GRIN3A,UNC13C,RIMS1,MYH1(	5.035736765
synapse	7	9.21E-04	DLGAP1,GRIK3,SYN3,CYFIP1,GRIN3A,UNC13C,RIMS1	6.187100953
cell junction	9	0.0013702	DLGAP1,CLCA2,GRIK3,SYN3,CYFIP1,ESAM,GRIN3A,UNC13C,RIMS1	4.173710402
GO:0044456~synapse part	7	0.0038731	SCAMP1,DLGAP1,GRIK3,SYN3,MAP1B,GRIN3A,RIMS1	4.603375527
GO:0030054~cell junction	9	0.0129604	DLGAP1,CLCA2,GRIK3,SYN3,CYFIP1,ESAM,GRIN3A,UNC13C,RIMS1	2.830158768
postsynaptic cell membrane	3	0.1209033	DLGAP1,GRIK3,GRIN3A	4.959501558
GO:0045211~postsynaptic membrane	3	0.1964854	DLGAP1,GRIK3,GRIN3A	3.628938372

Enrichment Score: 2.2326305260327985

Term	Count	PValue	Genes	Fold Enrichment
GO:0019226~transmission of nerve impulse	9	0.0019381	DLGAP1,APP,PDE7B,APITD1,SYN3,CNTNAP2,UNC13C,RIMS1,CACNA1A	3.919527964
GO:0007268~synaptic transmission	8	0.0032729	DLGAP1,APP,PDE7B,APITD1,SYN3,UNC13C,RIMS1,CACNA1A	4.076081369
GO:0050877~neurological system process	17	0.0045787	TECTA,DLGAP1,ADCY8,GRIN3A,RIMS1,APP,PDE7B,APITD1,SYN3,CNTNAP2,GNAS,	2.137477347
GO:0007267~cell-cell signaling	9	0.0404036	DLGAP1,APP,PDE7B,APITD1,SYN3,UNC13C,GRAP2,RIMS1,CACNA1A	2.289055483

Enrichment Score: 1.9928278543818019

Term	Count	PValue	Genes	Fold Enrichment
GO:0007155~cell adhesion	13	0.0017717	TECTA,PPFIA2,SELP,CLCA2,COL15A1,DSCAML1,PTPRT,BTBD9,NCAM1,APP,ROR2,(	2.838699525
GO:0022610~biological adhesion	13	0.0017926	TECTA,PPFIA2,SELP,CLCA2,COL15A1,DSCAML1,PTPRT,BTBD9,NCAM1,APP,ROR2,(	2.834729316
Cell adhesion	8	0.0085025	NCAM1,SELP,APP,CLCA2,COL15A1,CNTNAP2,DSCAML1,ESAM	3.433501078
GO:0009986~cell surface	7	0.0195473	PPFIA2,TECTA,NCAM1,SELP,APP,DSCAML1,PTPRT	3.249441549
GO:0016337~cell-cell adhesion	6	0.0343503	NCAM1,SELP,ROR2,DSCAML1,ESAM,PTPRT	3.293875613
hsa04514:Cell adhesion molecules (CAMs)	4	0.0608982	NCAM1,SELP,CNTNAP2,ESAM	4.27203331

Enrichment Score: 1.900073406794039

Term	Count	PValue	Genes	Fold Enrichment
GO:0022604~regulation of cell morphogenesis	6	0.0015047	MAP1B,GRLF1,CYFIP1,GAS2,CACNA1A,MYH10	7.086823289
GO:0008360~regulation of cell shape	4	0.0048413	GRLF1,CYFIP1,GAS2,MYH10	11.54889721
cell shape	3	0.007773	CYFIP1,GAS2,MYH10	22.31775701
hsa04810:Regulation of actin cytoskeleton	3	0.4433193	GRLF1,CYFIP1,MYH10	1.952219873

Enrichment Score: 1.815235674433365

Term	Count	PValue	Genes	Fold Enrichment
GO:0031175~neuron projection development	8	0.0014254	DCC,APP,MAP1B,CYFIP1,DSCAML1,GRIN3A,CACNA1A,MYH10	4.724548859
GO:0022604~regulation of cell morphogenesis	6	0.0015047	MAP1B,GRLF1,CYFIP1,GAS2,CACNA1A,MYH10	7.086823289
GO:0016358~dendrite development	4	0.0016387	APP,MAP1B,GRIN3A,CACNA1A	16.85514728
GO:0048812~neuron projection morphogenesis	7	0.002729	DCC,APP,MAP1B,CYFIP1,DSCAML1,CACNA1A,MYH10	4.960776302
GO:0030424~axon	6	0.0027832	NCAM1,DCC,APP,GRIK3,MAP1B,MYH10	6.137834037
GO:0048667~cell morphogenesis involved in neuron differentiati	7	0.0028543	DCC,APP,MAP1B,CYFIP1,DSCAML1,CACNA1A,MYH10	4.916084624
GO:0048858~cell projection morphogenesis	7	0.003327	DCC,APP,MAP1B,CYFIP1,DSCAML1,CACNA1A,MYH10	4.765811295
GO:0032990~cell part morphogenesis	7	0.0041008	DCC,APP,MAP1B,CYFIP1,DSCAML1,CACNA1A,MYH10	4.566404965
GO:0043005~neuron projection	8	0.0049256	NCAM1,DCC,APP,GRIK3,MAP1B,GRIN3A,CACNA1A,MYH10	3.766398159

GO:0000902~cell morphogenesis	8	0.0056198	DCC,APP,MAP1B,CYFIP1,DSCAML1,SOX6,CACNA1A,MYH10	3.690180174
GO:0000904~cell morphogenesis involved in differentiation	7	0.0059283	DCC,APP,MAP1B,CYFIP1,DSCAML1,CACNA1A,MYH10	4.230119328
GO:0048666~neuron development	8	0.0060781	DCC,APP,MAP1B,CYFIP1,DSCAML1,GRIN3A,CACNA1A,MYH10	3.63638746
GO:0050905~neuromuscular process	4	0.0074406	APP,GRIN3A,CACNA1A,MYH10	9.899054753
GO:0007409~axonogenesis	6	0.0088267	DCC,APP,MAP1B,CYFIP1,DSCAML1,MYH10	4.677303371
GO:0042995~cell projection	11	0.0101367	NCAM1,DCC,APP,SVIL,GRIK3,MAP1B,CYFIP1,GNAS,GRIN3A,CACNA1A,MYH10	2.53185654
GO:0030030~cell projection organization	8	0.0103885	DCC,APP,MAP1B,CYFIP1,DSCAML1,GRIN3A,CACNA1A,MYH10	3.282318155
GO:0032989~cellular component morphogenesis	8	0.0106721	DCC,APP,MAP1B,CYFIP1,DSCAML1,SOX6,CACNA1A,MYH10	3.265133243
GO:0007416~synaptogenesis	3	0.0174342	APP,MAP1B,CACNA1A	14.61657303
GO:0043025~cell soma	5	0.0193363	GRIK3,MAP1B,GRIN3A,CACNA1A,MYH10	4.817486017
GO:0050885~neuromuscular process controlling balance	3	0.0206596	APP,CACNA1A,MYH10	13.36372392
GO:0030182~neuron differentiation	8	0.0223054	DCC,APP,MAP1B,CYFIP1,DSCAML1,GRIN3A,CACNA1A,MYH10	2.815532503
GO:0050808~synapse organization	3	0.0622465	APP,MAP1B,CACNA1A	7.308286517
GO:0030425~dendrite	4	0.0809139	GRIK3,MAP1B,CACNA1A,MYH10	3.899329859
GO:0043062~extracellular structure organization	4	0.0947536	APP,HSD17B12,MAP1B,CACNA1A	3.647020172
GO:0021700~developmental maturation	3	0.138888	APP,MAP1B,CACNA1A	4.541071234
GO:0007411~axon guidance	3	0.1564219	DCC,APP,MYH10	4.213786821
GO:0044463~cell projection part	3	0.4140072	APP,GRIK3,MYH10	2.097740747
GO:0048471~perinuclear region of cytoplasm	3	0.5221904	APP,MAP1B,CYFIP1	1.702618346
GO:0015630~microtubule cytoskeleton	3	0.8560784	APP,MAP1B,MYH10	0.879937269

Enrichment Score: 1.5917505271819676

Term	Count	PValue	Genes	Fold Enrichment
GO:0032940~secretion by cell	7	0.0017788	SCAMP1,SYN3,GNAS,UNC13C,RIMS1,CACNA1A,MYH10	5.402825676
GO:0007268~synaptic transmission	8	0.0032729	DLGAP1,APP,PDE7B,APITD1,SYN3,UNC13C,RIMS1,CACNA1A	4.076081369
GO:0006887~exocytosis	5	0.0044373	SCAMP1,UNC13C,RIMS1,CACNA1A,MYH10	7.424291065
GO:0046903~secretion	7	0.0112674	SCAMP1,SYN3,GNAS,UNC13C,RIMS1,CACNA1A,MYH10	3.687063468
GO:0007269~neurotransmitter secretion	3	0.0229371	SYN3,RIMS1,CACNA1A	12.64136046
GO:0001505~regulation of neurotransmitter levels	3	0.0727926	SYN3,RIMS1,CACNA1A	6.681861958
GO:0006836~neurotransmitter transport	3	0.1073927	SYN3,RIMS1,CACNA1A	5.315117467
GO:0003001~generation of a signal involved in cell-cell signaling	3	0.1135249	SYN3,RIMS1,CACNA1A	5.139893814
GO:0046907~intracellular transport	8	0.1324225	SCAMP1,APP,AKAP12,AKAP6,GNAS,RIMS1,SH3GL2,MYH10	1.861613282
GO:0016192~vesicle-mediated transport	7	0.154111	SCAMP1,APP,UNC13C,RIMS1,CACNA1A,SH3GL2,MYH10	1.911332376

Enrichment Score: 1.5340859968846556

Term	Count	PValue	Genes	Fold Enrichment
IPR013098:Immunoglobulin I-set	7	1.44E-04	NCAM1,DCC,ROR1,ROR2,DSCAML1,ESAM,PALLD	8.746634376
IPR003598:Immunoglobulin subtype 2	8	2.37E-04	NCAM1,DCC,ROR1,ROR2,DSCAML1,ESAM,PALLD,IL1RAPL1	6.432709938
SM00408:IGc2	8	4.31E-04	NCAM1,DCC,ROR1,ROR2,DSCAML1,ESAM,PALLD,IL1RAPL1	5.690561529
domain:Ig-like C2-type 3	5	0.0050426	NCAM1,DCC,DSCAML1,PALLD,IL1RAPL1	7.20440367
Immunoglobulin domain	9	0.0070183	NCAM1,DCC,ROR1,ROR2,DSCAML1,ESAM,PTPRT,PALLD,IL1RAPL1	3.188251001
domain:Ig-like C2-type 4	4	0.0088189	NCAM1,DCC,DSCAML1,PALLD	9.356368402
GO:0009986~cell surface	7	0.0195473	PPFIA2,TECTA,NCAM1,SELP,APP,DSCAML1,PTPRT	3.249441549
domain:Ig-like C2-type 1	5	0.0231112	NCAM1,DCC,DSCAML1,PALLD,IL1RAPL1	4.594645198

domain:Ig-like C2-type 2	5	0.0234932	NCAM1,DCC,DSCAML1,PALLD,IL1RAPL1	4.571322125
GO:0016337~cell-cell adhesion	6	0.0343503	NCAM1,SELP,ROR2,DSCAML1,ESAM,PTPRT	3.293875613
domain:Fibronectin type-III 2	4	0.035036	NCAM1,DCC,DSCAML1,PTPRT	5.541848977
domain:Fibronectin type-III 1	4	0.0357178	NCAM1,DCC,DSCAML1,PTPRT	5.499544786
domain:Ig-like C2-type 5	3	0.0368574	NCAM1,DSCAML1,PALLD	9.824186822
IPR007110:Immunoglobulin-like	9	0.0455175	NCAM1,DCC,ROR1,ROR2,DSCAML1,ESAM,PTPRT,PALLD,IL1RAPL1	2.242680796
domain:Fibronectin type-III 4	3	0.0471272	DCC,DSCAML1,PTPRT	8.576671035
IPR013783:Immunoglobulin-like fold	8	0.0711525	NCAM1,DCC,ROR1,ROR2,DSCAML1,ESAM,PALLD,IL1RAPL1	2.184693941
domain:Fibronectin type-III 3	3	0.0765687	DCC,DSCAML1,PTPRT	6.510003316
IPR008957:Fibronectin, type III-like fold	4	0.0958159	NCAM1,DCC,DSCAML1,PTPRT	3.637343839
IPR003961:Fibronectin, type III	4	0.0992436	NCAM1,DCC,DSCAML1,PTPRT	3.581096254
SM00060:FN3	4	0.1274953	NCAM1,DCC,DSCAML1,PTPRT	3.16794147
IPR003599:Immunoglobulin subtype	5	0.1458657	DCC,DSCAML1,ESAM,PTPRT,IL1RAPL1	2.439370342
SM00409:IG	5	0.1932054	DCC,DSCAML1,ESAM,PTPRT,IL1RAPL1	2.157937659
disulfide bond	21	0.1971168	DCC,SELP,COL15A1,TNFRSF8,DSCAML1,PTPRT,PALLD,CSMD1,NCAM1,APP,APITD1	1.270546655
GO:0007156~homophilic cell adhesion	3	0.2087584	DSCAML1,ESAM,PTPRT	3.490524904
disulfide bond	19	0.2888856	DCC,SELP,TNFRSF8,DSCAML1,PTPRT,PALLD,CSMD1,NCAM1,APP,APITD1,TNFRSF1	1.210074874
IPR013151:Immunoglobulin	3	0.3556024	NCAM1,DSCAML1,IL1RAPL1	2.379221484

Enrichment Score: 1.4457532772113533

Term	Count	PValue	Genes	Fold Enrichment
GO:0044459~plasma membrane part	23	0.0099463	TECTA,SELP,DLGAP1,CLCA2,GRIK3,GRIN3A,RIMS1,STARD13,SLC2A8,NCAM1,APP	1.700086948
GO:0005886~plasma membrane	32	0.0311872	ADCY8,GRIK3,AKAP12,TNFRSF8,GRIN3A,RIMS1,STARD13,SLC2A8,APP,PRMT8,SY	1.380653113
cell membrane	17	0.1482859	TECTA,DLGAP1,CLCA2,GRIK3,TNFRSF8,GRIN3A,RIMS1,SLC2A8,NCAM1,PRMT8,SY	1.3929394

Enrichment Score: 1.371342181794841

Term	Count	PValue	Genes	Fold Enrichment
membrane	53	4.37E-04	SLC45A4,ADCY8,GRIK3,VPS53,GRIN3A,SLC2A8,APP,PRMT8,PIGB,CNTNAP2,NOS3	1.500115563
topological domain:Extracellular	25	0.0136388	DCC,GRIK3,TNFRSF8,DSCAML1,GRIN3A,CSMD1,SLC2A8,APP,CNTNAP2,ESAM,DPP	1.635580201
glycosylation site:N-linked (GlcNAc...)	34	0.0142102	DCC,ADCY8,GRIK3,DSCAML1,TNFRSF8,GRIN3A,CSMD1,SLC2A8,APP,EMID2,PIGB,	1.471346256
topological domain:Cytoplasmic	29	0.0155278	DCC,ADCY8,GRIK3,TNFRSF8,DSCAML1,GRIN3A,CSMD1,SLC2A8,APP,CNTNAP2,ES	1.537137334
glycoprotein	35	0.017566	DCC,ADCY8,GRIK3,DSCAML1,TNFRSF8,GRIN3A,CSMD1,SLC2A8,APP,EMID2,PIGB,	1.435555241
transmembrane region	36	0.0626918	DCC,SLC45A4,ADCY8,GRIK3,HSD17B12,DSCAML1,TNFRSF8,GRIN3A,CSMD1,SLC	1.297830925
signal peptide	25	0.0909674	DCC,GRIK3,TNFRSF8,DSCAML1,GRIN3A,CSMD1,APP,EMID2,CNTNAP2,ESAM,TM9	1.357886699
transmembrane	36	0.0910468	DCC,SLC45A4,ADCY8,GRIK3,HSD17B12,DSCAML1,TNFRSF8,GRIN3A,CSMD1,SLC	1.256600981
signal	25	0.0988669	DCC,GRIK3,TNFRSF8,DSCAML1,GRIN3A,CSMD1,APP,EMID2,CNTNAP2,ESAM,TM9	1.344038363
GO:0031224~intrinsic to membrane	40	0.1276853	DCC,SLC45A4,ADCY8,GRIK3,HSD17B12,DSCAML1,TNFRSF8,GRIN3A,CSMD1,SLC	1.177626712
GO:0016021~integral to membrane	38	0.1724771	DCC,SLC45A4,ADCY8,GRIK3,HSD17B12,DSCAML1,TNFRSF8,GRIN3A,CSMD1,SLC	1.157826387
disulfide bond	21	0.1971168	DCC,SELP,COL15A1,TNFRSF8,DSCAML1,PTPRT,PALLD,CSMD1,NCAM1,APP,APITD1	1.270546655
disulfide bond	19	0.2888856	DCC,SELP,TNFRSF8,DSCAML1,PTPRT,PALLD,CSMD1,NCAM1,APP,APITD1,TNFRSF1	1.210074874

Enrichment Score: 1.212722523303297

Term	Count	PValue	Genes	Fold Enrichment
GO:0043005~neuron projection	8	0.0049256	NCAM1,DCC,APP,GRIK3,MAP1B,GRIN3A,CACNA1A,MYH10	3.766398159

GO:0043204~perikaryon	3	0.013684	GRIK3,MAP1B,CACNA1A	16.5721519
GO:0043025~cell soma	5	0.0193363	GRIK3,MAP1B,GRIN3A,CACNA1A,MYH10	4.817486017
GO:0030425~dendrite	4	0.0809139	GRIK3,MAP1B,CACNA1A,MYH10	3.899329859
GO:0044057~regulation of system process	5	0.1296366	MYOCD,GRIK3,MAP1B,NOS3,CACNA1A	2.547550856
GO:0050804~regulation of synaptic transmission	3	0.2227239	GRIK3,MAP1B,CACNA1A	3.340930979
GO:0051969~regulation of transmission of nerve impulse	3	0.2485036	GRIK3,MAP1B,CACNA1A	3.097551901
GO:0031644~regulation of neurological system process	3	0.2626177	GRIK3,MAP1B,CACNA1A	2.979174122
