# Supplementary material for: Genetic and Metabolic Characterization of Insomnia
Source: PLoS One. 2011 Apr 6;6(4):e18455. doi: 10.1371/journal.pone.0018455 (PMC3071826; doi:10.1371/journal.pone.0018455)
Supplement: Table S3 — Primer sequences used in ChIP-PCR. (PDF) [file pone.0018455.s009.pdf]

Table S3. Primer sequences used in ChIP-PCR

Forward	Reverse	Chromosomal position	PCR product size (bp)	Gene	Antibody used in ChIP
GCTGGACAACATGAGCAAGA	CTGAAAAAGGACACCGGAAA	chr20 8685844~8686077	234	PLCB1	H3K4me1 CTCF PAX6
CGCAGGATCCCAAATTGTAT	ATGCACTGAGAAAGGCGAGT	chr20 8687668~8687900	233	PLCB1	CTCF PAX6
AGCCCAAGGTAAACGGAACT	CCTTTGTCTGAGCCATACCC	chr20 8718901~8719179	279	PLCB1	CTCF PAX6
AGGGGAAAGGCTCAGACAGCA	GCCTCTCCAGAACTGCAGCACA	chr21 26261904~26262115	212	APP	H3K4me1 H3K4me3 H3K27ac PAX6
CCTGAATGTGAAGCAGCAGA	TGCATTTTCTAAAGGGAAGTGA	chr21 26261416-26261577	162	APP	PAX6
TACTCAGGGCCTATTATT	GCAGATTGCCAGATGAGTGA	chr1 64083550-64083801	252	ROR1	H3K4me1 PAX6
CAGCATTTTTGGAGGCTGAG	AAAACAAAGCACGATGGCTTA	chr1 64087706-64088076	371	ROR1	H3K4me1 PAX6
