# Supplementary material for: Genetic and Metabolic Characterization of Insomnia
Source: PLoS One. 2011 Apr 6;6(4):e18455. doi: 10.1371/journal.pone.0018455 (PMC3071826; doi:10.1371/journal.pone.0018455)
Supplement: Table S4 — Differences of P values for the significant SNPs in ROR1 between different sex groups. (PDF) [file pone.0018455.s010.pdf]

Table S4. Differences of P values for the significant SNPs in ROR1 between different sex groups

rsNum	Chr	Position	P value	P Male	P Female
rs10889450	1	64083788	1.09E-05	3.88E-01	7.31E-06
rs11208300	1	64084120	1.16E-04	4.86E-01	6.58E-05
rs679622	1	64084235	3.74E-05	6.43E-01	1.13E-05
rs2132161	1	64086086	1.78E-03	7.44E-01	1.04E-04
rs11208302	1	64086528	1.16E-04	4.86E-01	6.58E-05
rs11208305	1	64088067	5.60E-06	3.88E-01	3.33E-06
