# Supplementary material for: Genetic and Metabolic Characterization of Insomnia
Source: PLoS One. 2011 Apr 6;6(4):e18455. doi: 10.1371/journal.pone.0018455 (PMC3071826; doi:10.1371/journal.pone.0018455)
Supplement: Table S5 — Differences of P values for the significant SNPs in PLCB1 between different sex groups. (PDF) [file pone.0018455.s011.pdf]

Table S5. Differences of P values for the significant SNPs in PLCB1 between different sex groups

rsNum	Chr	Position	P value	P Male	P Female
rs6056067	20	8686910	3.91E-04	4.05E-04	9.51E-02
rs6056072	20	8691803	4.94E-03	2.10E-03	2.90E-01
rs737532	20	8694125	4.41E-04	4.47E-04	9.91E-02
rs6077419	20	8696103	4.41E-04	4.47E-04	9.91E-02
rs6077421	20	8696529	4.41E-04	4.47E-04	9.91E-02
rs6056083	20	8697160	4.41E-04	4.47E-04	9.91E-02
rs6056091	20	8700308	4.41E-04	4.47E-04	9.91E-02
rs1040496	20	8708886	1.19E-04	1.45E-03	2.42E-02
rs6056107	20	8710026	1.25E-04	1.45E-03	2.57E-02
rs6086595	20	8712779	3.82E-05	7.64E-04	1.58E-02
rs718712	20	8714008	1.91E-05	7.25E-04	9.35E-03
rs6039268	20	8714438	3.77E-03	8.93E-02	2.72E-02
rs2327088	20	8714882	4.80E-03	8.35E-02	3.95E-02
rs6140722	20	8724110	1.19E-05	3.77E-04	7.20E-03
rs1474684	20	8724560	1.16E-03	6.95E-03	5.67E-02
rs6108193	20	8726246	1.84E-03	8.05E-03	6.63E-02
rs1474683	20	8729080	1.74E-03	8.05E-03	6.29E-02
