# Supplementary material for: Genetic and Metabolic Characterization of Insomnia
Source: PLoS One. 2011 Apr 6;6(4):e18455. doi: 10.1371/journal.pone.0018455 (PMC3071826; doi:10.1371/journal.pone.0018455)
Supplement: Table S6 — Differences of P values for the significant SNPs residing in ROR1 between different insulin levels. (PDF) [file pone.0018455.s012.pdf]

Table S6. Differences of P values for the significant SNPs residing in ROR1 between different insulin levels

rsNum	Chr	Position	P value	P High Insulin	P Low Insulin
rs10889450	1	64083788	1.09E-05	5.28E-02	1.44E-04
rs11208300	1	64084120	1.16E-04	1.41E-01	8.56E-04
rs679622	1	64084235	3.74E-05	1.08E-01	2.20E-04
rs2132161	1	64086086	1.78E-03	8.13E-02	2.05E-02
rs11208302	1	64086528	1.16E-04	1.41E-01	8.56E-04
rs11208305	1	64088067	5.60E-06	5.18E-02	1.02E-04
