# Supplementary material for: Genetic and Metabolic Characterization of Insomnia
Source: PLoS One. 2011 Apr 6;6(4):e18455. doi: 10.1371/journal.pone.0018455 (PMC3071826; doi:10.1371/journal.pone.0018455)
Supplement: Table S7 — Differences of P values for the significant SNPs residing in PLCB1 between different insulin levels. (PDF) [file pone.0018455.s013.pdf]

Table S7. Differences of P values for the significant SNPs residing in PLCB1 between different insulin levels

rsNum	Chr	Position	P value	P High Insulin	P Low Insulin
rs6056067	20	8686910	3.91E-04	4.80E-02	5.38E-04
rs6056072	20	8691803	4.94E-03	1.08E-01	5.23E-03
rs737532	20	8694125	4.41E-04	5.36E-02	5.28E-04
rs6077419	20	8696103	4.41E-04	5.36E-02	5.28E-04
rs6077421	20	8696529	4.41E-04	5.36E-02	5.28E-04
rs6056083	20	8697160	4.41E-04	5.36E-02	5.28E-04
rs6056091	20	8700308	4.41E-04	5.36E-02	5.28E-04
rs1040496	20	8708886	1.19E-04	2.52E-02	3.67E-04
rs6056107	20	8710026	1.25E-04	2.55E-02	3.85E-04
rs6086595	20	8712779	3.82E-05	1.37E-02	2.05E-04
rs718712	20	8714008	1.91E-05	1.12E-02	1.60E-04
rs6039268	20	8714438	3.77E-03	5.41E-02	1.27E-02
rs2327088	20	8714882	4.80E-03	6.64E-02	1.36E-02
rs6140722	20	8724110	1.19E-05	1.02E-03	1.95E-03
rs1474684	20	8724560	1.16E-03	2.85E-02	7.37E-03
rs6108193	20	8726246	1.84E-03	4.35E-02	7.36E-03
rs1474683	20	8729080	1.74E-03	4.17E-02	7.20E-03
